# Application of Human Plasma Targeted Lipidomics and Analysis of Toxic Elements to Capture the Metabolic Complexities of Hypothyroidism

**DOI:** 10.3390/molecules29215169

**Published:** 2024-10-31

**Authors:** Anna Błażewicz, Michał Kiełbus, Katarzyna Skórzyńska-Dziduszko, Andreas M. Grabrucker, Jacqueline Jonklaas, Piotr Sosnowski, Alicja Trzpil, Anna Kozub-Pędrak, Agnieszka Szmagara, Julia Wojnicka, Ewelina Grywalska, Agostinho Almeida

**Affiliations:** 1 Department of Pathobiochemistry and Interdisciplinary Applications of Ion Chromatography, Chair of Biomedical Sciences, Medical University of Lublin, 1 Chodźki Street, 20-093 Lublin, Poland; julia.wojnicka@umlub.pl; 2 Department of Biological Sciences, University of Limerick, V94 T9PX Limerick, Ireland; andreas.grabrucker@ul.ie; 3 Department of Experimental Hematooncology, Medical University of Lublin, Chodzki 1, 20-093 Lublin, Poland; michal.kielbus@umlub.pl; 4 Chair and Department of Biochemistry and Molecular Biology, Medical University of Lublin, Chodzki 1, 20-093 Lublin, Poland; 5 Department of Human Physiology, Medical University of Lublin, Radziwillowska 11 Str., 20-080 Lublin, Poland; katarzyna.skorzynska-dziduszko@umlub.pl; 6 Bernal Institute, University of Limerick, V94 T9PX Limerick, Ireland; 7 Health Research Institute (HRI), University of Limerick, V94 T9PX Limerick, Ireland; 8 Division of Endocrinology, Georgetown University, Washington, DC 20007, USA; jonklaaj@georgetown.edu; 9 Department of Bioanalytics, Medical University of Lublin, ul. Jaczewskiego 8b, 20-090 Lublin, Poland; piotr.sosnowski.phd@gmail.com (P.S.); alicja.trzpil@umlub.pl (A.T.); annakozub2810@gmail.com (A.K.-P.); 10 Department of Chemistry, Faculty of Medicine, Institute of Biological Sciences, The John Paul II Catholic University of Lublin, Konstantynow 1J, 20-708 Lublin, Poland; agnieszka.szmagara@kul.pl; 11 Department of Experimental Immunology, Medical University of Lublin, Chodźki 4a St., 20-093 Lublin, Poland; ewelina.grywalska@umlub.pl; 12 Associated Laboratory for Green Chemistry (LAQV) of the Network of Chemistry and Technology (REQUIMTE), Department of Chemical Sciences, Laboratory of Applied Chemistry, Faculty of Pharmacy, University of Porto, 50-313 Porto, Portugal

**Keywords:** human plasma lipidome, targeted lipidomics, toxic elements, hypothyroidism, Hashimoto disease

## Abstract

Background: Hypothyroidism (HT) affects millions worldwide and can lead to various lipid disorders. The metabolic complexity and the influence of toxic elements in autoimmune and non-autoimmune HT subtypes are not fully understood. This study aimed to investigate the relationships between plasma lipidome, toxic elements, and clinical classifications of HT in unexposed individuals. Methods: Samples were collected from 120 adults assigned to a study group with Hashimoto’s disease and non-autoimmune HT, and a healthy control group. Quantification of 145 pre-defined lipids was performed by using triple quadrupole tandem mass spectrometry (TQ MS/MS) in multiple reactions monitoring (MRM) mode via positive electrospray ionization (ESI). Levels of toxic elements were determined using inductively coupled plasma mass spectrometry (ICP-MS). Results: Significant associations between altered levels of several components of the plasma lipidome and Al, Cd, Ni, As, and Pb with HT were found. We show metabolic differences in lysophosphatidylcholines (LPC) and phosphatidylcholines (PC) between HT and controls, with distinct predicted activation patterns for lysolecithin acyltransferase and phospholipase A2. Conclusions: There are significant changes in the lipidome profiles of healthy subjects compared to euthyroid HT patients treated with L-thyroxine, which are related to the type of hypothyroidism and non-occupational exposure to toxic elements.

## 1. Introduction

Thyroid diseases are complex and often multifactorial, involving factors such as genetics, immune responses, hormonal imbalances, and environmental factors [1]. Hypothyroidism (HT) is a chronic condition characterized by a deficiency of thyroid hormones [2] and is associated with an increased risk of developing components of metabolic syndrome, such as obesity and insulin resistance [3]. Absent or inadequate treatment of HT results in harmful consequences for the human body [4]. Symptoms of HT are nonspecific and include mild to moderate weight gain, fatigue, lack of concentration, and depression.

Currently, HT affects up to 5% of the general population, while it is estimated that 5% of cases remain undiagnosed [5]. In iodine-sufficient areas, the most common cause of primary HT is autoimmune thyroiditis (Hashimoto’s disease) [6]. Thyroid hormones stimulate fatty acid synthesis (lipogenesis), triglyceride breakdown (lipolysis), fatty acid oxidation, cholesterol synthesis, and low-density lipoprotein (LDL) receptors. They modulate all pathways of lipoprotein metabolism, influence the expression of lipoprotein receptors, the production of apolipoproteins, the activity of plasma lipoprotein-modifying enzymes, and the blood concentrations of substrates for the synthesis of triglycerides (TG), such as fatty acids and glucose. Therefore, it is reasonable to evaluate the lipid profile in patients with HT, as it may be affected by altered levels of thyroid hormones [7]. 

Lipids, which play vital roles in building cell membrane structure, cell signaling, and energy storage, are sensitive indicators of metabolic changes in health and disease [8,9]. Much attention has been directed to total cholesterol (TC) and lipoprotein particles (low-density lipoproteins—LDL, high-density lipoproteins—HDL) as risk factors for serious health disorders. However, the knowledge gap regarding lipid disorders in endocrine diseases is widely recognized. In recent years, there has been increasing interest in the diagnostic potential of lipidomics [10], as it has been realized that lipidome analysis can help identify pathogenic mechanisms that contribute to the development or progression of various diseases and identify affected metabolic pathways, thus providing useful information in specifying potential drug targets [11]. Disturbed lipid metabolism plays a significant role in metabolic disorders that occur in various diseases, including obesity, diabetes, non-alcoholic fatty liver disease (NAFLD), non-alcoholic steatohepatitis (NASH), and neurodegenerative diseases [12]. Deteriorated thyroid status can lead to an unfavorable lipid profile, which can consequently increase the risk of serious conditions, particularly cardiovascular diseases [13]. 

Patients with hypothyroidism often suffer disproportionately from lipid disorders. And this has huge health and economic implications. To the best of our knowledge, human lipidome alterations have not been studied in patients with well-controlled hypothyroidism (i.e., with thyroid-stimulating hormone (TSH) levels within the normal range under the appropriate dose of levothyroxine (LT4) replacement therapy). Research shows that LT4 treatment can reduce TC and LDL-C levels in patients with subclinical HT, including those with mild impairment [14]. According to the Endocrine Society clinical practice guidelines, the lipid profile should be reassessed when the patient’s thyroid hormones are in the normal range after treatment stabilization [7]. Knowing that thyroid hormones modulate all pathways involved in lipid metabolism and that an increasing number of studies are reporting the role of specific lipid classes in endocrine pathologies, it seems reasonable and advisable to expand research and explore beyond routine cholesterol and triglyceride testing in patients with HT. There is published evidence for the rapid identification and quantification of numerous lipid compounds in plasma using commercially available metabolomic kits. Previously published studies indicating the role of lipid species involved in thyroid metabolism include a broad group of lipid molecules that can be analyzed using a targeted lipidomic approach. The targeted lipidomics focuses on the absolute quantification of pre-defined lipids. The targeted approach, although limited to the measurement of known substances of great (known) biological importance, is high-throughput because data generation and analysis are fast, straightforward, and quantitative. The number of analytical targets is often limited due to the lack of commercially available standards [15]. A study by Jiang et al. [16] found that phospholipids, such as sphingomyelins (SM), lysophosphatidylcholines (LPC), phosphatidylcholines (PC), influence the pathogenesis of Hashimoto’s thyroiditis. Zheng et al. [17] suggest that both CD3 antigen and sphingomyelin (d18:1/20:0, d16:1/22:0) could be identified as risk factors for hypothyroidism, and found the 1-(1-enyl-palmitoyl)-2-oleoyl-GPE (p-16:0/18:1), a derivative of glycerophospholipids (GPL), to be associated with decreased hypothyroidism risk. An increase in the percentage of CD3+ cells (T lymphocytes) can be observed in cases of excessive immune system activity, and as it is known, mechanistically, Hashimoto thyroiditis is characterized by a direct T-cell attack on the thyroid gland [18,19]. 

Although many chemical elements have the potential to disrupt the endocrine system of humans at ecologically relevant concentrations [20], it is still unknown to what extent such exposures to environmental chemical contaminants impact lipid levels in the human body. Numerous research articles highlight the pathophysiological significance of chemical elements such as Cd, As, Pb, Al, and Ni. They can bioaccumulate in living organisms, severely damaging various cells, tissues, and vital organs [21]. However, it is still unknown how individual lipid species are quantitatively altered in the presence of such toxic elements in the human body. 

In this context, we decided to test the hypothesis that changes in plasma lipidome related to HT occur, even in well-controlled patients on LT4 therapy and who present normal lipid profiles on routine tests (i.e., LDL-C, HDL-C, TC, and TG) [22]. Additionally, we evaluated the association of HT with altered levels of Al, As, Ni, Cd, and Pb. The aim was also to verify which specific lipid compounds differ quantitatively between healthy adults and hypothyroid individuals and whether toxic trace element levels allow differentiation between autoimmune and non-autoimmune HT subtypes. To the best of our knowledge, there are no detailed lipidome studies in patients with hypothyroidism. Likewise, there is a lack of current human biomonitoring data regarding toxic elements in relation to the Polish population. We decided to test whether, with compensated thyroid hormone levels, the lipid profile of patients differs compared to healthy subjects, and whether environmental exposure to toxic elements affects the potential differences reflected in the plasma lipidome. To this end, a modern targeted metabolomics approach aimed at the quantification of 40 acylcarnitines, 90 glycerophospholipids, and 15 sphingomyelins was combined with inductively coupled plasma mass spectrometry (ICP-MS) as the analytical technique used to determine the toxic elements at trace levels in biological samples. To analyze the dataset, Principal Component Analysis (PCA) was used combined with the Monte Carlo Feature Selection and Interdependency Discovery (MCFS-ID) method in order not to overlook potentially valuable information contained in the collected data. Focusing on selected 145 lipid species with important biological roles, we used the most widely used platforms for targeted analysis that provide high sensitivity, selectivity, and a wide dynamic range (i.e., triple quadrupole tandem mass spectrometry (TQ MS/MS) in multiple reactions monitoring (MRM) mode via positive electrospray ionization (ESI)).

## 2. Results

Detailed characteristics of patients with Hashimoto’s disease or non-autoimmune hypothyroidism (Hypo-non-Hashimoto) and the control group are presented in Table 1. The Cohort was defined as comprising the control group (Controls) and the entire HT group (see Table 1). The disease group was characterized as including the control group (Controls), along with both the autoimmune HT subgroup and the non-autoimmune HT subgroup (as detailed in Table 1). The groups were matched by age, sex, and BMI. There were no significant differences in TSH and free thyroid hormones (fT4 and fT3) levels between the two HT subgroups (which consisted of patients adequately treated with levothyroxine to maintain euthyroidism, as highlighted above) and healthy controls.

Table S1 Nomenclature of lipids used in the study and lipid abbreviations conformed to LipidMaps standards.

Based on the analysis of each lipid group or trace element content, each highlighted in separate figures, PCA correctly classified all participants into the respective group: HT patients or controls.

PCA revealed the clusters representing groups of data points (individuals) that are closely positioned in the reduced-dimensional space, indicating similar underlying characteristics or patterns. The clusters for acylcarnitines are shown in Supplementary Figure S1A, with the key compounds for patient stratification depicted in Supplementary Figure S1B–D. The variance explained by the first two principal components (PC1, PC2), used for visualization, reached 66.1%, as shown in Supplementary Figure S1E. Similarly, for glycerophospholipids, patient subgroups are shown in Supplementary Figure S2A. The essential compounds for stratification are shown in Supplementary Figure S2B–D, with PC1 and PC2 accounting for 81.4% of the variance, as shown in Supplementary Figure S2E. Sphingolipids also contributed to patient stratification, as shown in Supplementary Figure S3A. The key compounds and their impact on variance are shown in Supplementary Figure S3B–D and summarized in Supplementary Figure S3E, reaching 86.3% of the variance for the first two components. Lastly, toxic trace elements data led to comparable subgroup distinctions, depicted in Supplementary Figure S4A. The influential compounds and variance explained by the principal components are shown in Supplementary Figures S4B–D and S4E, with 89.2% of variance explained by PC1 and PC2. All lipid groups analyzed, i.e., acylcarnitines, glycerophospholipids, sphingolipids, as well as toxic trace elements played a pivotal role in stratifying participants (controls together with HT patients), showing the ability of PCA to highlight critical biomarkers across diverse biochemical domains.

In a subsequent statistical analysis step, the relationships between the distribution of clinical variables and the clustering of all individuals resulting from PCA for different groups of lipid compounds or trace elements were determined (Table 2).

Statistically significant associations (padj < 0.05) were found between the Cohort groups (Controls vs. HT patients) and all studied chemical compounds groups. Likewise, there were statistically significant associations between the disease group (Controls vs. Autoimmune HT vs. Non-autoimmune HT) and all chemical compound groups. Moreover, a significant association was observed between both TC and HDL with toxic trace element levels. These results suggest that specific clinical grouping of all examined individuals, such as (1) Controls vs. HT patients and more detailed (2) Controls vs. autoimmune HT vs. non-autoimmune HT, significantly impacts the chemical compound profiles measured across different substance groups. Particularly, strong associations with every analyzed lipid group and trace elements were observed for the Disease variable, which may indicate significant biological differences between disease groups in cellular metabolism. Additionally, HDL and total cholesterol levels showed significant associations with toxic trace elements, indicating a potential impact on individuals’ lipid profiles.

Detailed plots showing the relationship between the distribution of demographic, clinical, and biochemical variables, and the clustering of participants derived from the PCA, including PCA clustering and percentage contingency of each analyzed demographic, clinical, and biochemical variable, are shown in Supplementary Materials (Figures S5–S8).

PCA successfully segregated HT patients into two clear subgroups based on each lipid group or trace element content, each shown in separate figures. As shown in Supplementary Figure S9A, patients were clearly grouped based on the pattern of acylcarnitines, while the key compounds for stratifying these patients are illustrated in Supplementary Figure S9B–D.

The variance captured by PC1 and PC2 totaled 73.5%, as indicated in Supplementary Figure S9E. Similarly, glycerophospholipids distinguished patient subgroups, depicted in Supplementary Figure S10A, with key compounds for stratification shown in Supplementary Figure S10B–D. In this case, the principal components explained 90.8% of the variance, as detailed in Supplementary Figure S10E. Sphingolipids also played a significant role in clearly patients stratifying into separate clusters as shown in Supplementary Figure S11A, with key compounds and their contribution to variance illustrated in Supplementary Figure S11B–D. They accounted for 85.2% of the variance captured by the first two components. Furthermore, the concentration patterns of toxic trace elements allow patients to be separated into similar subgroups, outlined in Supplementary Figure S12A. The significant compounds and the variance explained by the principal components are shown in Supplementary Figure S12B–D, with a total of 86.9% of the variance captured by PC1 and PC2, as shown in Supplementary Figure S12E. Each group of lipids and toxic trace elements played a key role in stratifying patients, demonstrating the effectiveness of PCA in identifying crucial chemicals that allow us to separate the patients into distinct clusters (Table 3).

The detailed plots illustrating the correlation between the distribution of clinical variables and the clustering of all participants as determined by PCA, including PCA clustering and the percentage contingency of each analyzed clinical variable are presented in the supplementary data (Figures S13–S16).

PCA is widely used for dimensionality reduction in biological datasets that include multiple variables, such as chemical substances and their concentrations in participants, along with demographic, clinical, and biochemical features. Its primary function is to transform original variables into a new set of uncorrelated variables—principal components—that maximize variance. However, PCA has inherent limitations, especially when applied to complex and heterogeneous data. The effectiveness of PCA may be compromised on datasets with complex structures or nonlinear relationships between variables.

To overcome the limitations of PCA, we used the MCFS-ID algorithm. Unlike PCA, which focuses on variance, MCFS-ID evaluates features based on their predictive power and interdependencies without assuming linear relationships. This enables MCFS-ID to identify the most relevant features and reveal complex interactions, offering a more robust approach for analyzing complex datasets and understanding clinical outcomes.

Therefore, we used the MCFS-ID method to explore potential associations within the dataset, including between concentrations of analyzed chemical species together with trace elements and demographic, clinical, and biochemical variables. This data analysis produced a ranked list of features, highlighting those most effective for distinguishing between classes based on specific clinical characteristics.

**Figure 1:**
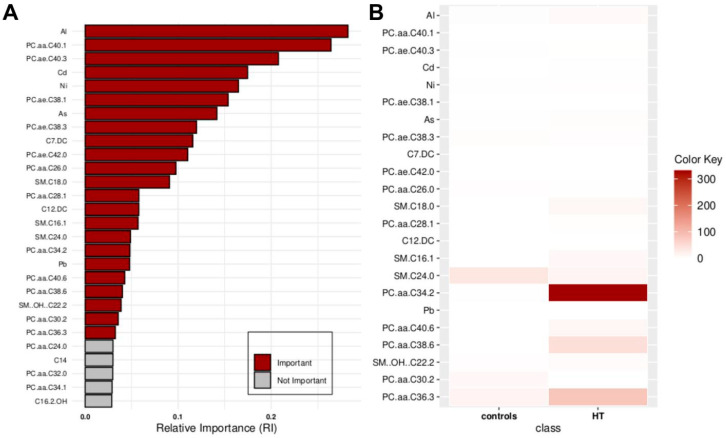
Key features identified at the top of the MCFS-ID prioritization analysis of all participants across controls vs. HT patients. (**A**) The Relative Importance (RI) of ranked features, with red color indicating significant features and gray denoting those below the cutoff (see Section 4). (**B**) The mean values of the significant features in each decision class correspond to the analyzed grouping variable.

Figure 1 shows the 23 critical features that are crucial for distinguishing controls from HT patients. These include the following: Al, PC.aa.C40.1, PC.ae.C40.3, Cd, Ni, PC.ae.C38.1, As, PC.ae.C38.3, C7.DC, PC.ae.C42.0, PC.aa.C26.0, SM.C18.0, PC.aa.C28.1, C12.DC, SM.C16.1, SM.C24.0, PC.aa.C34.2, Pb, PC.aa.C40.6, PC.aa.C38.6, SM..OH..C22.2, PC.aa.C30.2, and PC.aa.C36.3.

**Figure 2:**
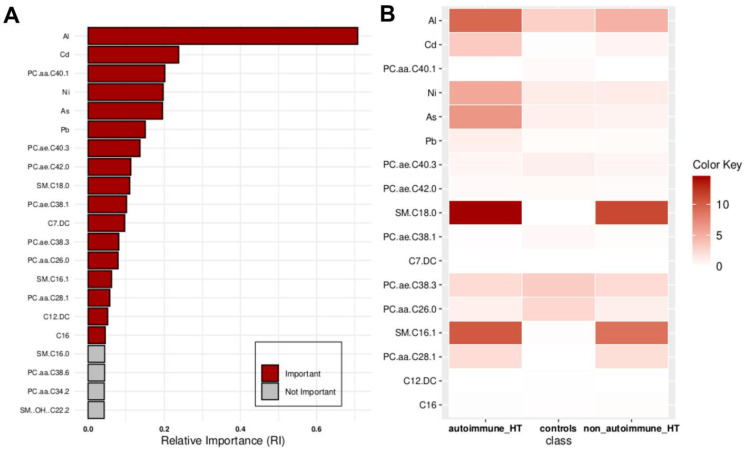
Key features identified at the top of the MCFS-ID prioritization analysis of all participants across autoimmune HT, non-autoimmune HT, and Controls. (**A**) The Relative Importance (RI) of ranked features, with red indicating significant features and gray denoting those below the cut-off point (refer to Section 4). (**B**) The average values of significant features across each decision class correspond to the analyzed grouping variable.

Figure 2 illustrates another set of 17 compounds found essential for distinguishing participants into controls, autoimmune HT, and non-autoimmune HT types, which were as follows: Al, Cd, PC.aa.C40.1, Ni, As, Pb, PC.ae.C40.3, PC.ae.C42.0, SM.C18.0, PC.ae.C38.1, C7.DC, PC.ae.C38.3, PC.aa.C26.0, SM.C16.1, PC.aa.C28.1, C12.DC, and C16. 

**Figure 3:**
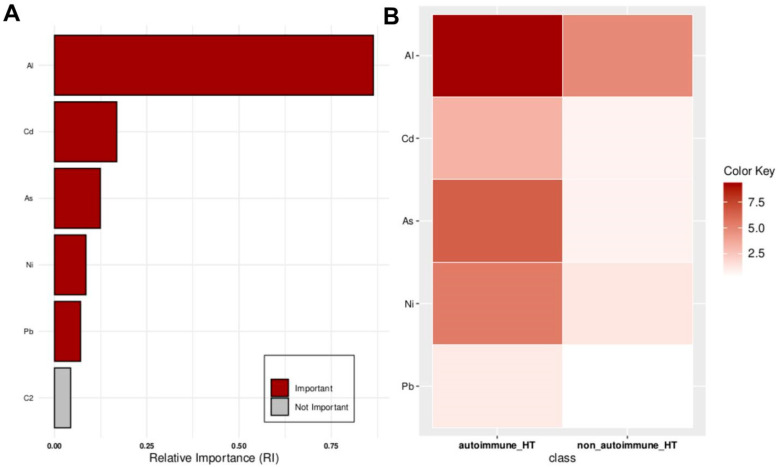
Key features identified at the top of the MCFS-ID ranking analysis of the HT patients across autoimmune HT and non-autoimmune HT. (**A**) The Relative Importance (RI) of ranked features, with red indicating significant features and gray denoting those below the cut-off point (refer to Section 4). (**B**) The average values of significant features across each decision class correspond to the analyzed grouping variable.

Focusing specifically on HT patients, the data analysis identified Al, Cd, As, Ni, and Pb as significant features for classifying the autoimmune versus non-autoimmune types of HT, as detailed in Figure 3.

To strengthen the Monte Carlo Feature Selection and Interdependency Discovery (MCFS-ID) results, a canonical statistical analysis using multiple comparisons with the Mann–Whitney–Wilcoxon test was performed. This traditional statistical analysis complements the MCFS-ID by validating differences between study groups. By using both statistical tools, we aimed to examine the data in a comprehensive way, highlighting statistically significant differences. This dual approach increases the robustness of the data analysis, increasing the credibility of the conclusions drawn.

**Figure 4:**
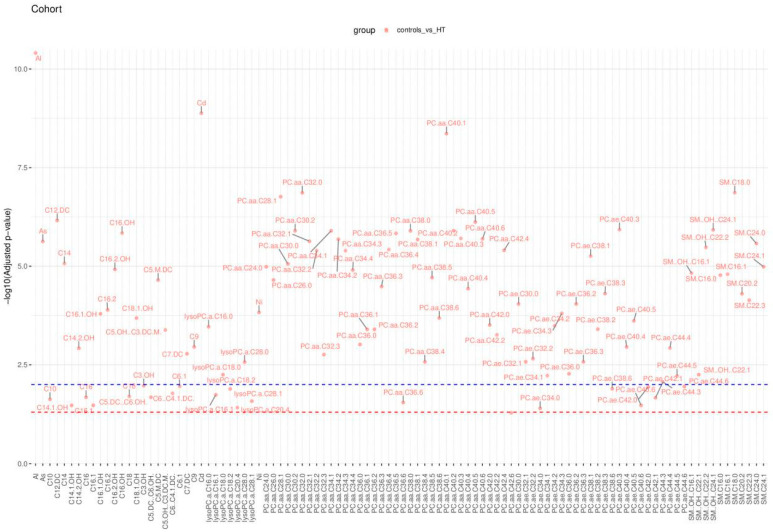
Significant comparisons of analyzed chemical substance concentrations in controls and HT patients across the controls and HT patients.

**Figure 5:**
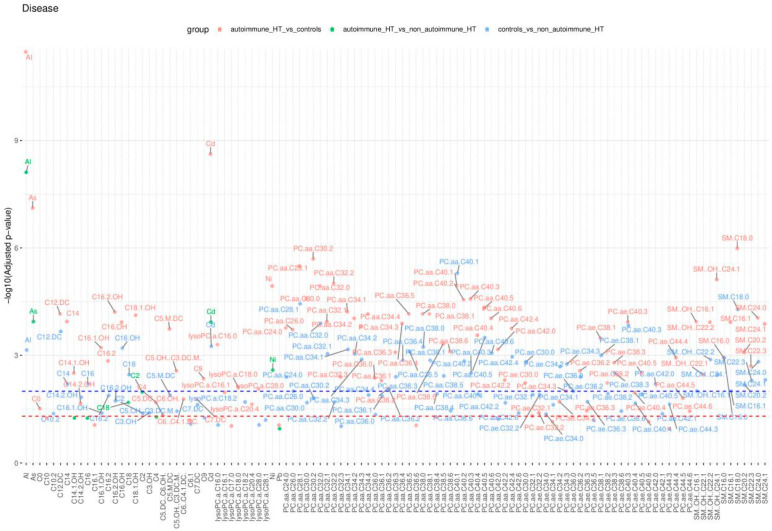
Significant comparisons of analyzed chemical substance concentrations in controls and HT patients across autoimmune HT and non-autoimmune HT patients.

**Figure 6:**
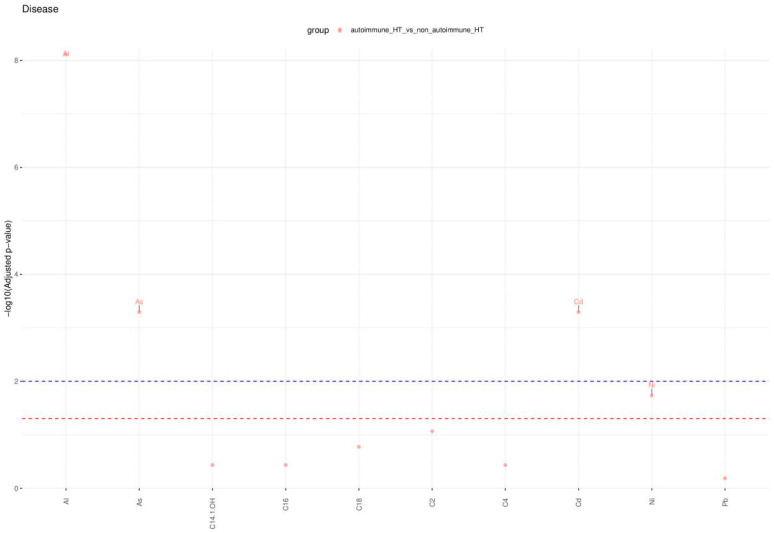
Significant comparisons of analyzed chemical substance concentrations in HT patients across disease variables.

The results of this statistical analysis are detailed in Figure 4, Figure 5 and Figure 6, which illustrate significant differences in the concentrations of chemical compounds and trace elements between participant groups, grouped by demographic, clinical, and biochemical variables. Figure 4 compares control subjects and HT patients, highlighting the differences in the biochemical profile between analyzed subgroups. Figure 5 displays these comparisons, providing insight into how compound concentrations differ by disease classification. Figure 6 focuses on HT patients only, comparing the concentrations of trace elements between autoimmune HT and non-autoimmune HT patients. These figures present a clear view of significant statistical findings, showing how the concentration of chemical compounds analyzed varies across different clinical categories. The results presented in Figure 4, Figure 5 and Figure 6 are also summarized in Table 4, Table 5 and Table 6, which highlight the most significantly altered lipids and toxic elements that differentiate between controls, autoimmune HT, and non-autoimmune HT.

The completed statistical analysis results corresponding to Figure 4, Figure 5 and Figure 6 are also attached in Supplementary Tables S2–S4 (ST2–ST4).

The Manhattan plot illustrates the −log10 adjusted *p*-values of the Mann–Whitney–Wilcoxon tests. The red and blue dashed lines depict the thresholds for adjusted *p*-values, set at 0.05 and 0.01, respectively. Only those points representing statistically significant comparisons have been marked with labels on the plot.

The Manhattan plot illustrates the −log10 adjusted *p*-values from the Mann–Whitney–Wilcoxon test to compare the concentrations of analyzed chemical compounds between the participant groups studied. The dashed red and blue lines delineate the thresholds for adjusted *p*-values at 0.05 and 0.01, respectively. Additionally, only those points representing statistically significant comparisons have been annotated with labels on the plot.

The Manhattan plot illustrates the −log10 adjusted *p*-values from the Mann–Whitney–Wilcoxon test to compare the concentrations of analyzed chemical compounds between the patient groups studied. The dashed red and blue lines delineate the thresholds for adjusted *p*-values at 0.05 and 0.01, respectively. Additionally, only those points representing statistically significant comparisons have been annotated with labels on the plot.

**Figure 7:**
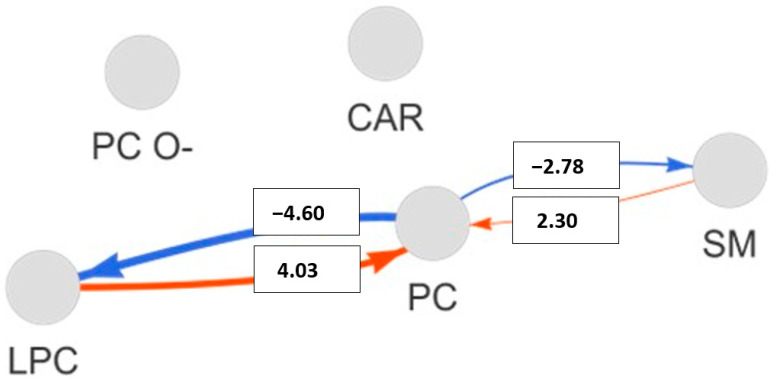
Pathway activity network of lipid classes comparing controls and HT. The figure depicts a pathway activity network connecting lipid classes, with nodes representing phosphatidylcholine (PC), acylcarnitines (CAR), lysophosphatidylcholine (LPC), and sphingomyelins (SM). Pathways between these lipid classes are illustrated by lines, with color-coding: red for pathways with positive activity values and blue for negative ones. The thickness and color intensity of the lines represent the frequency and strength of each pathway’s activity, which is quantified by a pathway activity score.

The pathway activity network analysis highlights several key lipid metabolic reactions that differentiate HT patients from healthy controls (Figure 7). The conversion of lysophosphatidylcholine (LPC) to phosphatidylcholine (PC), with a pathway score of 4.035, is mediated by the genes LPCAT1, LPCAT2, LPCAT3, LPCAT4, MBOAT1, and MBOAT2, indicating increased activity in HT. The conversion of sphingomyelin (SM) to PC, with a score of 2.297, involves SGMS1 and SGMS2, showing activation in HT. Conversely, the conversion of PC to SM, with a negative pathway score of −2.782, also involves SGMS1 and SGMS2, indicating inhibition. The reaction from PC to LPC, with the most negative score of −4.596, involves a large array of genes, including ABHD3, JMJD7-PLA2G4B, PLA2G10, PLA2G12A, PLA2G12B, PLA2G15, PLA2G1B, PLA2G2A, PLA2G2C, PLA2G2D, PLA2G2E, PLA2G2F, PLA2G3, PLA2G4A, PLA2G4B, PLA2G4C, PLA2G4D, PLA2G4E, PLA2G4F, PLA2G5, PLA2G6, PLAAT1, PLAAT2, PLAAT3, PLAAT4, PLB1, PNPLA2, PNPLA3, PNPLA4, and PNPLA8, showing significant inhibition of this pathway in HT. These findings suggest specific disruptions in lipid metabolism that are distinct in HT.

The Lipid Reaction Network analysis, applied to lipidomics data to compare metabolic differences between hypothyroidism (HT) and controls, reveals that the critical metabolic reactions between lysophosphatidylcholine (LPC) and phosphatidylcholine (PC) are regulated differently in these groups, with certain pathways being activated in HT and inhibited in controls, and vice versa. The conversion of LPC to PC is catalyzed by lysolecithin acyltransferase (enzyme 2.3.1.23, 1-acylglycerophosphocholine O-acyltransferase), with involvement from the six genes listed above. The reverse reaction, from PC to LPC, is catalyzed by phospholipase A2 (enzyme 3.1.1.4), with 32 genes involved and listed above. These pathways play a crucial role in lipid metabolism, regulating the dynamic balance between LPC and PC. The Lipid Reaction Network analysis did not identify any additional enzymes that might be differentially affected between the analyzed groups. Additionally, we used the KEGG database to annotate genes that were estimated, based on pathway activity network analysis, to be involved in the metabolism of lipids showing differential concentrations between HT and controls (Supplementary Table S5, ST5).

## 3. Discussion

Our study demonstrates the potential of targeted lipidomics, as well as biomonitoring studies in humans, to provide valuable information on the plasma lipidomic profiles and non-occupational exposure to toxic elements of the adult hypothyroid and healthy population. In addition, we present the interrelationships between individual metabolites and toxic element content depending on the type of hypothyroidism. 

Lipidomics follows the tenets of translational medicine, whose overarching goal is to transfer knowledge gained in the laboratory to everyday clinical practice. Since our aim was to focus on lipid compounds with already defined structures and important biological roles (using targeted lipidomics), we did not look for new biomarkers of hypothyroidism. Patients with both types of hypothyroidism described here manifest similar symptoms, and lipid abnormalities usually worsen during the course of the disease, regardless of the origins, although not in everyone, and not equally. Hypothyroidism is a condition that requires treatment and threatens serious health complications, including those associated with lipid disorders. This condition, as is well known, increases the risk of various cardiovascular-related diseases. Examining lipid profiles significantly accelerates the diagnosis of hypothyroidism. Normalization of thyroid hormones often also restores normal levels of plasma lipids. 

Studies report that when the concentration of thyroid hormones decreases, the processes of lipolysis are impaired, and total cholesterol, LDL fraction cholesterol, triglycerides, and lipoproteins A and B often increase. On the other hand, the concentration of HDL fraction cholesterol can be both normal and reduced or increased [23]. An interesting study by Jung et al. [24] on a group of patients with differentiated thyroid cancer who underwent total thyroidectomy and radioactive iodine (RAI) treatment showed that total cholesterol, triglycerides, low-density lipoprotein cholesterol, apoB, and the apoA-I/II ratio were significantly elevated in RAI-treated patients and returned to baseline values after levothyroxine replacement. HDL-C and apoE levels were consistently elevated despite levothyroxine substitution. Paraoxonase-1 activity, adjusted for apoA-I, decreased in the overt hypothyroid state, but returned to baseline values after levothyroxine substitution. Cholesterol efflux also decreased significantly in the overt hypothyroid state, but remained low despite restoration of thyroid function. The cited study, although included only 27 patients and described acute dynamic changes in thyroid function, confirmed that changes in thyroid function are associated with changes in the concentrations of various plasma lipid components. The current literature suggests that complex lipid metabolism abnormalities associated with hypothyroidism may involve lipid metabolism-related enzymes, receptors, and transport proteins [25].

Our study confirms that hypothyroidism is associated with important changes in the plasma lipid profile of patients beyond those already reported by other researchers regarding TSH [26], LDL-cholesterol, and TG levels [27]. Studies suggest that overt HT is a secondary cause of hyperlipidemia [28], so further insights into the human lipidome in HT patients are necessary. The use of lipidomics, and thus insights into the world of molecules such as acylcarnitines, glycerophospholipids, and sphingomyelins (overlooked in routine testing) in patients with pharmacologically balanced thyroid hormone levels, may not only help in drug selection, but may also predict some health complications. This includes also patients with Hashimoto’s thyroiditis, in which thyroid autoimmune processes affect lipid metabolism by altering thyroid hormone levels [29]. 

Most of the effects of the thyroid hormones result from their interaction with thyroid hormone receptors (THR). A recent study by Zhang et al. highlighted the role of THR in lipid metabolism [30]. The role of THR and subsequent pathways in lipid metabolism appears complex and is still under investigation. Referring to animal studies, the authors emphasize that thyroid hormones not only directly regulate lipogenic gene expression, but also influence the activity of other transcription factors, such as sterol regulatory element binding protein-1c (SREBP1c) and carbohydrate-responsive element binding protein (ChREBP), which indirectly affect hepatic lipogenesis [31]. 

In addition, thyroid hormones promote lipolysis of white adipose tissue, which is a source of circulating free fatty acids (FFAs), induce the expression of protein transporters such as fatty acid transporter protein (FATP), liver fatty acid binding protein (L-FABP) and fatty acid translocase [32]. Thyroxine (T4) and triiodothyronine (T3) exert their effects by binding to specific nuclear receptors that modulate gene expression [33]. Glycerophospholipids provide the normal cell membrane structure necessary for the function of these receptors [34]. Abnormalities in glycerophospholipid levels and structure may play a role in immune recognition and response, as changes in membrane composition can potentially affect antigen presentation and immune responses [35,36]. Importantly, oxidative damage affecting, among others, glycerophospholipids leads to lipid peroxidation and the release of inflammatory molecules, which exacerbates thyroid dysfunction [37].

It should be emphasized that the prevalence of HT in patients with hyperlipidemia reaches almost 13% [38]. In many patients, worrying signs of metabolic and hormonal imbalances can go unnoticed for a long time. Recent advances in omics techniques and methodologies make it increasingly possible to link specific lipid species and metabolic pathways to disease onset and progression [39,40,41]. Many quantitative and qualitative changes in plasma lipid species remain undetected when simply resorting to enzymatic assays routinely performed in clinical laboratories. The quantitative determination of other important lipid compounds, such as acylcarnitines, glycerophospholipids, and sphingomyelins, can provide important information about the dyslipidemia that affects patients with HT. The carnitine system allows metabolically active fatty acids to be transported across the mitochondrial membrane and thus forms an integral part of the cell energy-producing system. More specifically, the plasma acylcarnitine profile can aid in the diagnosis of fatty acid oxidation disorders [42]. Glycerophospholipids are structural components of biological membranes, essential constituents of lipoproteins, and play key roles in several cellular processes, such as signal induction and transport [43]. Sphingomyelins have important structural and functional roles in the cell, both as components of the plasma membrane and as participants in many signaling pathways [44]. 

Our results suggest that many lipid species correlate with either Hashimoto’s (autoimmune) or non-Hashimoto’s (non-autoimmune) HT. Glycerophospholipids are present in cell membranes, influencing many cellular functions [9]. Disturbed homeostasis affects ion transport. To our knowledge, no study has described a significant association of glycerophospholipids such as phosphatidylcholines: PC ae C30:1, PC ae C36:5, or PC ae C42:0 (PC O-30:1, PC O-36:5, or PC O-42:0, respectively, in accordance with abbreviations conformed to LipidMaps standards; please see Table S1) with any disease. Recently, Dong et al. reported an association of acylcarnitines, lysophospholipids, and phosphatidylcholines with BMI and type 2 diabetes (T2D) [45]. However, there is no conclusive data that glycerophospholipids directly affect the onset and development of thyroid diseases, including non-Hashimoto’s and Hashimoto’s HT. Glycerolipids are key molecules for membrane formation, energy storage, and crucial intracellular signaling processes. Triacylglycerol species, essential for normal physiology, are markers of lipotoxicity. It is reported that their excessive accumulation in adipose tissue and other organs can result in obesity, insulin resistance, steatohepatitis, and cardiomyopathy [46].

Sphingolipids, including sphingomyelins, are known to be involved in cell signaling and membrane functions, and cholesterol contributes to membrane stability, fluidity, and lipid raft formation [47]. Sphingolipids contain long-chain sphingoid bases. Sphingosine, which can be formed from palmitoyl-CoA and serine, is used by cells to form ceramides, so their quantitative measurement is crucial for understanding sphingolipid metabolism [9]. Their specific involvement in thyroid diseases such as hypothyroidism, including Hashimoto’s thyroiditis, has not yet been established. Available studies on the de novo synthesis of sphingolipids and the sphingomyelinase pathway concern, among other diseases, autoimmune encephalomyelitis [48] and arthritis [49]. To our knowledge, no research into the relationship between sphingomyelins and Hashimoto’s disease in patients undergoing treatment with levothyroxine and in euthyroid status has been published.

Wong et al. presented the first studies to evaluate acylcarnitine species in individuals with hypo- or hyperthyroidism [50]. The authors suggested that a serum acylcarnitine profile, used as a diagnostic test for metabolic myopathies, would have similar sensitivity and specificity in patients with treated or untreated thyroid disorders. However, the power of the study was very low due to the evaluation of only 12 patients (6 with hypothyroidism) and the lack of a control group. The authors acknowledged that larger-scale studies are needed to test whether differences in the profiles of these molecules truly represent reproducible pathophysiological changes associated with a given form and/or severity of thyroid disease. As acylcarnitine is responsible for carrying out the beta-oxidation of fatty acids, one of the most important pathways for metabolic energy production [51], studies in populations with metabolic and endocrine diseases should be carried out in order to make explicit the changes identified in acylcarnitine profiles. 

It is unknown whether the levels of the specific molecules such as those described above, as a result of compensating for hormonal deficiencies with a dose of synthetic hormone, also return to normal in hypothyroid patients. Future research directions that stand out in the area of hypothyroidism today focus, among other things, on understanding the detailed mechanisms of TSH involvement in lipid metabolism and its broader implications for various disorders (including, for example, neuropsychiatric) [52]. Our lipidomic study, based on a selected group of patients with normal (stabilized) TSH levels, will help other researchers widen the search for solutions to these complex mechanisms. Taking into consideration that blood plasma contains plenty of distinct lipid molecular species, searching for an explanation by conducting only partial lipid analysis (such as these tested during routine analyses) may not be sufficient. The role of individual lipid metabolites belonging to various lipid classes and how their levels change in response to hormonal therapy remain largely unknown. We consider that it would be beneficial to supplement our studies with research into other thyroid diseases and various treatments for their pathologies, which could provide in-depth insights into thyroid metabolism and pathogenesis. 

Both the synthesis, transport, and metabolism of thyroid hormones can be modified by various toxic substances, which can disrupt the functioning of hormone receptors and contribute to the development of autoimmunity [53]. It is shown that some toxic elements (including Al, Pb, As, Cd, and Ni determined in our study) are capable of interfering with hormone action and have been recognized to impact thyroid function and health a long time ago [54].

Our study shows the content of toxic elements in people with normal levels of fT3, fT4, and TSH in relation to health and disease state, suggesting the great importance of small trace levels of the toxins analyzed. As we have shown the hormonal changes are not out of the norm at the plasma concentrations of Al, As, Cd, Pb, and Ni we detected, but the effects of these toxic elements on the lipidome are revealed even at such low concentrations. To our knowledge, the present study is the first to demonstrate a significant association between multiple components of the human lipidome and toxic substances in both non-autoimmune and autoimmune HT. Exposure to toxic trace elements, such as Cd, As, Al, Pb, and Ni, is a public health problem worldwide, so they were determined in samples collected from all participants in our study. A considerable body of evidence documents the deleterious human health effects of toxic trace elements and although high levels of several metals have been linked to different disease processes, the exact mechanisms underlying many of these conditions are not completely understood [55,56,57]. It is known that toxic trace elements can substitute essential elements in the body (e.g., Ca may be substituted by Pb or Al, Zn by Cd, etc.) [58], inhibit the activity of various enzymes, and induce the production of oxidative stress through increased formation of free radicals and other reactive species [59]. Thus, toxic trace elements can affect all organs, mainly the heart, kidneys, liver, lungs, and nervous system, through DNA damage, lipid peroxidation, and protein sulfhydryl depletion [60]. Membrane phospholipids, containing high levels of polyunsaturated fatty acids, are predominantly susceptible to peroxidation damage [61]. It should be noted that chronic low-level exposure to many toxic metals has been associated with an increased risk for the same health problems found in individuals occupationally exposed to much higher levels [62]. Some epidemiological data highlight the association of exposure to toxic metals with dyslipidemia and a particularly increased risk of cardiovascular diseases [59]. Research on blood lipid profile abnormalities in Cd-exposed workers in China revealed that high concentrations were associated with an increased prevalence of dyslipidemia [63]. The accumulation of Cd in hepatocytes can inhibit the activity of gluconeogenesis enzymes, which affects lipid synthesis pathways and consequently blood lipid profiles [64]. Excessive hepatic gluconeogenesis promotes excessive lipid synthesis in insulin-resistant hepatocytes and is an important factor in the development of dyslipidemia [65]. Rats exposed to Cd exhibited progressive dyslipidemia characterized by increased serum levels of FFA, TC, VLDL and LDL fractions, and decreased HDL fraction. The reported results have shown features similar to human metabolic syndrome [66]. 

The effects of As exposure were also studied in rats, and a decrease in HDL-C and HDL-C/LDL-C ratio was observed, with an increase in TG, TC, and LDL-C [67]. Arsenic is known to generate reactive oxygen species (ROS), resulting in oxidative stress, which causes DNA damage, alters ion channel function, and increases lipid peroxidation. In addition, As affects the lipolysis process through G-protein-coupled receptors by activating hormone-sensitive lipase, decreasing lipid storage, and increasing plasma lipids, further promoting atherosclerosis and cardiovascular disease [68]. Results from animal studies have shown significant effects of arsenic on cholesterol and lipid metabolism, while suggesting that arsenic-induced health effects may be attenuated by modulation of the gut microflora [69]. Interestingly, the composition of the gut microbiota affects the availability of essential micronutrients such as iodine, selenium, and iron for the thyroid gland. Moreover, gut microbial imbalance (dysbiosis) may promote autoimmune diseases such as autoimmune thyroid disease [69]. Unfortunately, so far we have not found human studies supporting a link between arsenic exposure and dysbiosis in people with thyroid diseases.

Lead can induce the synthesis of ROS, which results in oxidative stress in the cell, decreasing the levels of antioxidants and cytosolic calcium. Pb exposure upregulates lipid peroxidation, which is directly related to membrane tissue damage [70]. Rehman studied the potential of Pb to induce lipid peroxidation in erythrocytes, which are highly vulnerable to this damage [61]. It was found that the phospholipids composition of erythrocyte membranes was highly affected by Pb exposure. It has been reported that, in battery manufacturing workers, increased TC/HDL-C and LDL-C/HDL-C ratios were associated with Pb exposure, which in turn led to an increased risk of cardiovascular diseases in that population [9].

Aluminum interference with iron-dependent enzymatic activities in the tricarboxylic acid cycle and electron transport chain results in decreased mitochondrial ATP production, contributing to iron-mediated oxidative stress and lipid peroxidation. This and the modulation of α-ketoglutarate and L-carnitine disrupt lipid metabolism, leading to dyslipidemia. The effect of Al exposure on glycolysis causes, among other effects, fat accumulation and an increase in the pro-inflammatory response. Metalloestrogenic properties have been attributed to Al, as it can modulate estrogen receptors [71]. Studies suggest that Al increases lipid biosynthesis and secretion. Furthermore, it hinders the normal physiological estrogen pathway, leading to severe dyslipidemia [72].

Nickel exposure can induce oxidative stress by inducing ROS production in cells, and ROS causes lipid peroxidation. This process damages cell membranes and alters lipid composition, disrupting lipid metabolism. Lipid peroxidation products can further exacerbate oxidative stress and inflammation, further contributing to lipid dysregulation [73]. Polyunsaturated fatty acids are especially susceptible to this damage [74]. The association between Ni exposure and blood lipids in humans remains largely unclear, and studies on the effect of Ni on human blood lipids are still scarce [75]. According to Wang et al., who identified BMI as an important indicator of the relationship between exposure to Ni and serum lipid profiles (TC, non-HDL-C, and TC/HDL-C ratio) in humans, Ni can block key sulfhydryl groups of enzymes involved in fatty acid synthesis, thus inhibiting their activity [76]. Recent studies in the NHANES general population have indicated a linear association of Ni exposure with serum TC and LDL-C, and a nonlinear association with HDL-C [77]. Nickel exposure can trigger an inflammatory response in the cells and tissues. It can activate signaling pathways involved in inflammation, such as nuclear factor kappa B (NF-κB) and mitogen-activated protein kinase (MAPK) [78]. These molecules, released in response to Ni exposure, may interfere with cellular processes, including those related to lipid metabolism, by inhibiting insulin signaling, promoting lipogenesis, and impairing lipid oxidation, but no data are available at present. Ni exposure can also interfere with lipid-derived signaling molecules, such as prostaglandins and leukotrienes, and induce inflammation [79].

Considering all the available toxicological evidence and the ubiquity of toxic metals in the human environment, future directions of research on toxic metals and other toxins affecting the thyroid gland are aimed at identifying their harmful effects, developing risk assessment methods, and explaining the mechanisms of action in populations exposed to the frequent complications of comorbidities. Our study contributes to the understanding of lipid disorders in hypothyroid patients and should be replicated on a larger number of patients also with other thyroid pathologies, preferably at different time points.

### Strengths and Limitations of the Study

This study represents a valuable approach to clarify possible links between lipid metabolism, toxic trace elements exposure, and hypothyroidism. In addition to the compounds currently analyzed for the purposes of clinical diagnosis and monitoring of dyslipidemias (i.e., triglycerides, cholesterol), human plasma contains thousands of other lipid molecular species, such as acylcarnitines, glycerophospholipids, sphingolipids, etc. Lipidomics studies, such as the one presented here, will contribute to deepening the understanding of the functions of different lipids in normal physiological conditions as well as their association with specific diseases. We have identified significant differences in both plasma lipidome and toxic element content in HT patients compared with healthy subjects, as well as in different subtypes of autoimmune and non-autoimmune HT. Our results demonstrate that plasma lipidomic studies can further contribute to the understanding of complex metabolism in HT.

A strength of the work was the fulfillment of strict criteria for the qualification of participants. The patient group and control group were well matched regarding age and BMI and there were also no statistically significant differences between groups in the serum levels of TC, LDL-C, HDL-C, and TG. Furthermore, common comorbidities most often associated with hypothyroidism, such as insulin resistance, diabetes, and cardiovascular disease, were excluded and it was also ensured that there were well-controlled levels of thyroid hormones in the patient group, thus allowing us to assume that lipidome changes observed are typical of hypothyroidism itself, even when therapeutically compensated.

We also addressed the issue of important metabolic pathways in the context of hypothyroidism. The key pathways include those related to lipid metabolism (e.g., glycerophospholipids, linoleic acid, and ether lipid metabolism), as thyroid hormones regulate lipid synthesis and degradation [80]. Hormonal pathways, such as ovarian steroidogenesis, GnRH, and oxytocin signaling, are also important due to the hormonal imbalances often observed in thyroid dysfunction [81]. In addition, pathways such as MAPK and Ras signaling are important because they are involved in metabolism and cell growth, which can be influenced by thyroid hormone levels in certain cell types [82]. Pathways related to the immune system (e.g., Fc gamma R-dependent phagocytosis) may be important, especially in the context of autoimmune thyroid diseases [83,84,85]. Moreover, processes such as mitochondrial biogenesis, membrane transport, and lipid biosynthesis are crucial, as thyroid hormones play a significant role in regulating cellular energy production, membrane integrity, and the synthesis of essential lipids [80,86,87]. Pathways related to acylglycerol degradation and triacylglycerol biosynthesis are critical for lipid homeostasis, as thyroid hormones regulate the breakdown and synthesis of fats [80]. Ferroptosis, a form of cell death caused by iron-dependent lipid peroxidation, is not directly related to hypothyroidism; however, thyroid cancer, lipid, and iron metabolism disorders may sensitize cells to this process, especially under conditions of oxidative stress associated with thyroid dysfunction [88].

When it comes to the relationship between toxic trace elements and hypothyroidism it is an area of growing research interest. Exposure to these elements has been associated with various adverse health effects, including potential impacts on thyroid function. The present study is the first to demonstrate a significant association of toxic trace elements with the human lipidome profile in patients with hypothyroidism (both non-autoimmune and autoimmune forms).

Some limitations of the study are also acknowledged. First, we cannot rule out reverse causality in this cross-sectional study. Thus, whether differences in toxic trace element levels are a cause or consequence of hypothyroidism and changes in the lipidome is an open question. Second, we collected data at a single time point. Thus, the data (level of toxic trace elements and lipid profile) reflect the current patient’s status while hypothyroidism generally develops slowly, often with an evolution of several years. Third, potential confounders, such as other chemicals, may have influenced the observed associations. Future research should address the identified limitations. 

In our study, we determined lipid molecules in human plasma and also used this biological specimen to determine selected toxic trace elements. We are aware that most of the elements analyzed are usually measured in whole blood samples (not plasma). However, for example, for Pb, some studies suggest that plasma levels may better reflect the toxicologically labile fraction of circulating Pb, which will be more available for exchange with target tissues, than Pb levels in whole blood [89].

In most cases, human biomonitoring does not distinguish between natural and human-caused sources, but it is a reliable tool for assessing human exposure to chemicals from different sources, through different pathways, and at specific times in life [90]. Current data on biomonitoring of the general population in Poland, aimed at establishing baseline concentrations of the chemical elements in the Polish population and tracking trends in concentrations over time, are not available. Our measurements do not indicate the source or route of exposure to a given chemical element. The content we measured represents the amount that entered the body through any or all routes of exposure (such as ingestion, inhalation, or skin contact) and from any or all sources (such as water, air, soil, food, and consumer products). It should be emphasized that the presence of Al, As, Ni, Cd, and Pb in the human body may result from exposure to a single source or multiple sources. Any exposure assessment in future studies should include consideration of the size and nature of exposed populations, as well as the magnitude, frequency, duration, and routes of exposure.

## 4. Materials and Methods

### 4.1. Patients and Control Group

The study protocol received ethical approval from the Bioethics Committee of the Medical University of Lublin (KE-0254/7/2021). Written informed consent was obtained from each participant. The study was conducted in accordance with the principles of the Declaration of Helsinki. Study participants were recruited for the project entitled “Study of endocrine disrupting substances, elemental and lipid profiles in biological fluids and tissues in people with endocrine disorders”) in the period 2021–2023 at the Medical University of Lublin. One hundred and twenty participants without occupational exposure to metals were selected and assigned to two groups: the study group (*n* = 59), individuals diagnosed with either Hashimoto’s disease (*n* = 32) or non-autoimmune hypothyroidism (hypo-non-Hashimoto, *n* = 27) according to endocrinological assessment; and the control group, consisting of 61 healthy volunteers with normal thyroid function. Participant characteristics are presented in Table 1.

Study subjects were recruited from a specialized thyroid outpatient unit observing higher frequencies of thyroid disorders than the general population. Thyroid-stimulating hormone (TSH), free T4 (fT4), free T3 (fT3), lipid profile, thyroid peroxidase antibody (TPOAb), and antithyroglobulin antibody (TgAb) were assessed to confirm autoimmune thyroiditis in patients with Hashimoto’s disease. All patients, whether with Hashimoto’s disease or non-autoimmune HT, were receiving thyroid hormone replacement therapy with levothyroxine tablets (Euthyrox^®^) at a stable dose of at least 75 µg/day for at least 1 year. A stable euthyroid state was defined as a stable serum TSH level within the reference range of 0.4–4.0 µIU/mL. LT4 doses were carefully selected (based on information such as the patient’s weight, age, and other medical conditions) to maintain euthyroidism. There were no differences in TSH, fT4, and fT3 levels between the patient group and healthy controls (see Table 1 in Section 2).

Study participants were not receiving any other medical treatment. Aware that comorbidities can significantly modify the individual metabolic profile, patients with common diseases such as diabetes, insulin resistance, liver disease, and hypertension were excluded. Participants had not taken mineral or vitamin supplements for at least three months before collecting the samples for analysis, nor did they follow any special diet beforehand. Importantly in the context of toxic metal exposure studies, none of the participants were active smokers in the six months prior to the study. 

All participants (study group and control group) were residents of the same geographic area (central and south-eastern Poland, a predominantly agricultural region). None of the study participants had occupational exposure to toxic metals.

Participants were categorized by body mass index (BMI) values according to US Centres for Disease Control and Prevention (CDC) criteria for adults as “underweight”, “healthy_weight”, “overweight”, and “obesity” [91]. Two weight groups were considered: “underweight_or_healthy weight” and “overweight_or_obesity”.

### 4.2. Sample Collection

Blood samples (about 4 mL) were collected after overnight fasting into commercially available collection tubes (BD Vacutainer™ plastic blood collection tubes for trace element testing with K_2_EDTA, Thermo Fisher Scientific, Waltham, MA, USA). Plasma was separated from cells by centrifugation for 10 min at 1800× *g* using a refrigerated centrifuge. Samples were stored in plastic vials at a temperature below −80 °C. Plasma samples were used for metabolomic analysis and quantitative measurements of toxic trace elements. None of the samples showed signs of hemolysis, which could affect the measurement results. 

TC and TG levels were determined with commercial enzymatic kits and HDL-C was measured by immunoassay in serum samples from routine analysis. LDL-C was calculated using the Friedewald formula: LDL-C = TC − HDL-C − TG/5 [92]. Thyroid function parameters, including free T3 (fT3; normal range: 1.5–4.0 ng/L), free T4 (fT4; normal range: 0.8–1.8 ng/dL), and TSH (normal range: 0.4–4.0 µIU/mL), were measured using an Immunoassay Analyser Alinity I (Abbott Laboratories, Abbott Park, IL, USA). 

Samples from patients and controls were collected and analyzed under exactly the same conditions.

### 4.3. Targeted Metabolomic Analysis

Targeted Metabolomic Analysis (TMA) was used because it is an analytical approach with better quantitative analytical capabilities than untargeted approaches, where the metabolites, namely the lipid species of interest, are not predefined [93]. Quantification of a broad panel of metabolites, such as 40 acylcarnitines, 90 glycerophospholipids (phosphatidylcholines—PC, lysophosphatidylcholines—lysoPC), and 15 sphingomyelins (SM), was performed using an AbsoluteIDQ^®^ p180 kit (Biocrates Life Sciences AG, Innsbruck, Austria). The purchased kits were stored at –80 °C and were prepared within one working day. Sample preparation was performed, following the manufacturer’s recommended protocol. Blood plasma (10 µL) was pipetted onto the kit filter plate. Then, 10 µL internal standards were added and the plate was dried under a nitrogen stream. Phenylisothiocyanate was used as a derivatization reagent and the samples were then dried again under a nitrogen stream. Metabolites were extracted using 5 mM ammonium acetate in methanol. After dilution, the samples were introduced by direct flow injection analysis (FIA, injection volume: 20 μL) and analyzed using a triple quadrupole mass spectrometer (6470 QQQ triple quadrupole, Agilent Technologies, Santa Clara, CA, USA). FIA-ESI-MS/MS (QQQ) was performed in positive ion mode. The automation of the flow injection was realized with HPLC equipment (Agilent 1290 Infinity II LC system). 

Acquisition methods were set as recommended by the AbsoluteIDQ^®^ p180 kit. Data acquisition was performed by MassHunter Data Acquisition for LC/TQ B.10.0 (Agilent Technologies) and data analysis was performed using MetIDQ™ (Biocrates) and Mass Hunter Quantitative Analysis v.10.2 software (Agilent Technologies). Metabolite concentrations were calculated using internal standards and reported in µM/L. Analytical specifications of AbsoluteIDQ^®^ p180, including analytical performance characteristics, were provided by the manufacturer [94]. Table S6 lists the limits of detection (LODs) of all the lipid species determined in our study.

### 4.4. Determination of Toxic Elements 

For trace element analysis, plasma samples were subjected to acid mineralization in an ETHOS™ UP high-performance microwave digestion system (Milestone, Sorisole, Italy) equipped with the SK-15 easyTEMP high-pressure rotor (PTFE-TFM vessels). After thawing, the samples were homogenized by sonification (15 min) and vortexing (30 s). Then, 0.5 mL of plasma sample were mineralized with 3.5 mL of nitric acid 65% (Suprapur^®^ grade, Supelco^®^, Merck, Darmstadt, Germany) and 1.5 mL of deionized water (DI water, conductivity <0.08 µS/cm, obtained with an HLP10 system, Hydrolab, Poland). After mineralization, samples were diluted to a final volume of 7 mL with DI water. Solutions were stored at 4 °C until analysis by ICP-MS. Calibration standard solutions were prepared from individual single element standards (1.000 mg/L, TraceCERT^®^, Sigma-Aldrich Production GmbH, Buchs, Switzerland), and calibration curves were obtained in the range of 0.2–50 µg/L. All solutions were prepared in freshly decontaminated plastic tubes (nitric acid and DI 1:1 at least three times). The concentration of Al, Ni, As, Cd, and Pb was determined with an XSeries 2 ICP-MS instrument (Thermo Fisher Scientific, Bremen, Germany) equipped with a collision/reaction cell operated using 7% H_2_/He gas mixture (Linde Gaz Polska, Kraków, Poland), an ASX-510 autosampler (CETAC, Omaha, NE, USA) and the PlasmaLab software version 2.6.1.335 (Thermo Fisher Scientific, Waltham, MA, USA). Each sample was analyzed in triplicate. The confirmation of linearity was performed based on the lack-of-fit test and regression model test using ANOVA. The limit of detection (LOD) of the method was calculated based on a 3 × standard deviation of 100 analytical blanks. Analytical accuracy was checked using certified reference material (CRM) with the serum matrix (Seronorm Trace Elements serum L2, Sero, Billingstad, Norway) with certified concentrations of analyzed elements. Additional non-matrix matched CRMs—EP-H-2 (EnviroMAT Drinking Water), and EU-H-3 (EnviroMAT Waste Water) (SCP Science, Quebec, ON, Canada)—were applied for validation of the analytical procedure. The concentrations found were in good agreement with the certified values. Recoveries were in the range of 95–110%. Table S7 lists the limits of detection (LODs) of all the toxic elements determined in our study.

### 4.5. Data Analysis and Statistical Methods

For the imputation of values below the LOQ, the following approach was adopted: values below the LOD were set to 0, acknowledging that such values fall below the detection capability of the measurement method. For values that fell between the LOD and LLOQ, the mean of the values within this range (greater than LOD but less than LLOQ) was computed and used for imputation. This mean value replaces missing values or those within the specified range, ensuring that the imputed data reflect a conservative estimate of concentration, grounded in the observed data distribution. This methodological choice aims to maintain the integrity of the dataset while addressing the challenges posed by values that are not quantifiable within the established analytical range.

#### 4.5.1. Principal Component Analysis

The prcomp function in the R stats package v. 3.6.2 (with centered data and “scale”. set as TRUE, so that data are standardized) was used to conduct PCA. Visualization of PCA results was performed using the factoextra R package. Loading scores, correlating the analyzed variables with principal components, were calculated and their absolute values were assessed to gauge each variable’s contribution. Variables exceeding a predefined threshold (the cut-off value for the absolute loading scores was 0.15 for glycerophospholipids and 0.25 for the other groups of lipids and analyzed trace elements) for the first two components (PC1 and PC2) were selected as having significant contribution. The goal was to focus on the features that most explain the variance captured by these components, as they typically contain the bulk of the information in the dataset. This approach improves the interpretability and efficiency of PCA by analyzing a reduced set of pertinent variables. The approach to determining the number of clusters, defined as the groups of data points that are close to each other in the reduced-dimensional space, in the dataset combined scree plot and K-means clustering analysis. Scree plots visually determined the variance explained for different numbers of clusters, pinpointing the “elbow” where more clusters do not noticeably improve variance explanation. This indicated that the two clusters were optimally balanced, minimizing within-cluster variance across all groups analyzed and maximizing between-cluster distinction.

The relationship between the analytical parameters evaluated (lipid compounds and toxic trace elements) and the patients’ clinicopathological data was investigated. Continuous variables were analyzed using the Mann–Whitney–Wilcoxon test. Log fold change (logFC) was calculated as log((mean2+1)/(mean1+1)), with a pseudo count of 1 added to prevent division by zero. Power analysis for the Wilcoxon test was conducted using the wmwpow package, based on Shieh’s method [95]. The power for a two-sided test with a 0.05 significance level was estimated via the shiehpow() function. Effect sizes were calculated using the wilcox_effsize() function from the rstatix package. Categorical variables were evaluated using the asymptotic chi-square or Fisher’s exact tests, depending on the count of observed subgroups, as described in the statistical package documentation. Enrichment, in this context, refers to the overrepresentation of certain subgroups based on demographic, clinical, and biochemical data within PCA-derived clusters. The Benjamini–Hochberg (BH) *p*-value adjustment method was employed to manage the false discovery rate for multiple comparisons. The results were considered statistically significant for adjusted *p*-values (padj) < 0.05. Data visualization and statistical analyses were performed using the ggpubr and ggplot2 R packages, version 4.1.3.

#### 4.5.2. Monte Carlo Feature Selection and Interdependency Discovery

Data simplification with simultaneous preservation of informative features was achieved by applying feature selection through the MCFS-ID method, using the rmcfs package [96]. Default parameters with 100 permutations were set to run MCFS-ID. The algorithm identifies features that collaborate to classify objects into subclasses, exploring multidimensional dependencies between classes and features. It creates a classifier to accurately categorize objects, each described by a feature vector. The MCFS-ID algorithm ranks features based on their relevance for classification, revealing both linear and nonlinear interdependencies regardless of the classifier used. This is achieved by building decision trees from randomly selected subsets of features and objects. The relative importance of features for classification is evaluated through decision tree analysis. With enough iterations for the algorithm to converge, the most effective classification features rise to the top of the ranking, allowing the algorithm to uncover collaborative feature effects on class assignments through multidimensional analysis.

#### 4.5.3. Pathway Analysis on the Significantly Affected Lipids

Pathway Activity Network of Lipid Classes and The Lipid Reaction Network analyses were conducted using the LipidSig 2.0 platform [97]. Initially, the Differential Expression (DE) module was employed on percentage normalized and log10 transformed data. The genes identified from the initial analysis were imputed into KEGG Mapper [98] to annotate metabolic pathways, signaling pathways, and biological processes.

## 5. Conclusions

Our study makes a significant contribution to deciphering the plasma lipidome precisely in people with hypothyroidism, as these are the patients who will potentially develop different manifestations of lipid disorders. A significant association between altered levels of several components of the plasma lipidome and several toxic trace elements with hypothyroidism was found. A set of 23 chemical substances have been identified that are fundamental in differentiating between healthy individuals and patients suffering from hypothyroidism. These include Al, Cd, Ni, As, and Pb, along with a large set of lipid compounds. We also identified a set of 17 different compounds and elements that were significantly different in patients depending on the type of disease: autoimmune or non-autoimmune hypothyroidism.

For several reasons, in-depth knowledge of the lipid profile of patients with hypothyroidism can be important. First, it is important for developing personalized treatment strategies that can effectively address each patient’s unique needs, thereby improving overall management and outcomes in treating patients. Hypothyroidism has a significant impact on metabolic rate, which can lead to profound changes in energy expenditure and nutrient utilization. Slow metabolism in HT patients can result in rapid weight gain. Understanding the specific metabolic changes associated with hypothyroidism can help tailor dietary treatments more effectively. Second, knowledge of the lipidome profile can provide information about risk factors and potential complications associated with HT. In particular, it is well known that altered lipid metabolism can increase the risk of cardiovascular diseases, which are a common comorbidity in HT patients. Profiling metabolic changes makes it possible to pre-emptively address these risks with appropriate interventions. Finally, metabolic profiling can also be a tool to monitor treatment effectiveness. Changes in specific metabolites can help assess the organism’s response to thyroid hormone replacement therapy or other treatments, allowing for timely adjustments in management strategies. 

This work also highlights the great scientific potential of modern analytical techniques such as FIA-ESI-MS/MS and ICP-MS, capable of quickly generating a huge amount of data about a biological sample, as well as the application of novel statistical approaches in the analysis of bioanalytical and clinical data. In fact, when dealing with large datasets, it is crucial to choose the appropriate statistical analysis tools to extract the most information from them. The approach we used, based on MCFS-ID, has proven to be a powerful tool for dealing with biochemical data, increasing our understanding of the mechanisms underlying diseases. Our findings should be extended and complemented with other omics data such as proteomics and genomics, which could provide a much greater understanding of thyroid dysfunction and associated lipid imbalances.

## Figures and Tables

**Table 1:** Summary table of demographic, clinical, and biochemical data of all study subjects (patients and controls) *.

No	Variable	Stats/Values	Freqs (% of Valid)	Graph	Valid	Missing
1	Gender [character]	1. F	104 (86.7%)	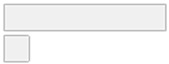	120 (100.0%)	0 (0.0%)
		2. M	16 (13.3%)			
2	Age [numeric]	Mean (sd): 29.6 (9.1)	30 distinct values	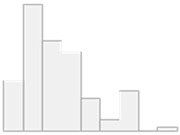	120 (100.0%)	0 (0.0%)
		min ≤ med ≤ max:				
		18 ≤ 28 ≤ 59				
		IQR (CV): 11.2 (0.3)				
3	BMI [numeric]	Mean (sd): 23.7 (3.3)	103 distinct values	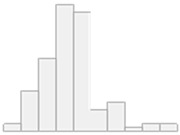	120 (100.0%)	0 (0.0%)
		min ≤ med ≤ max:				
		17.7 ≤ 23.7 ≤ 35.3				
		IQR (CV): 3.7 (0.1)				
4	LDL [numeric]	Mean (sd): 98.7 (30.4)	93 distinct values	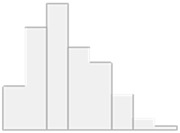	120 (100.0%)	0 (0.0%)
		min ≤ med ≤ max:				
		47 ≤ 94.2 ≤ 186.2				
		IQR (CV): 42.3 (0.3)				
5	HDL [numeric]	Mean (sd): 57.2 (12.9)	73 distinct values	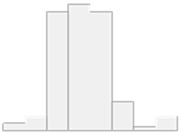	120 (100.0%)	0 (0.0%)
		min ≤ med ≤ max:				
		23.3 ≤ 56 ≤ 98				
		IQR (CV): 16.5 (0.2)				
6	TC [numeric]	Mean (sd): 177.7 (38.9)	84 distinct values	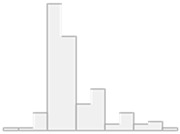	120 (100.0%)	0 (0.0%)
		min ≤ med ≤ max:				
		98 ≤ 165.2 ≤ 301.2				
		IQR (CV): 44 (0.2)				
7	TG [numeric]	Mean (sd): 90.1 (30.4)	88 distinct values	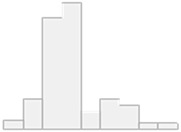	120 (100.0%)	0 (0.0%)
		min ≤ med ≤ max:				
		36 ≤ 83.7 ≤ 182				
		IQR (CV): 27.2 (0.3)				
8	TSH [numeric]	Mean (sd): 2.2 (0.9)	94 distinct values	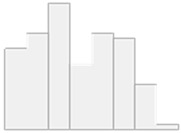	120 (100.0%)	0 (0.0%)
		min ≤ med ≤ max:				
		0.7 ≤ 2 ≤ 4.3				
		IQR (CV): 1.6 (0.4)				
9	fT4 [numeric]	Mean (sd): 1.5 (1.2)	62 distinct values	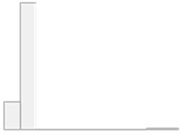	120 (100.0%)	0 (0.0%)
		min ≤ med ≤ max:				
		0.8 ≤ 1.5 ≤ 10.2				
		IQR (CV): 0.5 (0.8)				
10	fT3 [numeric]	Mean (sd): 2.9 (1)	90 distinct values	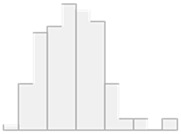	120 (100.0%)	0 (0.0%)
		min ≤ med ≤ max:				
		0.8 ≤ 2.9 ≤ 6.1				
		IQR (CV): 1.4 (0.3)				
11	Cohort [character]	1. Controls	61 (50.8%)	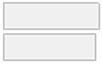	120 (100.0%)	0 (0.0%)
		2. HT patients	59 (49.2%)			
12	Disease [character]	1. Autoimmune_HT	32 (26.7%)	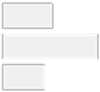	120 (100.0%)	0 (0.0%)
		2. Controls	61 (50.8%)			
		3. Non_autoimmune_HT	27 (22.5%)			
13	TSH_level [character]	1. high	1 (0.8%)	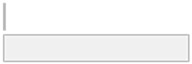	120 (100.0%)	0 (0.0%)
		2. normal	119 (99.2%)			
14	fT3_level [character]	1. high	14 (11.7%)	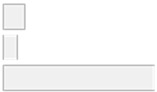	120 (100.0%)	0 (0.0%)
		2. low	9 (7.5%)			
		3. normal	97 (80.8%)			
15	fT4_level [character]	1. high	11 (9.2%)	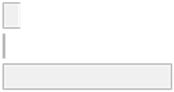	120 (100.0%)	0 (0.0%)
		2. low	1 (0.8%)			
		3. normal	108 (90.0%)			
16	TC_level [character]	1. normal	78 (65.0%)	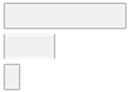	120 (100.0%)	0 (0.0%)
		2. primary_prevention	32 (26.7%)			
		3. secondary_prevention	10 (8.3%)			
17	LDL_level [character]	1. normal	69 (57.5%)	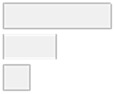	120 (100.0%)	0 (0.0%)
		2. primary_prevention	34 (28.3%)			
		3. secondary_prevention	17 (14.2%)			
18	HDL_level [character]	1. low	19 (15.8%)	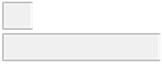	120 (100.0%)	0 (0.0%)
		2. normal	101 (84.2%)			
19	TG_level [character]	1. high	11 (9.2%)	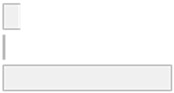	120 (100.0%)	0 (0.0%)
		2. low	1 (0.8%)			
		3. normal	108 (90.0%)			
20	Weight_status [factor]	1. Underweight	5 (4.2%)	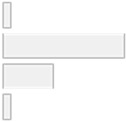	120 (100.0%)	0 (0.0%)
		2. Healthy_weight	78 (65.0%)			
		3. Overweight	32 (26.7%)			
		4. Obesity	5 (4.2%)			
21	Weight_groups [factor]	1. Underweight_or_Healthy	83 (69.2%)	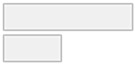	120 (100.0%)	0 (0.0%)
		2. Overweight_or_Obesity	37 (30.8%)			

* Abbreviations: BMI—body mass index; LDL—low-density lipoprotein; HDL—high-density lipoprotein; TC—total cholesterol; TG—triglycerides; TSH—thyroid-stimulating hormone; fT3—free triiodothyronine; fT4—free thyroxine.

**Table 2:** Adjusted *p* values for enrichment of clinical categories within PCA of all individuals clustering based on statistical association tests. Calculations used the asymptotic chi-square or Fisher’s exact tests with BH *p*-value correction on all the analyzed participants.

	Acylcarnitines	Glycerophospholipids	Sphingolipids	Toxic Trace Elements
Variable	padj	padj	padj	padj
Gender	0.478351867	0.565579025	0.565579025	0.090293429
Cohort (Controls vs. HT patients)	2.36664 × 10^−5^	8.6635 × 10^−8^	8.6635 × 10^−8^	3.02895 × 10^−6^
Disease (Controls vs. autoimmune HT vs. non-autoimmune HT)	3.7519 × 10^−5^	1.12254 × 10^−7^	1.12254 × 10^−7^	1.6082 × 10^−11^
fT3_level	0.223051666	0.769335436	0.769335436	1
fT4_level	0.665371092	0.916228719	0.916228719	0.818701672
TC_level	0.373032061	0.086842311	0.086842311	0.00382032
LDL_level	0.478351867	0.769335436	0.769335436	0.58095462
HDL_level	0.223051666	0.769335436	0.769335436	0.00382032
TG_level	0.11759458	0.250574397	0.250574397	0.114958437
Weight_status	0.478351867	0.769335436	0.769335436	0.220153881
Weight_groups	0.665371092	1	1	0.198591583

**Table 3:** Table of adjusted *p* values for enrichment of clinical subgroups within PCA participants clustering based on statistical association tests. The calculations were performed using asymptotic Chi-square or Fisher’s test with BH *p*-value correction on the HT patients’ cohort.

Variable	Acylcarnitines padj	Glycerophospholipids padj	Sphingolipids padj	Toxic Trace Elements padj
Gender	1	0.970383293	0.970383293	0.265454073
Disease (autoimmune HT vs. non-autoimmune HT)	0.851078962	0.970383293	0.970383293	1.41723 × 10^−5^
fT3_level	1	0.698006698	0.698006698	1
fT4_level	0.851078962	1	1	1
TC_level	0.851078962	0.955552885	0.955552885	1
LDL_level	0.851078962	0.970383293	0.970383293	0.688602232
HDL_level	1	1	1	0.037488777
TG_level	0.851078962	1	1	1
Weight_status	0.087940642	0.955552885	0.955552885	0.369851258
Weight_groups	0.159298729	0.955552885	0.955552885	1

**Table 4:** Summary of lipids and toxic elements distinguishing controls from HT. The table presents the top 20 lipids and toxic elements ranked by absolute log fold change (logFC) between controls and HT.

Chemicals	Comparisons		logFC	padj
PC.aa.C36.4	controls	HT	2.712505038	3.78063 × 10^−6^
PC.aa.C38.6	controls	HT	2.6773185	0.000205764
PC.aa.C34.2	controls	HT	2.673914809	2.06004 × 10^−6^
PC.aa.C38.5	controls	HT	2.330352069	1.94959 × 10^−5^
SM.C24.1	controls	HT	2.286497476	1.03295 × 10^−5^
SM.C18.0	controls	HT	2.254098795	1.37211 × 10^−7^
PC.aa.C40.6	controls	HT	2.235902323	2.02544 × 10^−6^
PC.aa.C34.1	controls	HT	2.162178595	1.26346 × 10^−6^
SM.C16.1	controls	HT	1.939967921	1.59859 × 10^−5^
SM.C16.0	controls	HT	1.929180292	1.67982 × 10^−5^
PC.aa.C32.1	controls	HT	1.893584951	2.32902 × 10^−6^
PC.aa.C34.3	controls	HT	1.621706394	4.01834 × 10^−6^
PC.aa.C36.5	controls	HT	1.561174879	1.47071 × 10^−6^
PC.aa.C36.3	controls	HT	1.466726329	3.27657 × 10^−5^
As	controls	HT	1.354226923	2.34859 × 10^−6^
PC.aa.C32.0	controls	HT	1.321623991	1.37967 × 10^−7^
PC.aa.C40.5	controls	HT	1.292471945	7.61078 × 10^−7^
PC.aa.C38.1	controls	HT	−1.265456573	2.0985 × 10^−6^
Cd	controls	HT	1.234674307	1.31768 × 10^−9^
PC.aa.C32.2	controls	HT	−1.199293026	4.01834 × 10^−6^

**Table 5:** Summary of lipids and toxic elements distinguishing autoimmune and non-autoimmune HT from controls. The table displays the top 20 lipids and toxic elements, ranked by absolute log fold change (logFC), comparing controls with autoimmune and non-autoimmune HT.

Chemicals	Comparisons		logFC	padj
PC.aa.C38.6	autoimmune_HT	controls	−2.801529125	0.000783075
PC.aa.C36.4	autoimmune_HT	controls	−2.76501264	0.000128351
PC.aa.C34.2	autoimmune_HT	controls	−2.71255371	9.24707 × 10^−5^
PC.aa.C36.4	controls	non_autoimmune_HT	2.647693225	0.001373544
PC.aa.C34.2	controls	non_autoimmune_HT	2.626738818	0.000905786
PC.aa.C38.6	controls	non_autoimmune_HT	2.514758915	0.035409203
PC.aa.C38.5	autoimmune_HT	controls	−2.421935645	0.0004787
SM.C18.0	autoimmune_HT	controls	−2.421344404	1.02889 × 10^−6^
SM.C24.1	autoimmune_HT	controls	−2.36087846	0.000132443
PC.aa.C40.6	autoimmune_HT	controls	−2.355839891	9.24707 × 10^−5^
PC.aa.C34.1	autoimmune_HT	controls	−2.269229324	6.00058 × 10^−5^
PC.aa.C38.5	controls	non_autoimmune_HT	2.213697601	0.003754317
SM.C24.1	controls	non_autoimmune_HT	2.193069239	0.004835913
SM.C16.1	autoimmune_HT	controls	−2.092035671	0.000117817
SM.C16.0	autoimmune_HT	controls	−2.086614683	0.00114639
PC.aa.C40.6	controls	non_autoimmune_HT	2.079498496	0.000866283
SM.C18.0	controls	non_autoimmune_HT	2.026957117	5.23654 × 10^−5^
PC.aa.C34.1	controls	non_autoimmune_HT	2.024073637	0.000680065
PC.aa.C32.1	autoimmune_HT	controls	−2.017971279	0.000113843
As	autoimmune_HT	controls	−1.80228527	7.65009 × 10^−8^

**Table 6:** Summary of top toxic elements distinguishing autoimmune from non-autoimmune. The chemicals were ranked by absolute log fold change (logFC) concentration in both analyzed sub-groups.

Chemicals	Comparisons		logFC	padj
As	autoimmune_HT	non_autoimmune_HT	−1.263201744	0.000510539
Cd	autoimmune_HT	non_autoimmune_HT	−1.073686488	0.000510539
Ni	autoimmune_HT	non_autoimmune_HT	−1.044931292	0.018508604
Al	autoimmune_HT	non_autoimmune_HT	−0.972513698	7.7266 × 10^−9^

## Data Availability

The original contributions presented in the study are included in the article/supplementary material, further inquiries can be directed to the corresponding author.

## Supplementary Materials

The following supporting information can be downloaded at https://www.mdpi.com/article/10.3390/molecules29215169/s1. The article has accompanying supplementary files: Figures S1–S16 and Tables S1–S7. Supplementary Figure S1. Identification of key acylcarnitines for patient classification using PCA; Supplementary Figure S2. Identification of key glycerophospholipids for patient classification using PCA; Supplementary Figure S3. Identification of key sphingolipids for patient classification using PCA; Supplementary Figure S4. Identification of key toxic trace elements for patient classification using PCA; Supplementary Figure S5. Detailed analysis of PCA clustering of acylcarnitines for all patients; Supplementary Figure S6. Detailed analysis of PCA clustering of glycerophospholipids for all patients; Supplementary Figure S7. Detailed analysis of PCA clustering of sphingolipids for all patients; Supplementary Figure S8. Detailed analysis of PCA clustering of lipids and toxic elements for all patients; Supplementary Figure S9. Identification of key acylcarnitines for HT patient stratification using PCA; Supplementary Figure S10. Identification of key glycerophospholipids for all HT patient stratification using PCA; Supplementary Figure S11. Identification of key sphingolipids for HT patient stratification using PCA; Supplementary Figure S12. Identification of key toxic elements for HT patient stratification using PCA; Supplementary Figure S13. Detailed analysis of PCA clustering of acylcarnitines for disease-only patients; Supplementary Figure S14. Detailed analysis of PCA clustering of glycerophospholipids for disease-only; Supplementary Figure S15. Detailed analysis of PCA clustering of sphingolipids for disease-only patients; Supplementary Figure S16. Detailed analysis of PCA clustering of toxic elements for disease-only patients. Description for S1–S4 and S9–S12 figures: (A) PCA plots showing cluster analysis results. The figure presents a PCA dot plot, showing the distribution of data points along the first two principal components, PC1 and PC2. Dots are color-coded according to two distinct clusters, providing visual differentiation of the data groups based on their PCA scores. (B) PCA features. The arrow length corresponds to the square of the cosine (cos^2^) values for each variable, with higher values suggesting a stronger association with the respective principal components. (C) and (D) feature bar plots of PC1 and PC2, respectively. Bar plots detailing the contribution of variables to PC1 and PC2, respectively. Viewing the dataset’s structure allows the identification of compounds that most significantly influence the variance explained by PC1 and PC2. (E) PCA scree plot. The plot displays the proportion of variance explained by each principal component (Dimension), effectively mapping the contribution of each dimension. Description for S5–S8 and S13–S16 figures: Each panel consists of a PCA clustering scatter plot (on the left) alongside a normalized bar plot of a contingency table for each analyzed grouping feature, as indicated in the legend. The PCA clustering displays the first two principal components (PC1 and PC2), with events colored according to the grouping feature. The bar plot of a contingency table shows the relationship of patients grouped by PCA clusters (labeled as “Cluster”) across the grouping feature. The *p*-value from Fisher’s test is also displayed, allowing for the assessment of statistical significance between the groups. Supplementary Tables: Table S1. Nomenclature of lipids; Table S2. Comparative analysis of chemical compound concentrations in all patients grouped by Cohort variable using Mann–Whitney–Wilcoxon Test with *p*-value adjustment and descriptive statistics; Table S3. Comparative analysis of chemical compound concentrations in all patients grouped by Disease variable using Mann–Whitney–Wilcoxon Test with *p*-value adjustment and descriptive statistics; Table S4. Comparative analysis of chemical compound concentrations in HT patients grouped by Disease variable using Mann–Whitney–Wilcoxon Test with *p*-value adjustment and descriptive statistics; Table S5. KEGG Annotated Metabolic Pathways, Signaling Pathways, and Biological Processes Differentiating Between Controls and HT; Table S6. Chemical Species Concentrations. The table presents the concentrations of the analyzed chemicals along with metadata annotations; Table S7. Limits of detection of toxic elements in plasma of the study population (N = 120); Table S8. Limits of detection of studied lipid species in plasma of the study population (N = 120). Supplementary Tables S2–S4 Description: the Mann–Whitney–Wilcoxon analysis of differences in lipids and trace elements concentration among analyzed subgroups. The *p*-values of the Mann–Whitney–Wilcoxon test (p_value) and padj (adj_p_value) are shown. The column ‘Continuous_Variable’ represents analyzed chemicals under analysis, while ‘Subgroup_Variable’ defines the subgroups being compared. ‘Group1’ and ‘Group2’ correspond to the compared subgroups, with their respective sample sizes (N1 and N2). Descriptive statistics such as median (Median_Group1, Median_Group2), mean (Mean_Group1, Mean_Group2), and standard deviation (SD_Group1, SD_Group2) are provided for both subgroups. The log fold change (logFC), Mann–Whitney statistic (Statistic), effect size (Effect_Size), and power of the test (Power) are also included. Each row corresponds to a single statistical comparison.
