# Supplementary figures and images for: Application of Human Plasma Targeted Lipidomics and Analysis of Toxic Elements to Capture the Metabolic Complexities of Hypothyroidism
Source: Molecules. 2024 Oct 31;29(21):5169. doi: 10.3390/molecules29215169 (PMC11547455; doi:10.3390/molecules29215169)

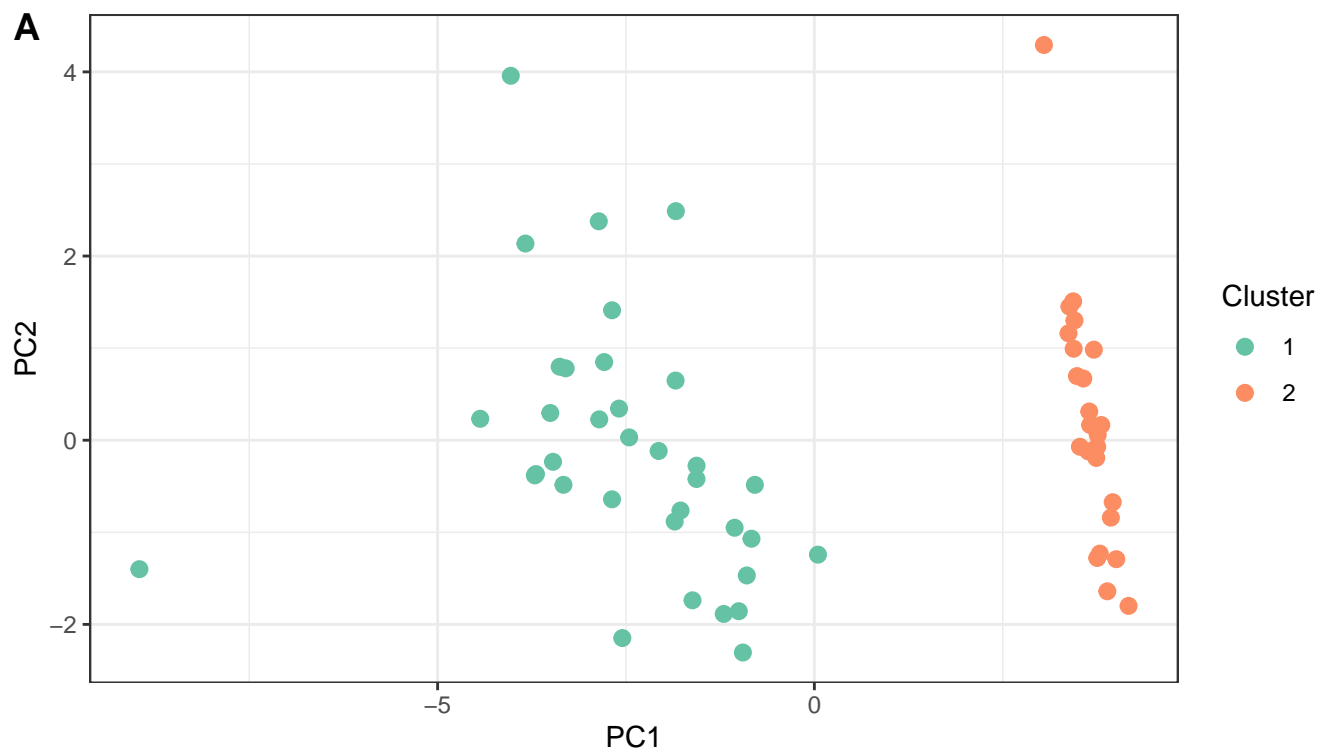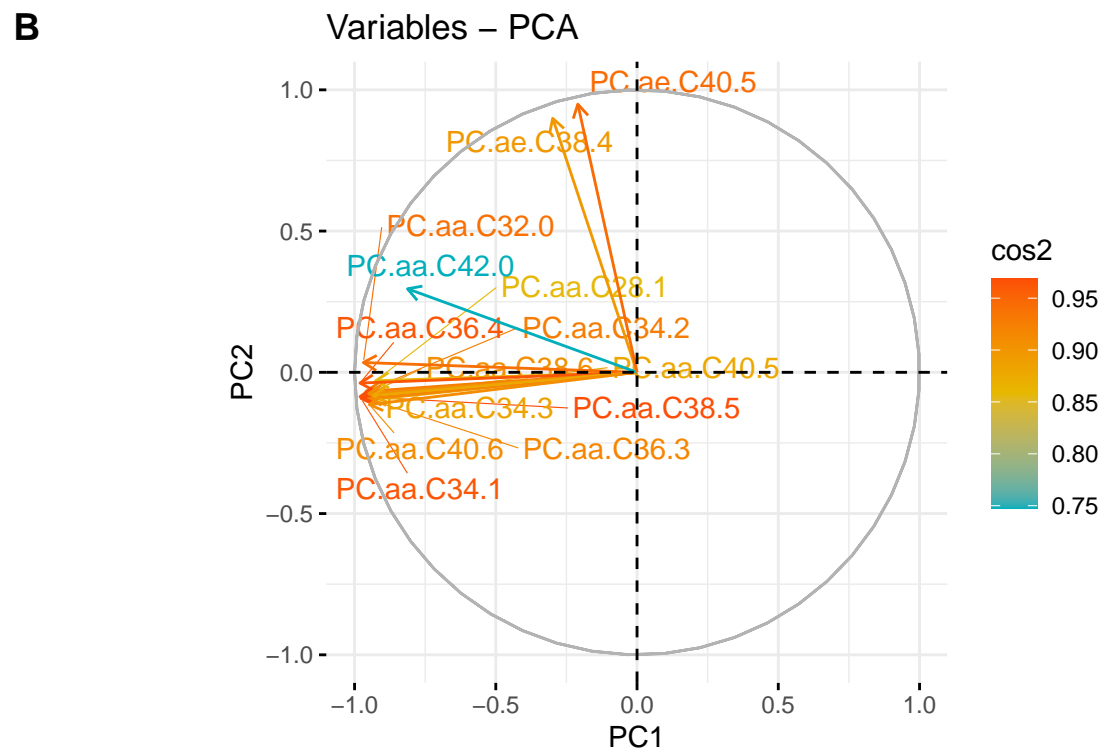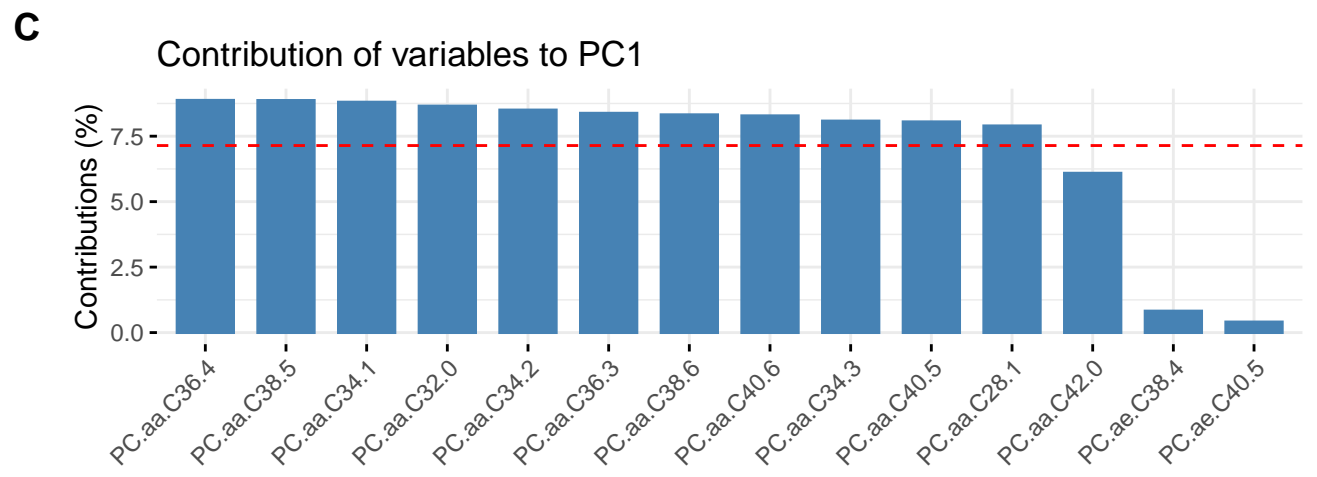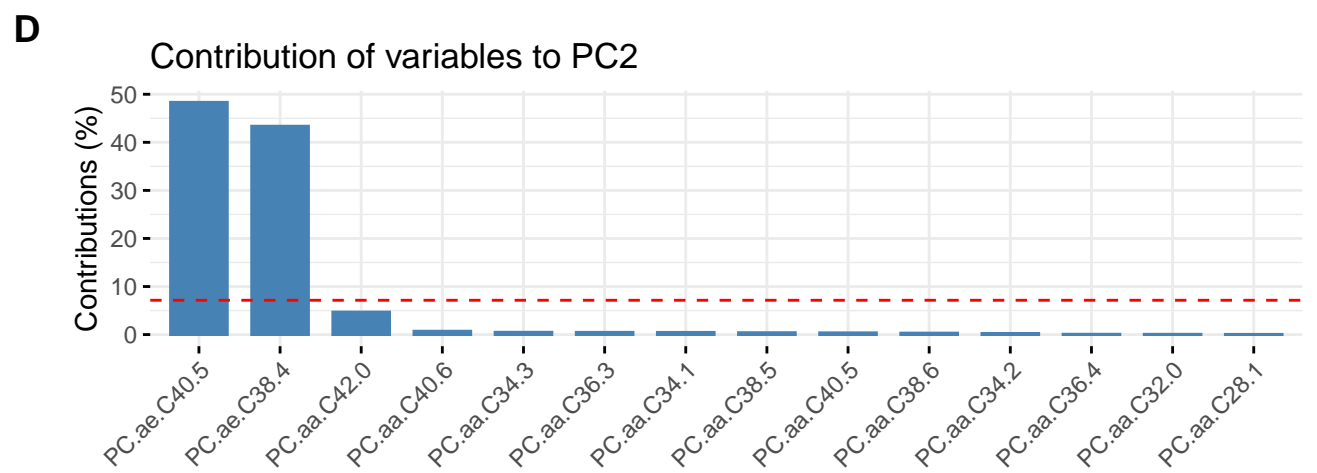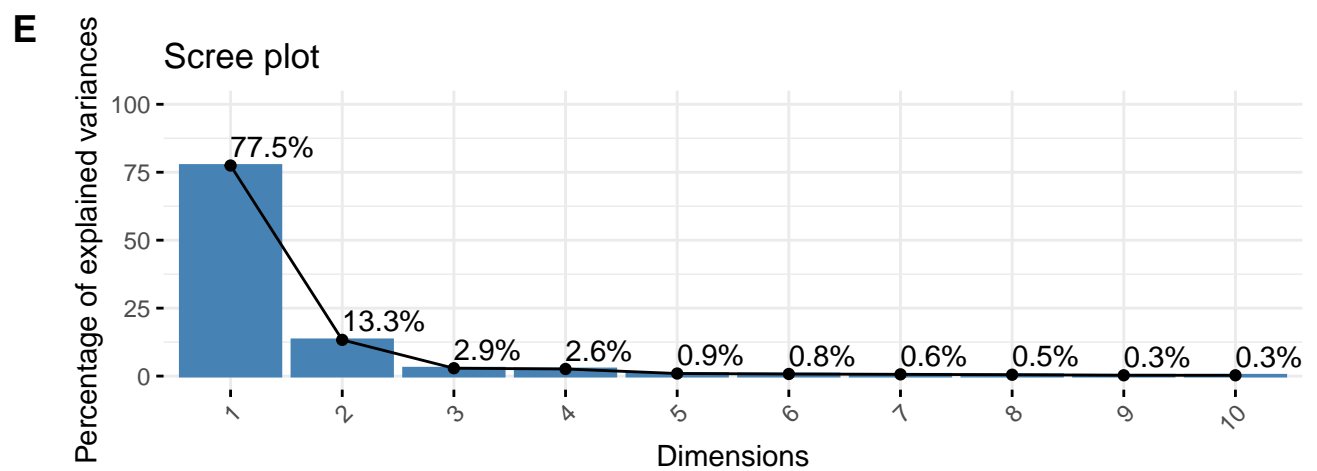

Supplement: Supplementary file 1 [file molecules-29-05169-s001.zip › molecules-3242400-supplementary/F10_filtered_biplot_eig_contrib.pdf]

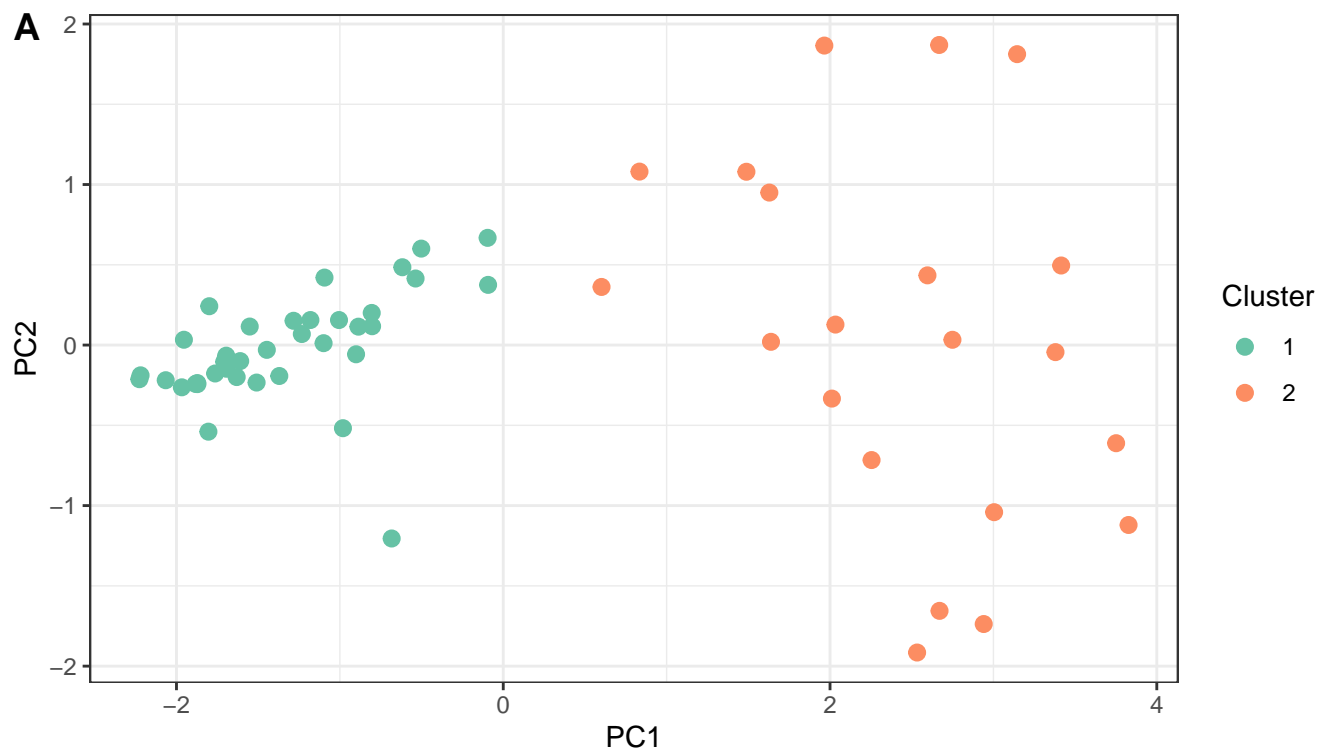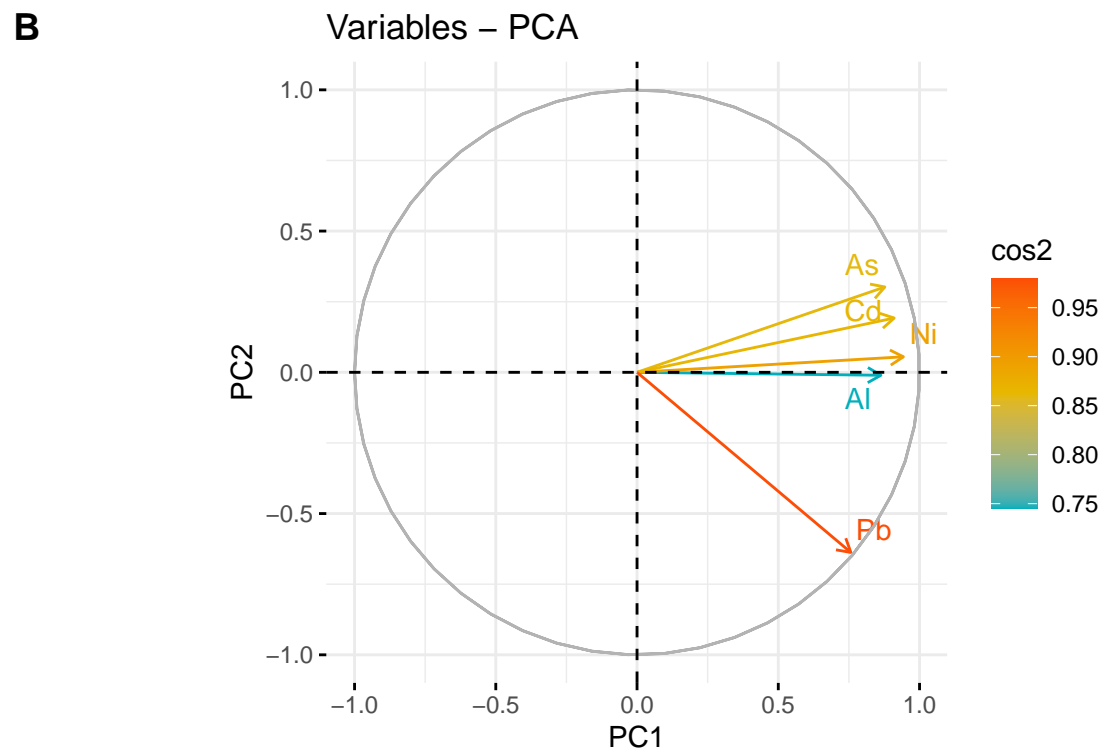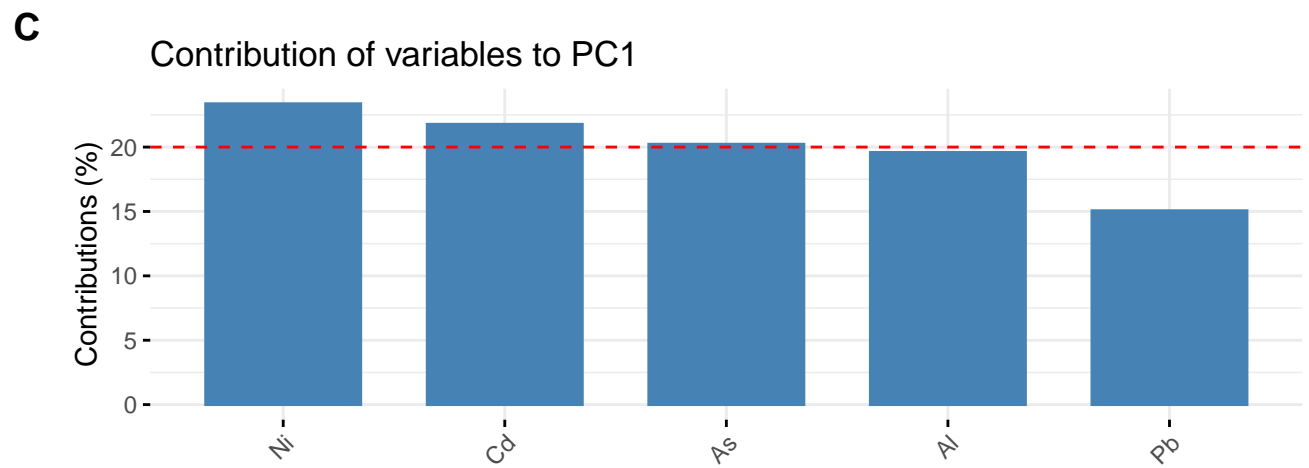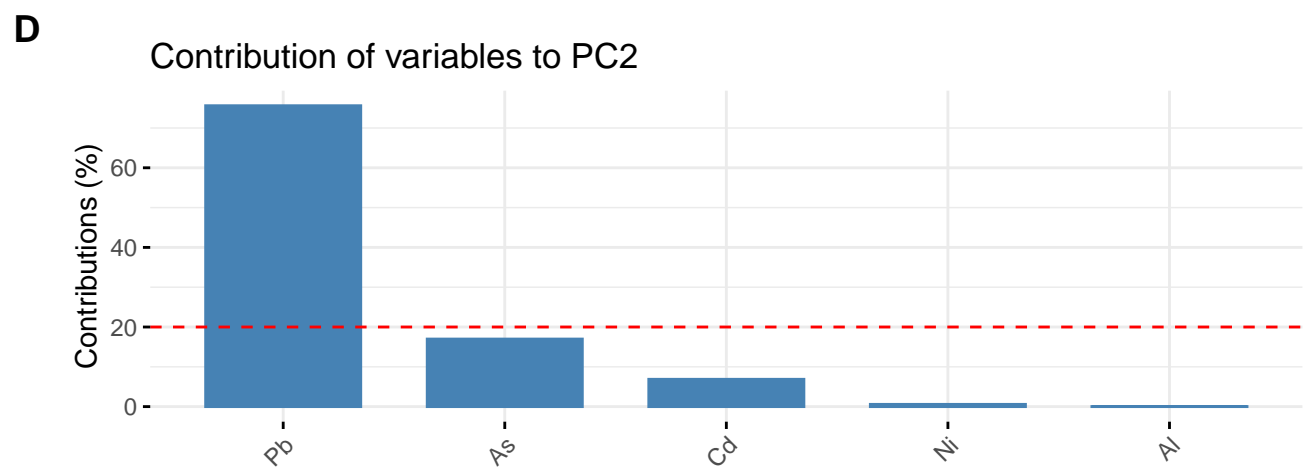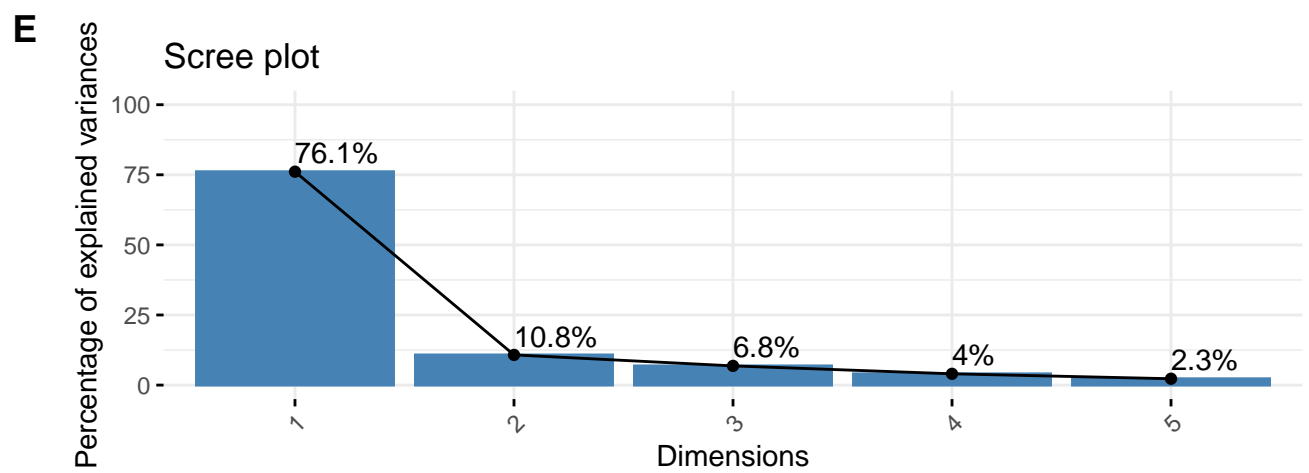

Supplement: Supplementary file 1 [file molecules-29-05169-s001.zip › molecules-3242400-supplementary/F12_filtered_biplot_eig_contrib.pdf]

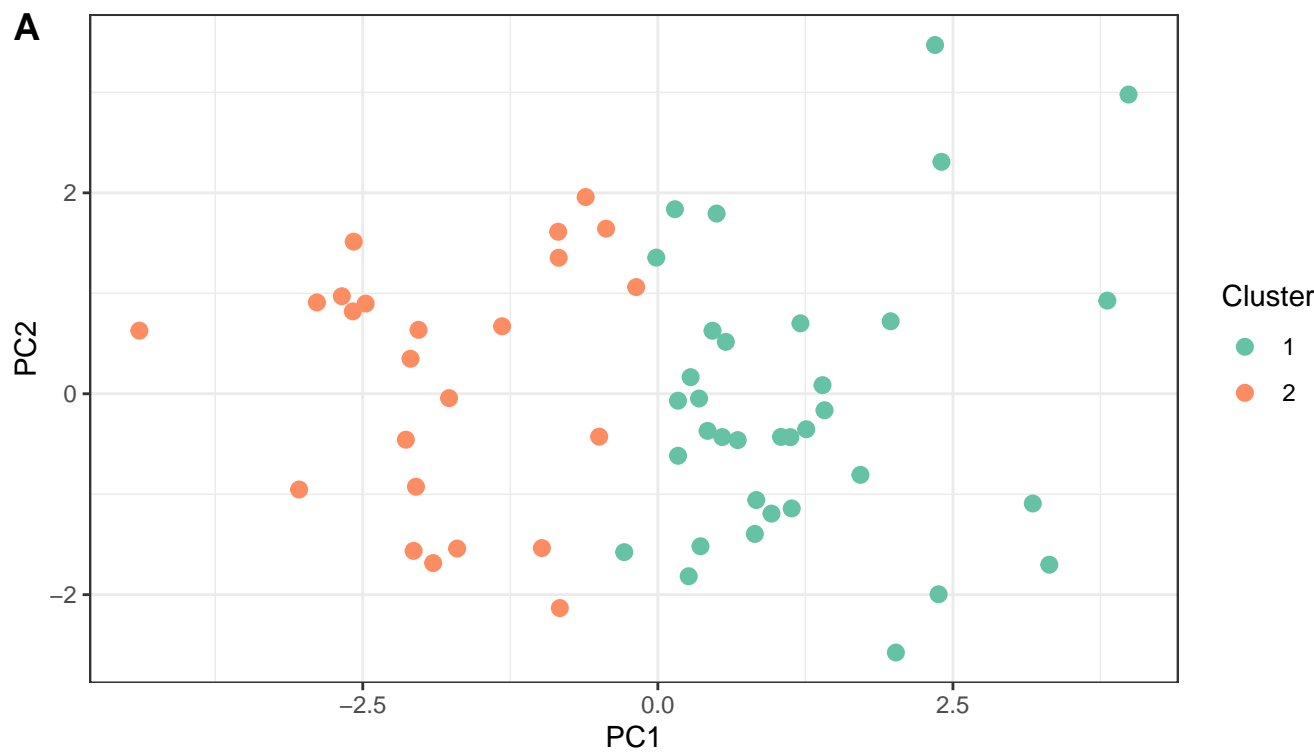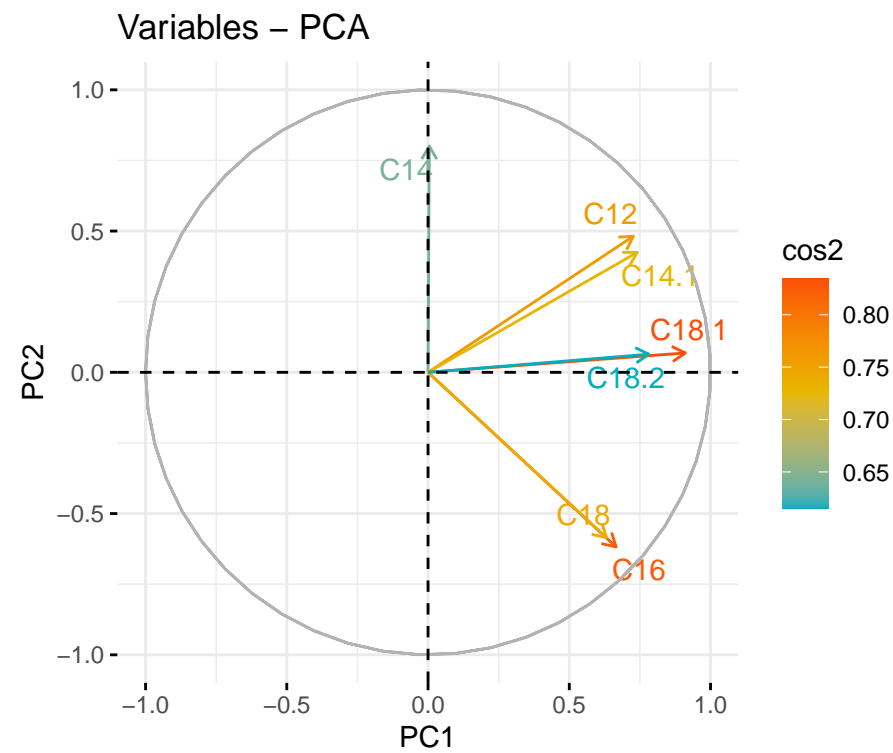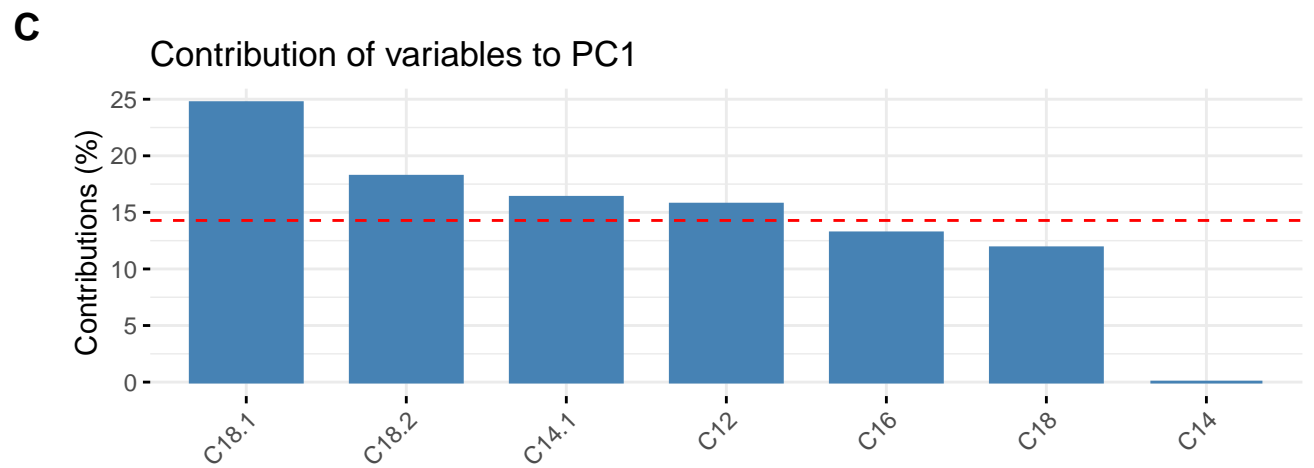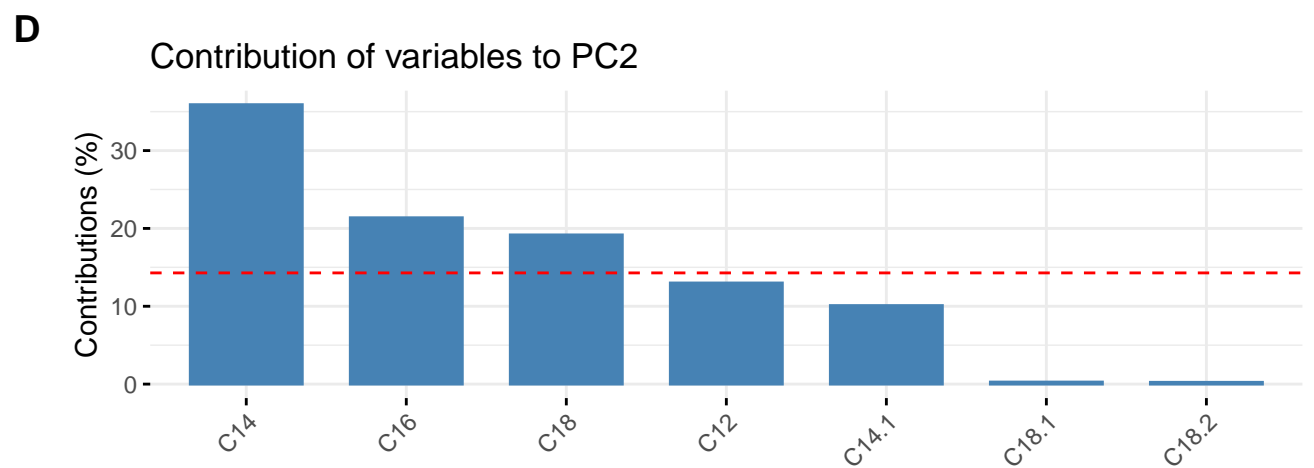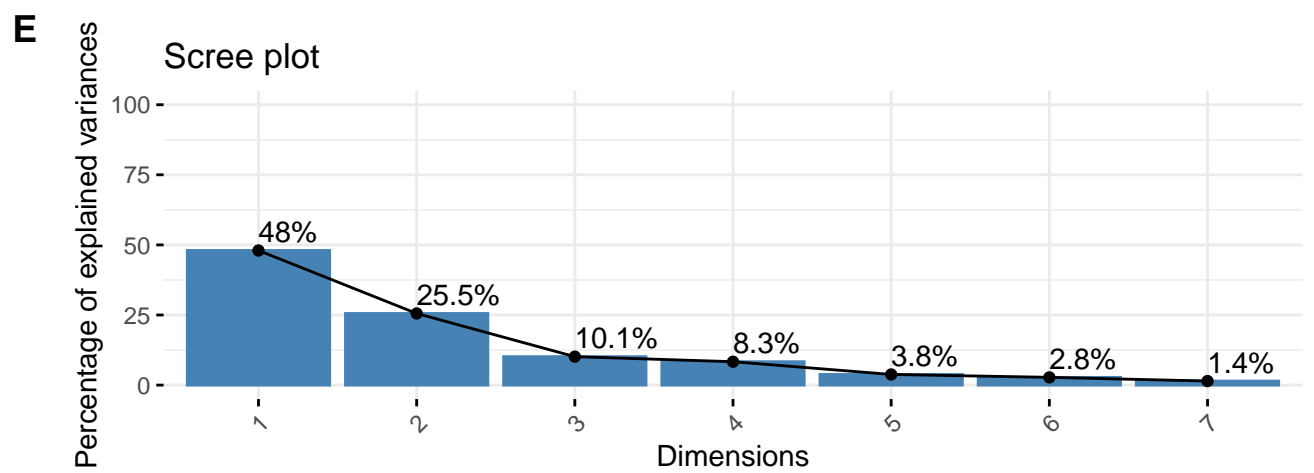

Supplement: Supplementary file 1 [file molecules-29-05169-s001.zip › molecules-3242400-supplementary/F9_filtered_biplot_eig_contrib.pdf]

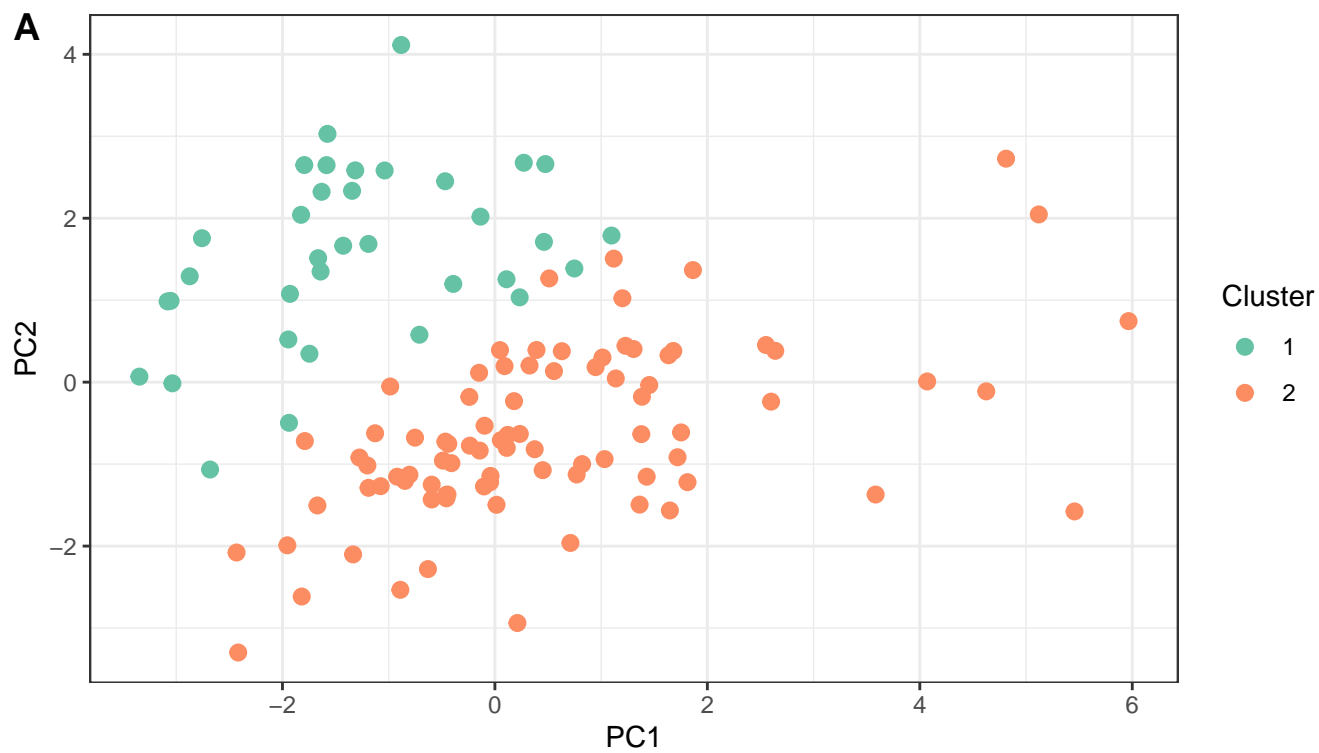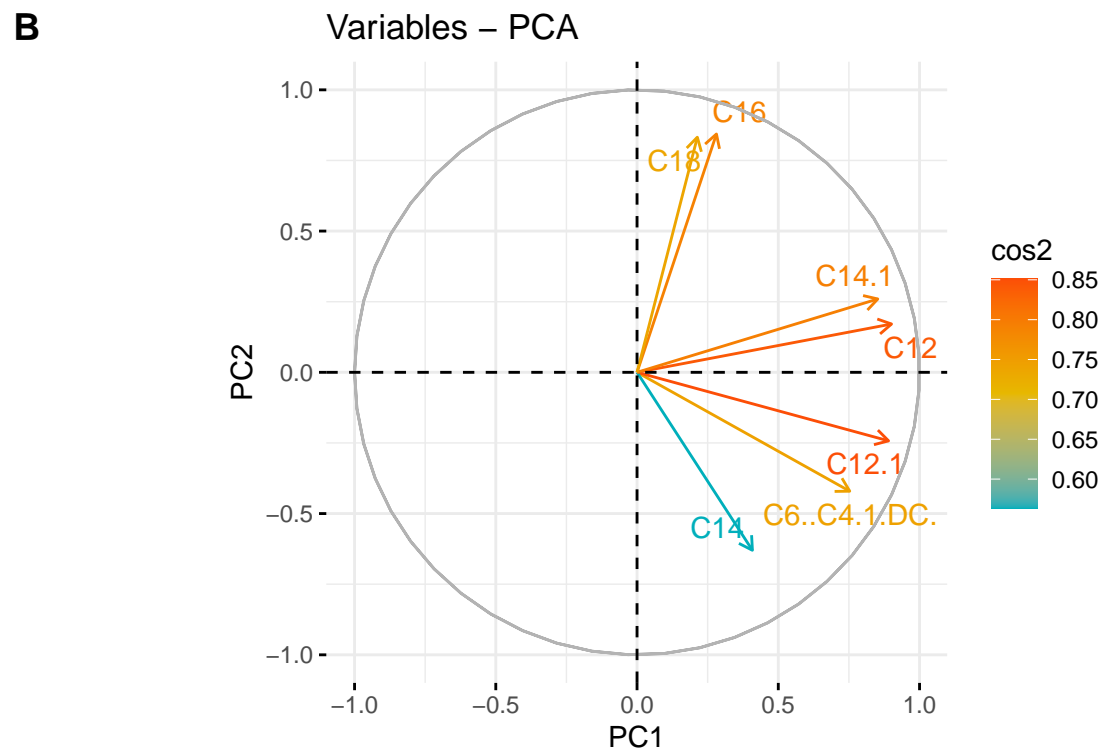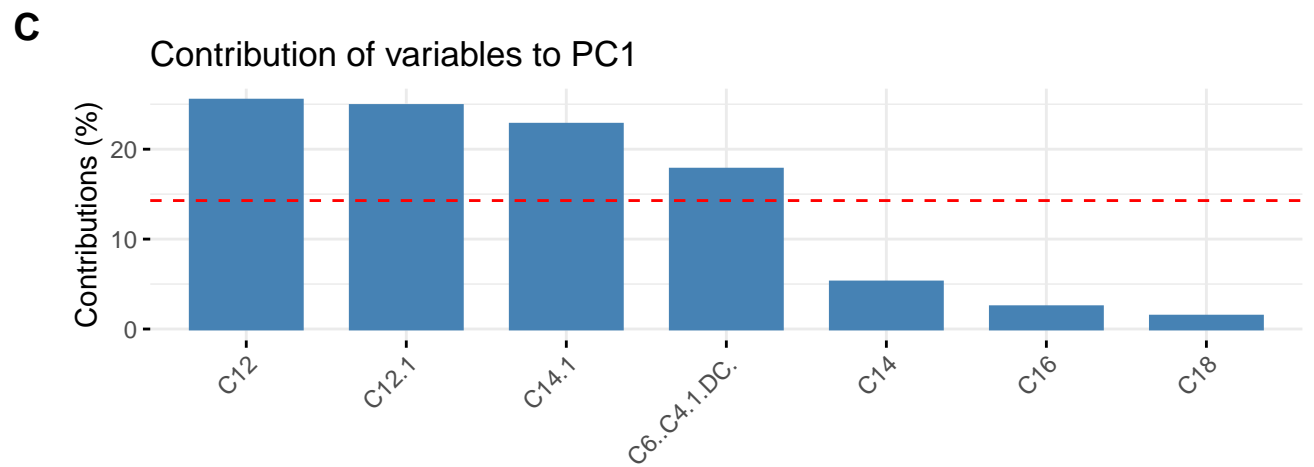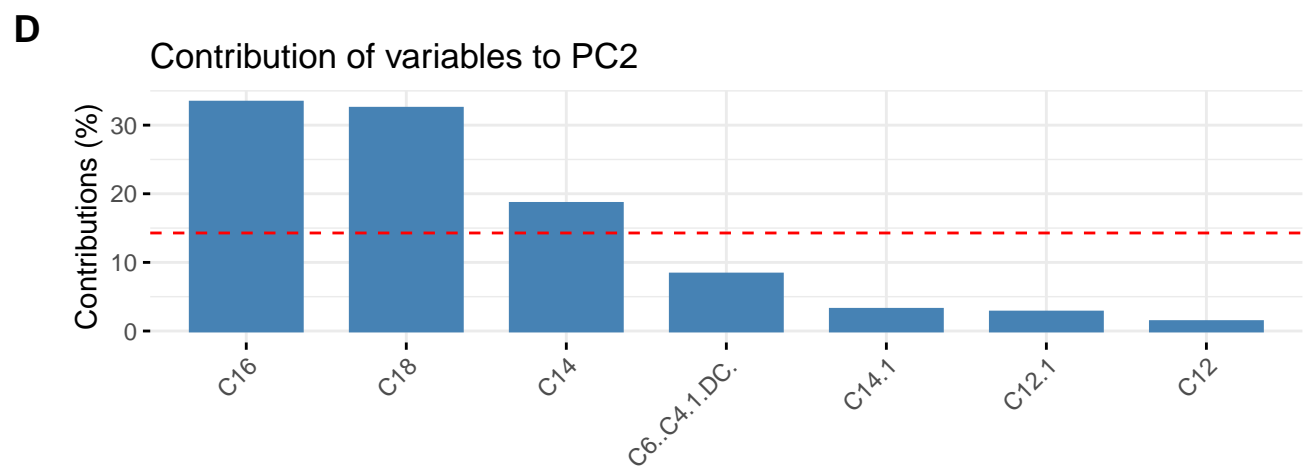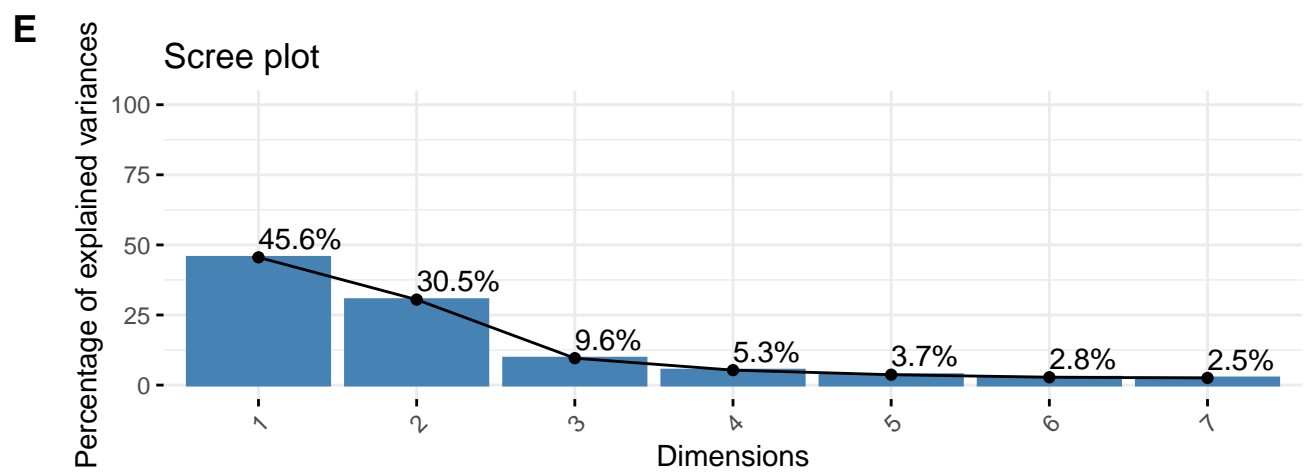

Supplement: Supplementary file 1 [file molecules-29-05169-s001.zip › molecules-3242400-supplementary/S1_filtered_biplot_eig_contrib.pdf]

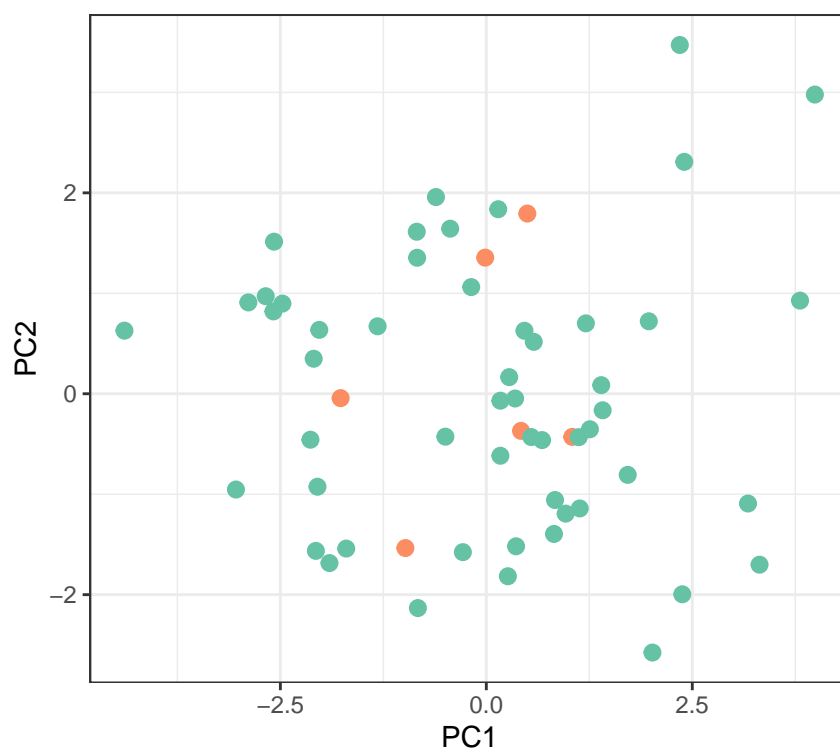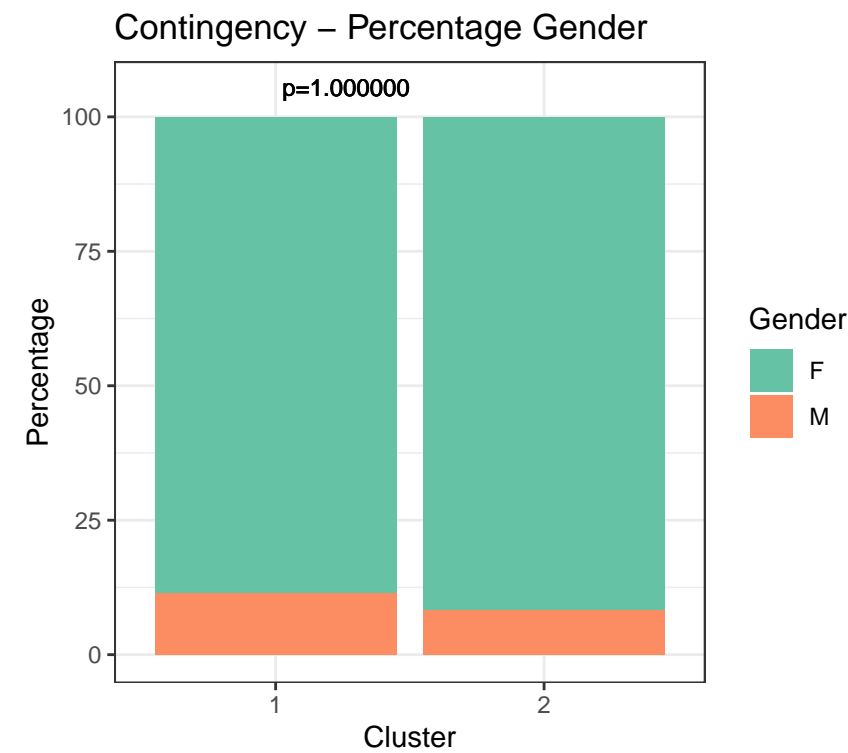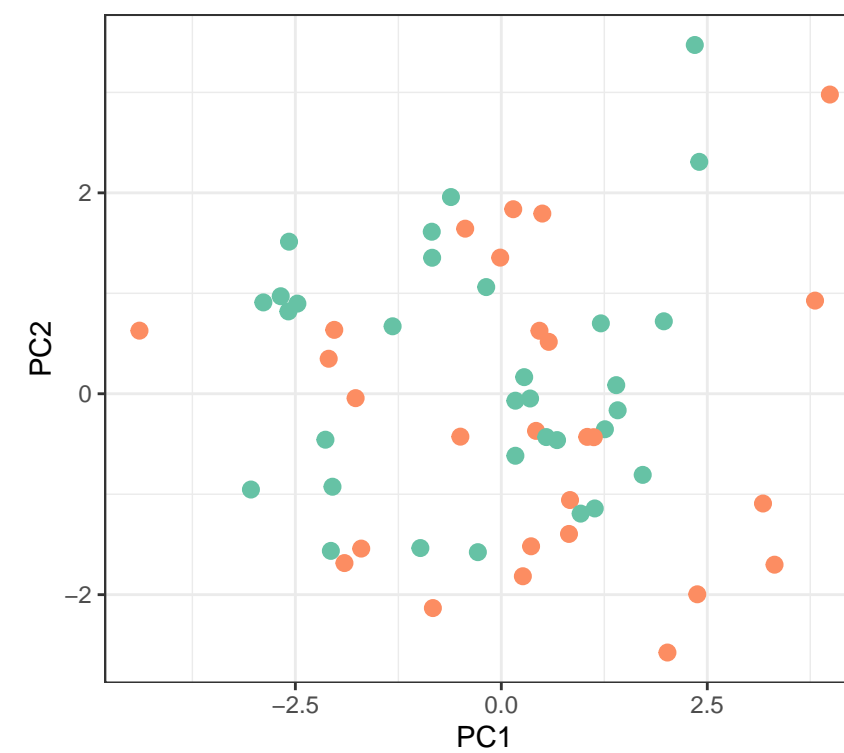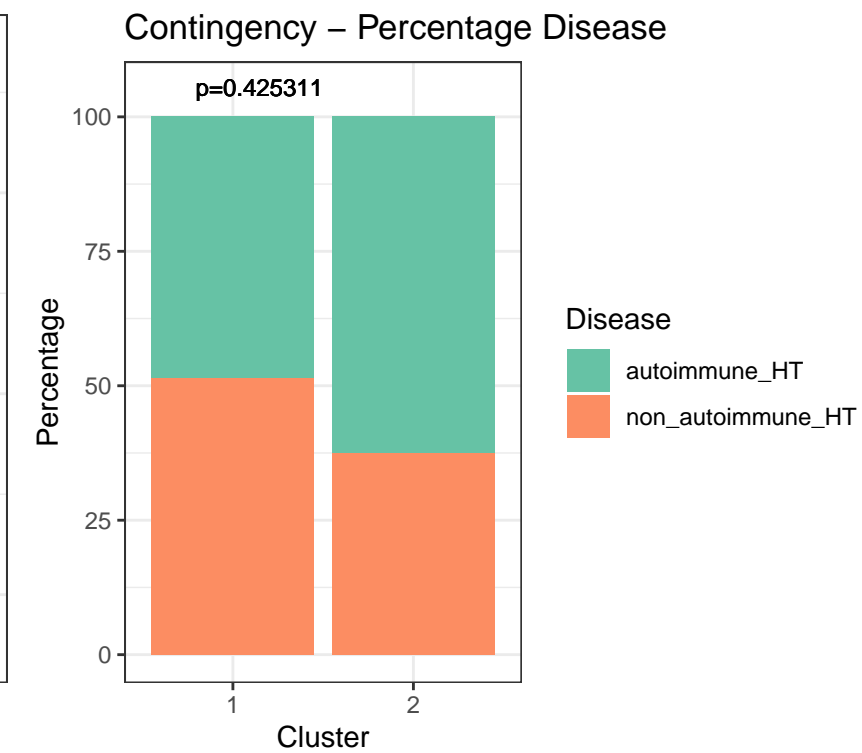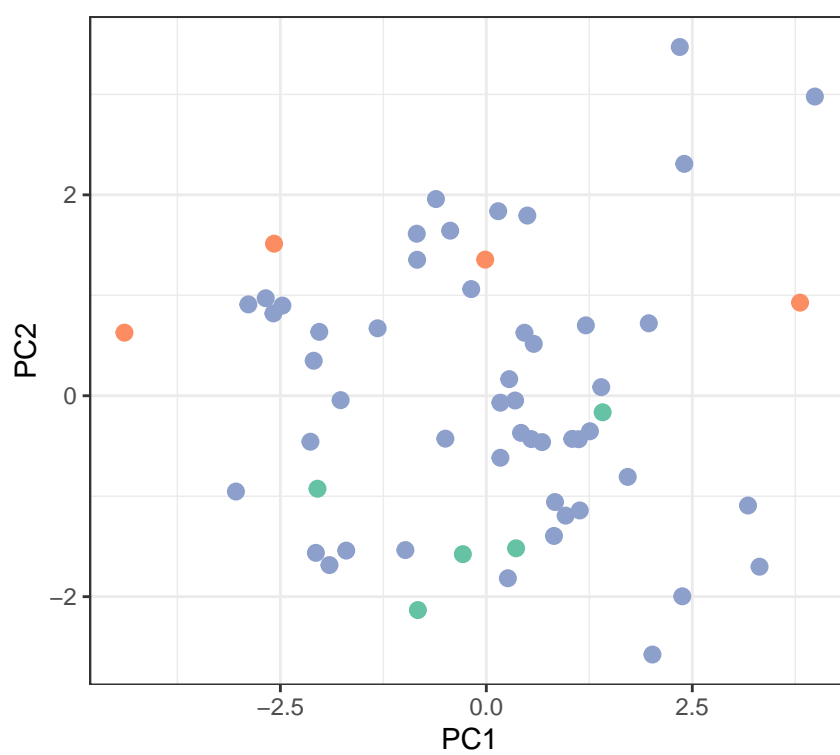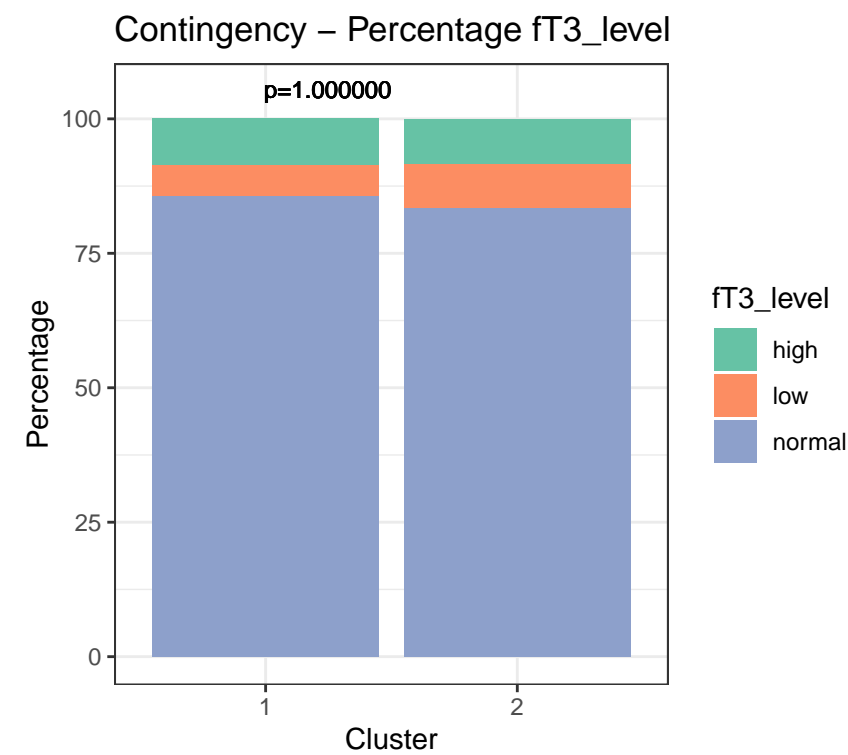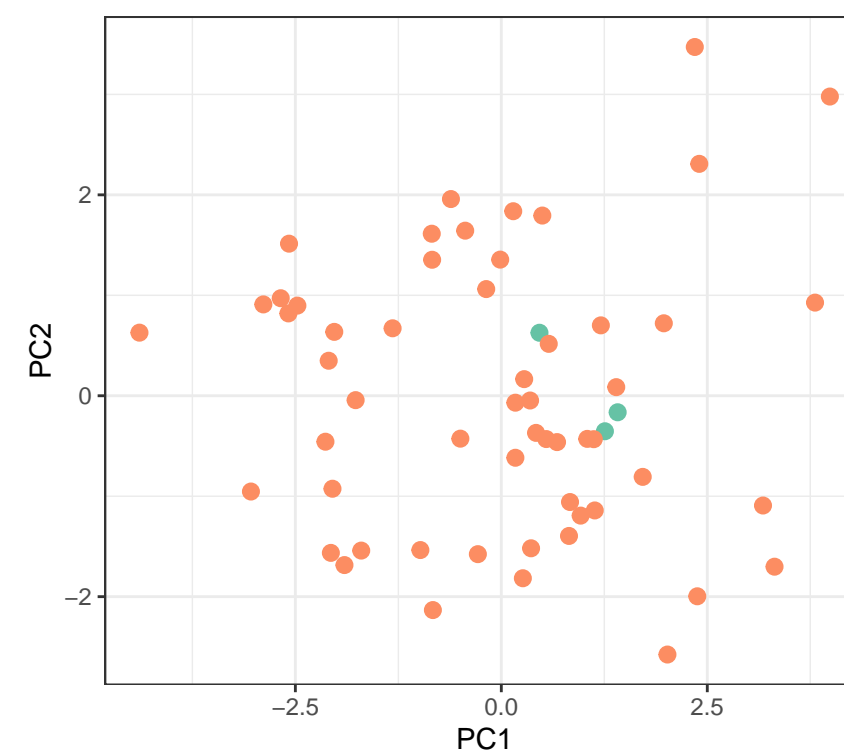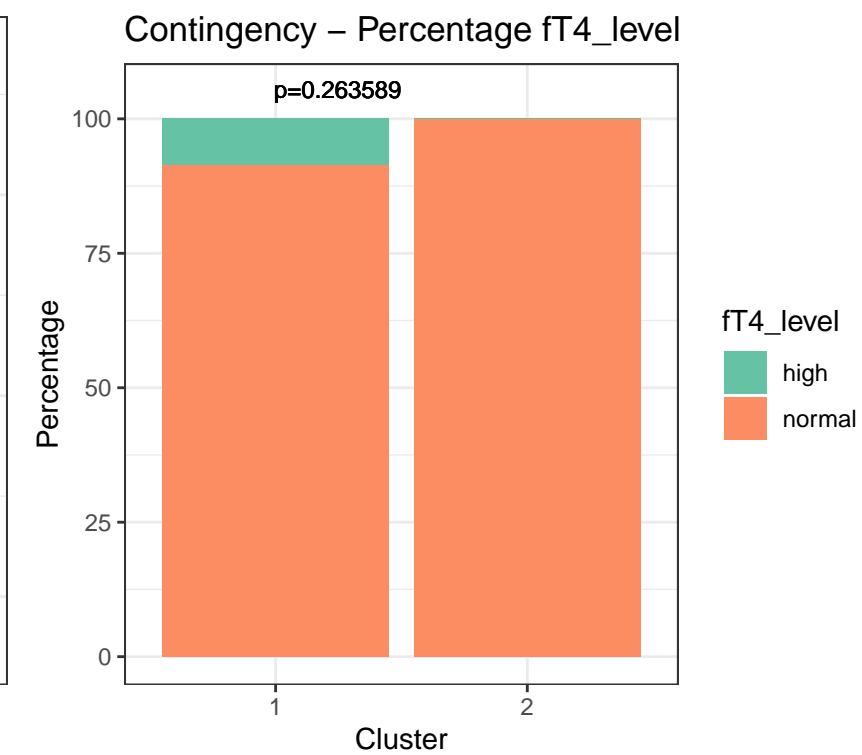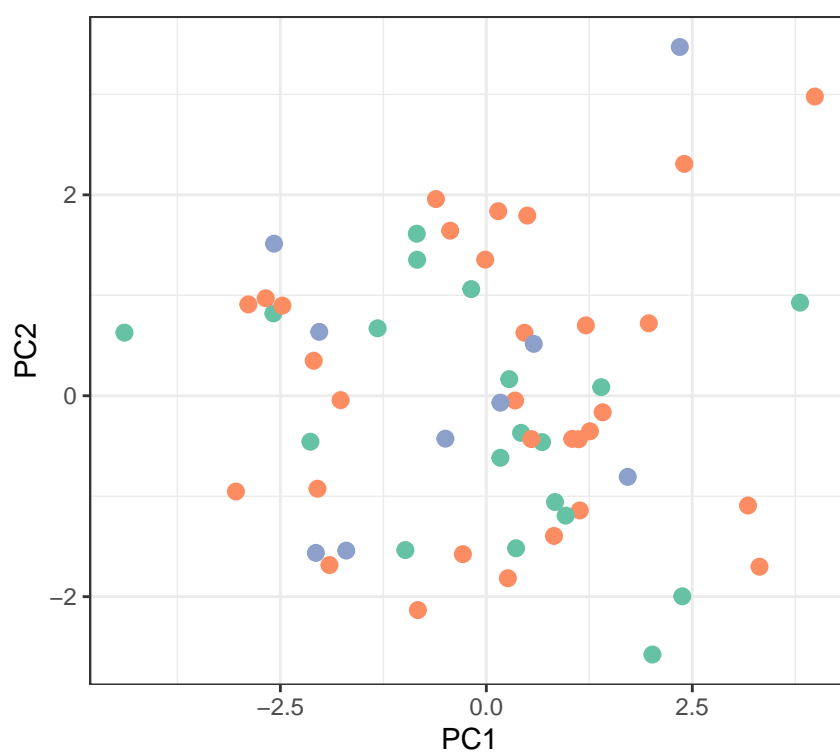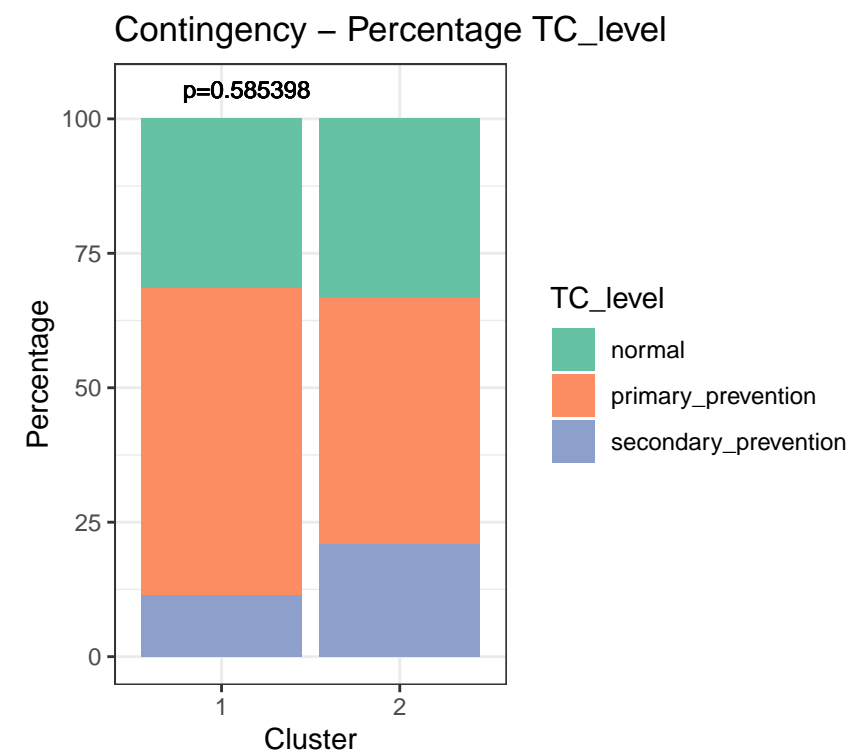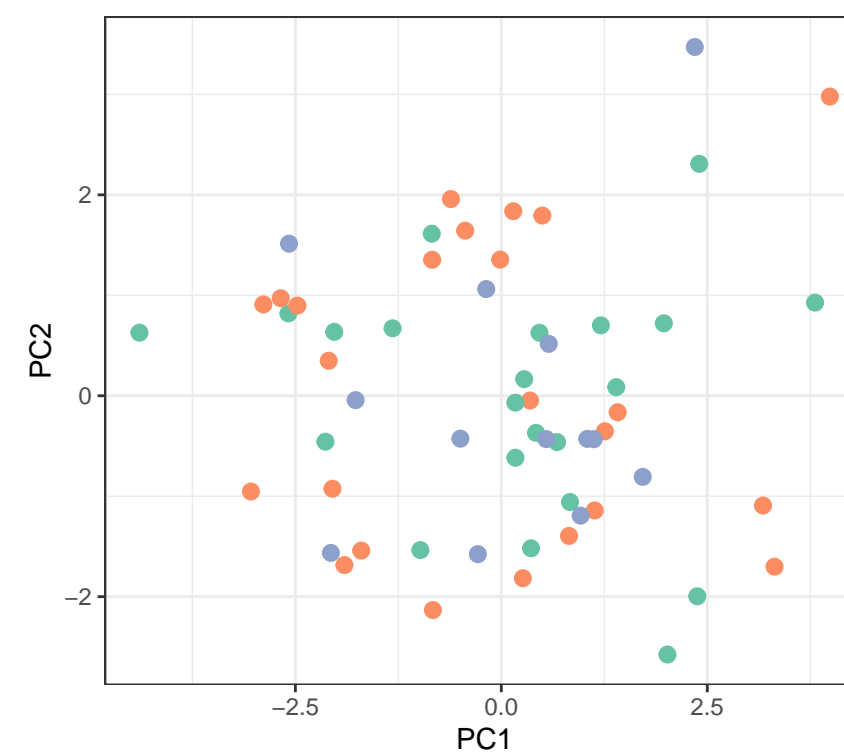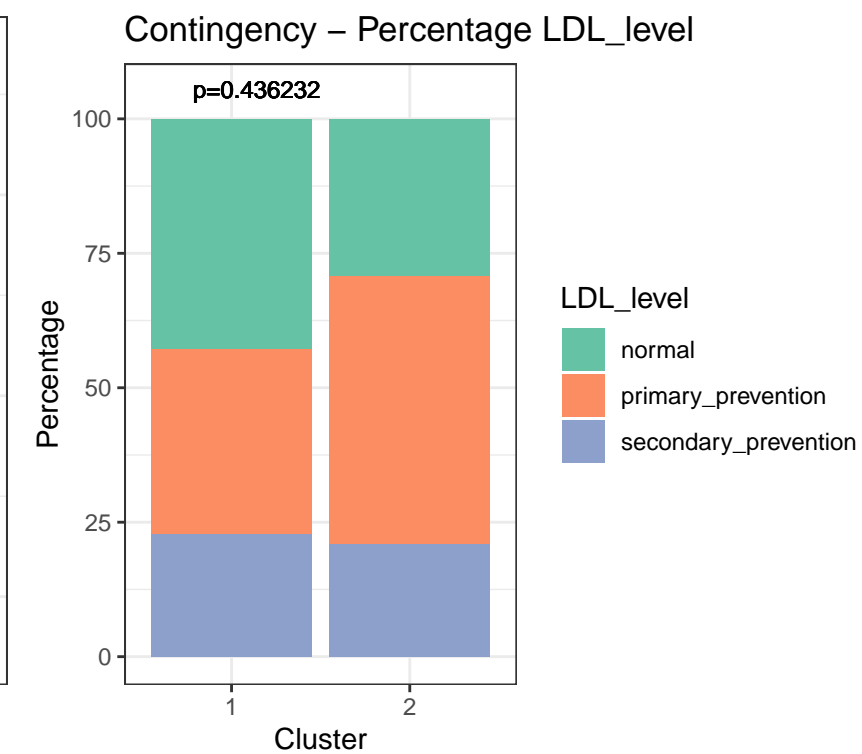

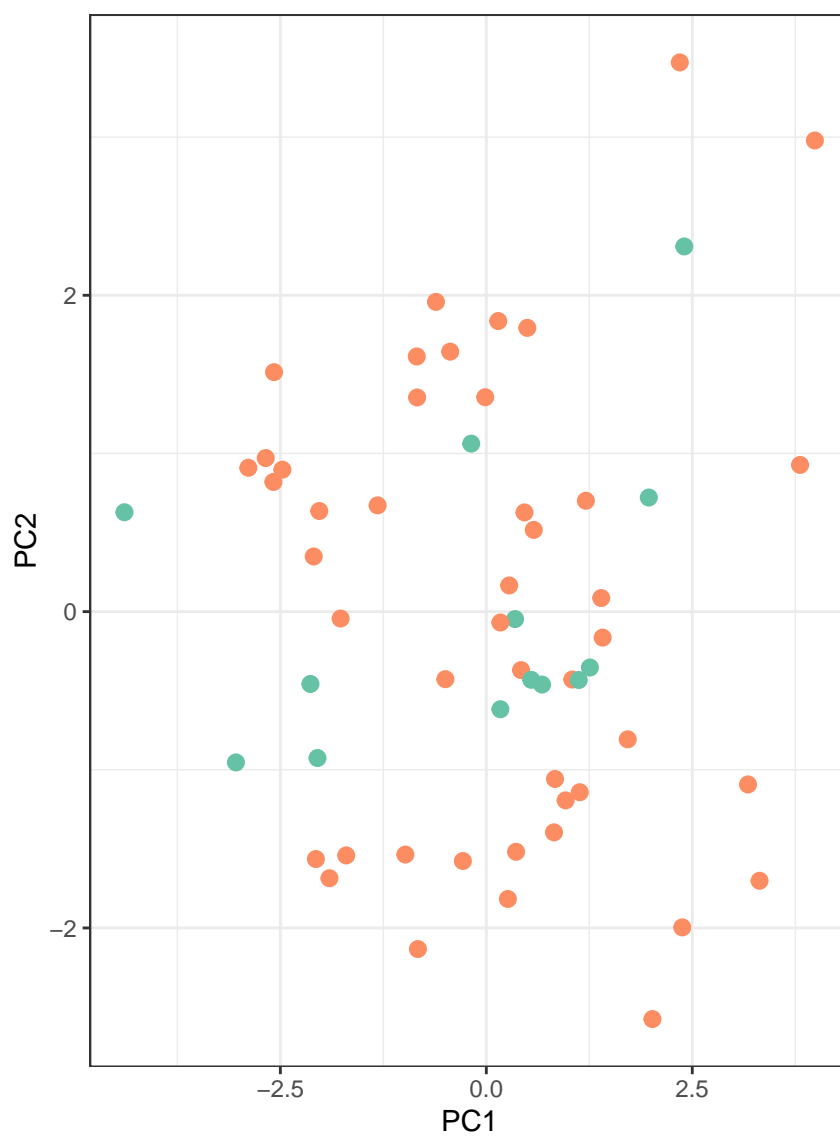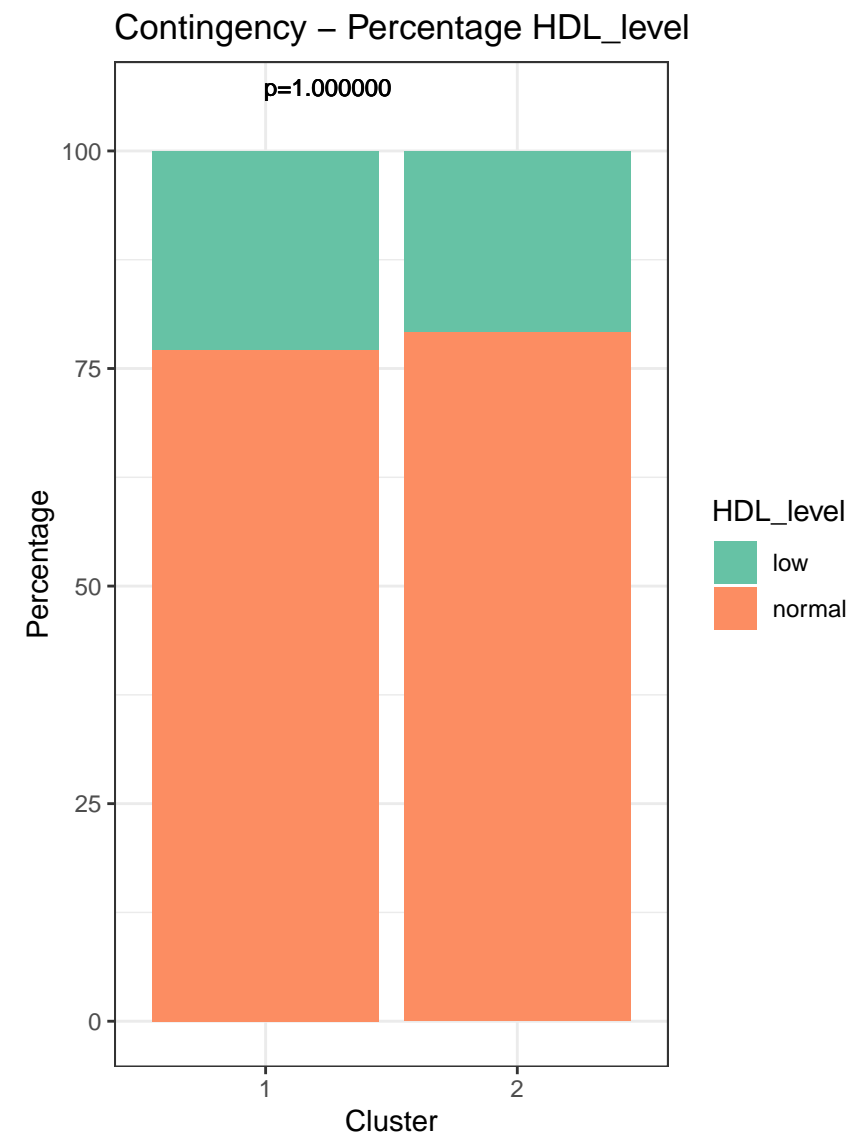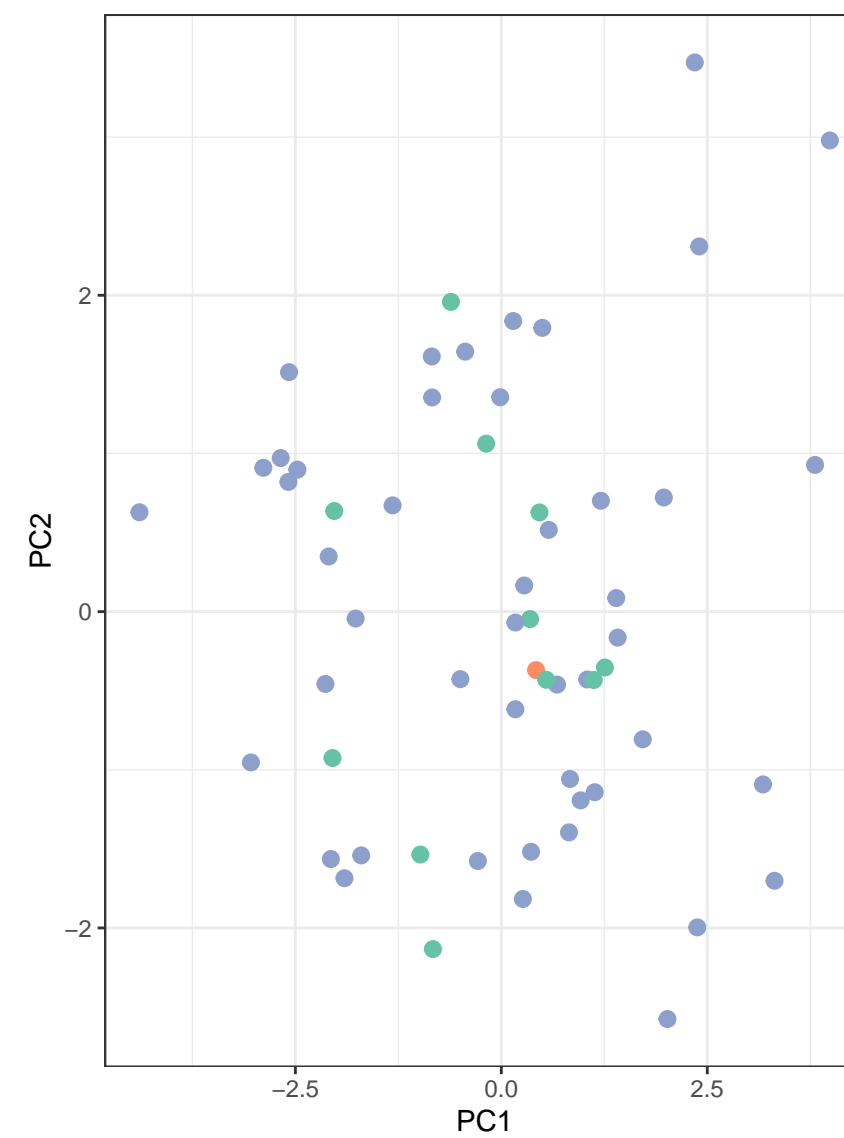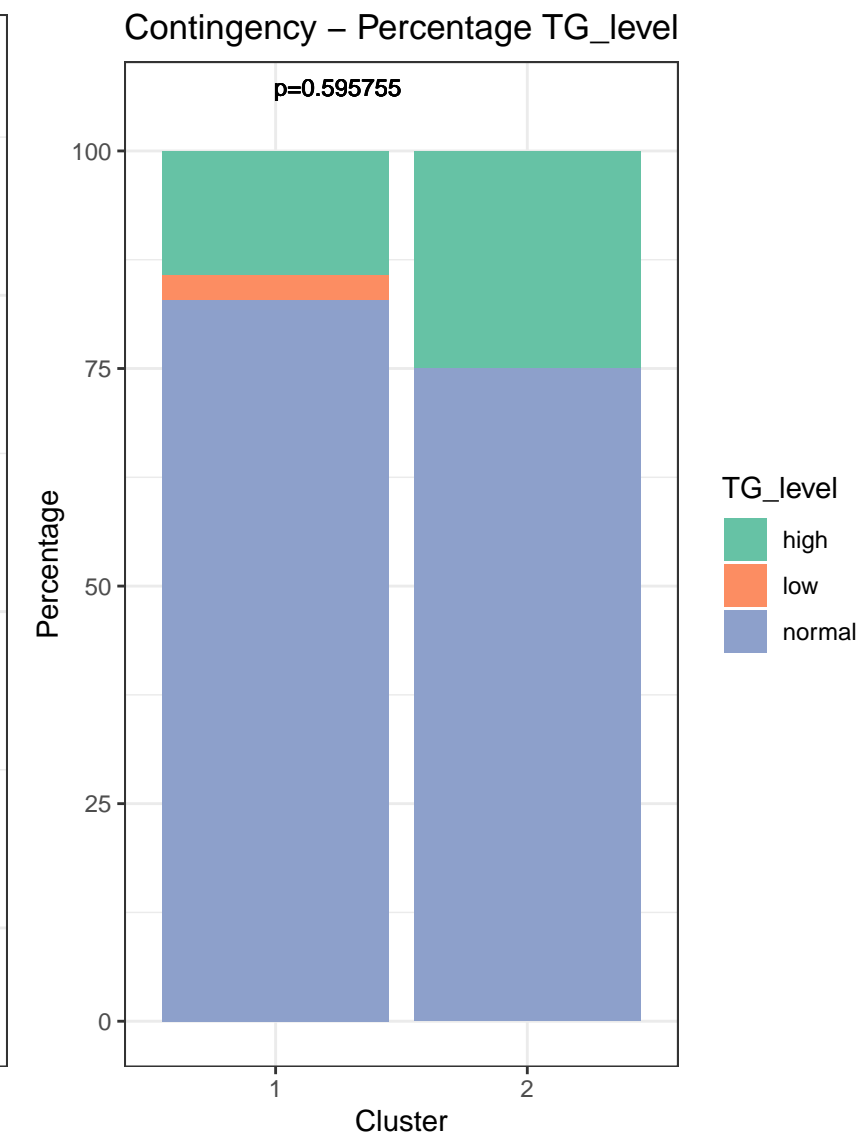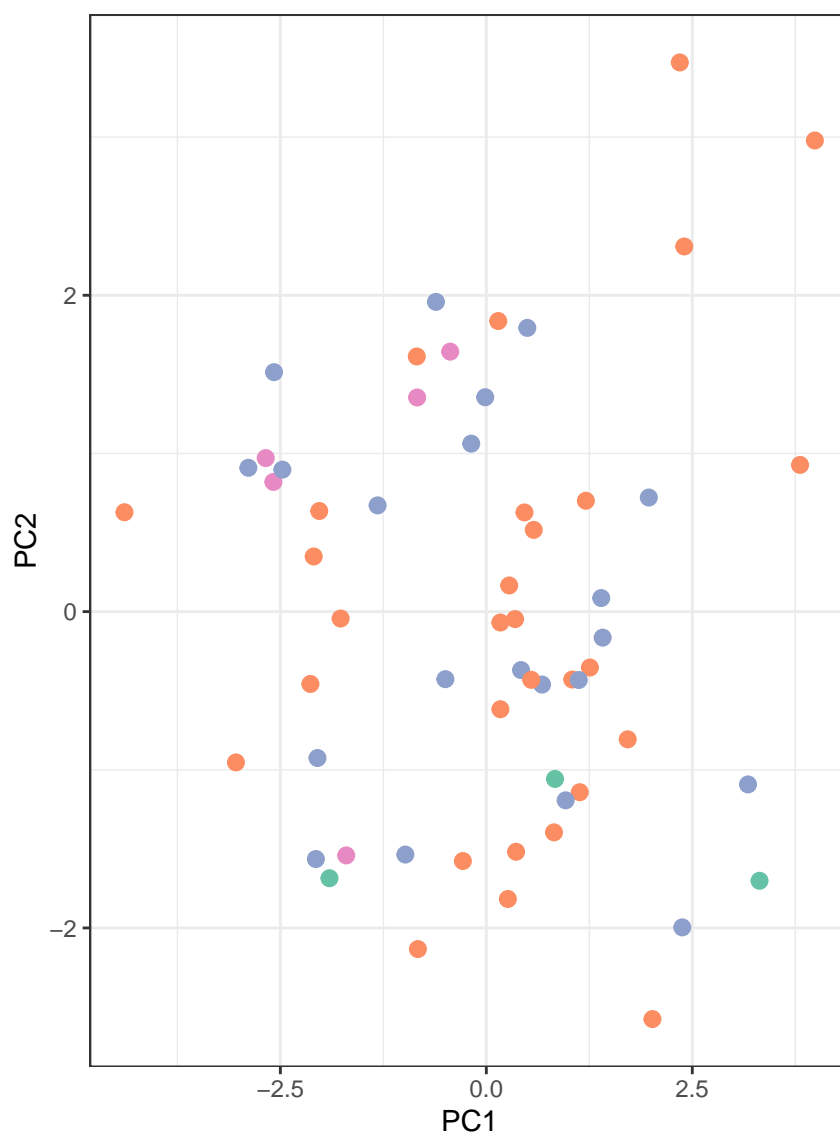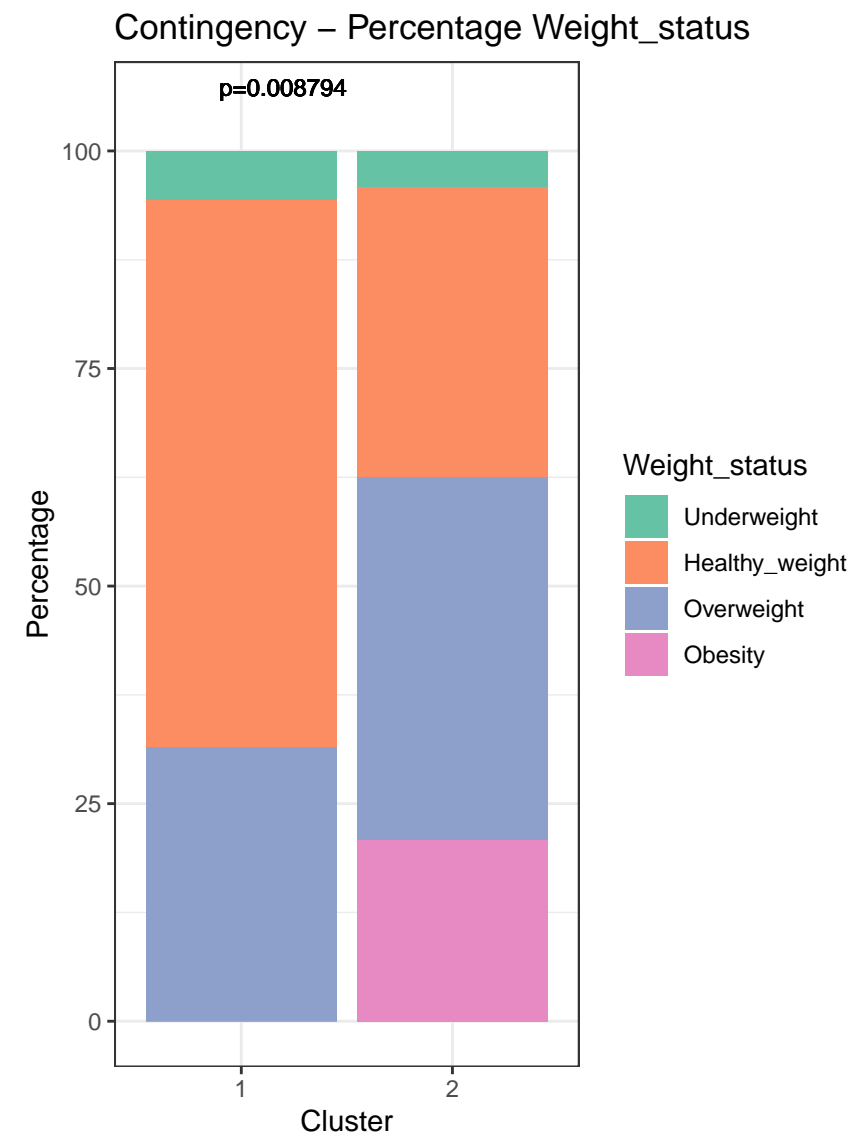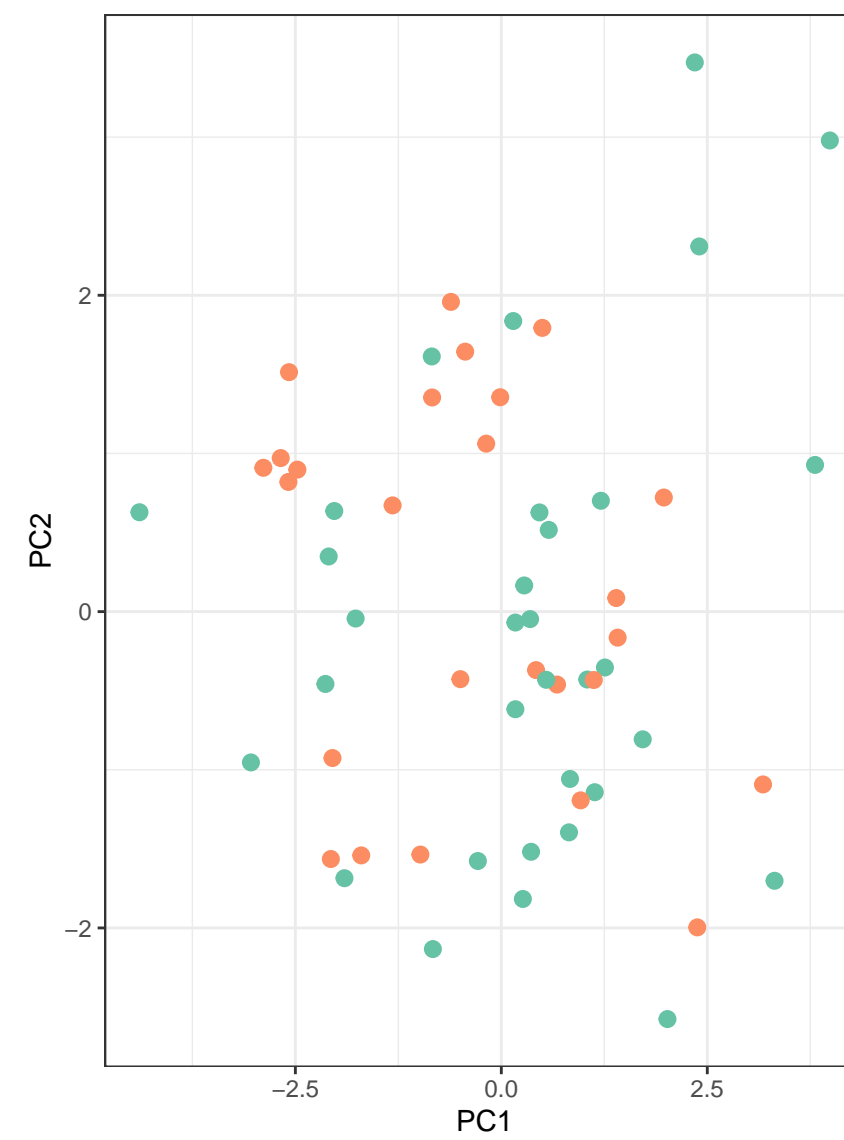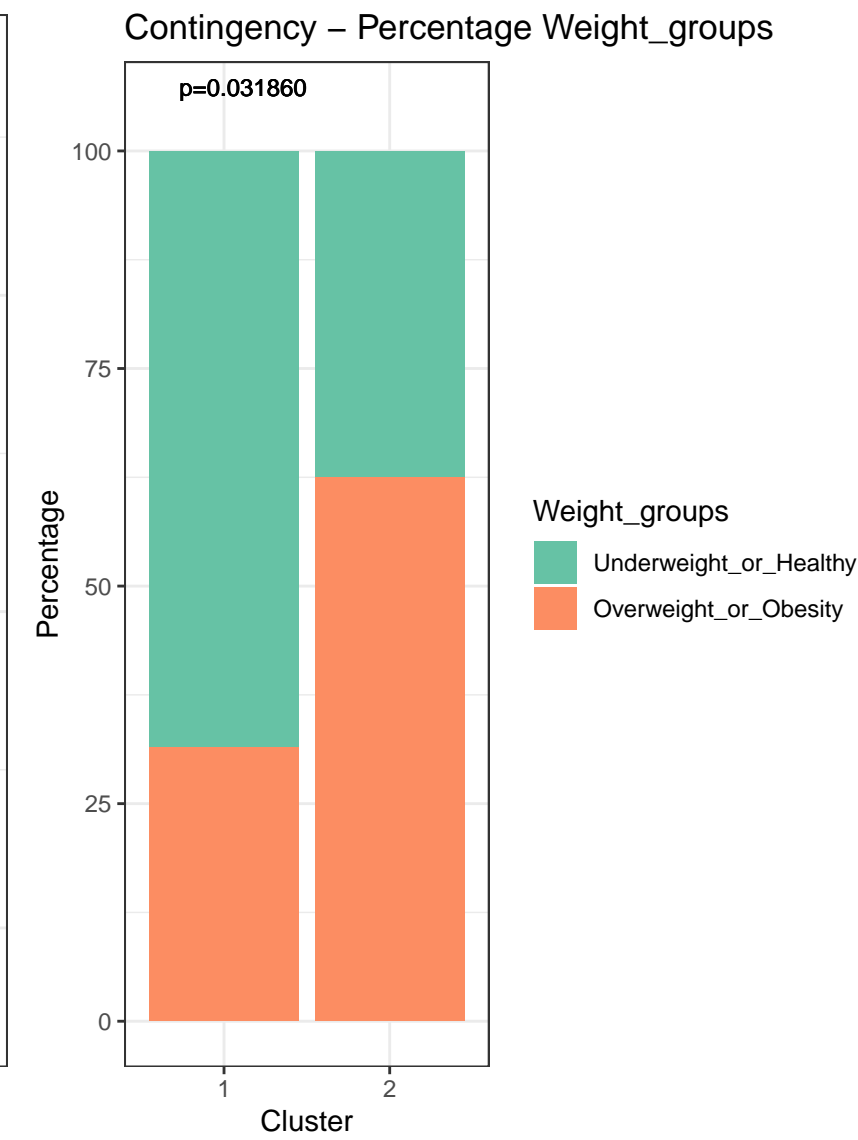

Supplement: Supplementary file 1 [file molecules-29-05169-s001.zip › molecules-3242400-supplementary/S13_disease_only_acylcarnitines.pdf]

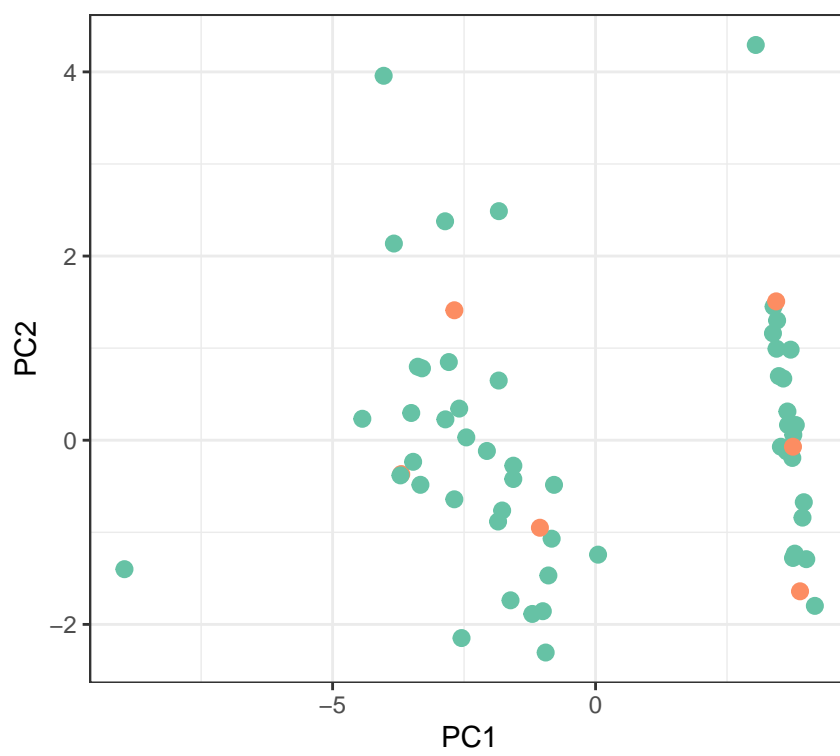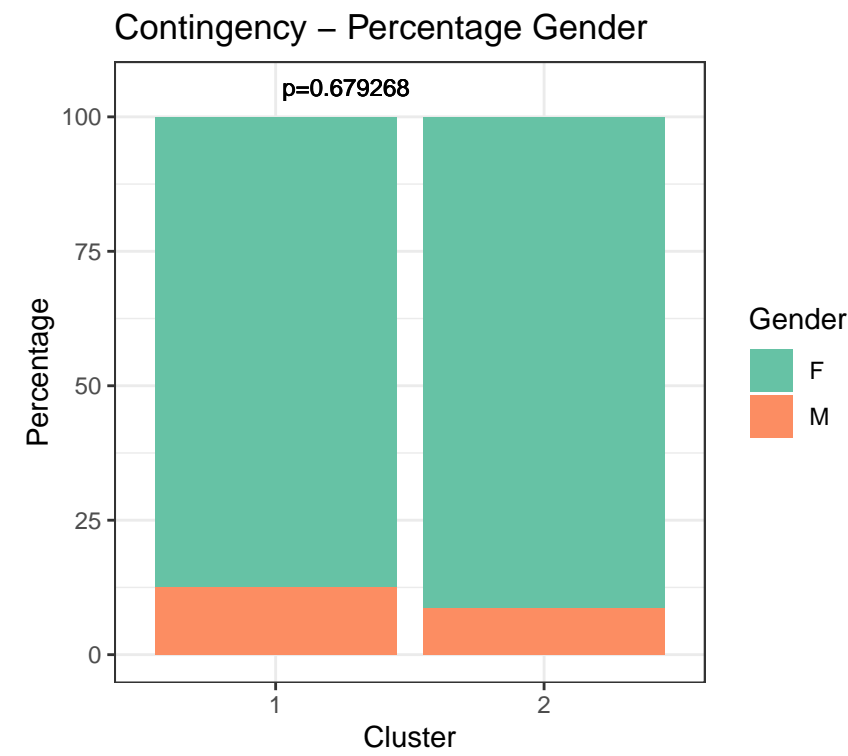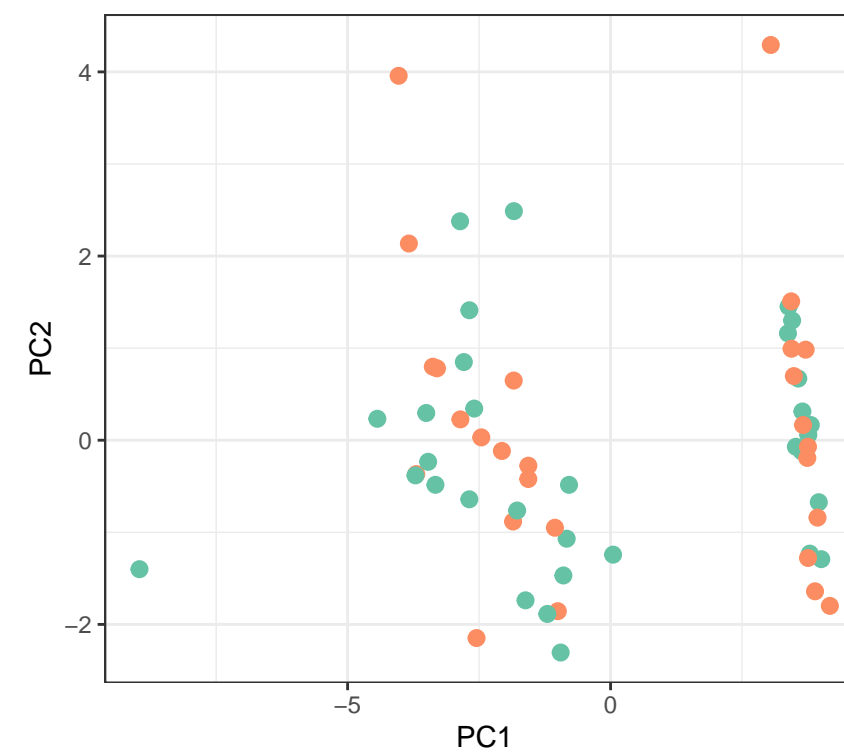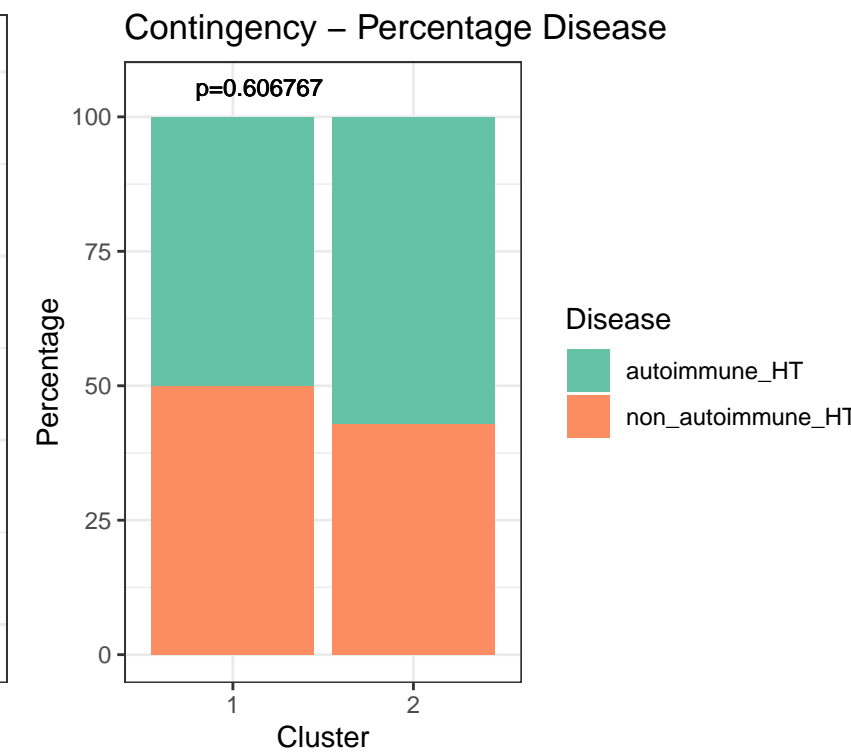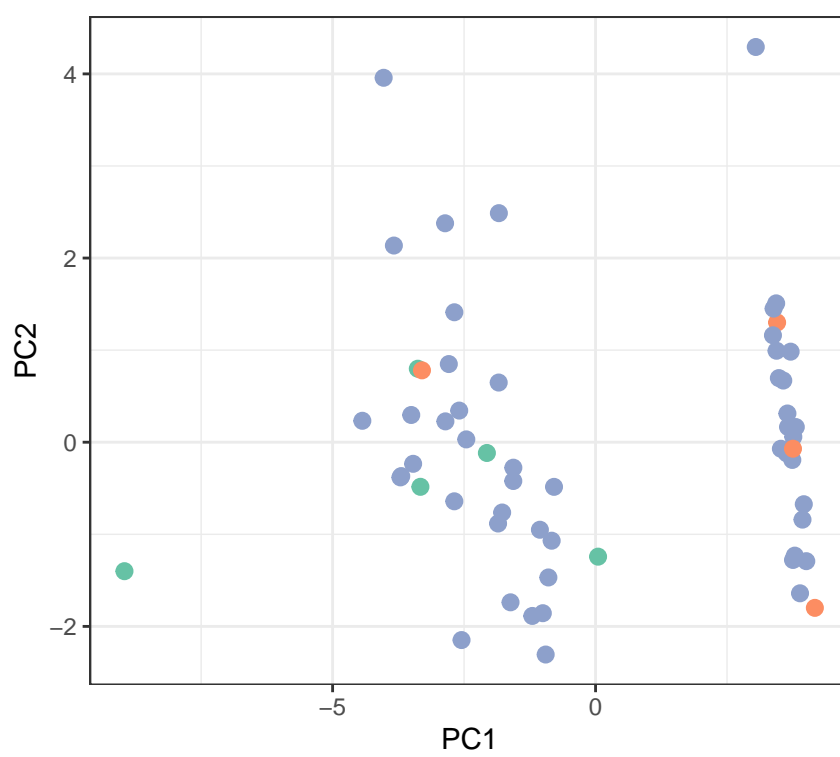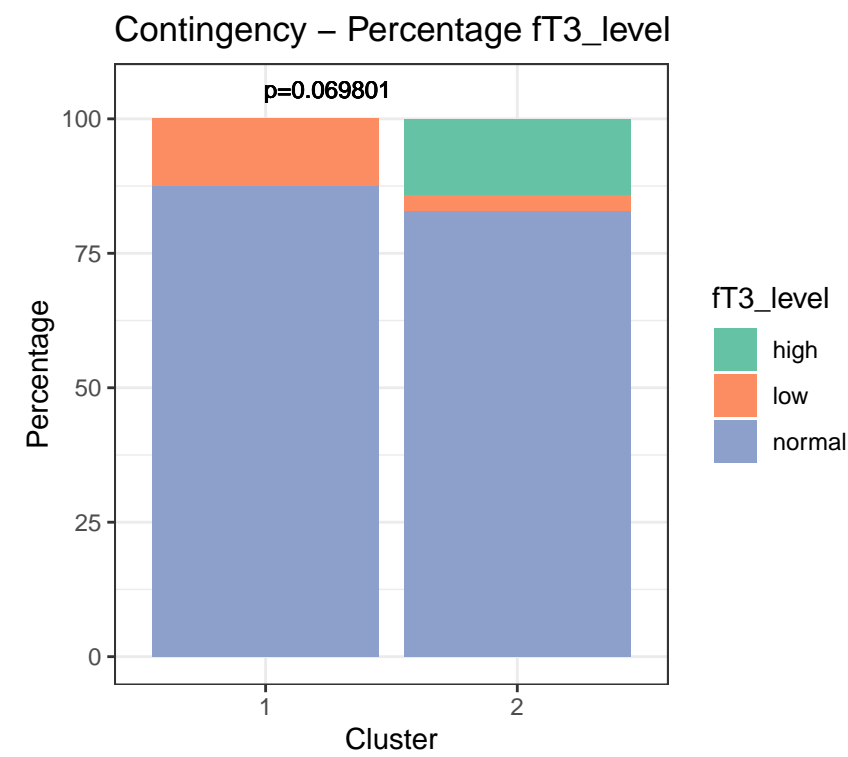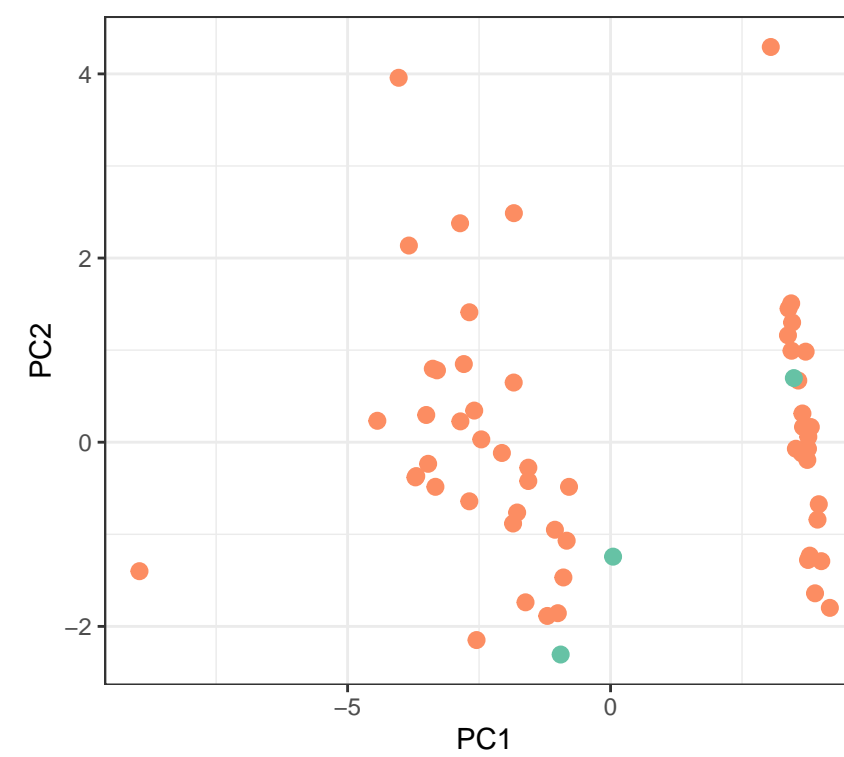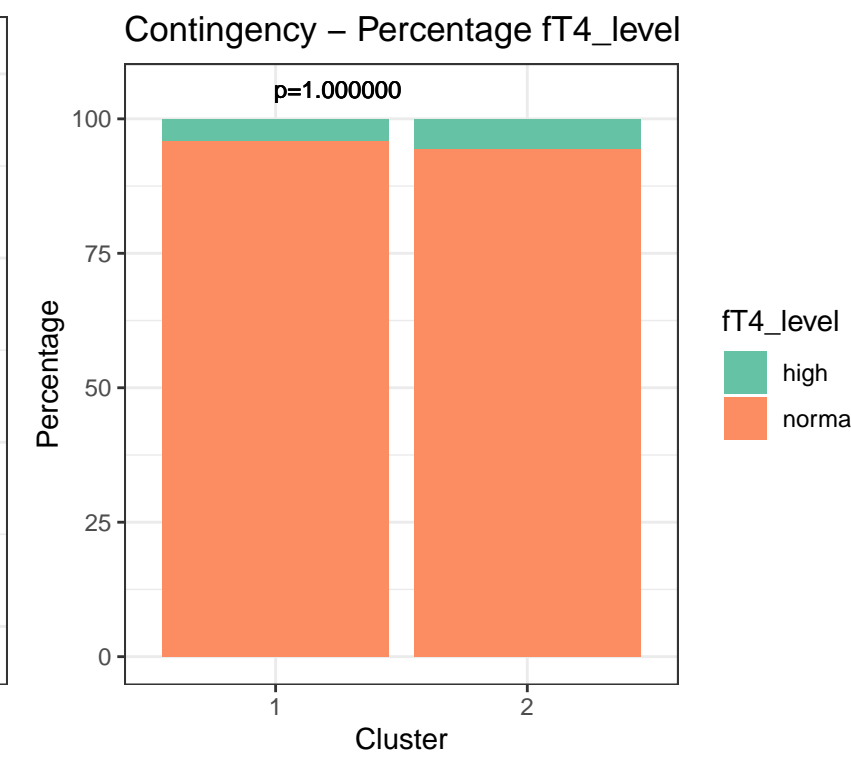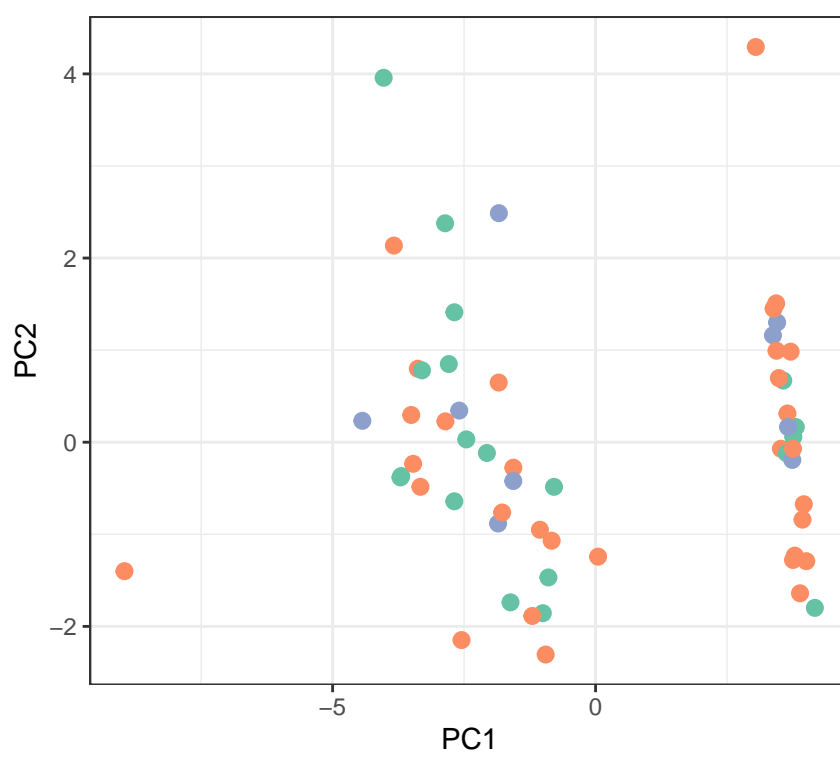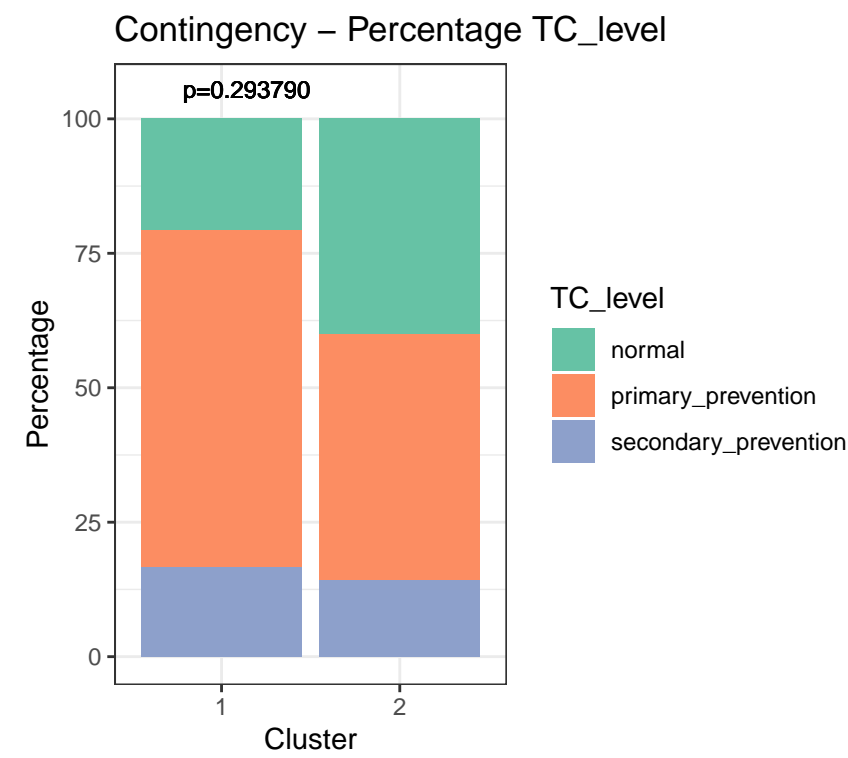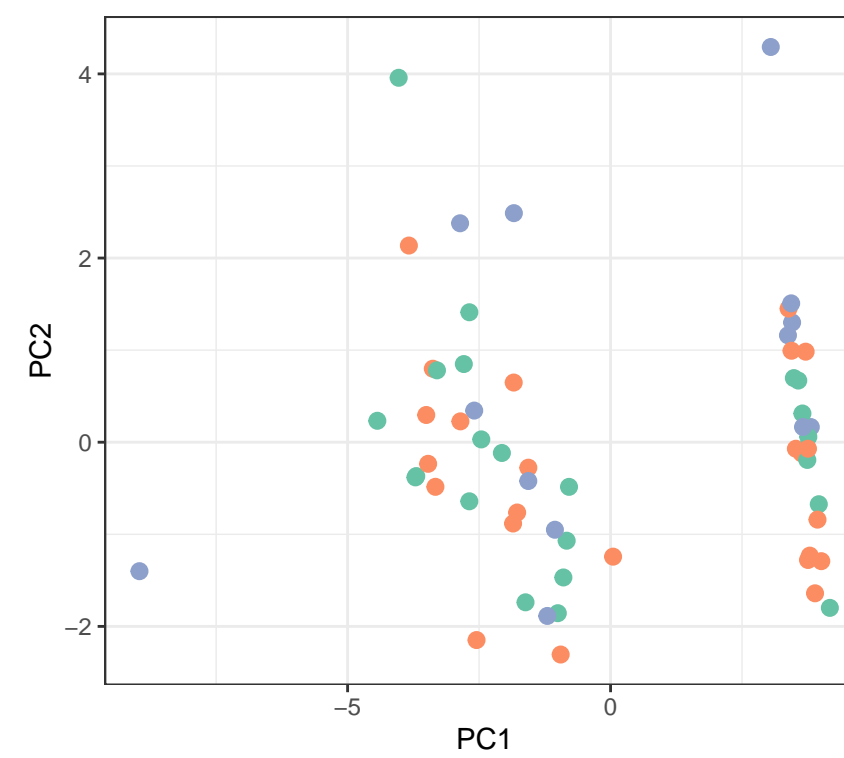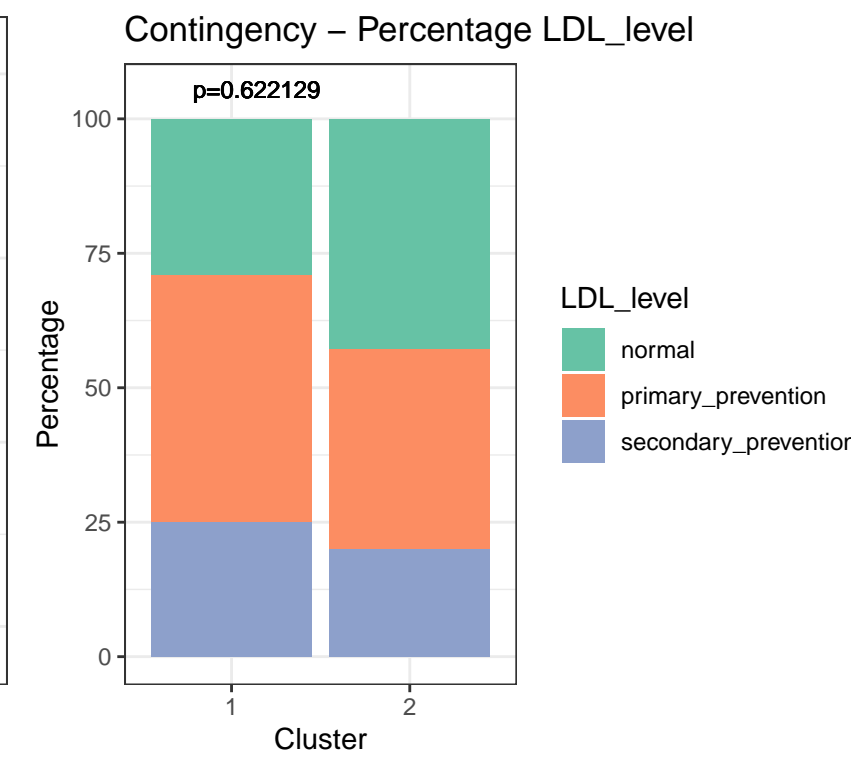

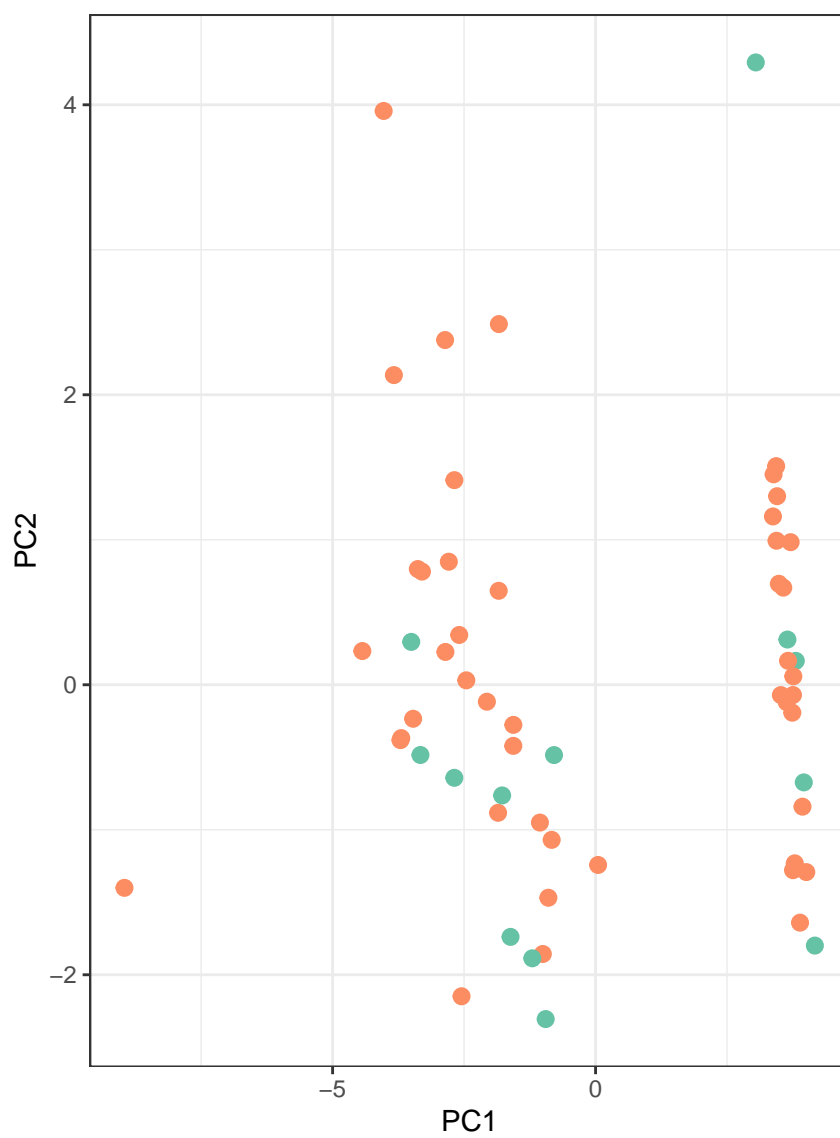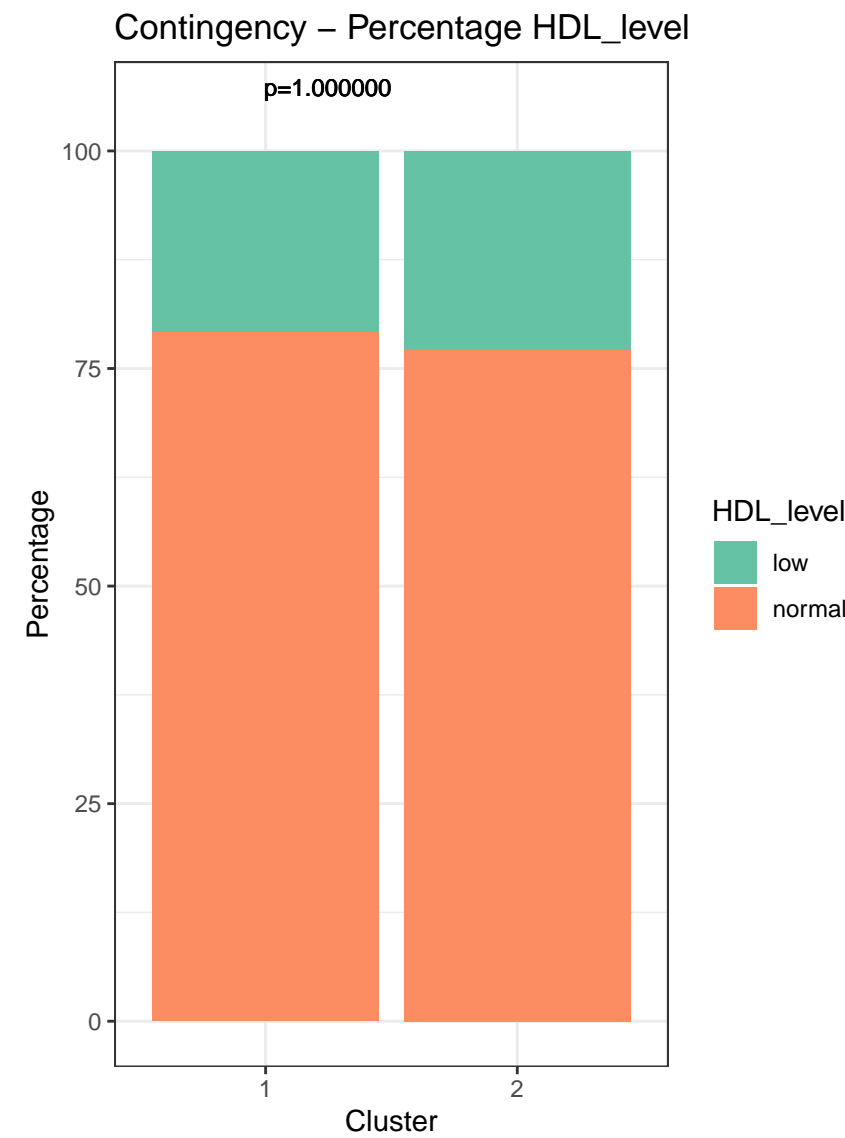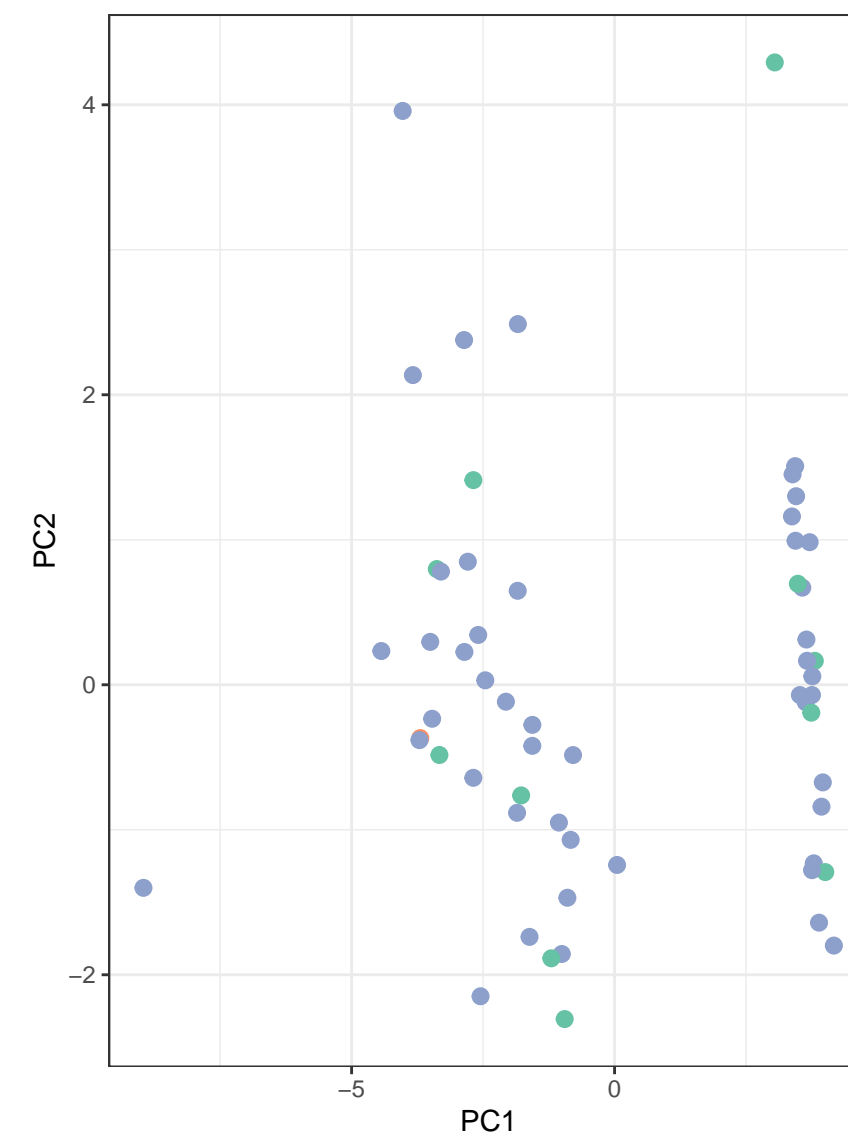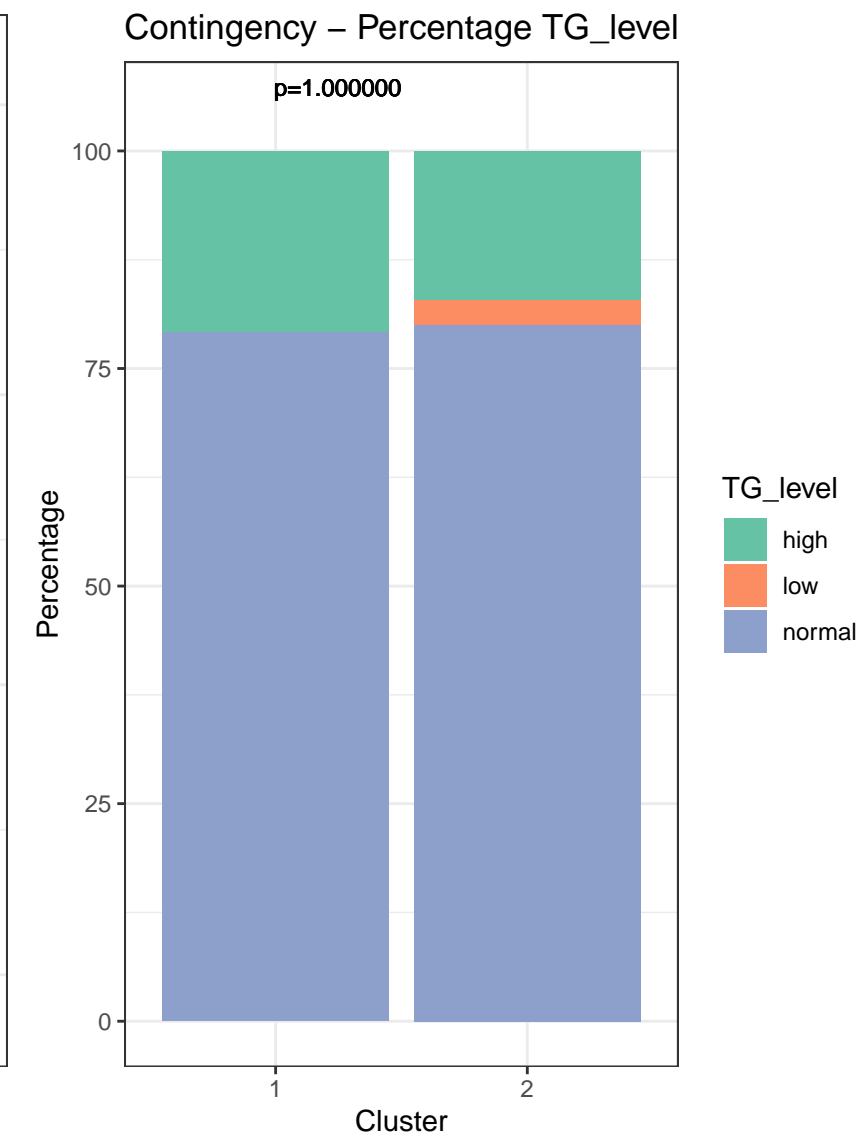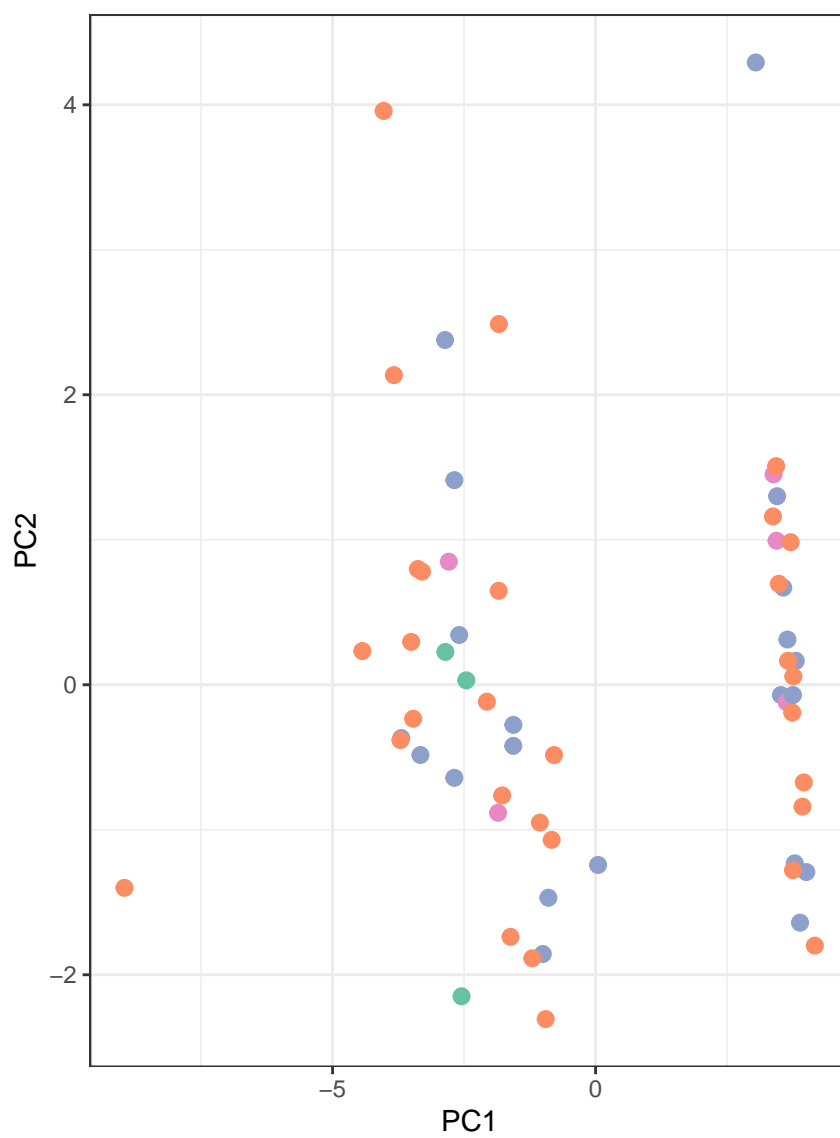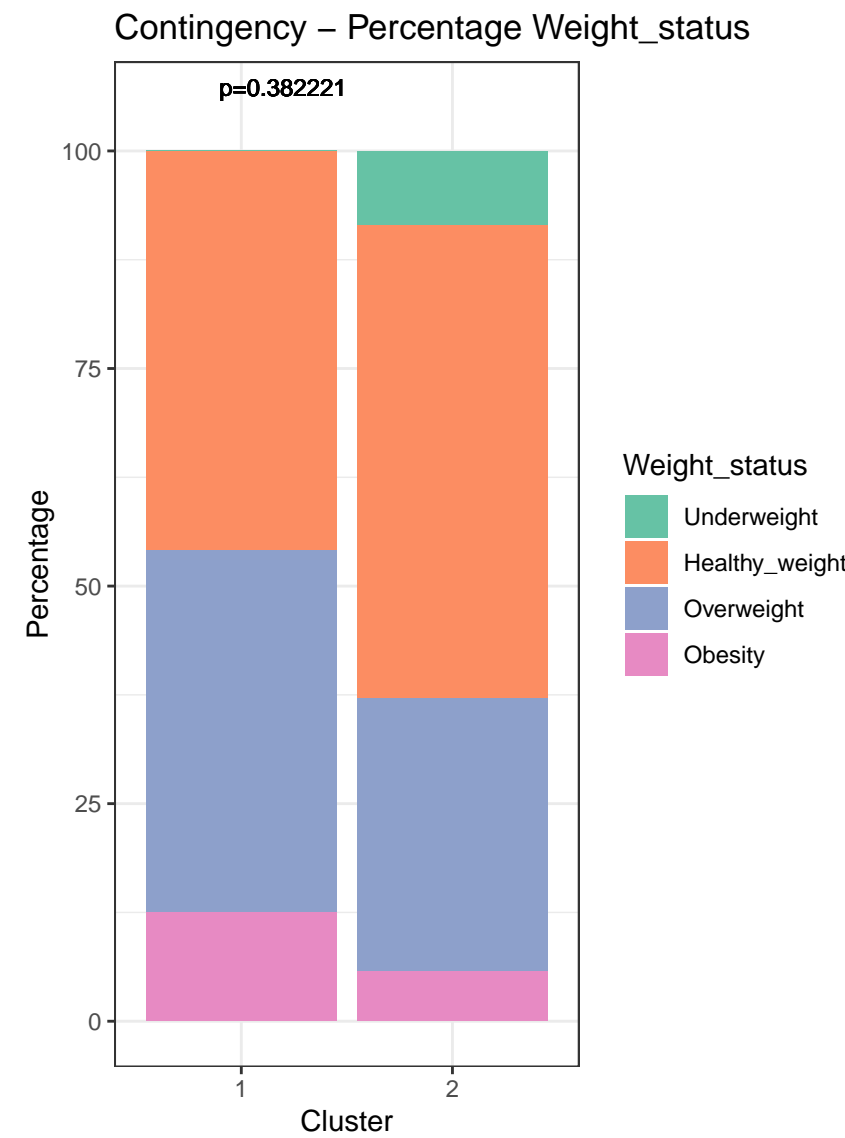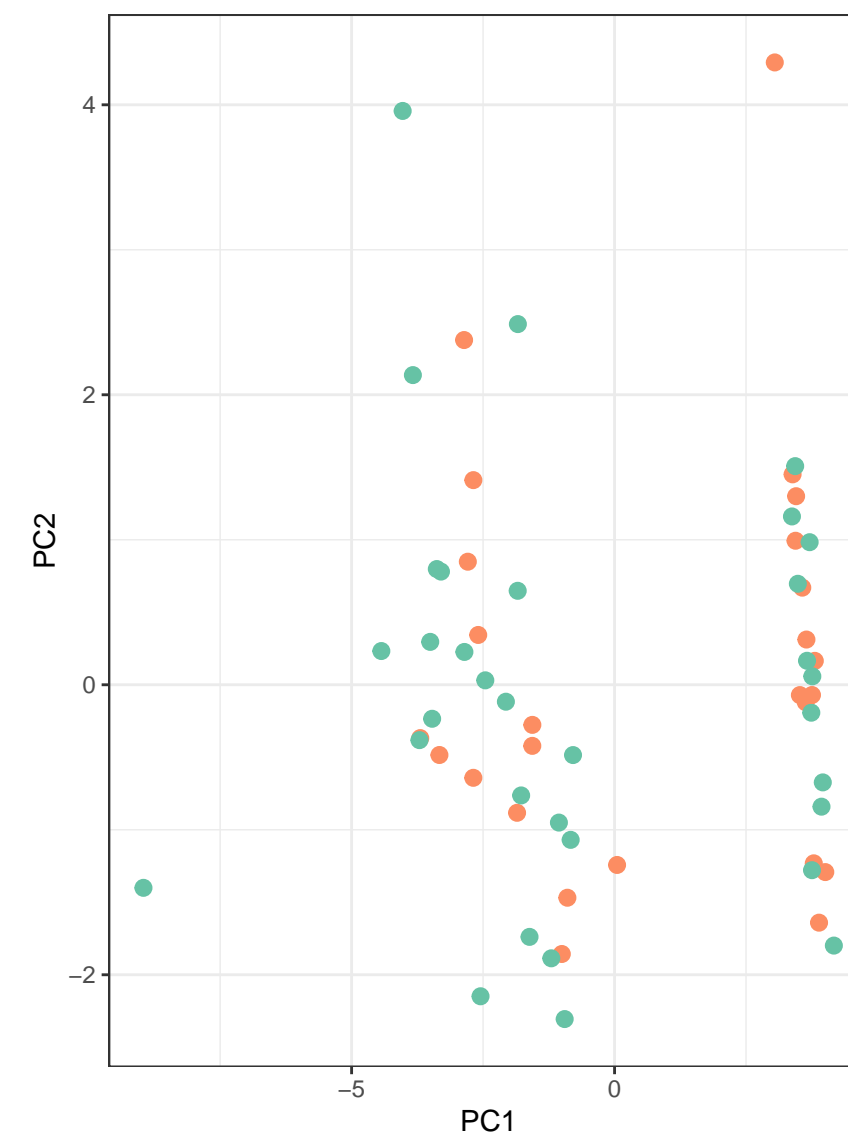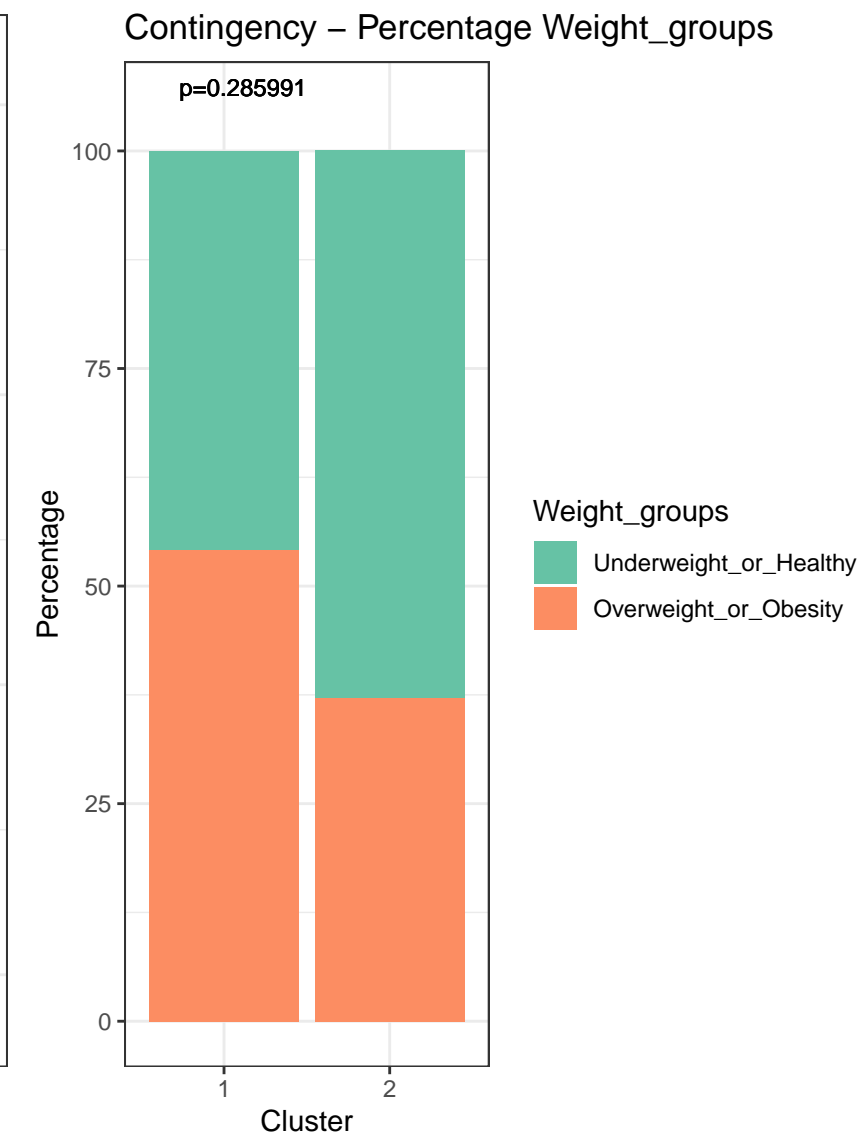

Supplement: Supplementary file 1 [file molecules-29-05169-s001.zip › molecules-3242400-supplementary/S14_disease_only_glycerophospholipids.pdf]

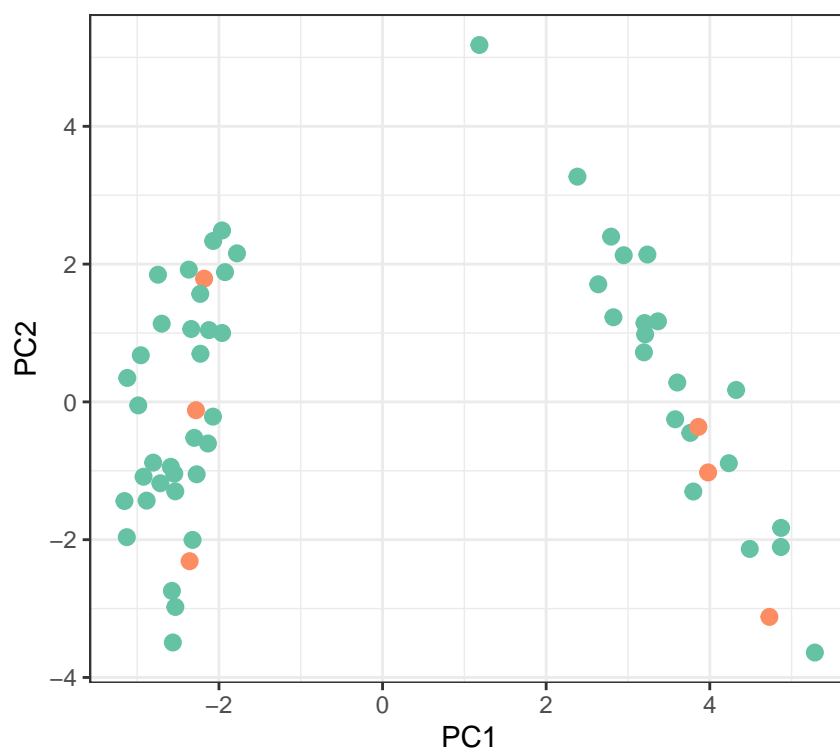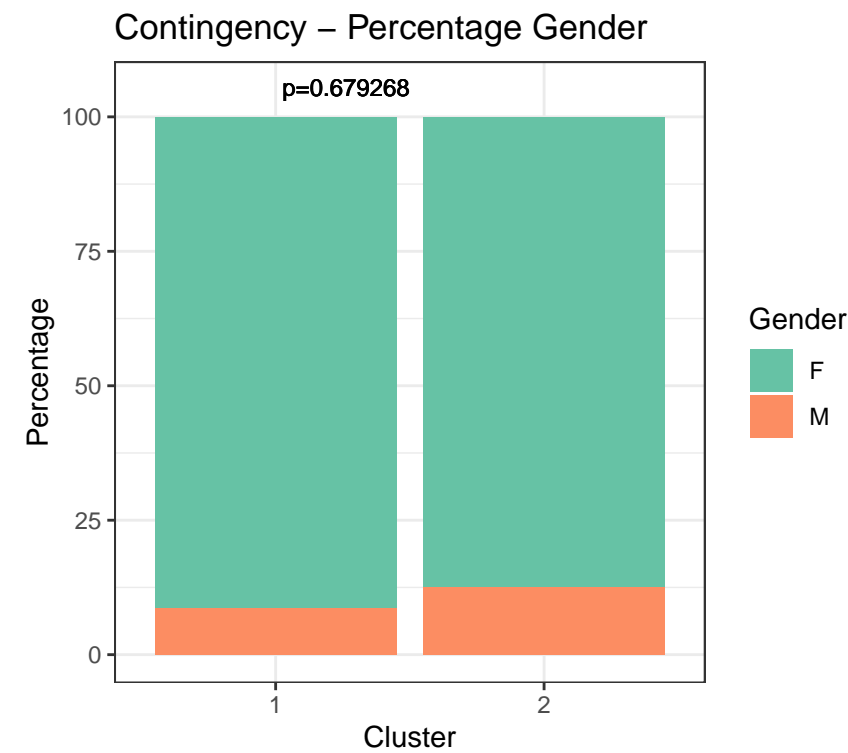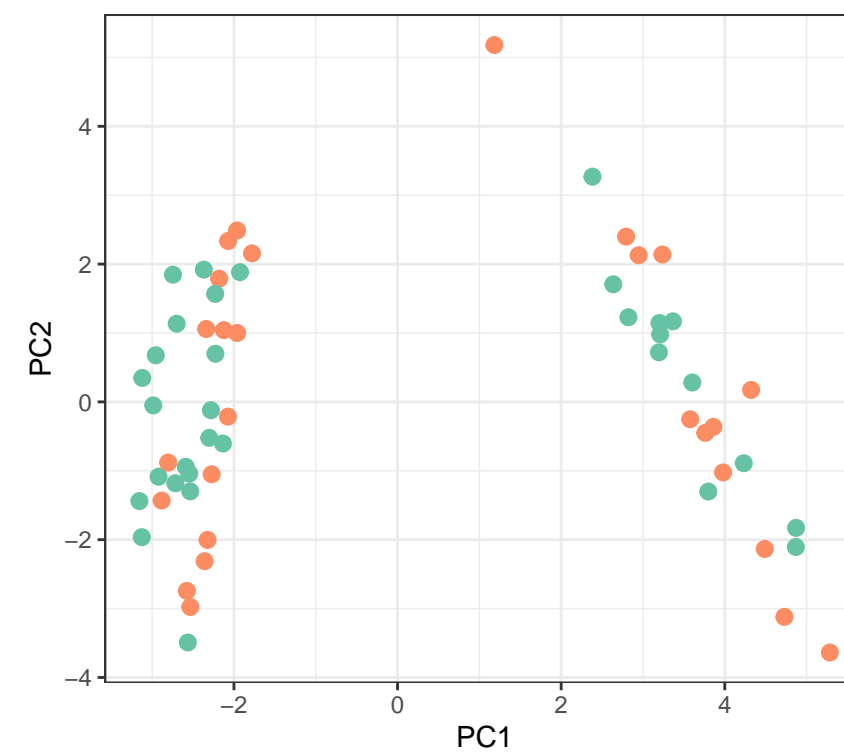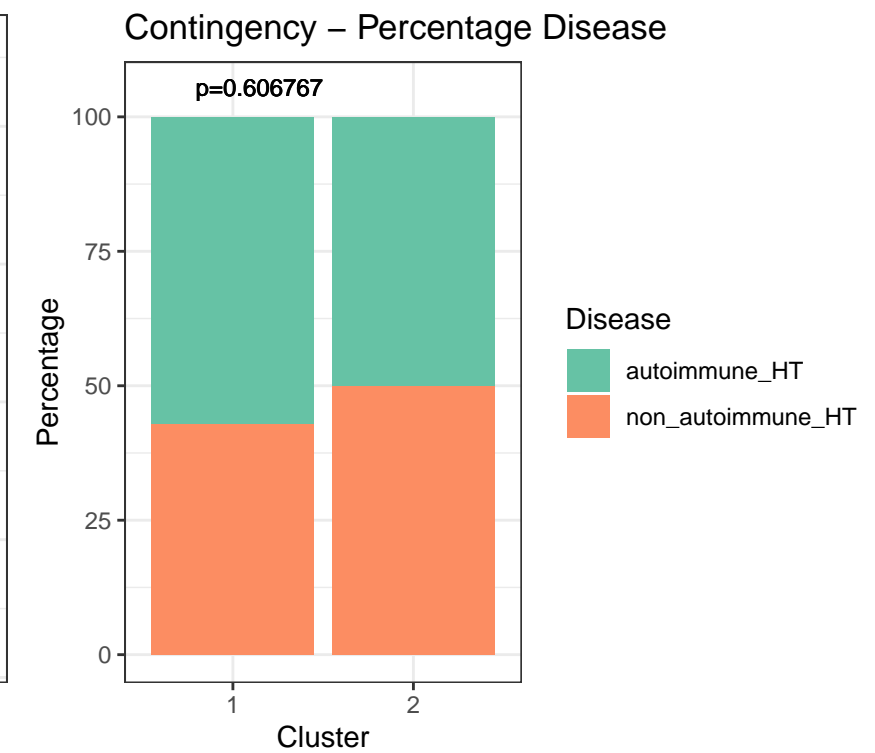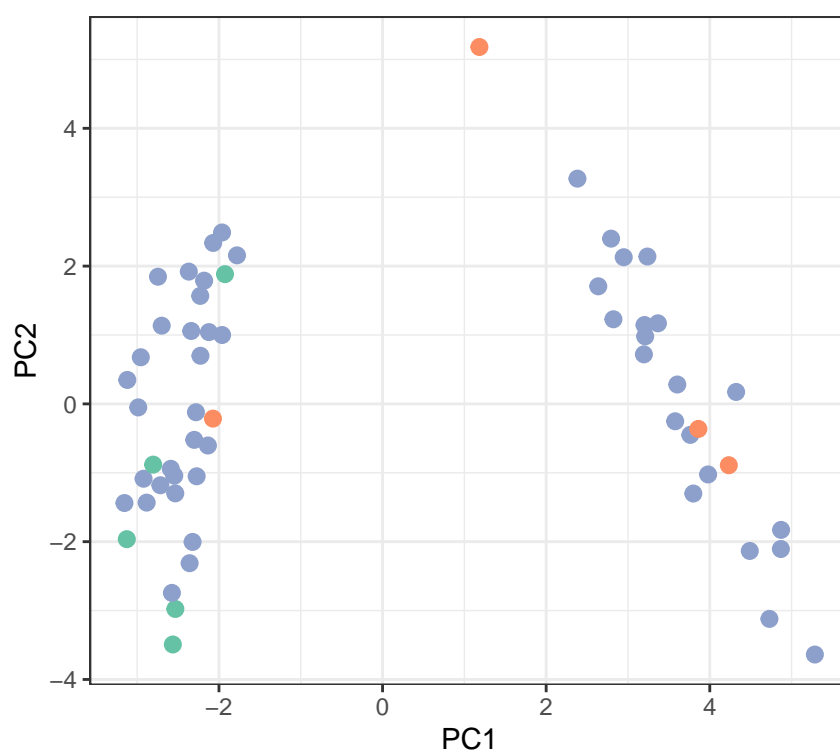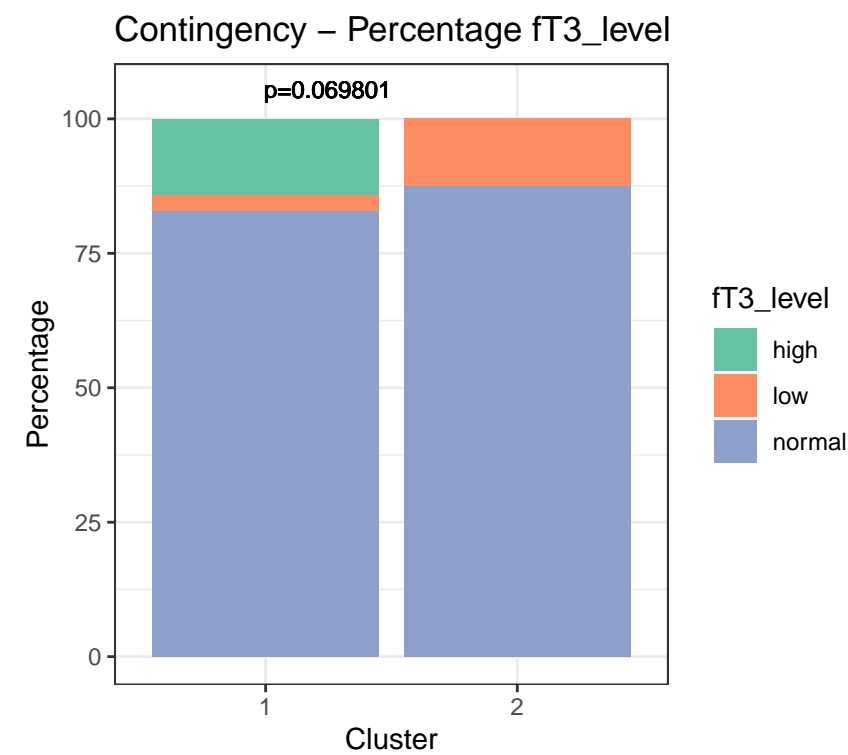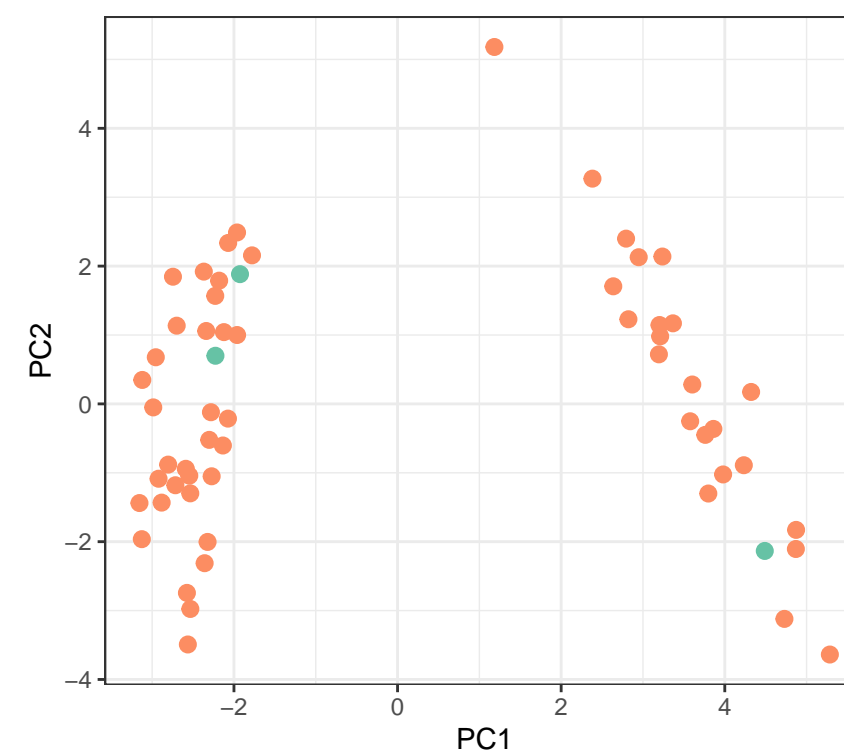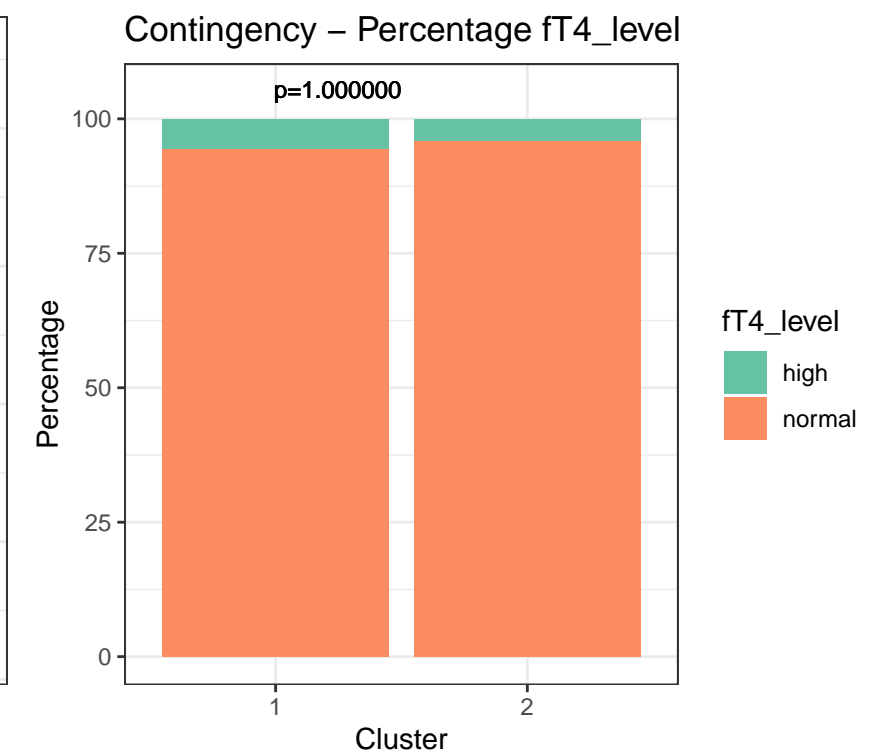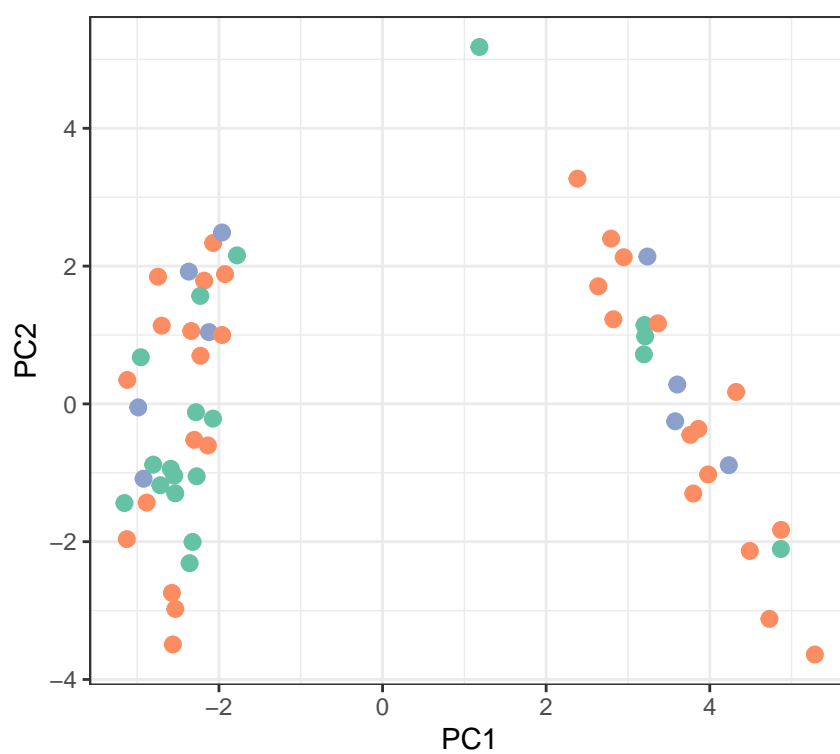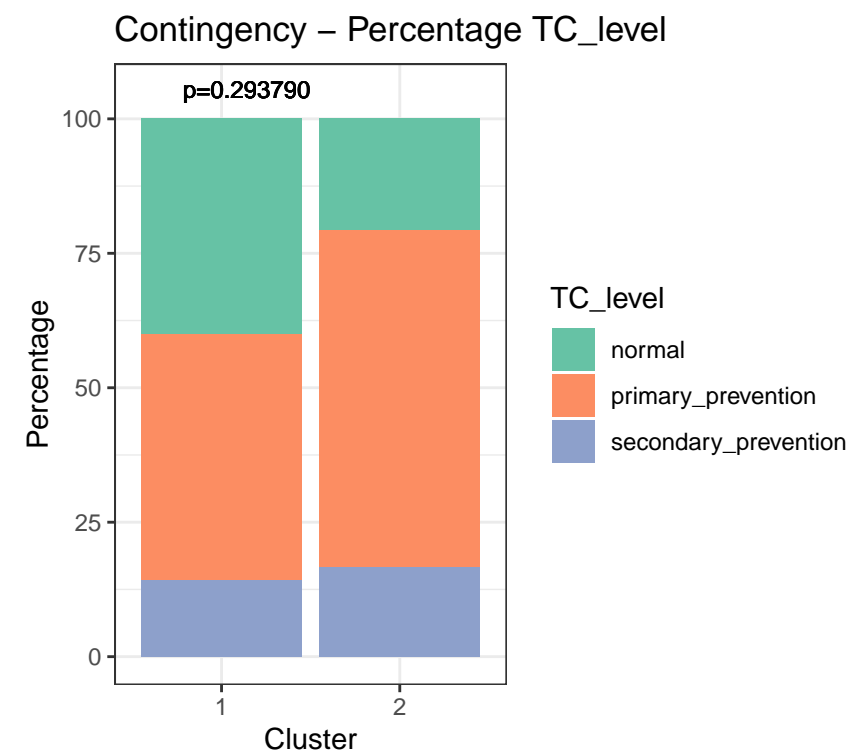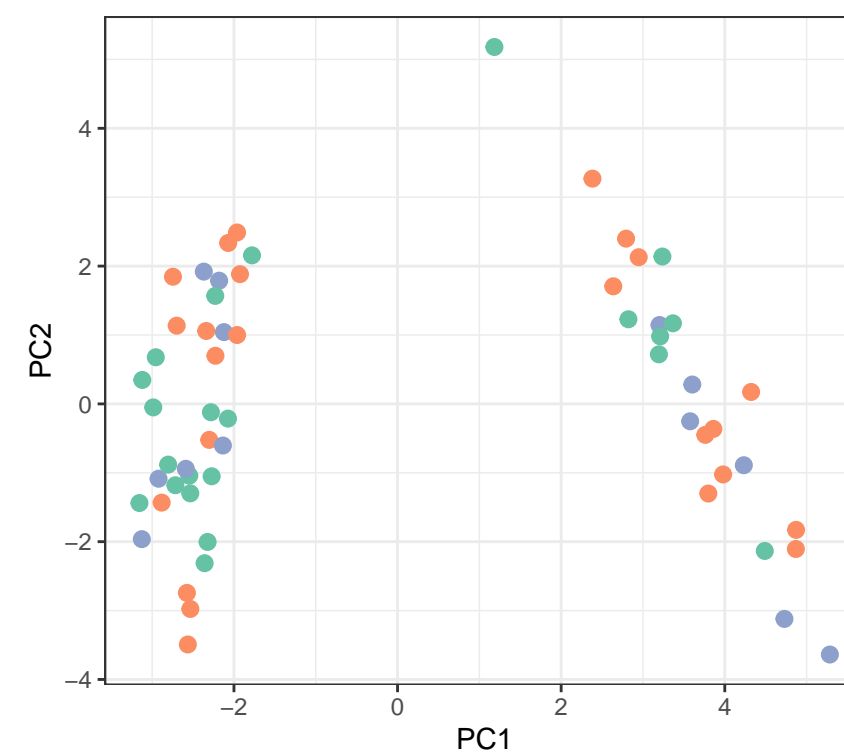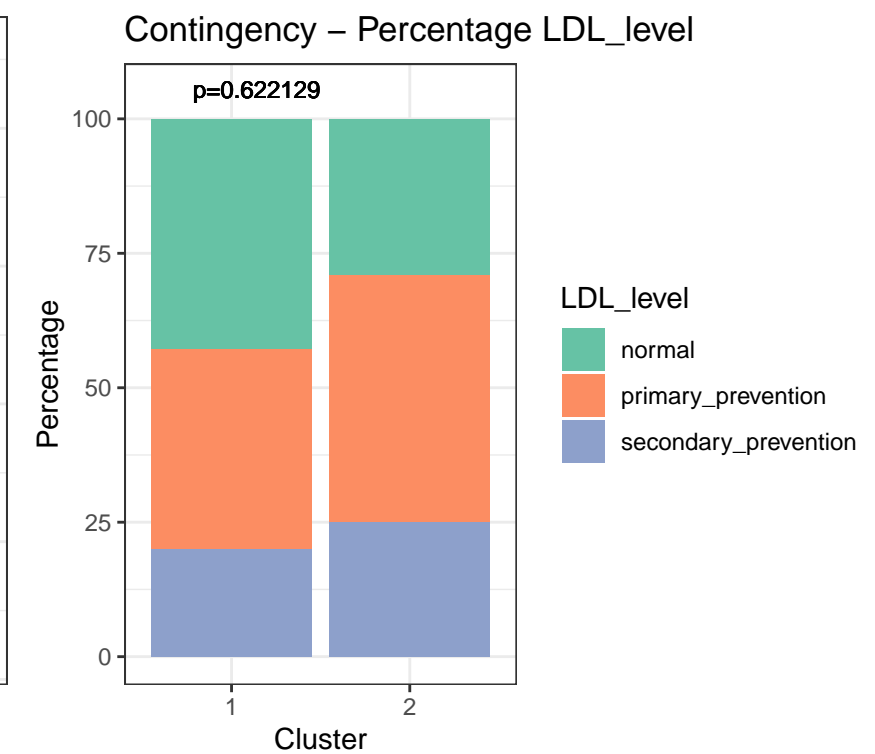

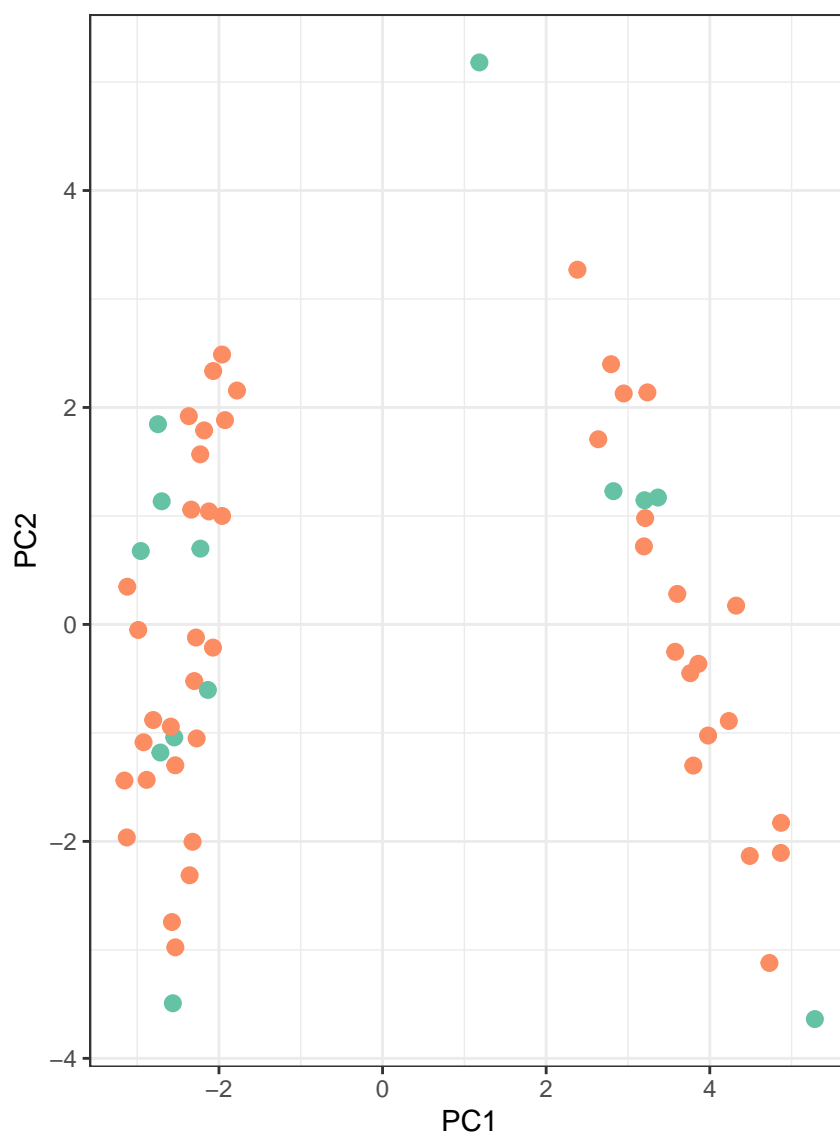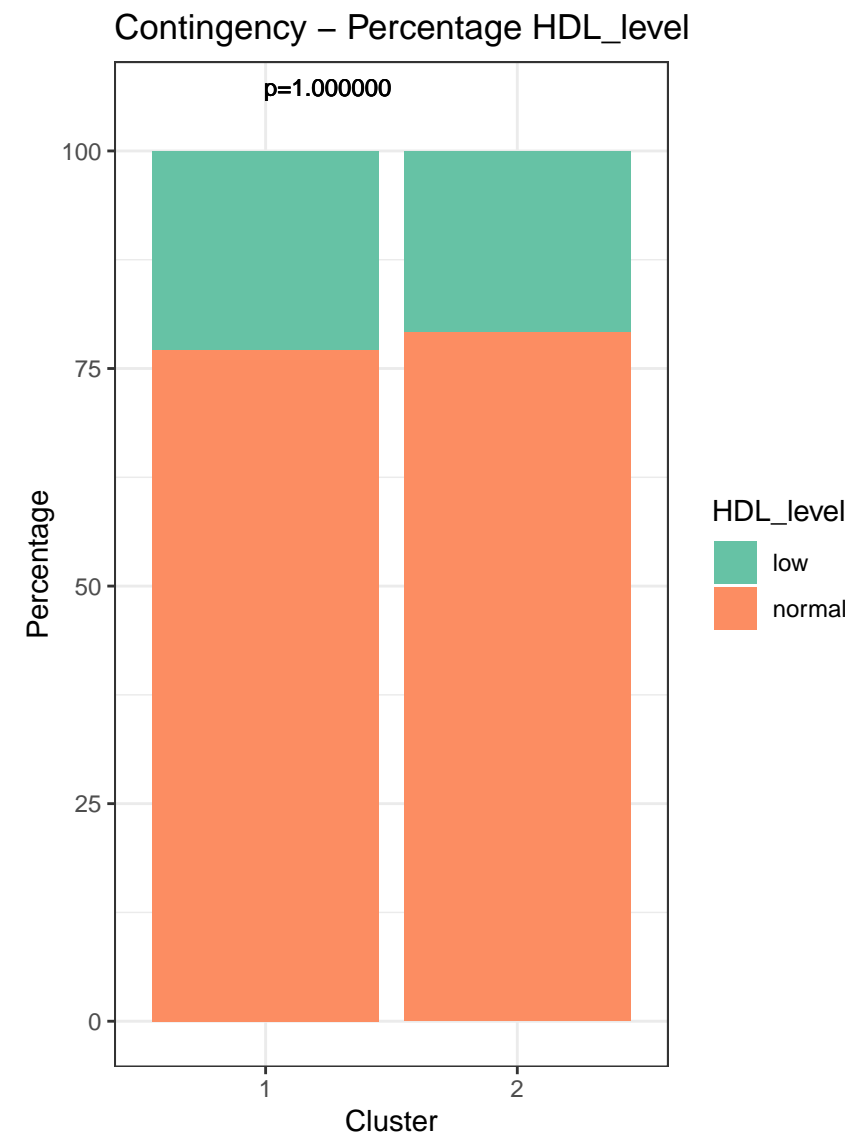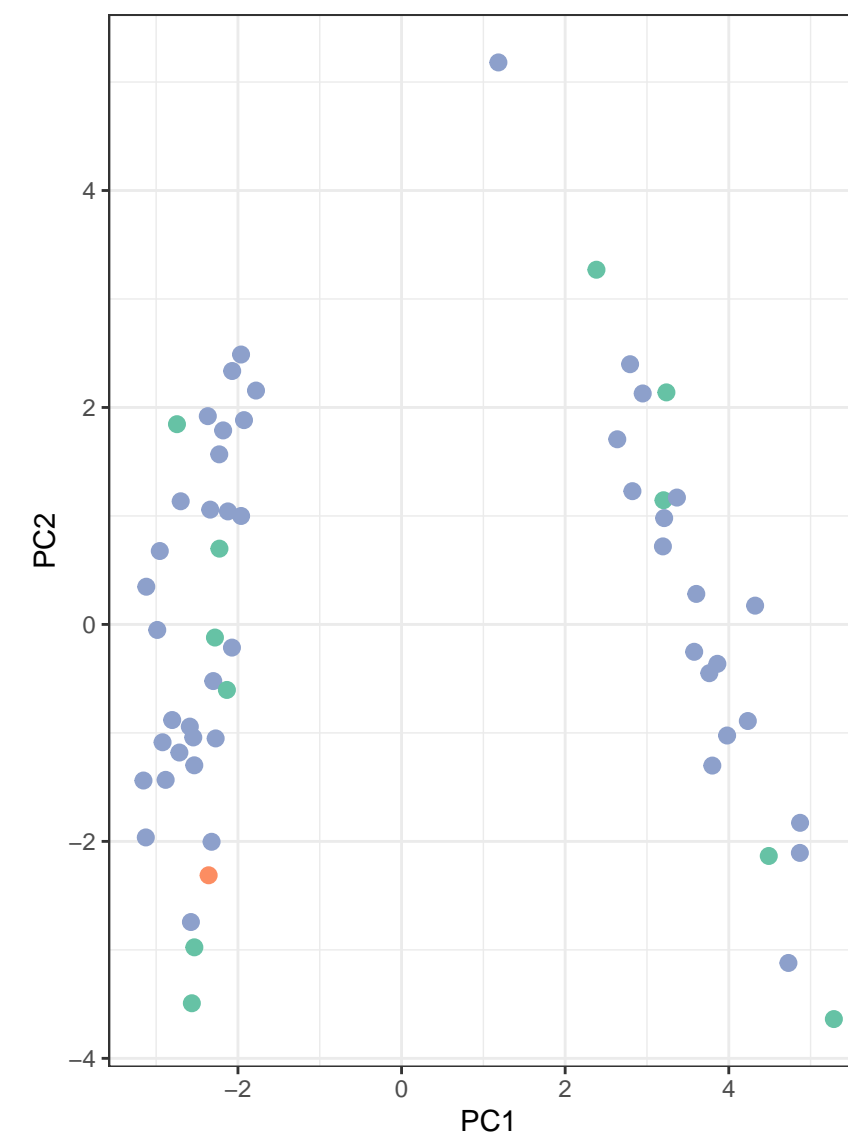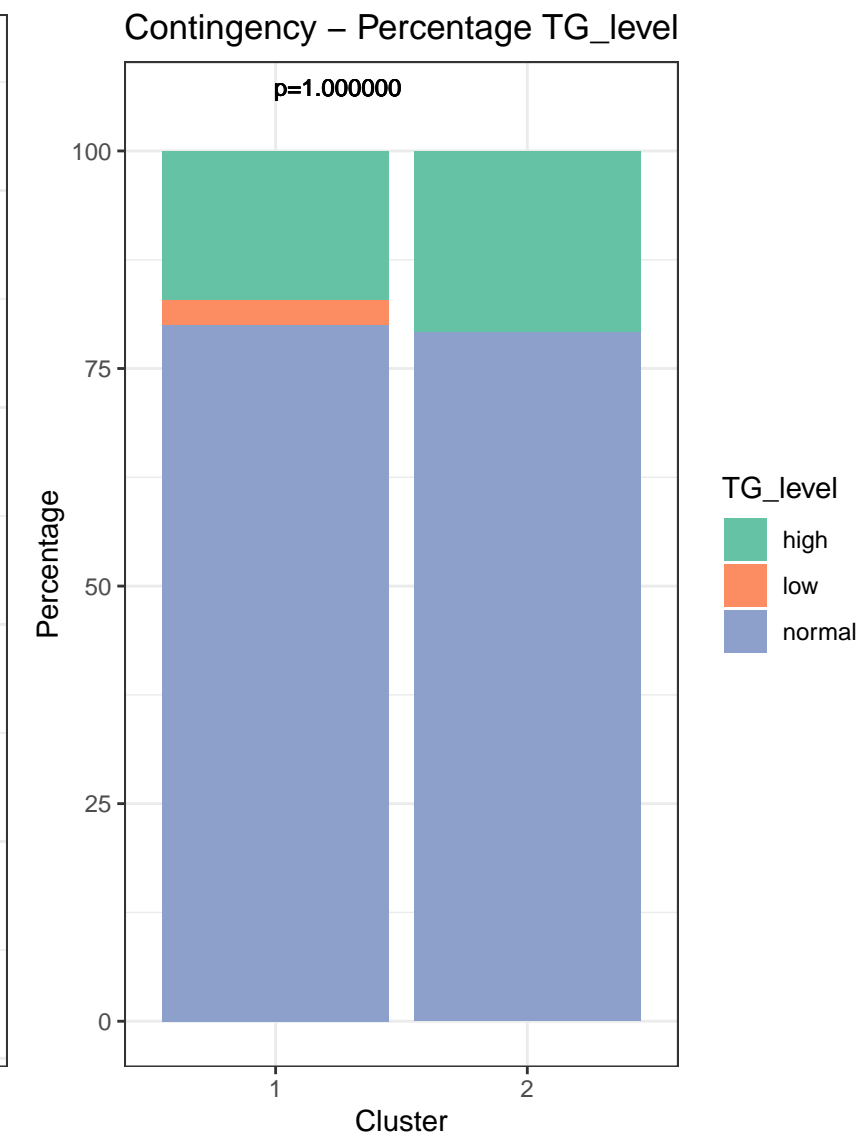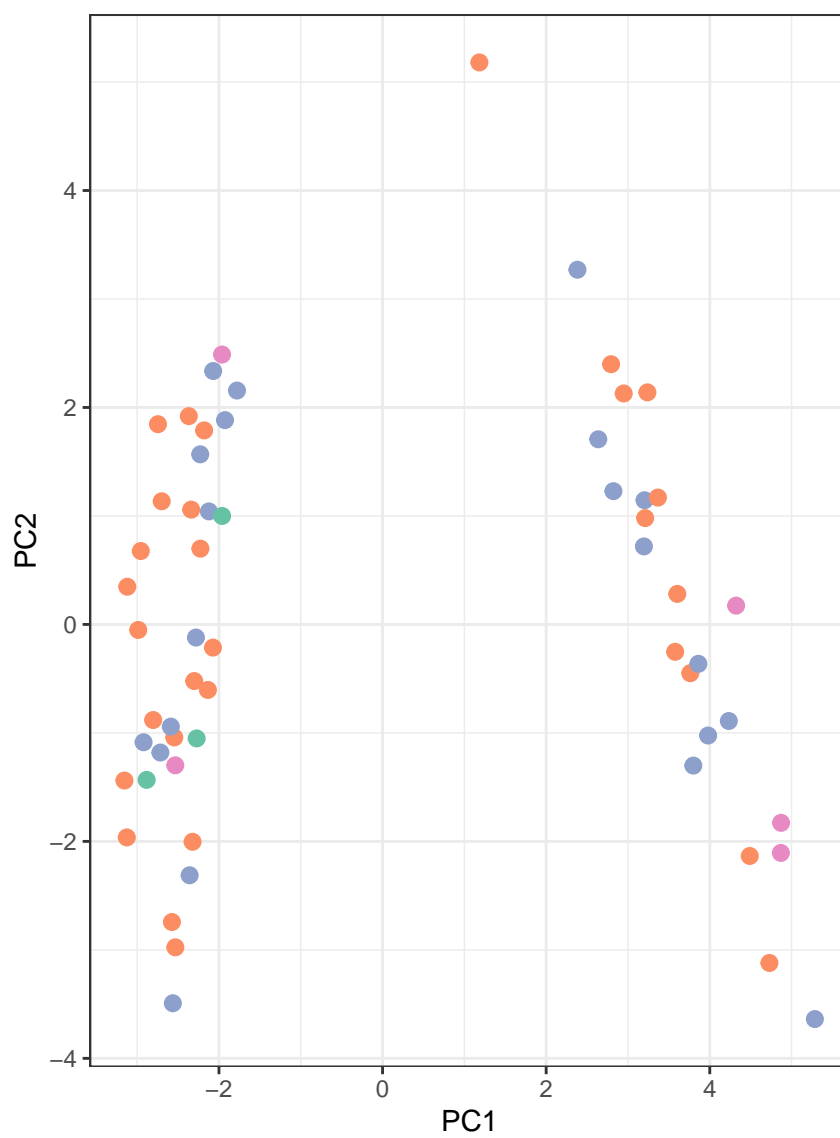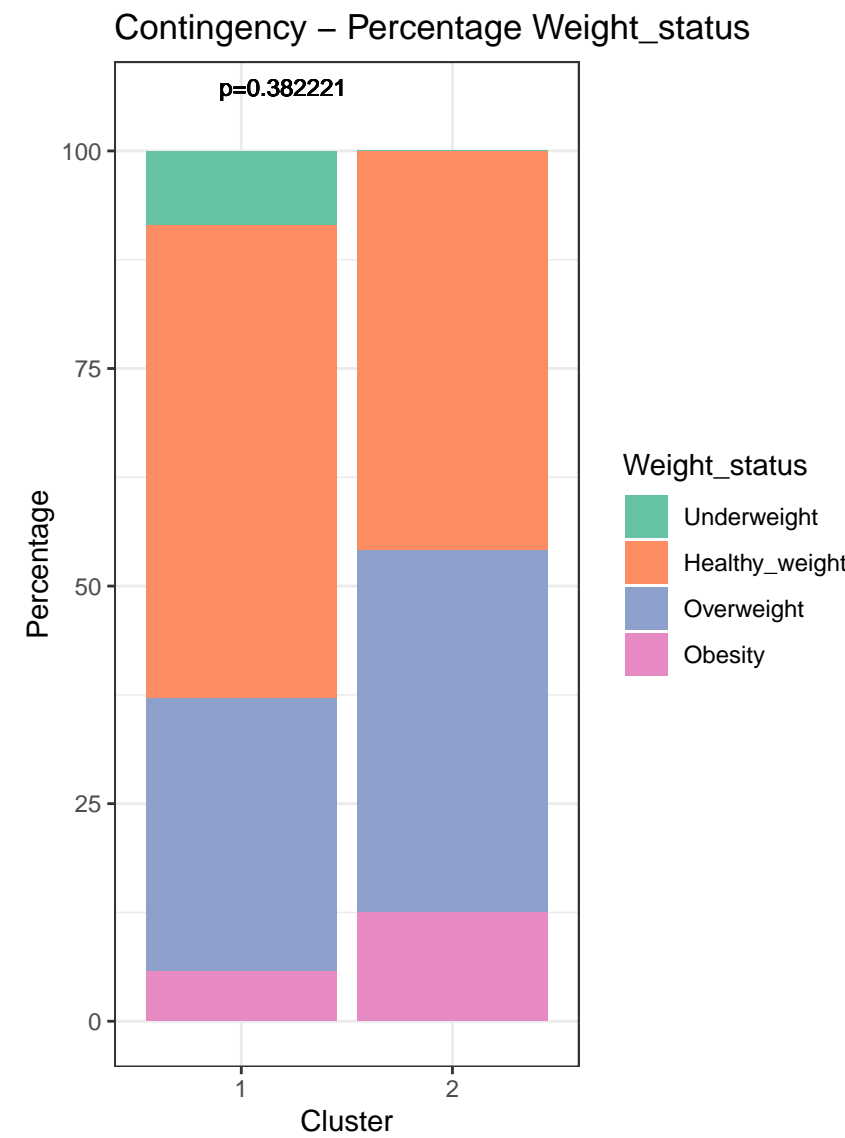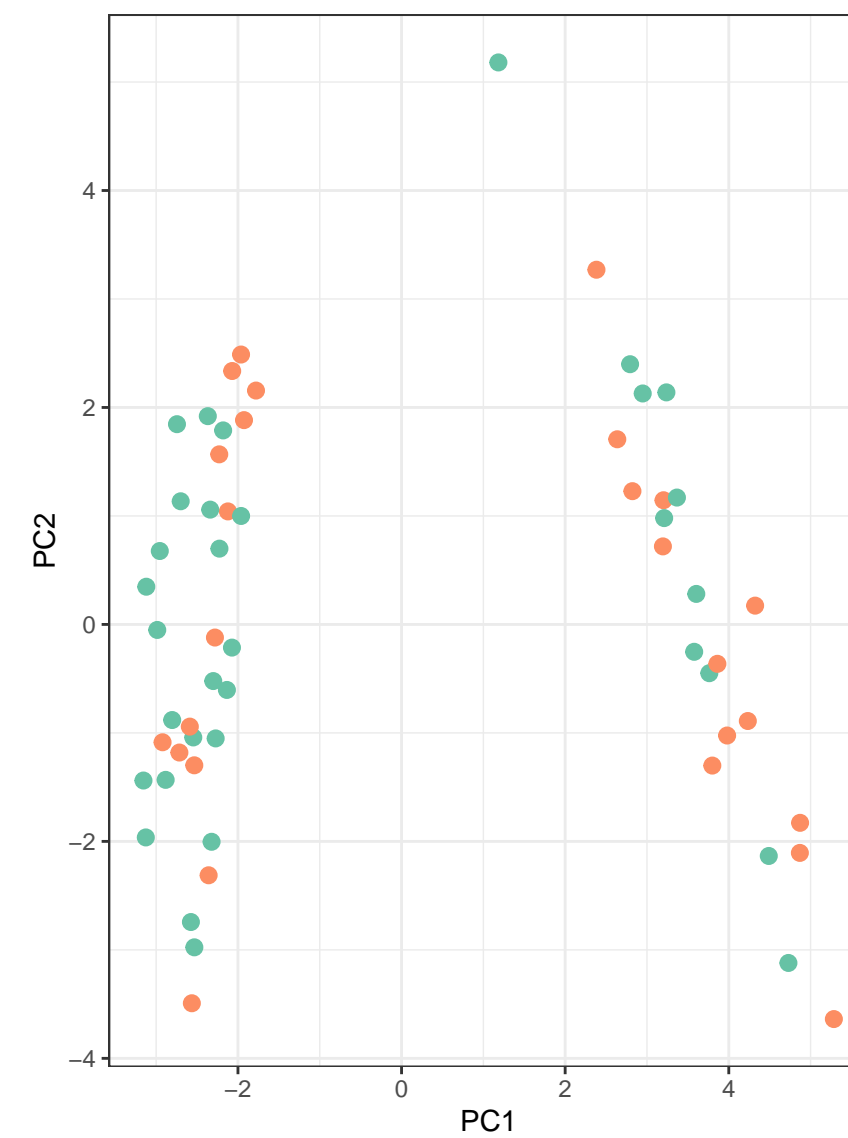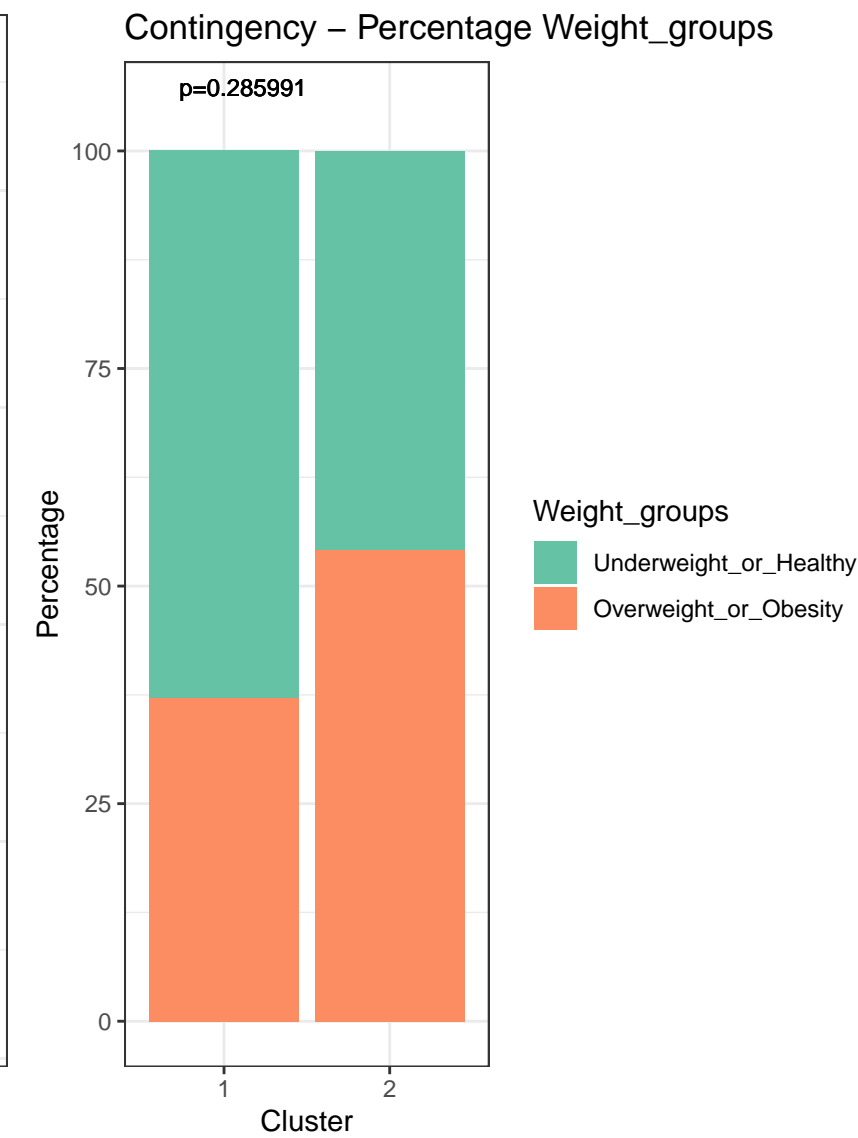

Supplement: Supplementary file 1 [file molecules-29-05169-s001.zip › molecules-3242400-supplementary/S15_disease_only_sphingolipids.pdf]

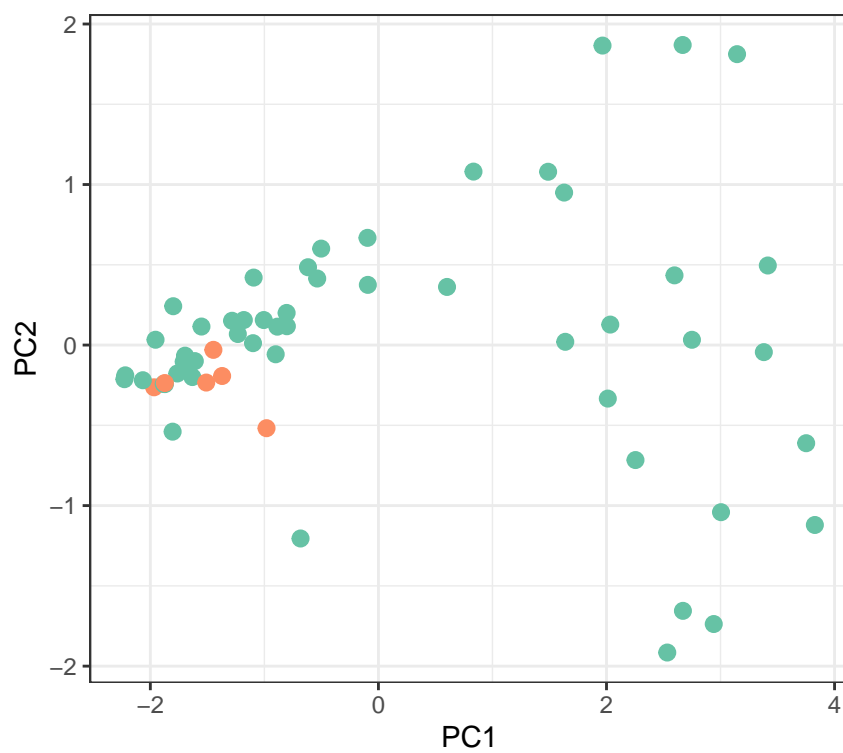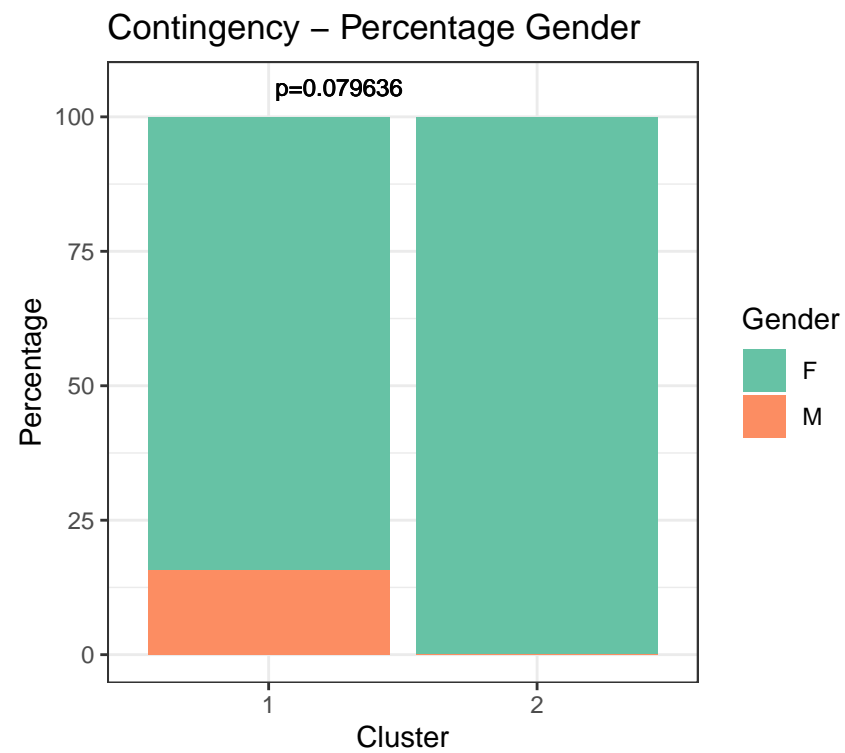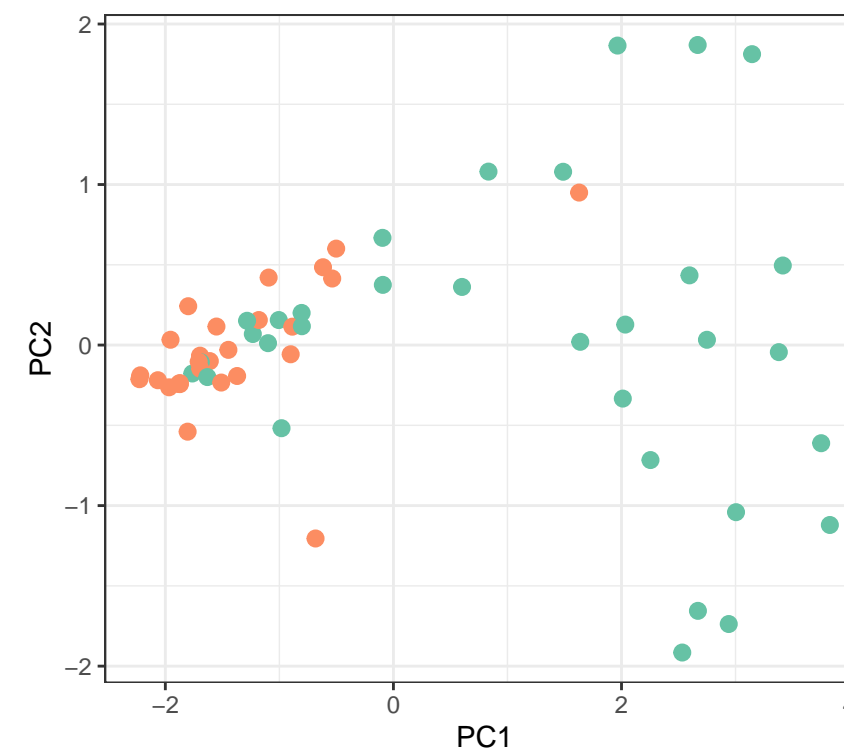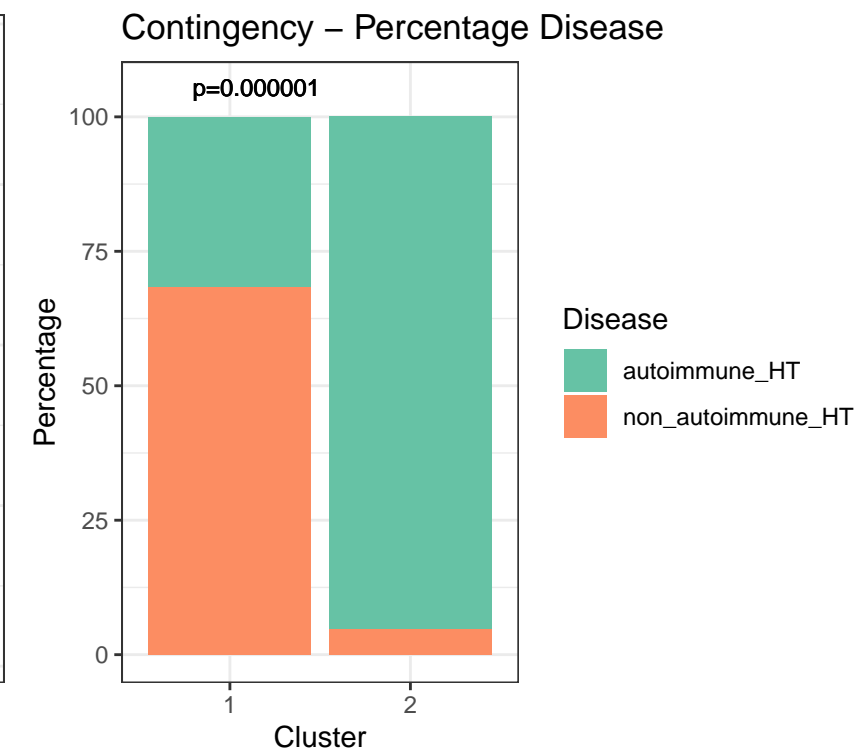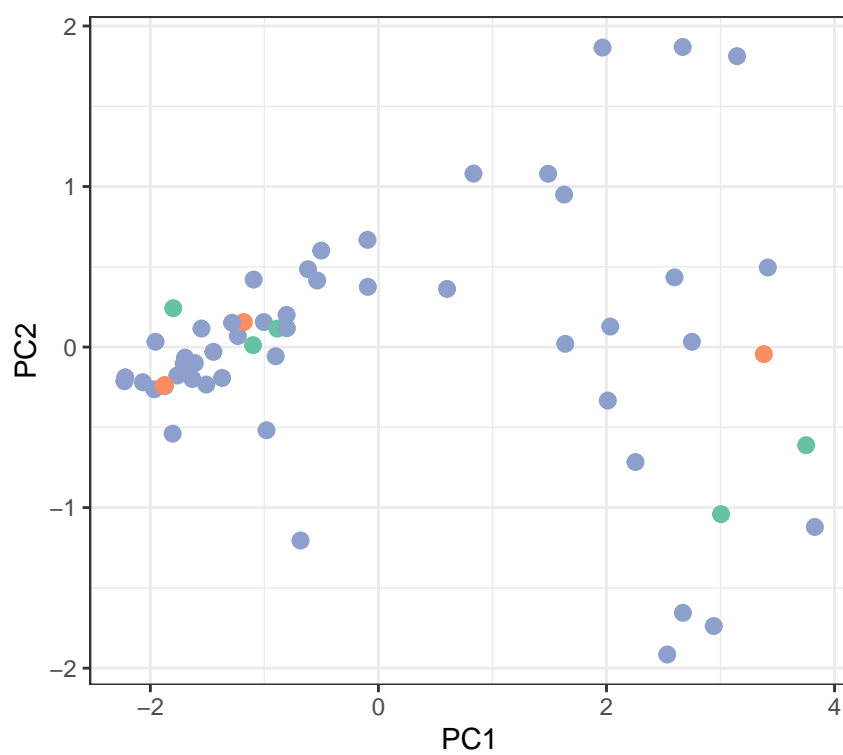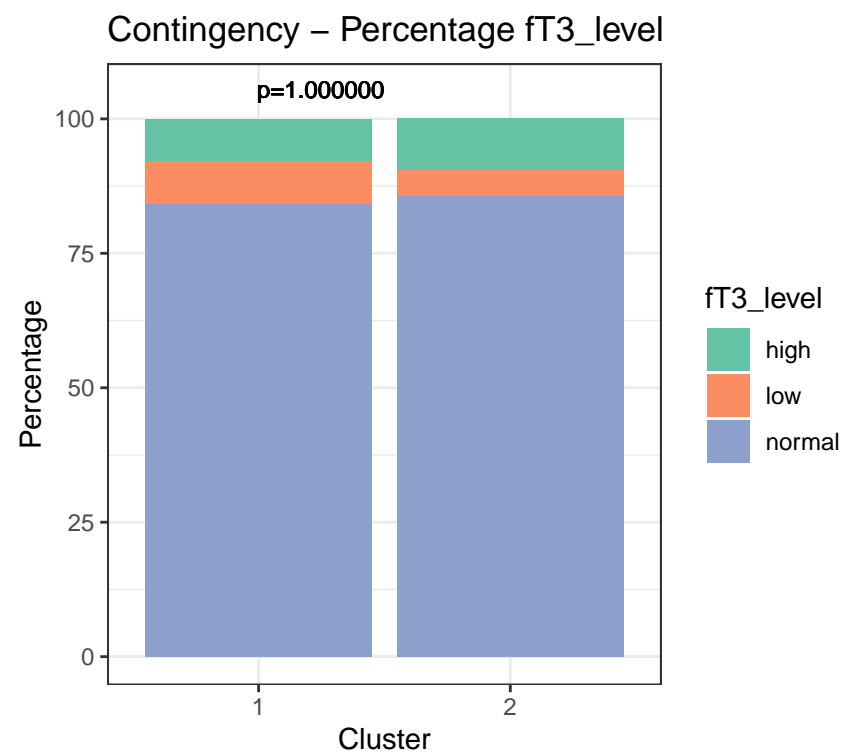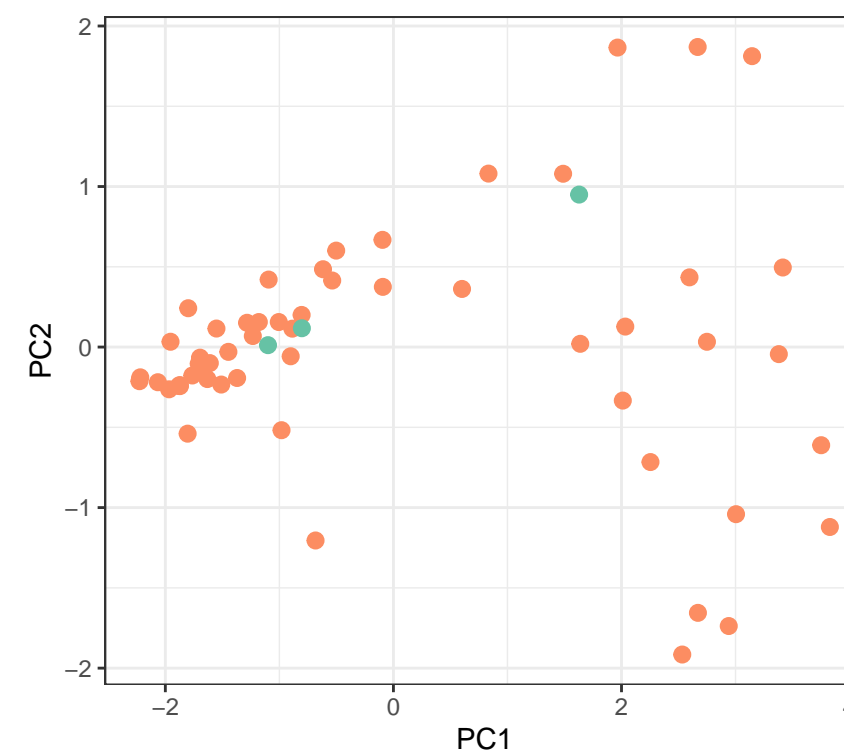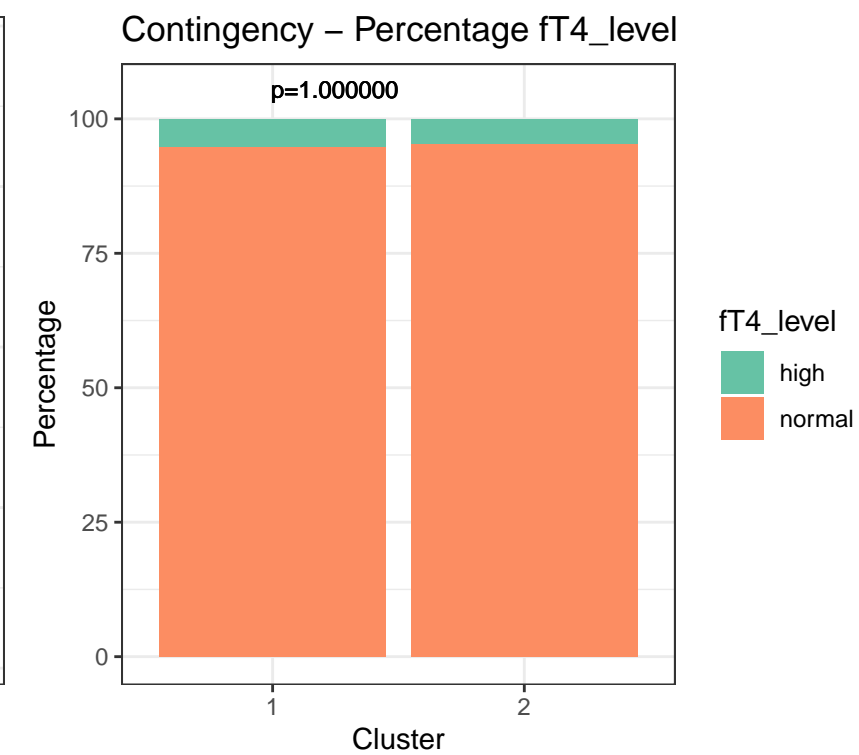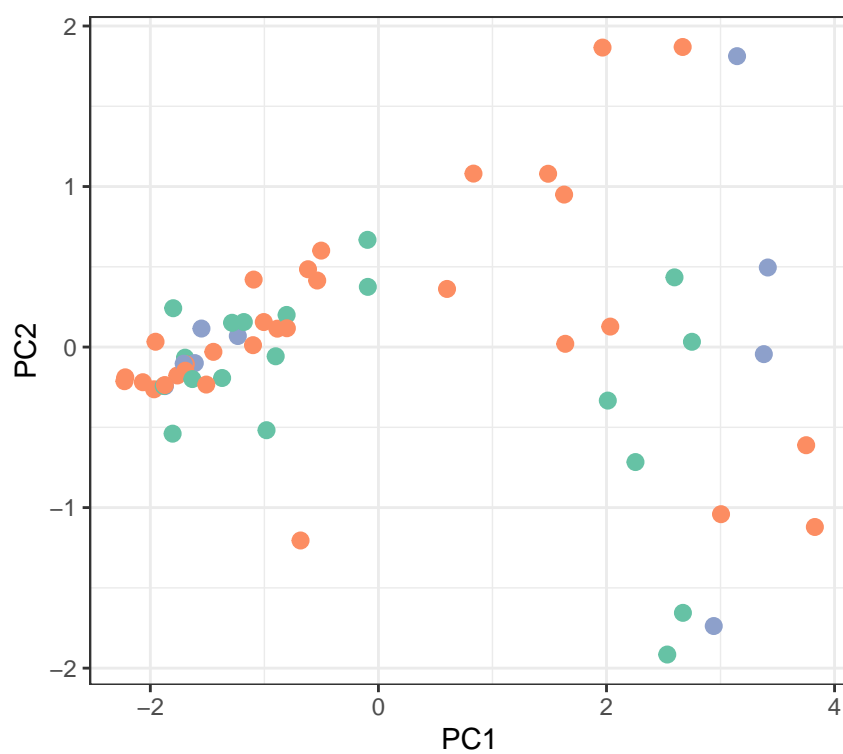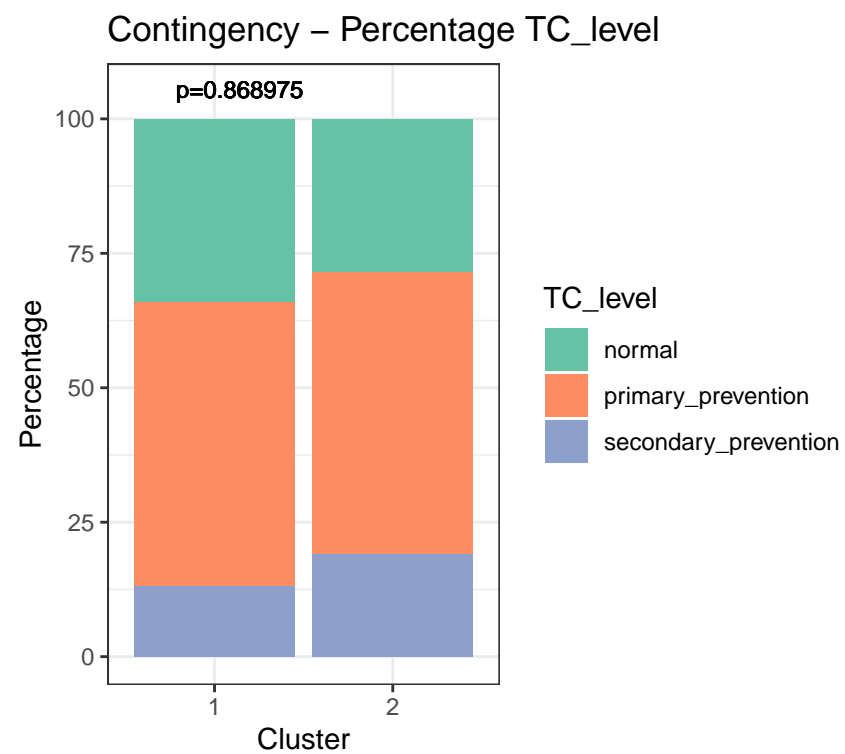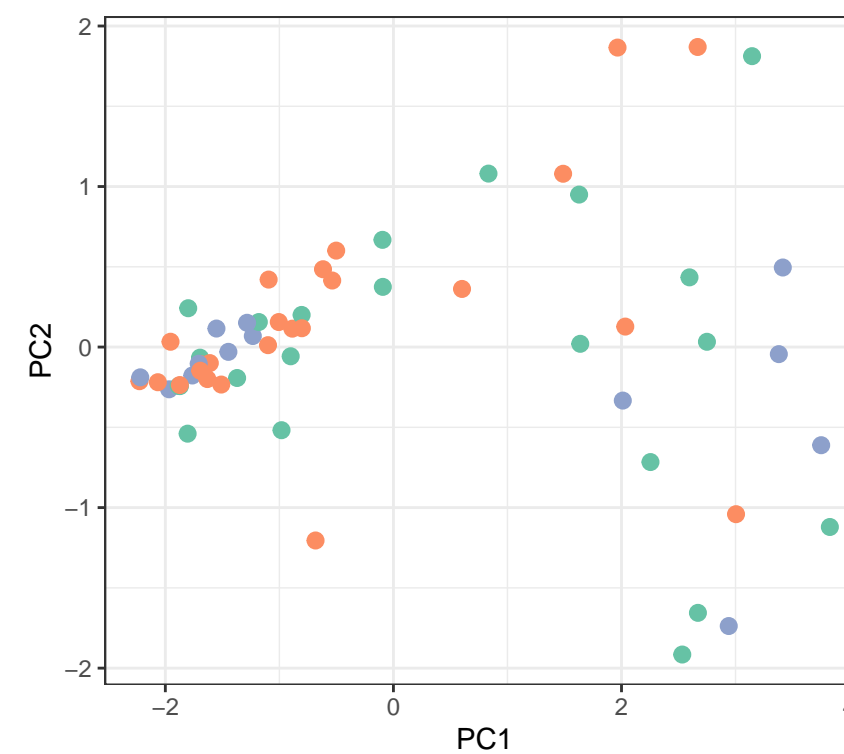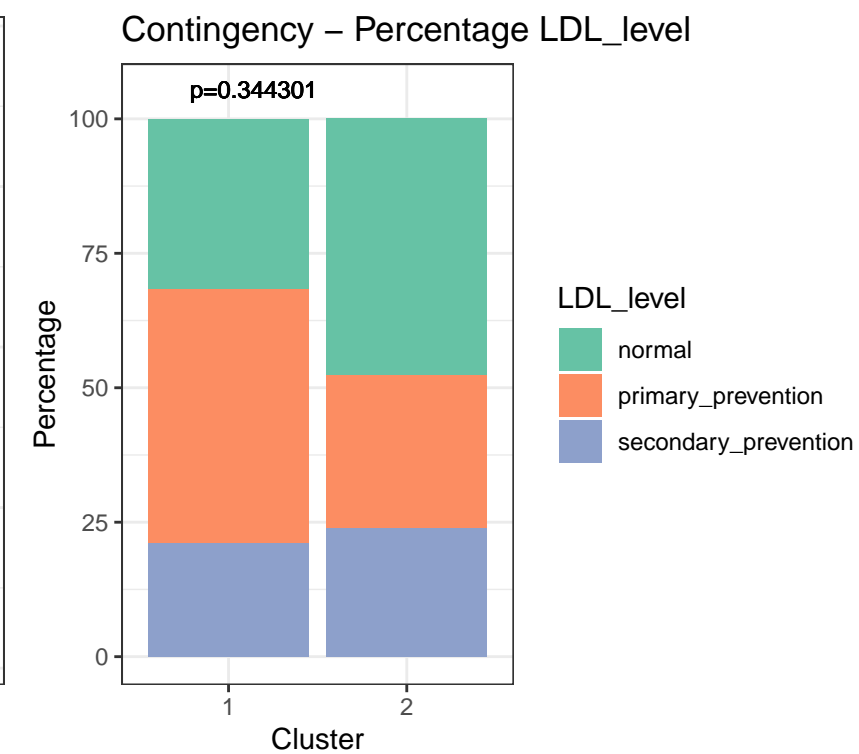

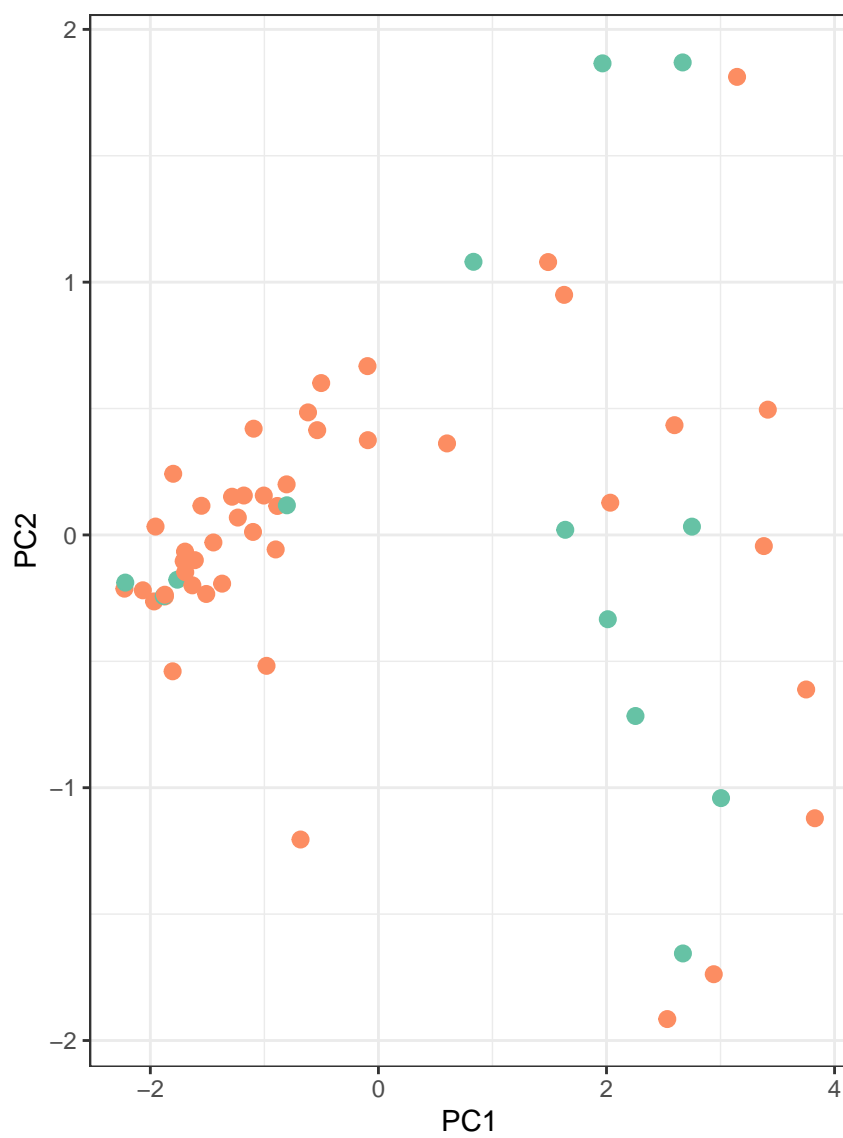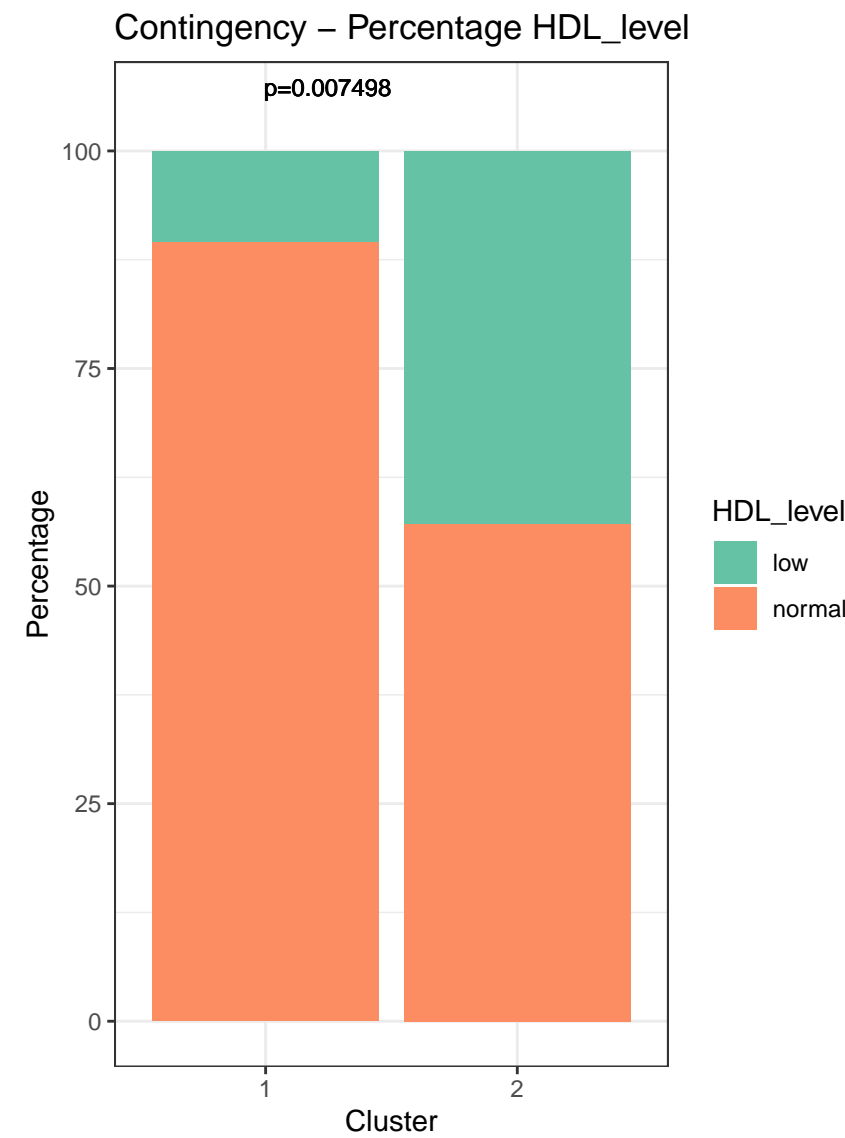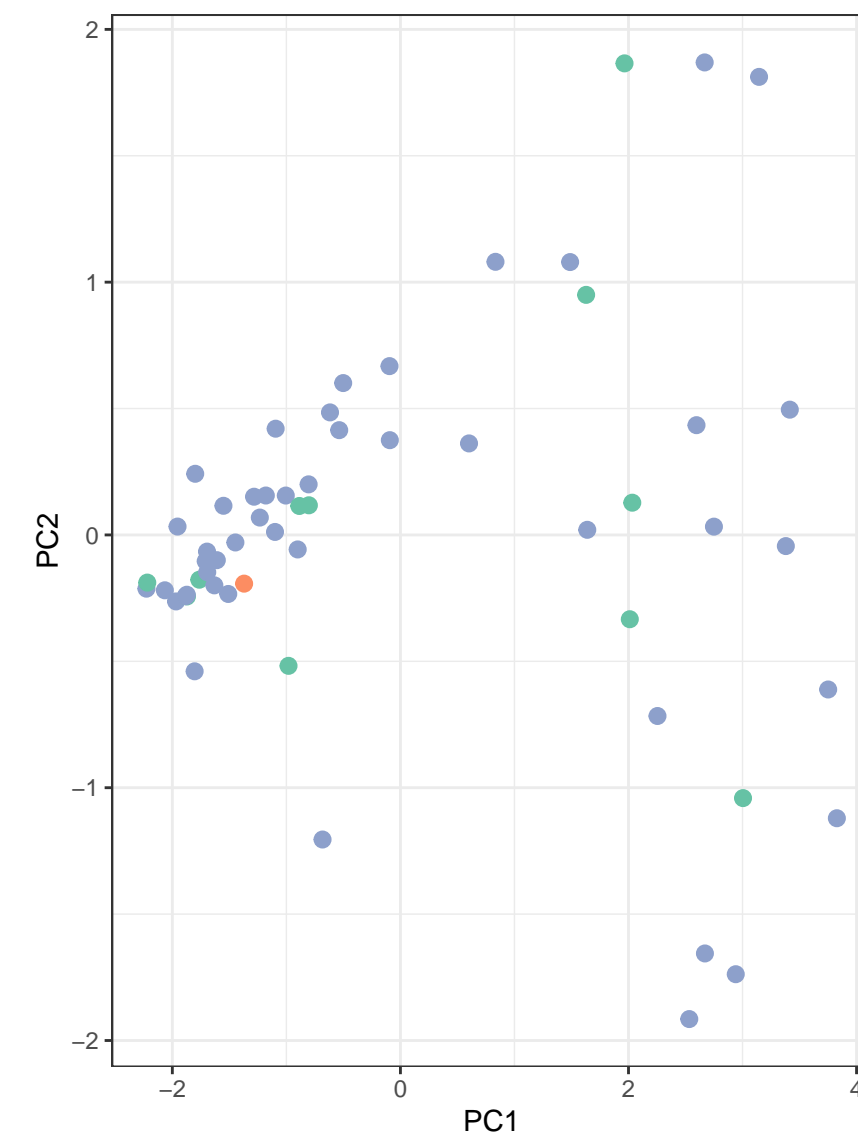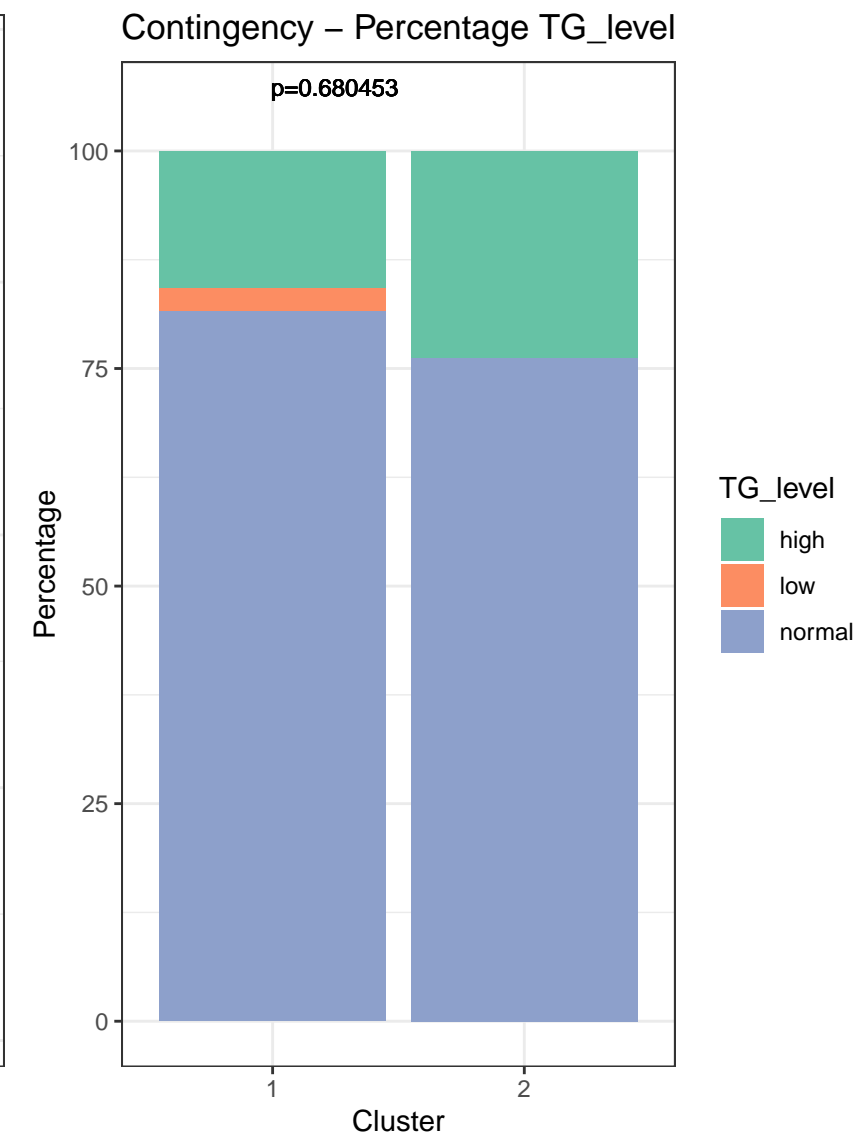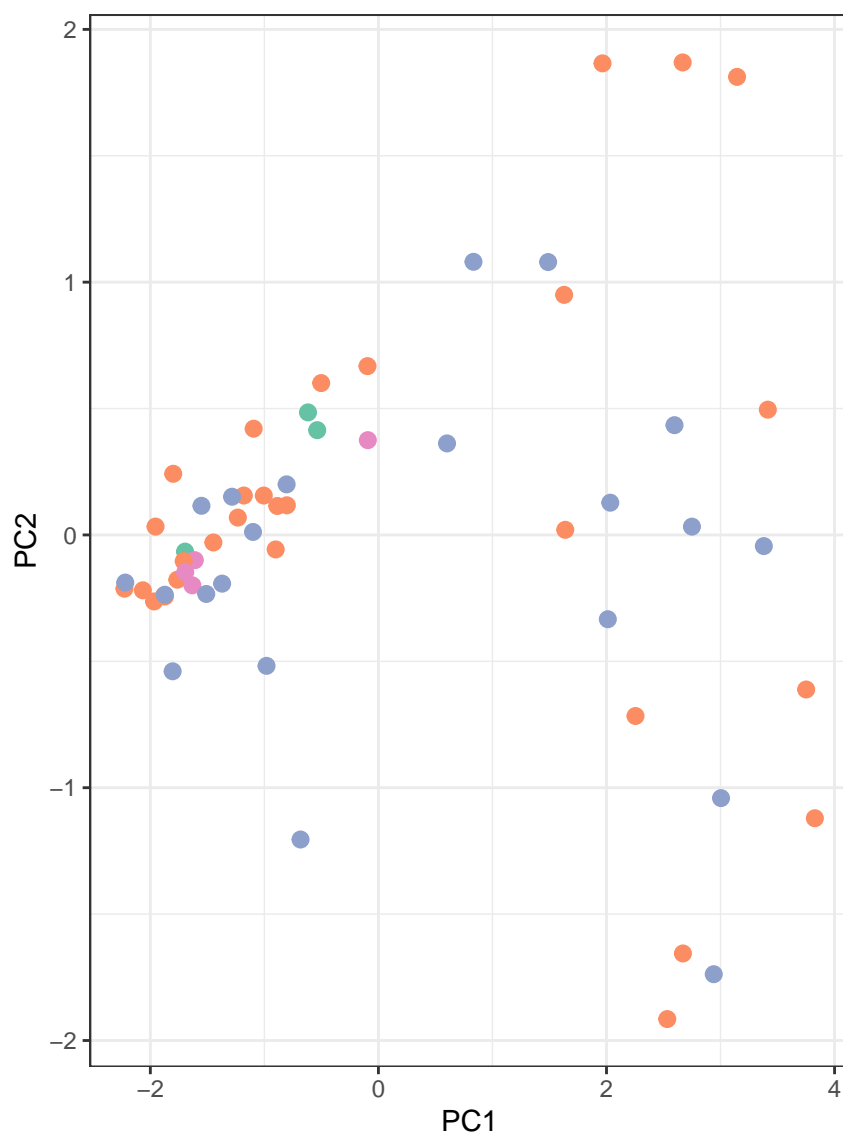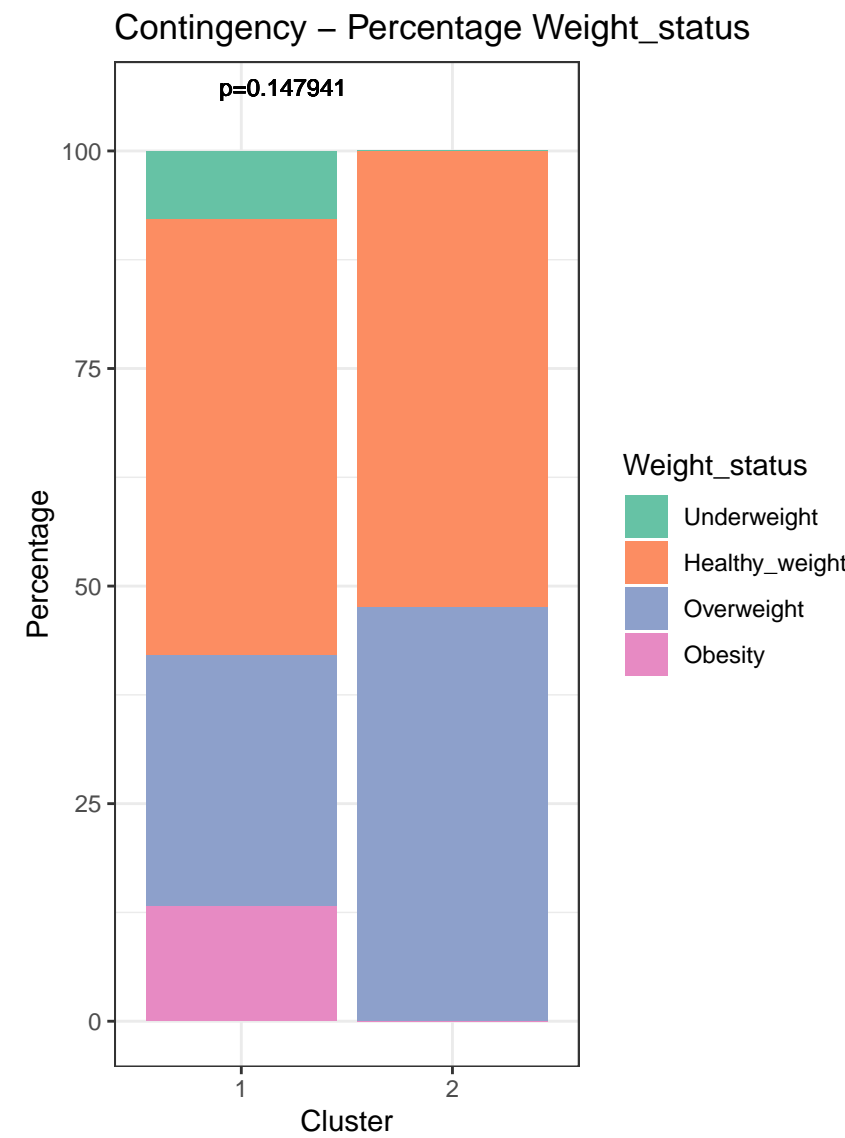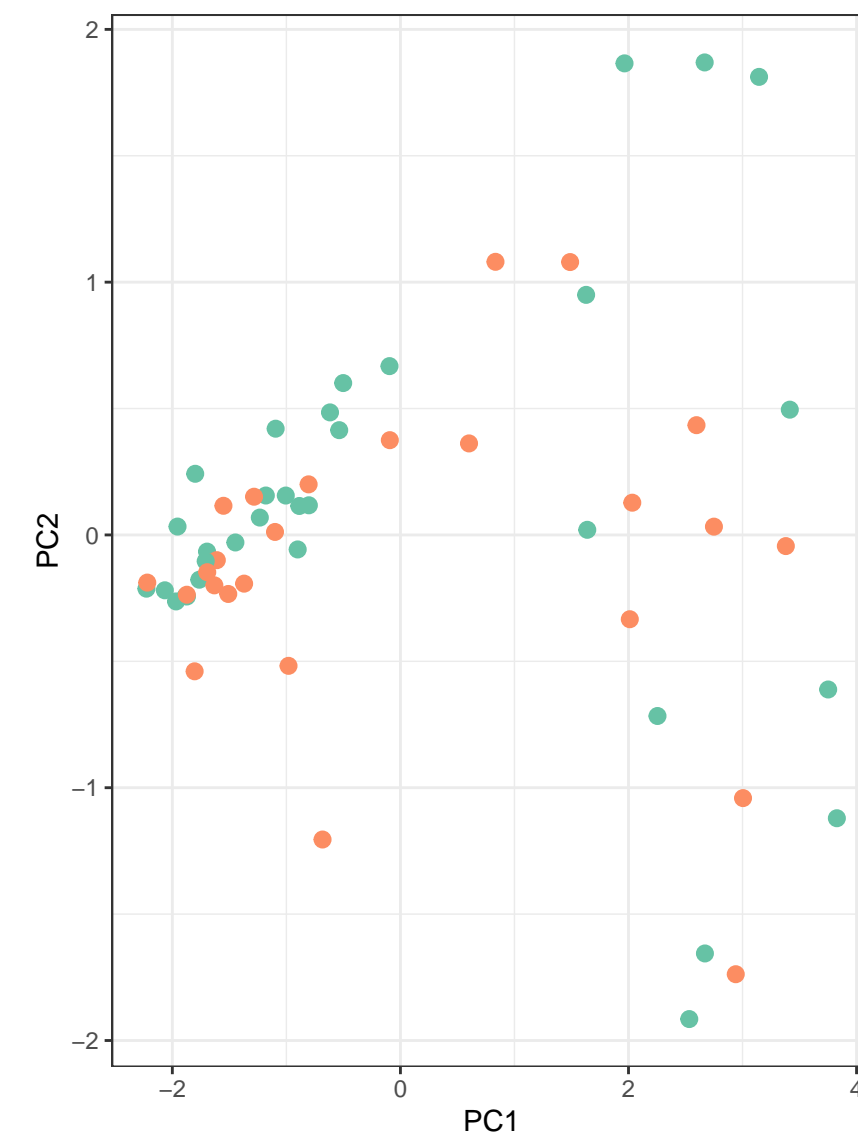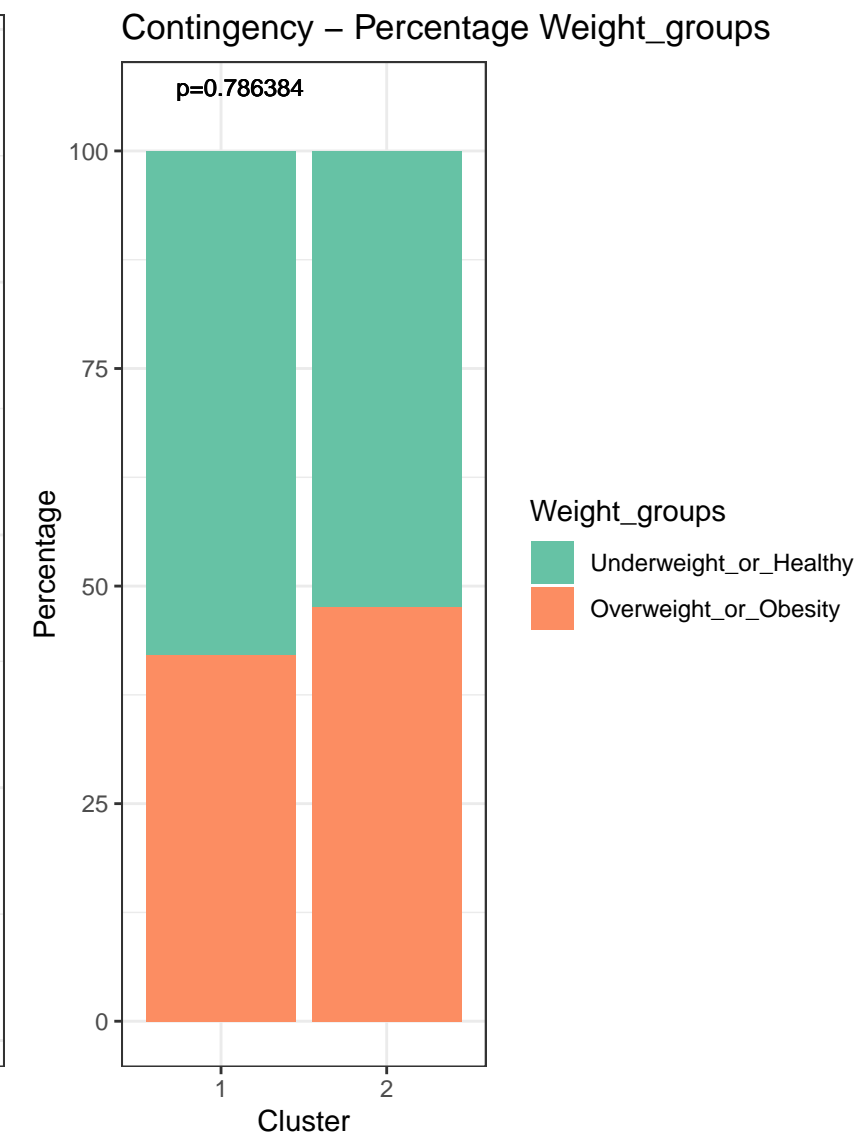

Supplement: Supplementary file 1 [file molecules-29-05169-s001.zip › molecules-3242400-supplementary/S16_disease_only_toxic_elements.pdf]

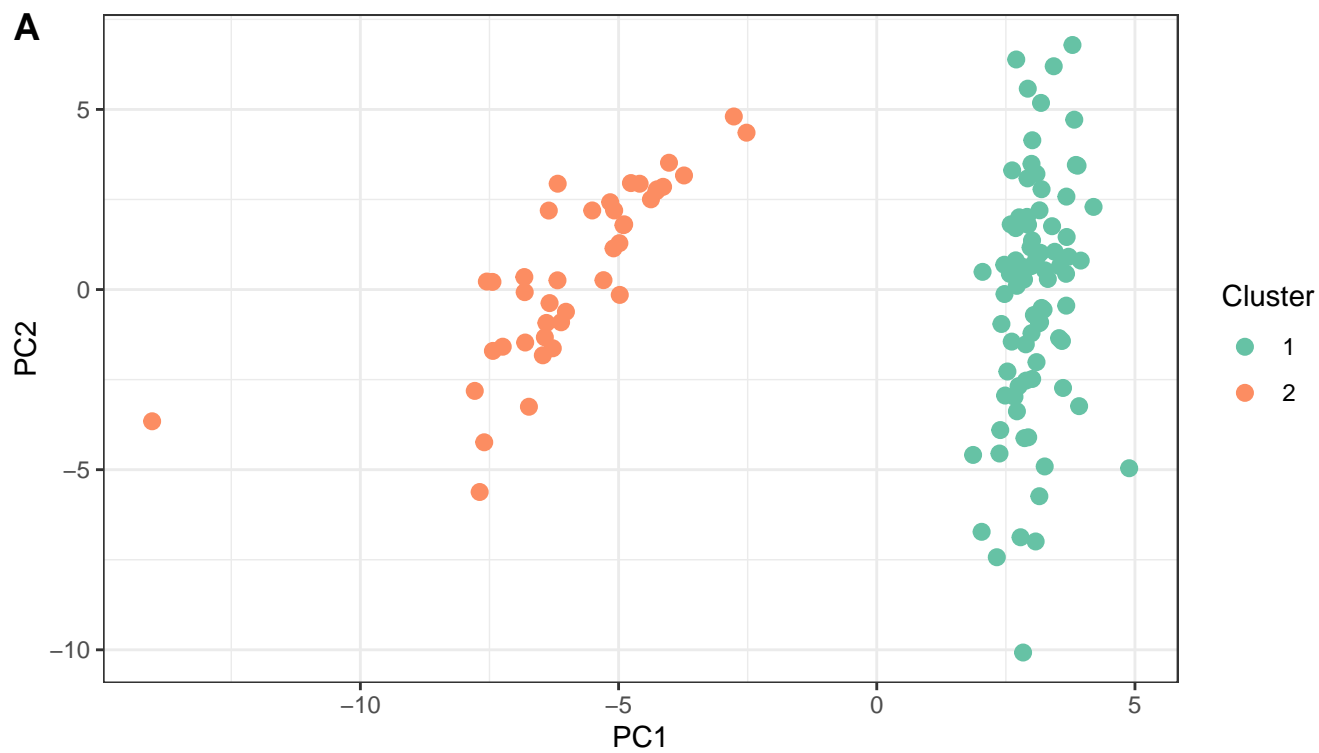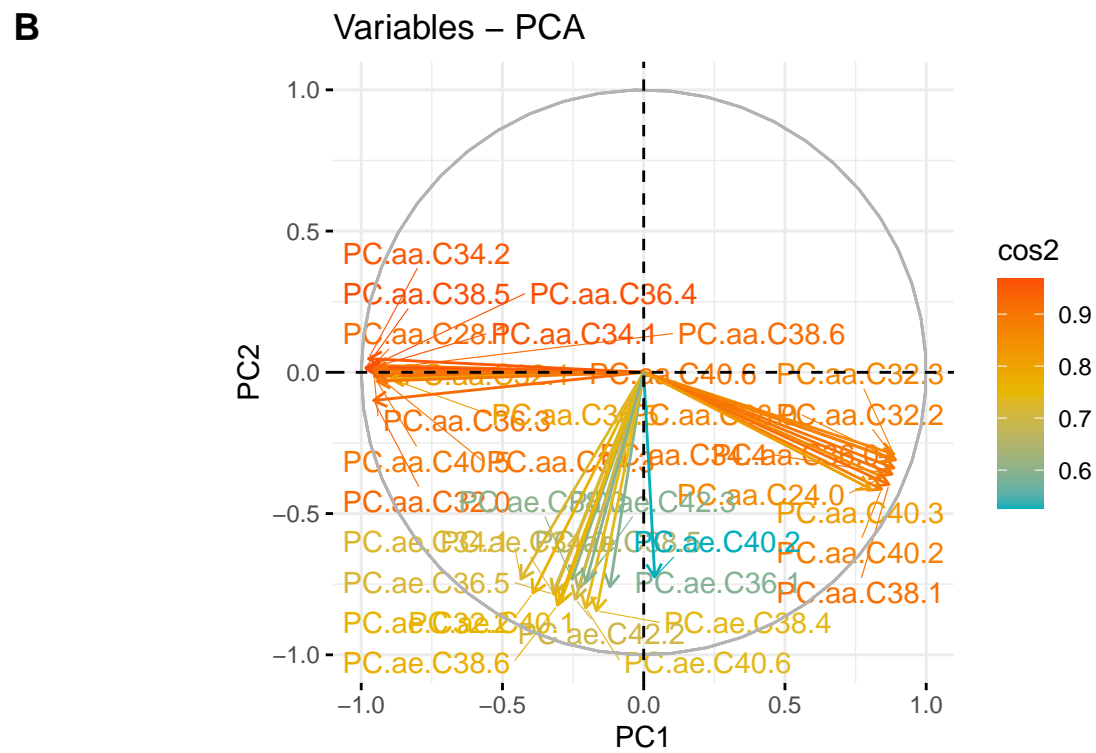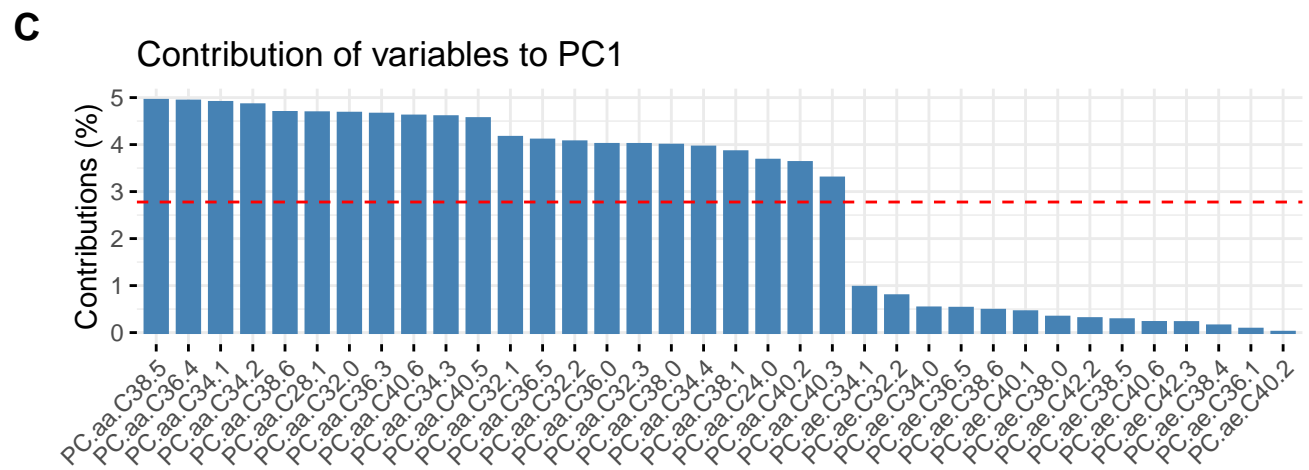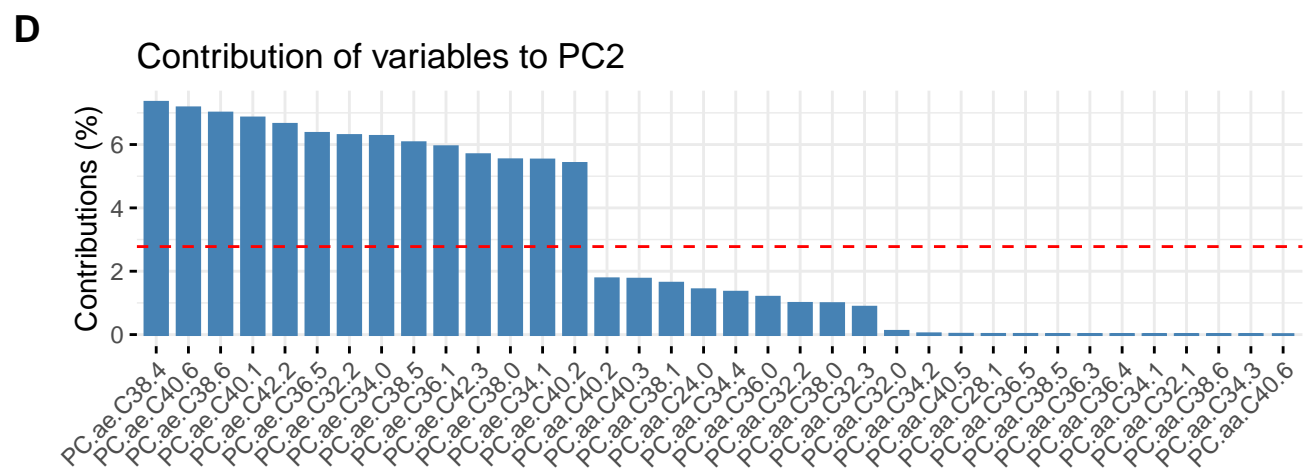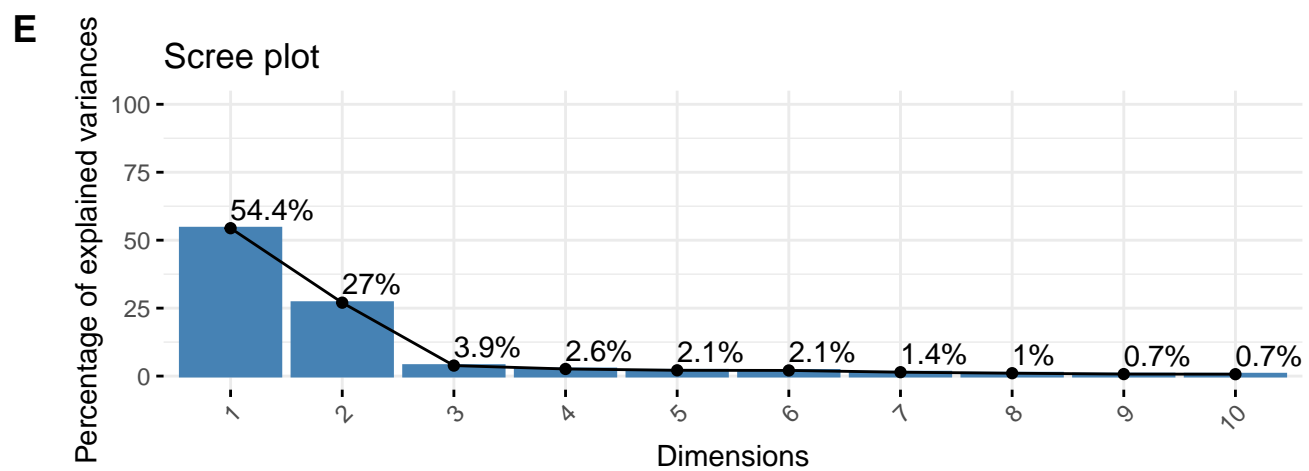

Supplement: Supplementary file 1 [file molecules-29-05169-s001.zip › molecules-3242400-supplementary/S2_filtered_biplot_eig_contrib.pdf]

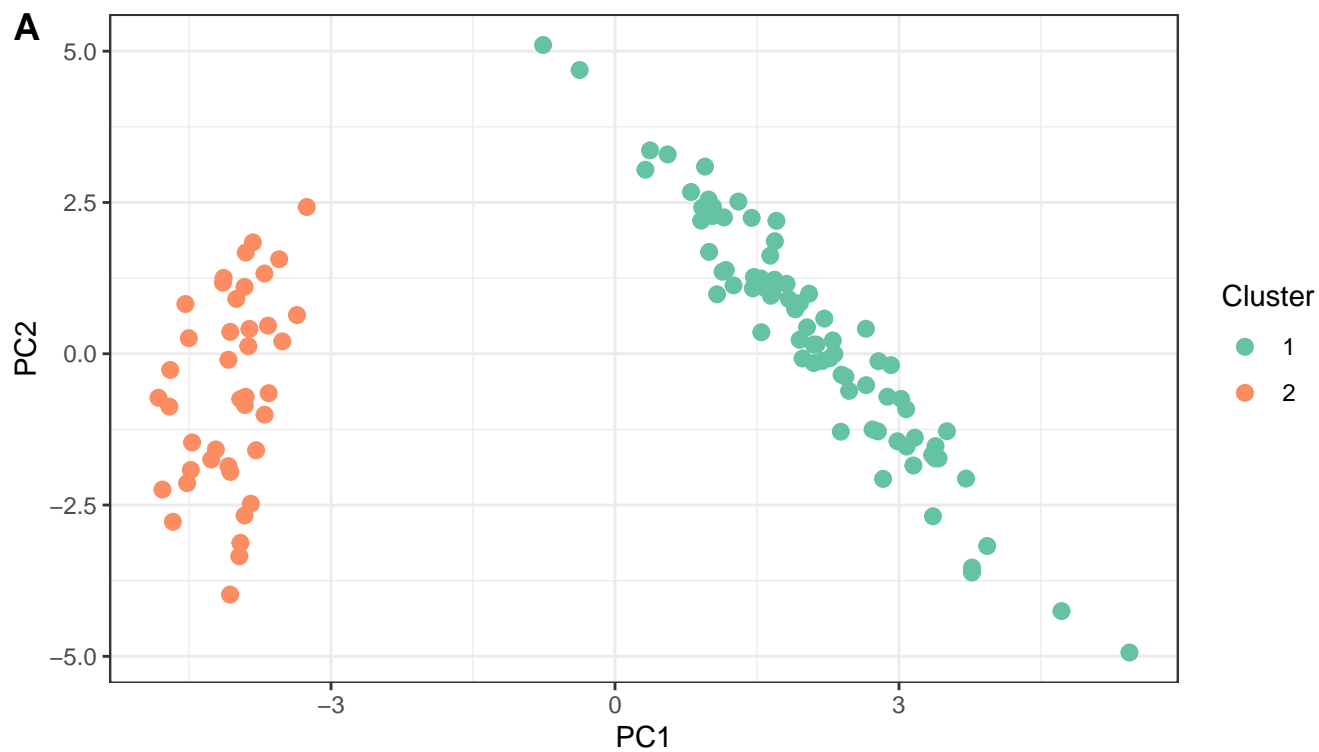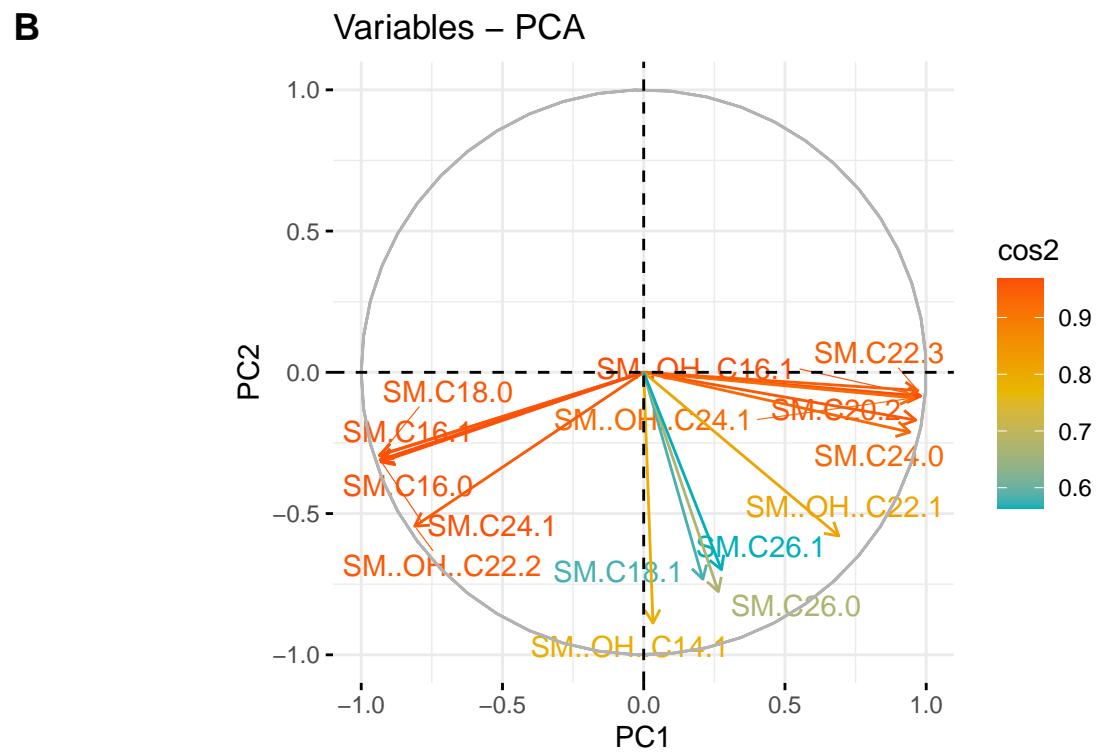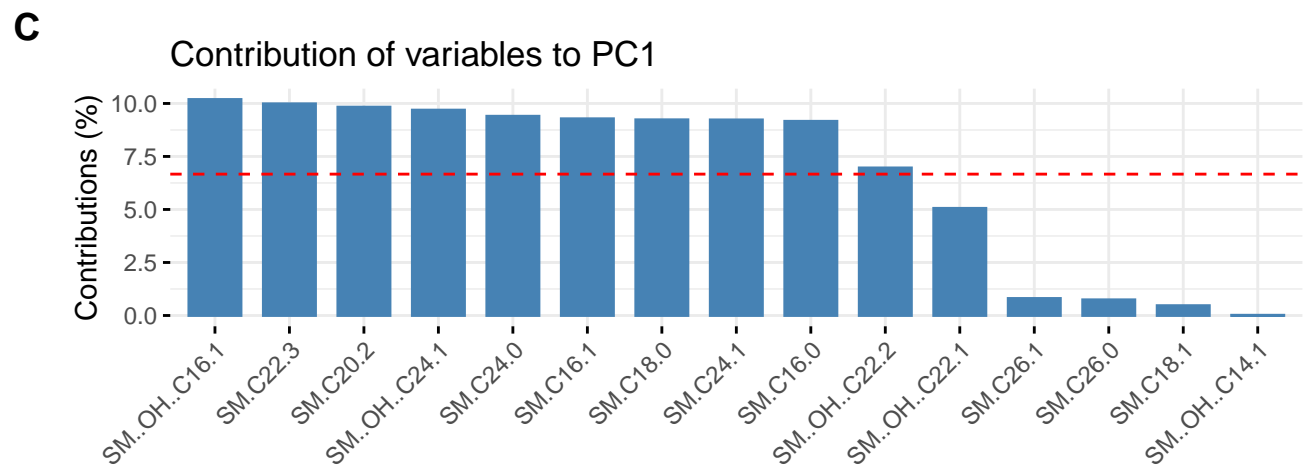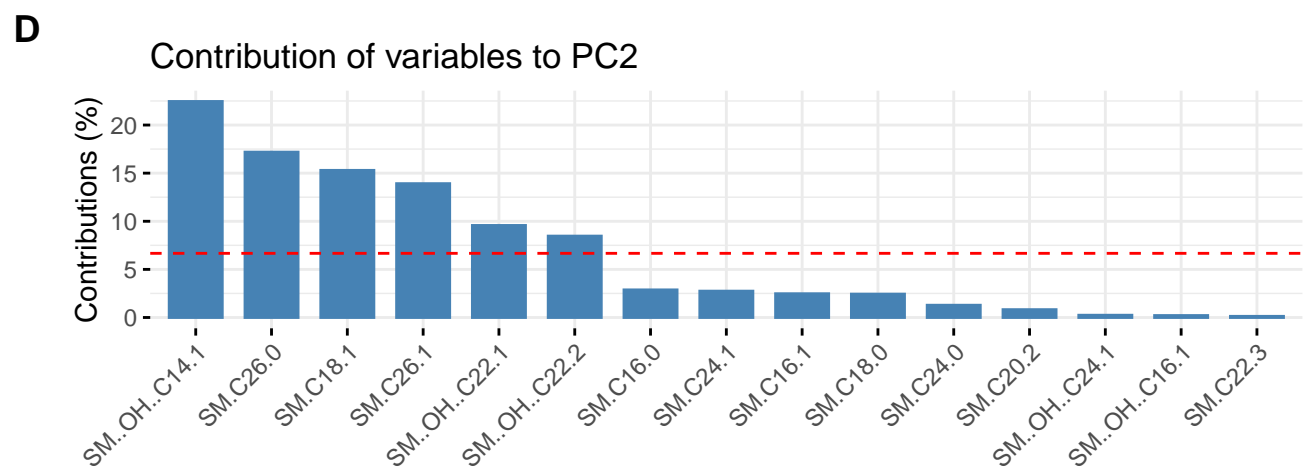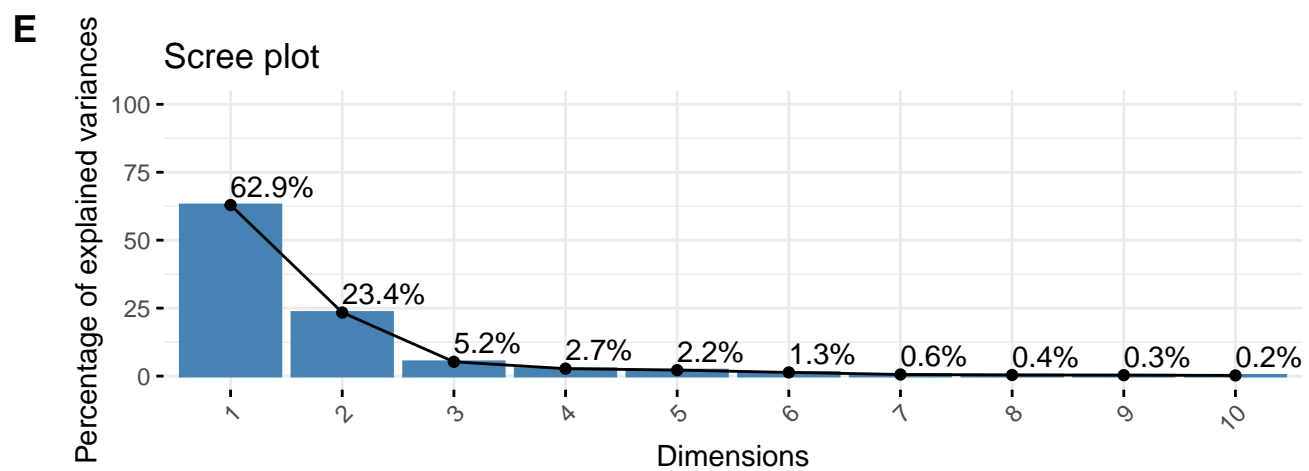

Supplement: Supplementary file 1 [file molecules-29-05169-s001.zip › molecules-3242400-supplementary/S3_filtered_biplot_eig_contrib.pdf]

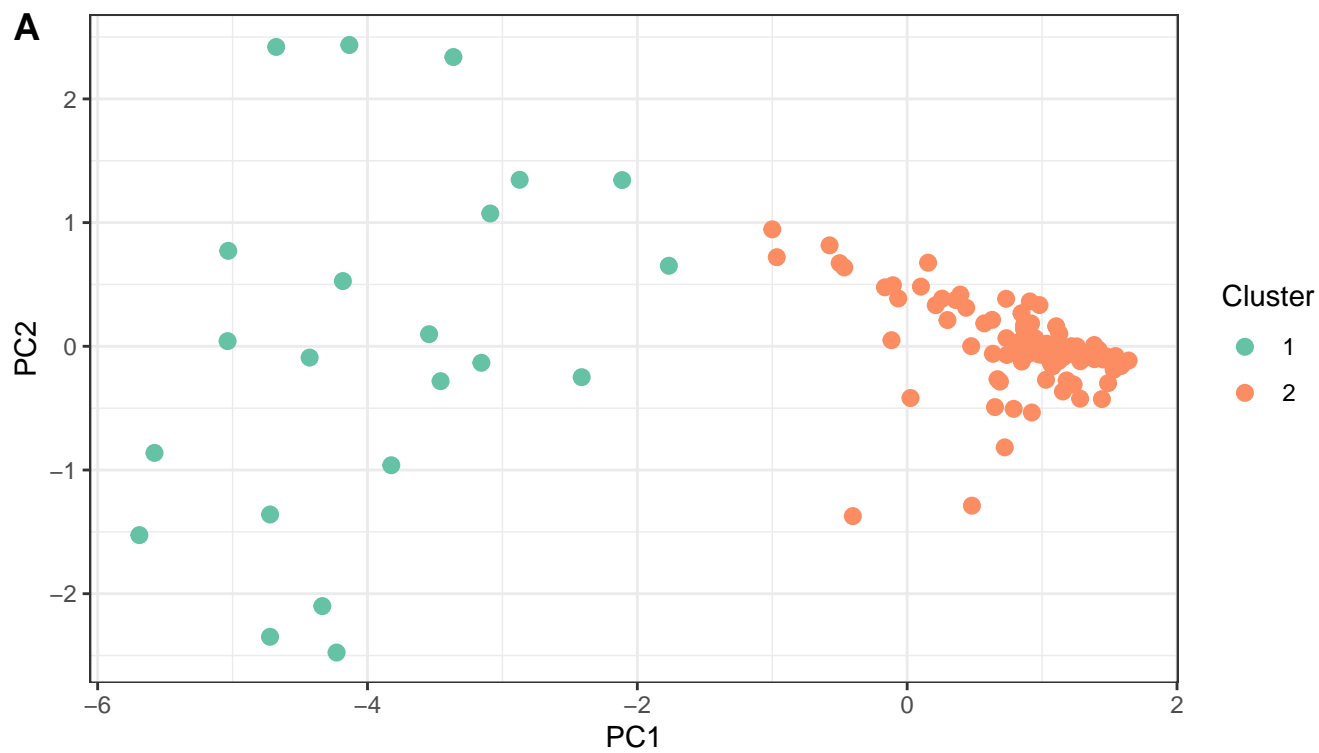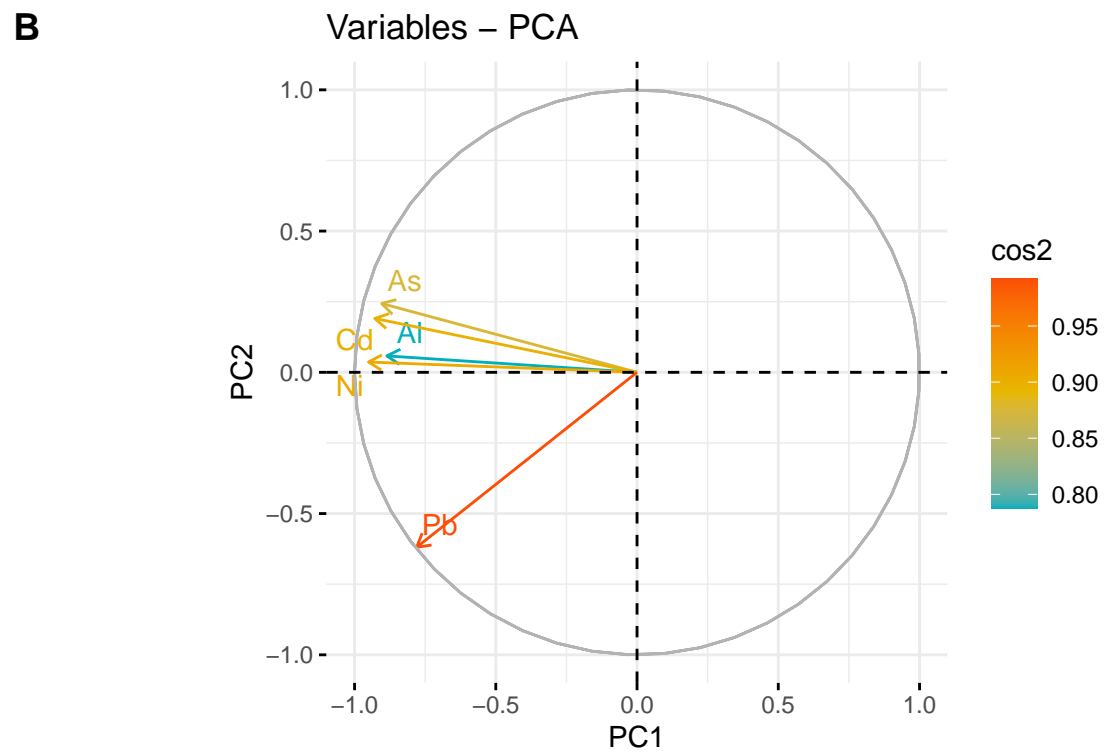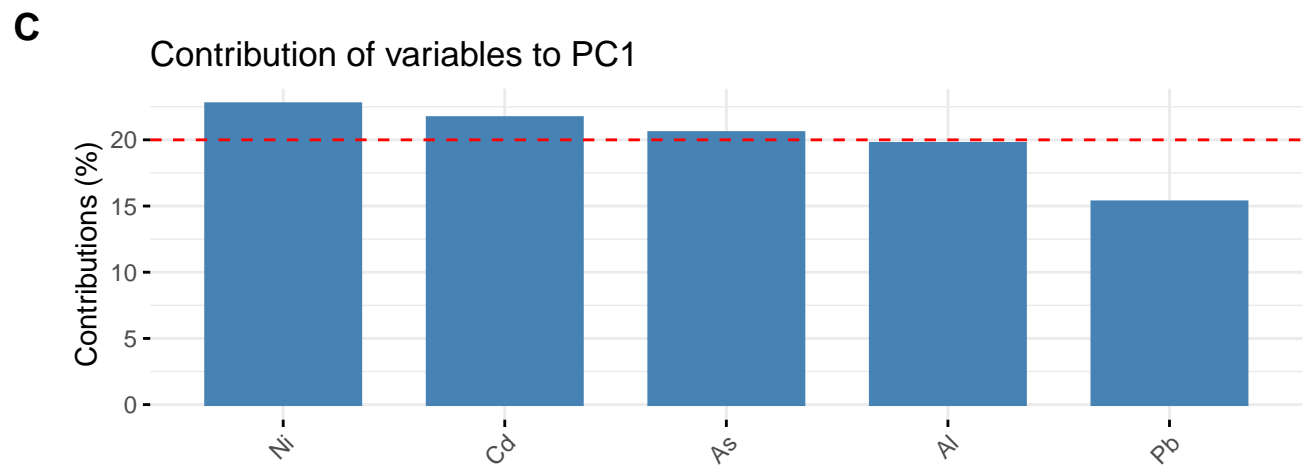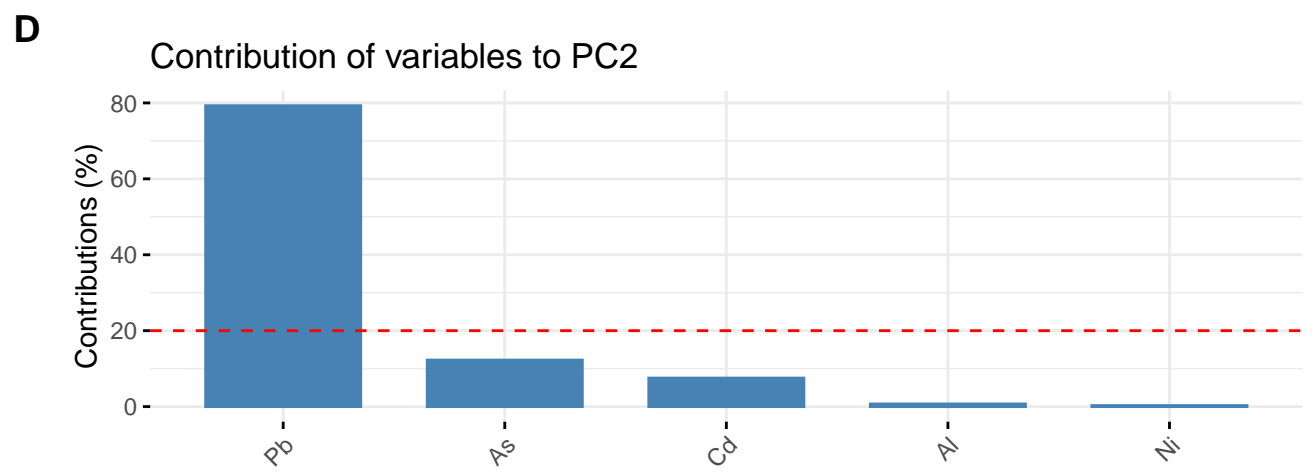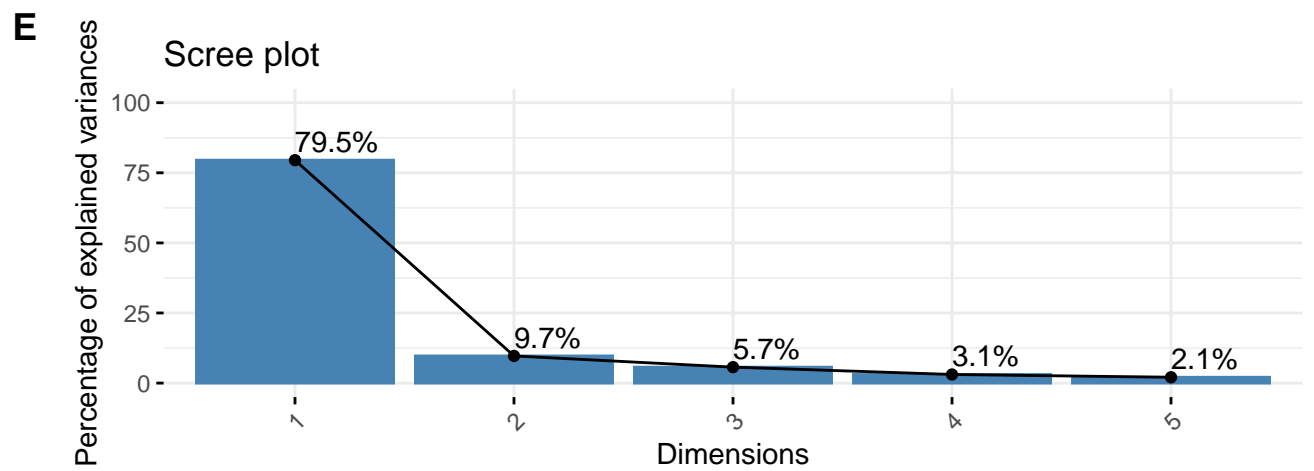

Supplement: Supplementary file 1 [file molecules-29-05169-s001.zip › molecules-3242400-supplementary/S4_filtered_biplot_eig_contrib.pdf]

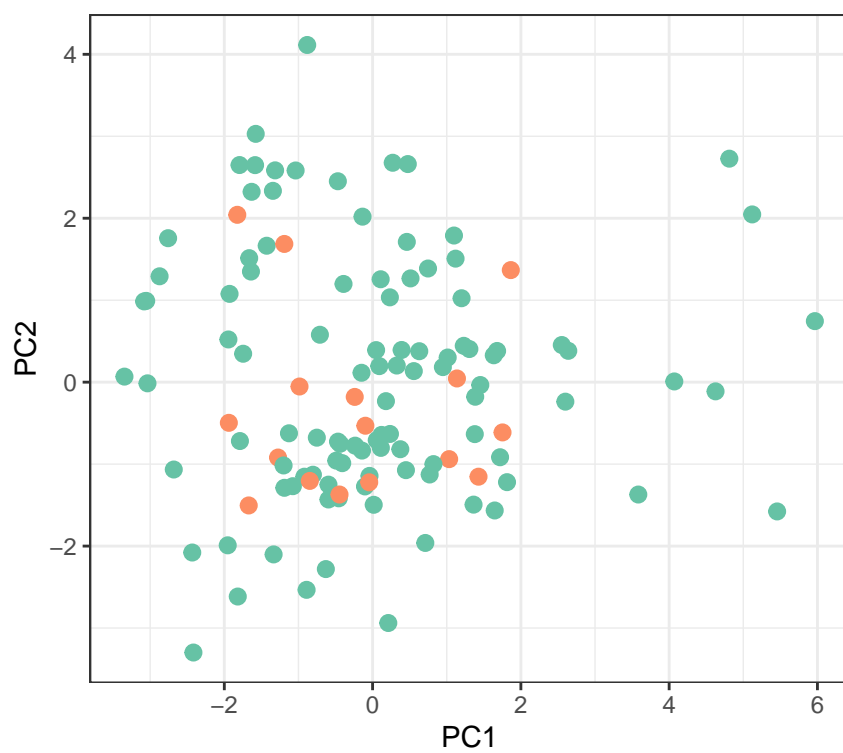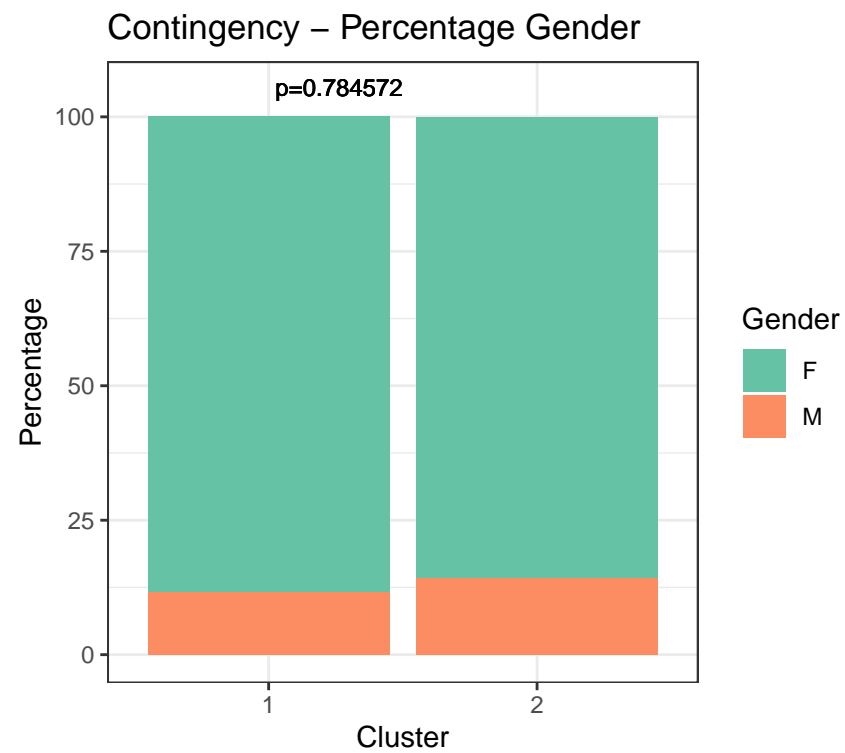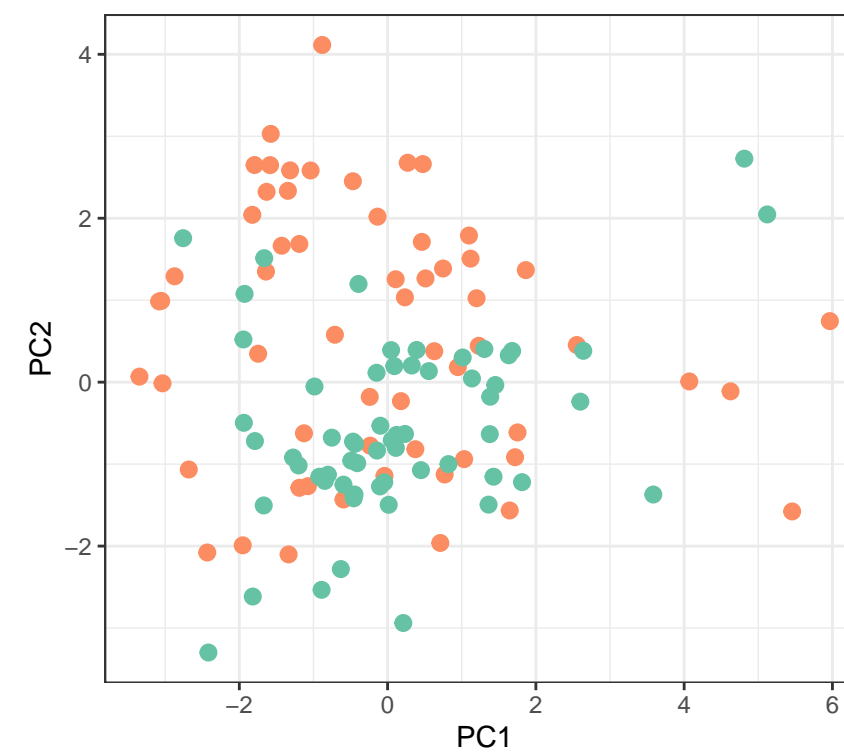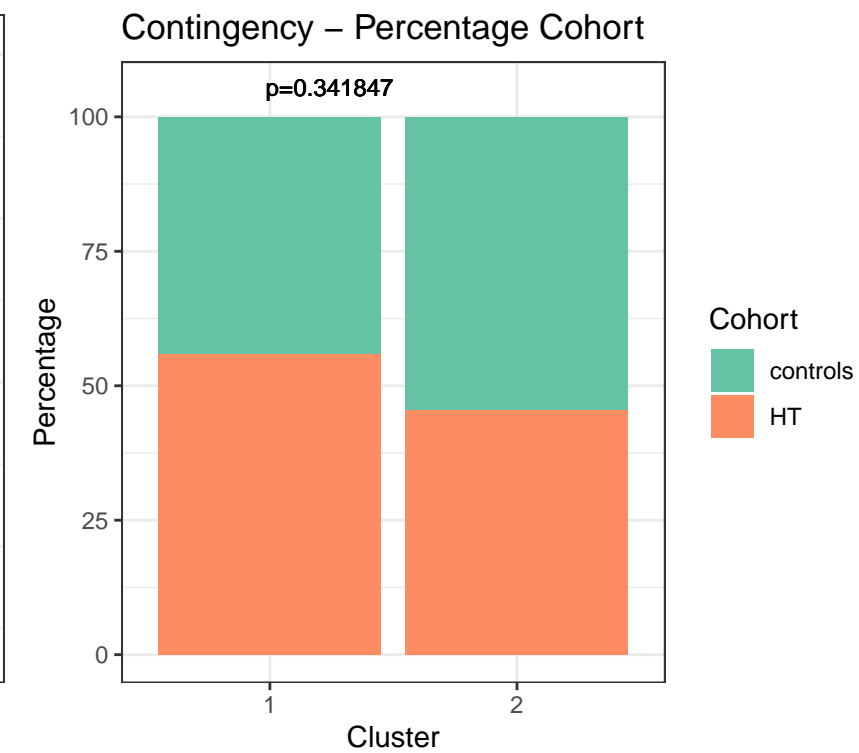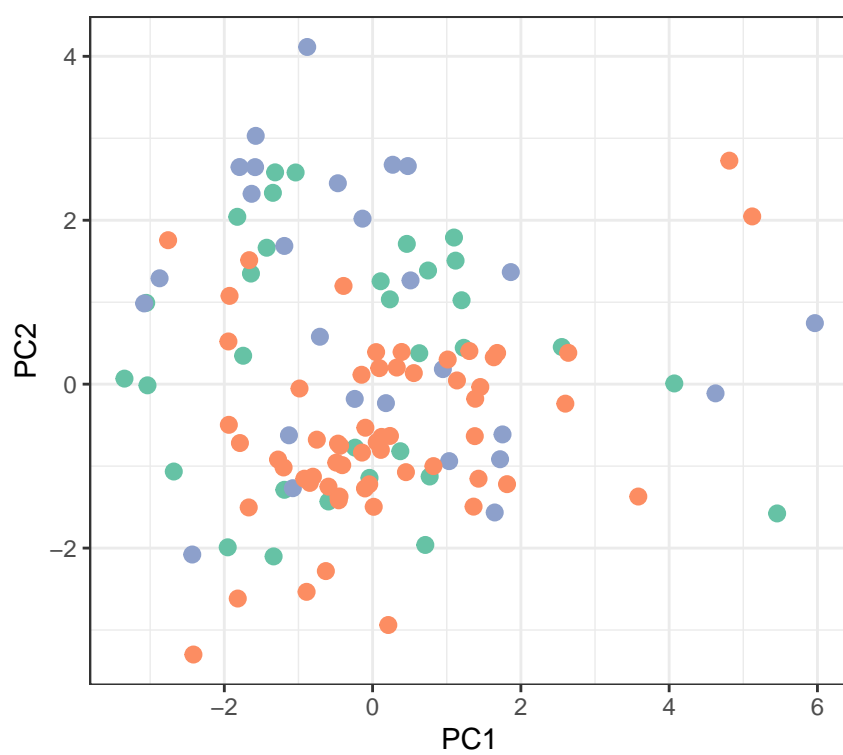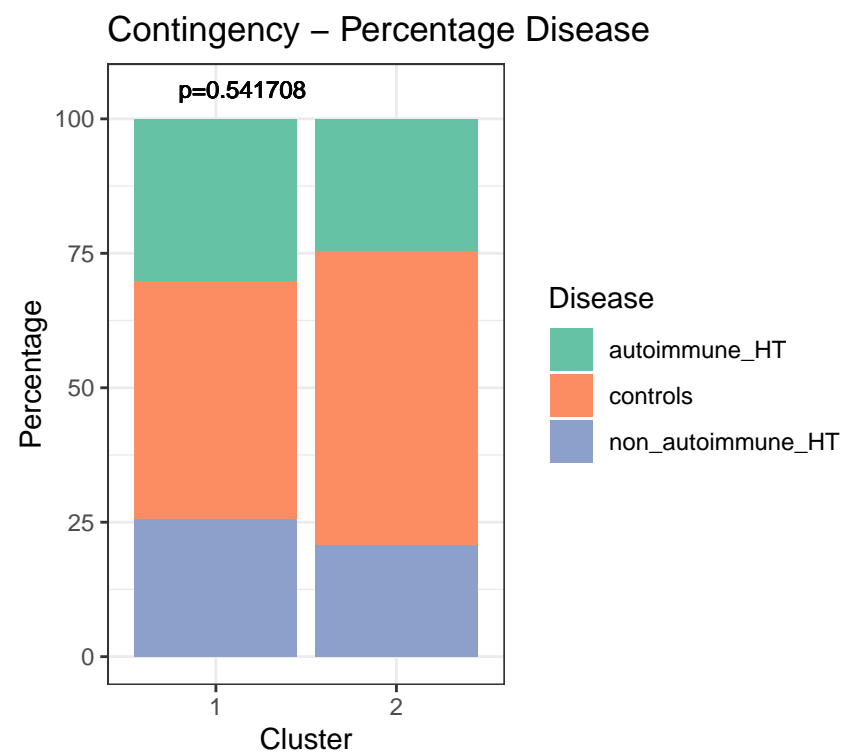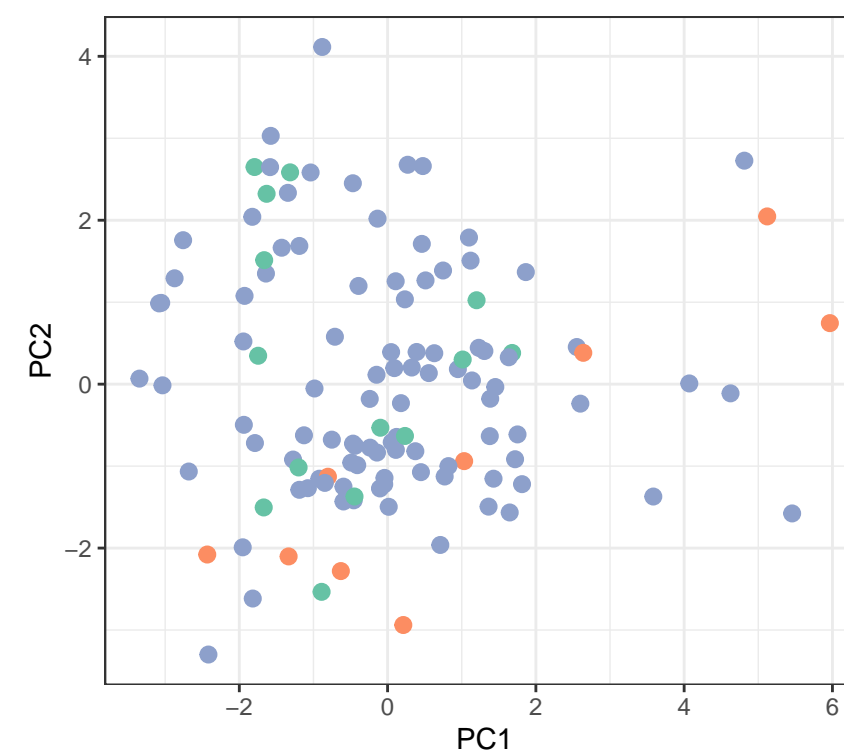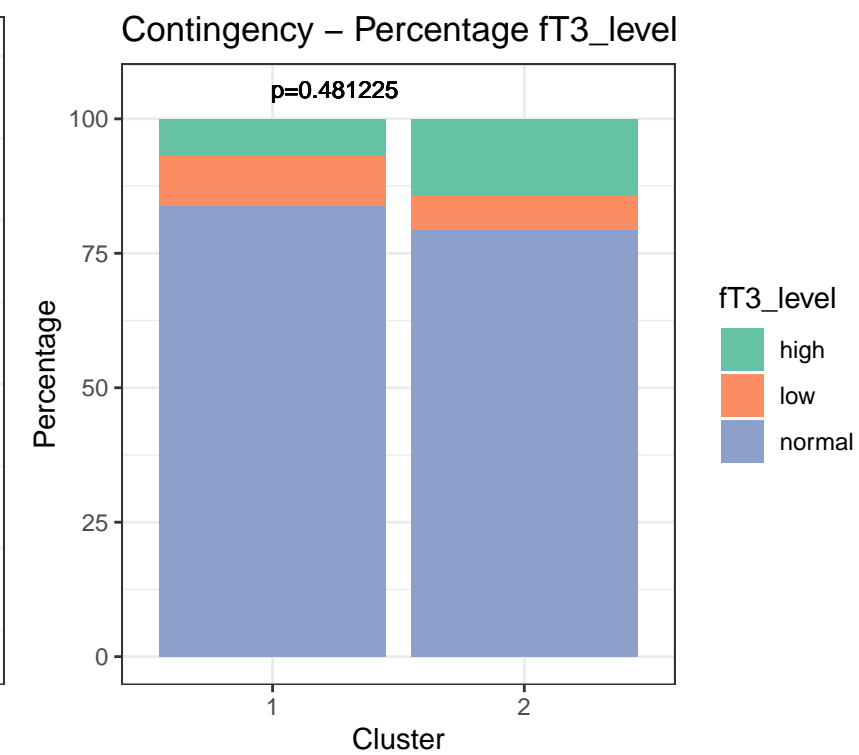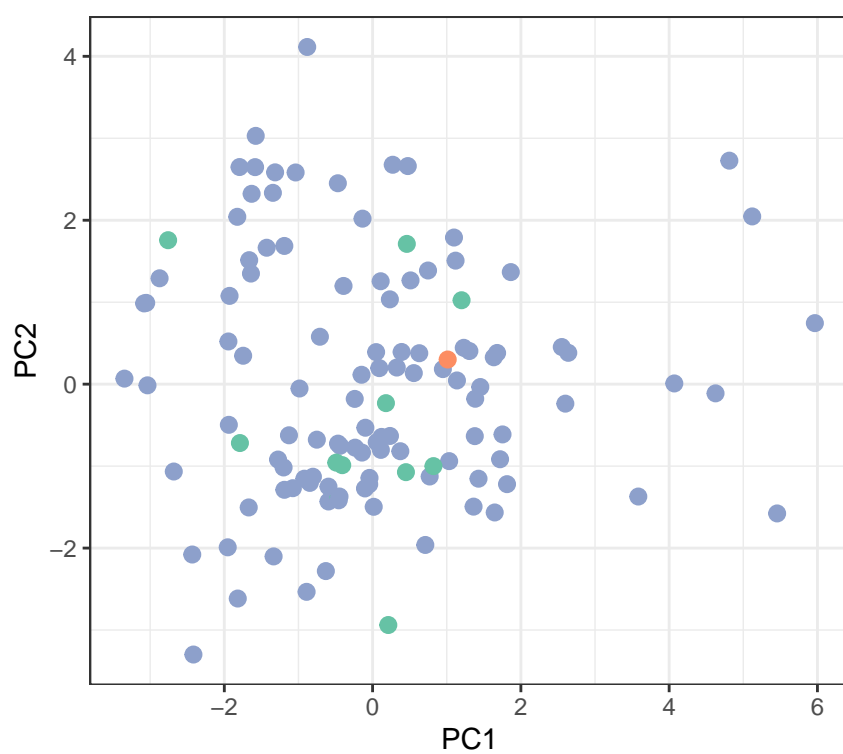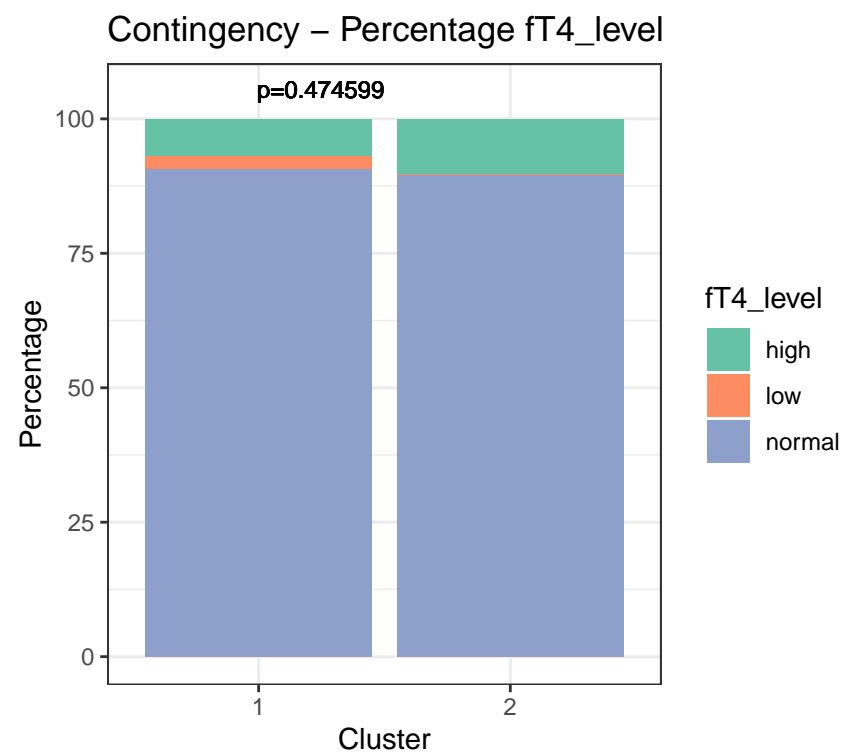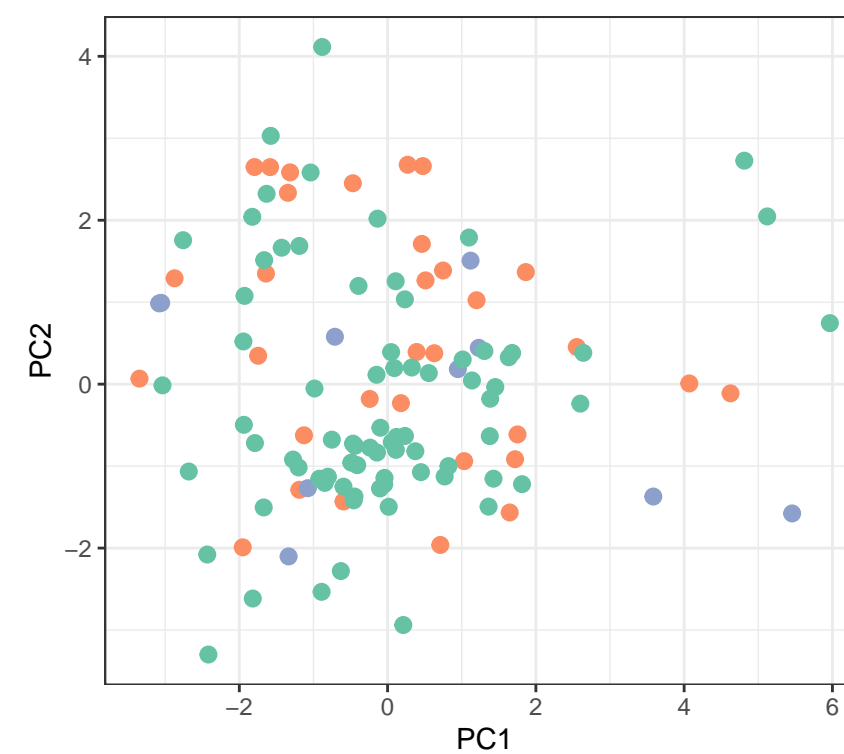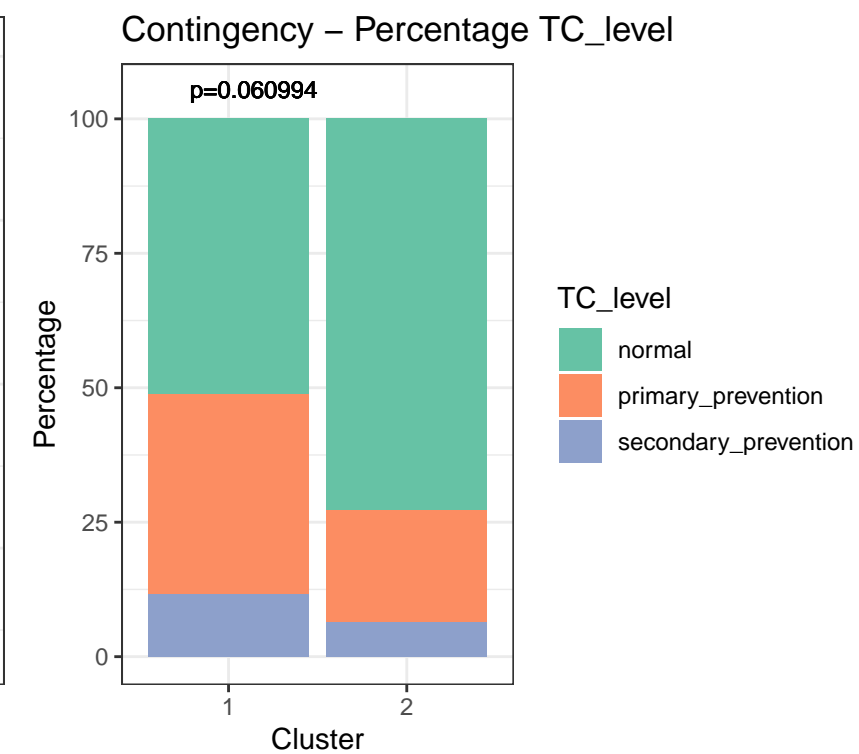

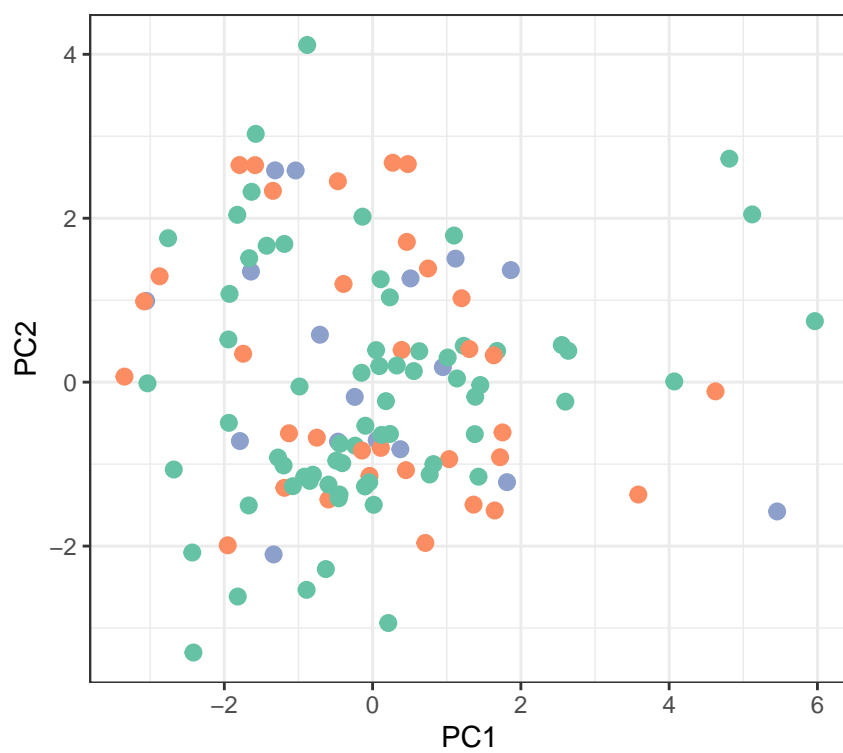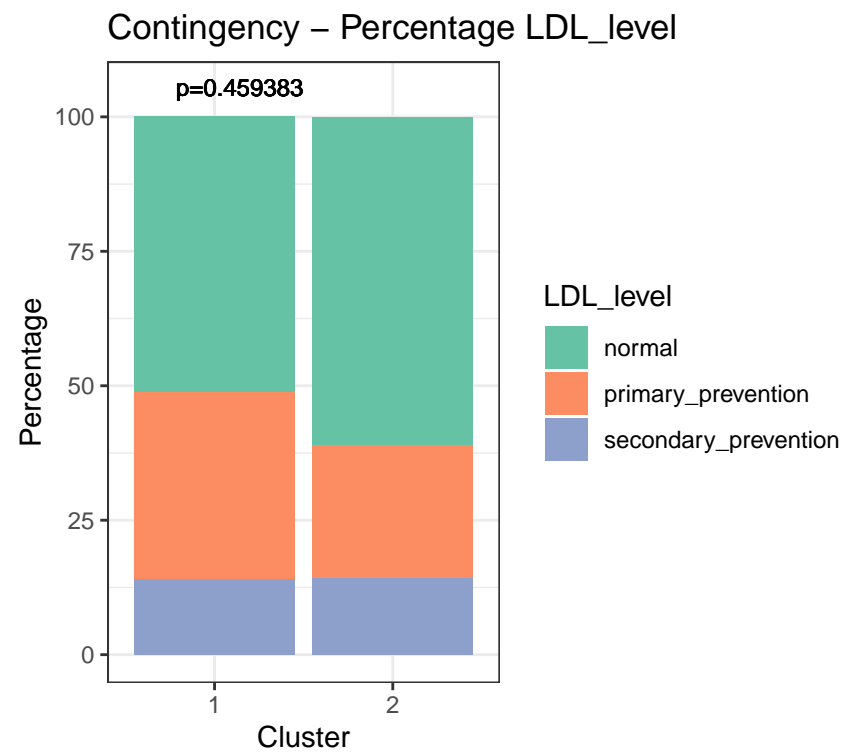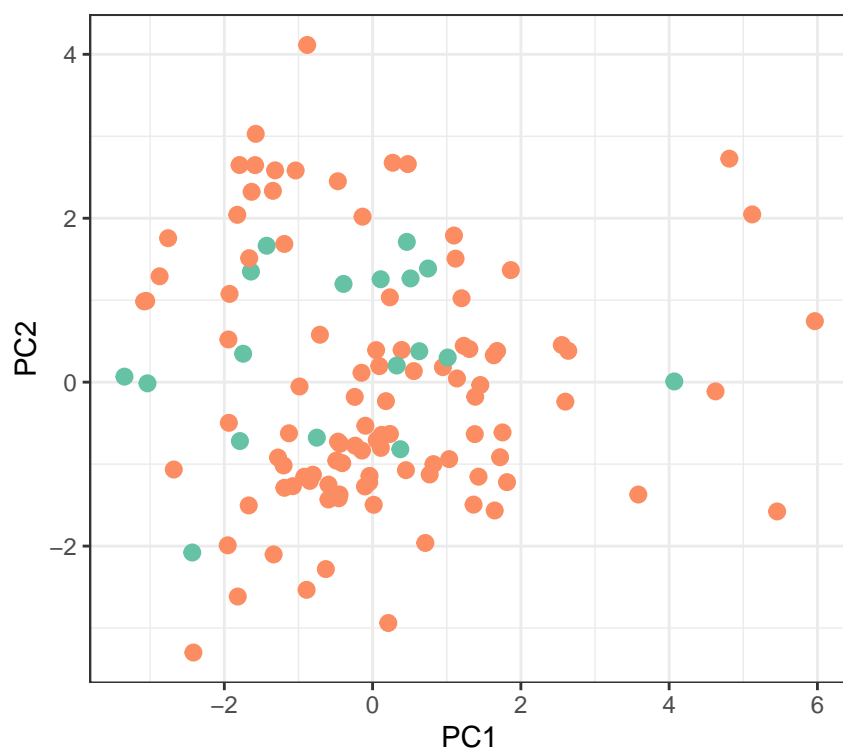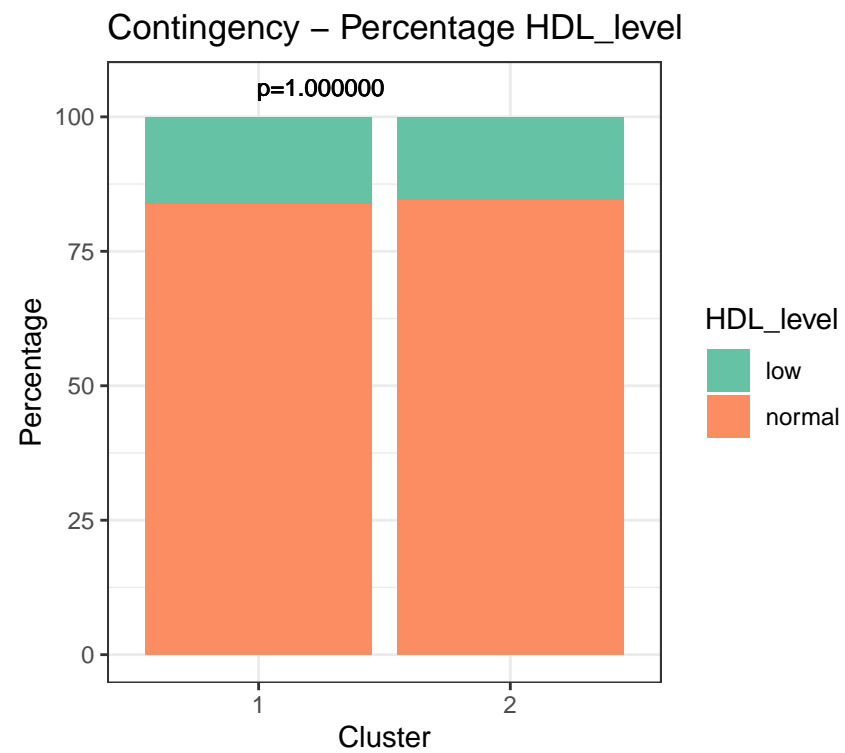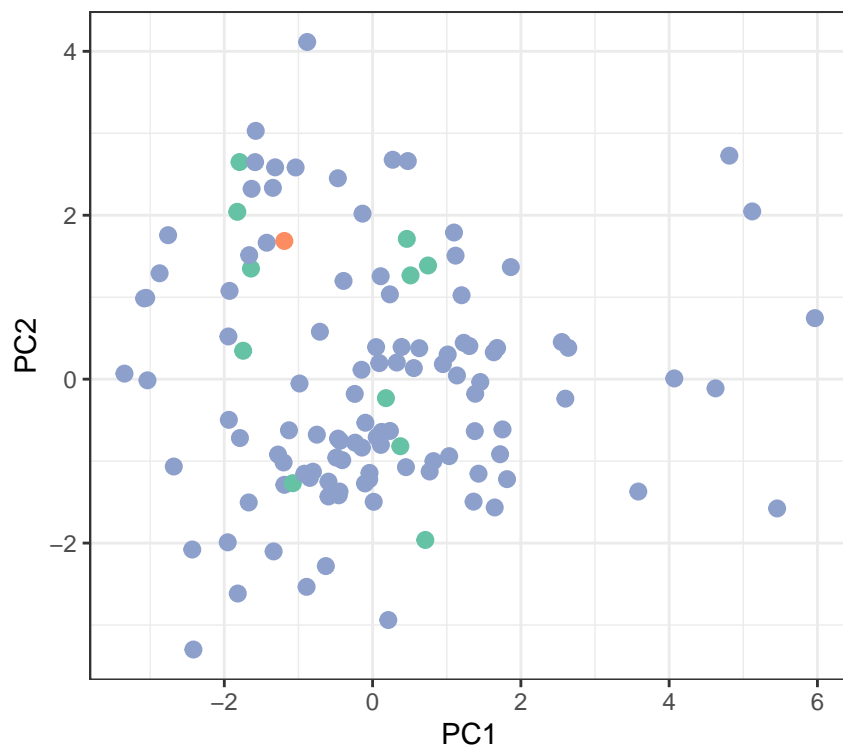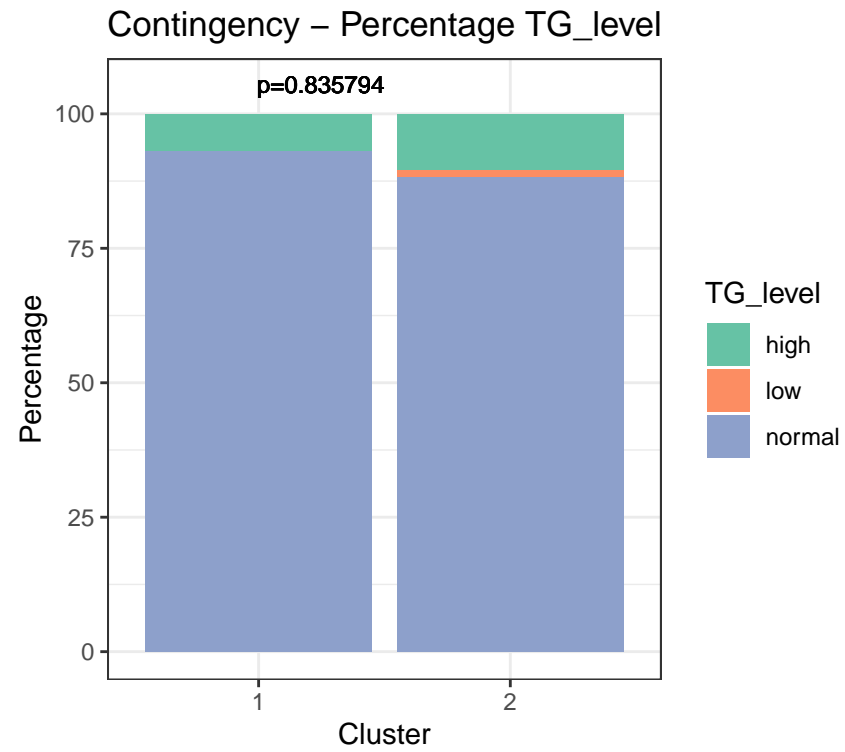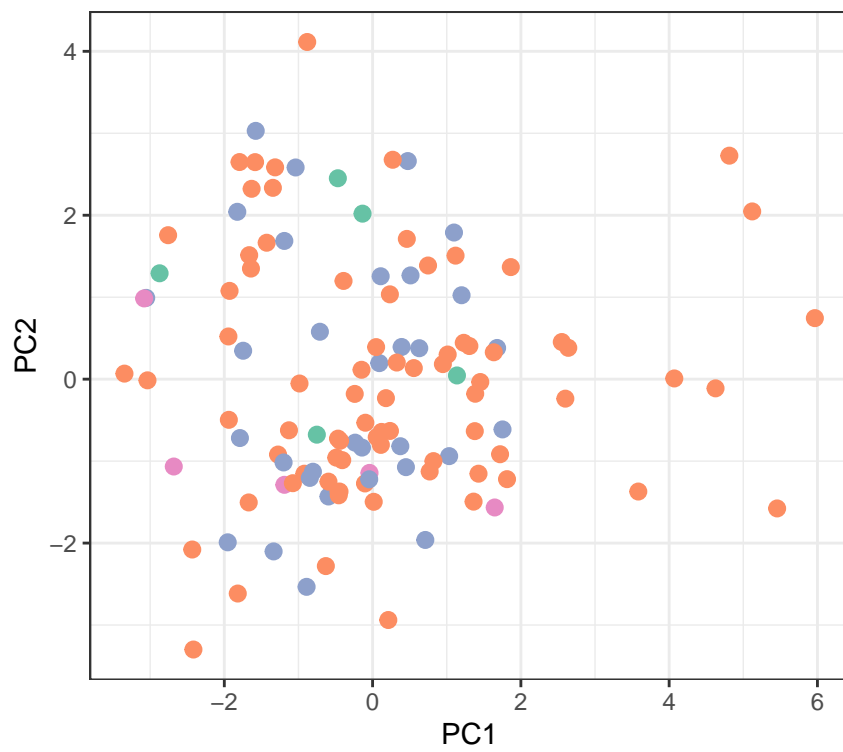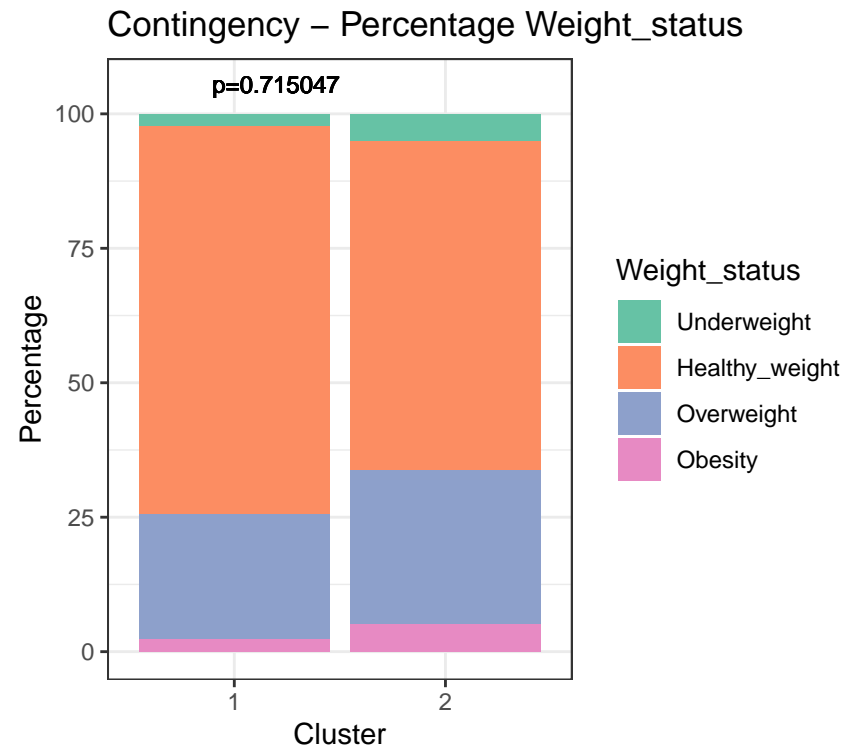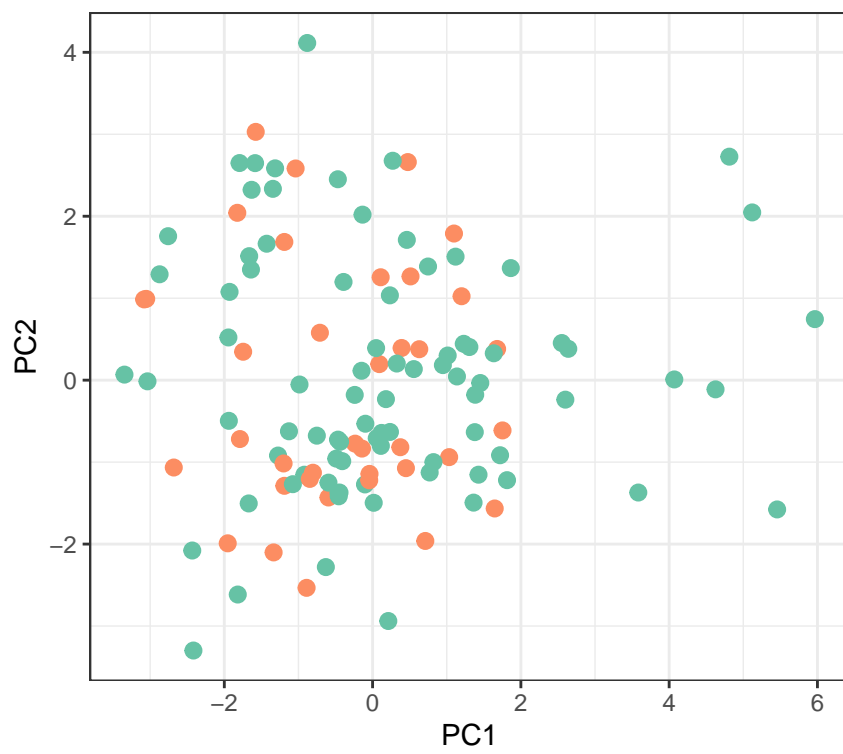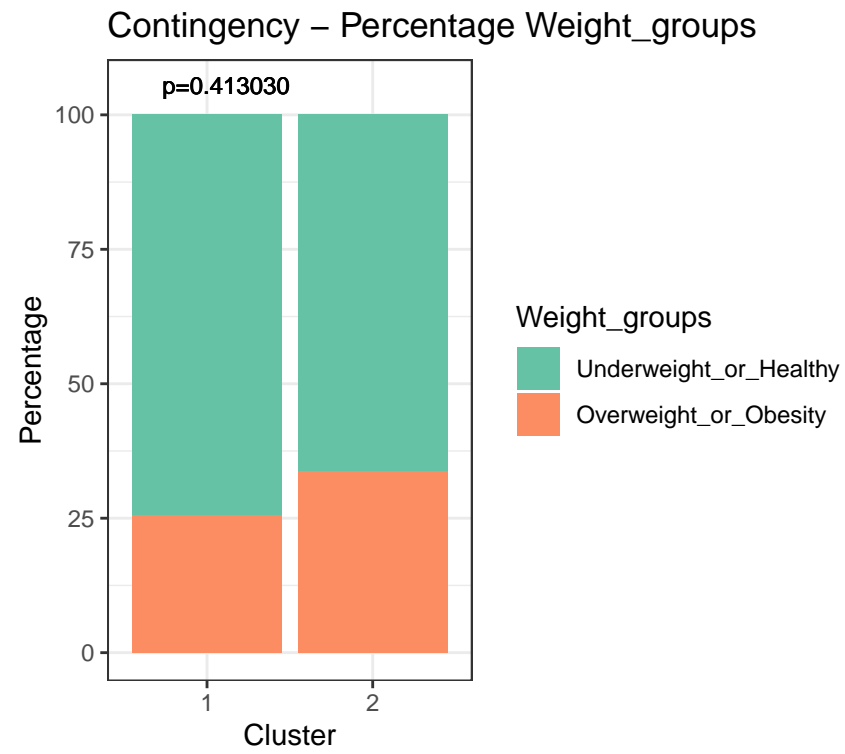

Supplement: Supplementary file 1 [file molecules-29-05169-s001.zip › molecules-3242400-supplementary/S5_all_patients_acylcarnitines.pdf]

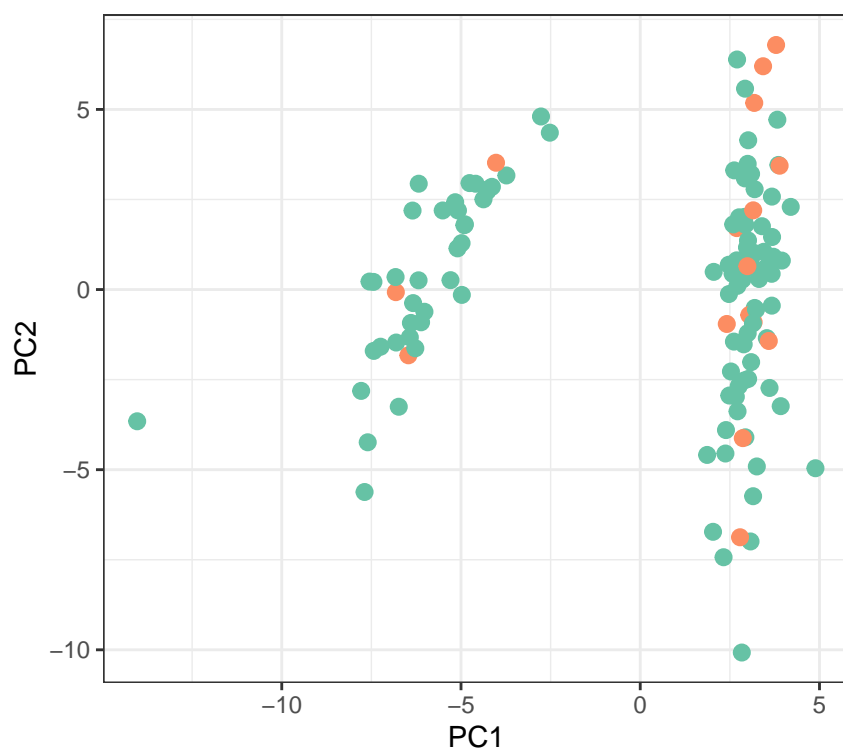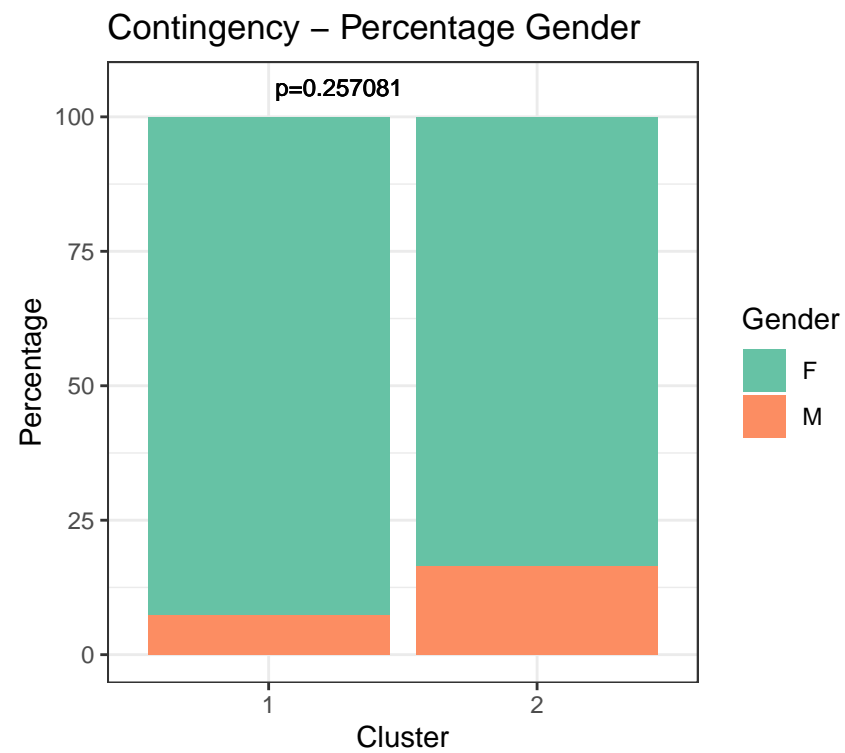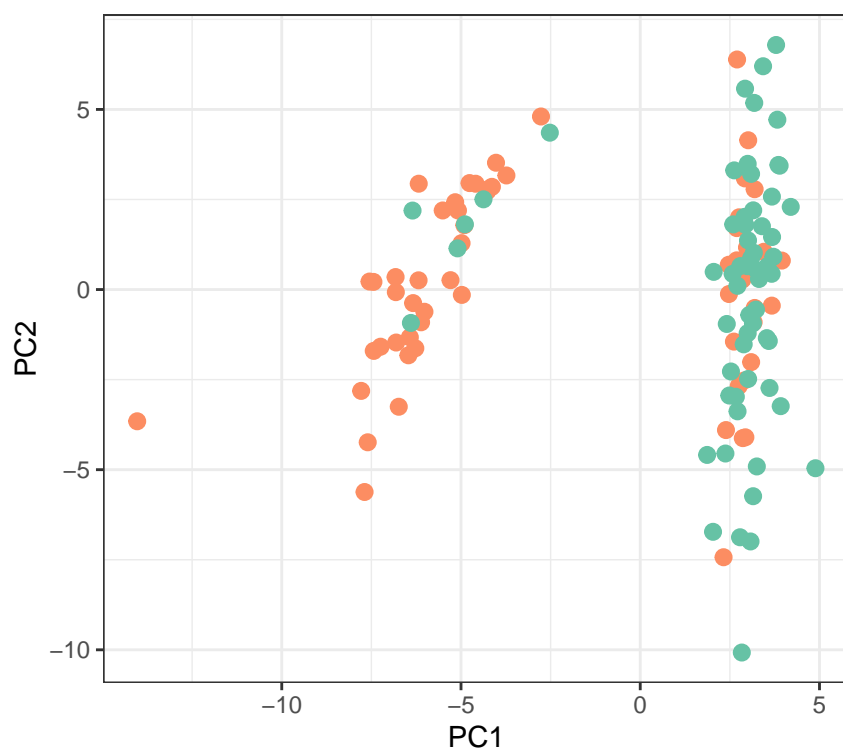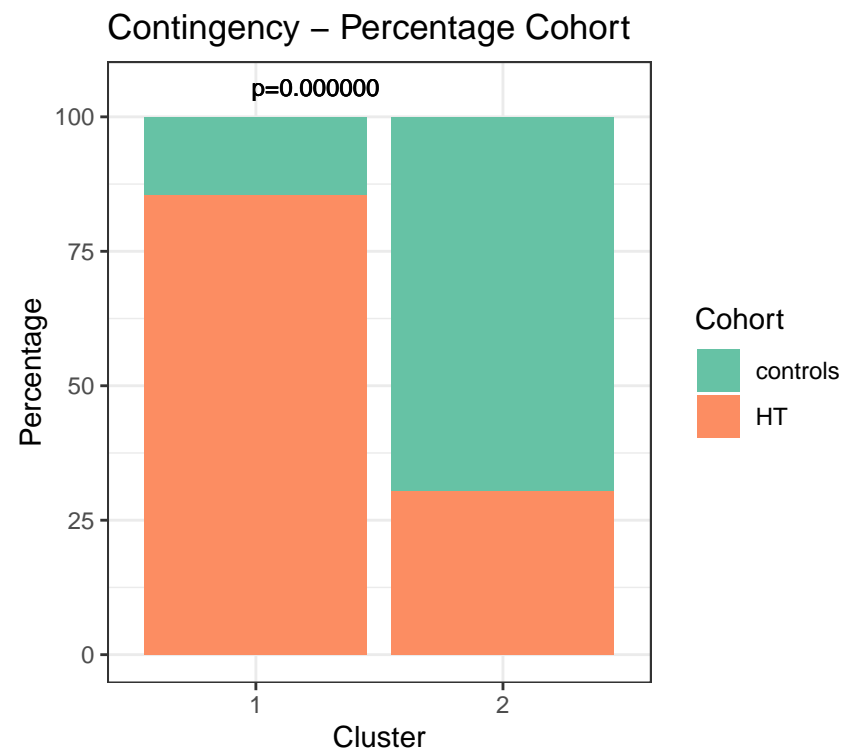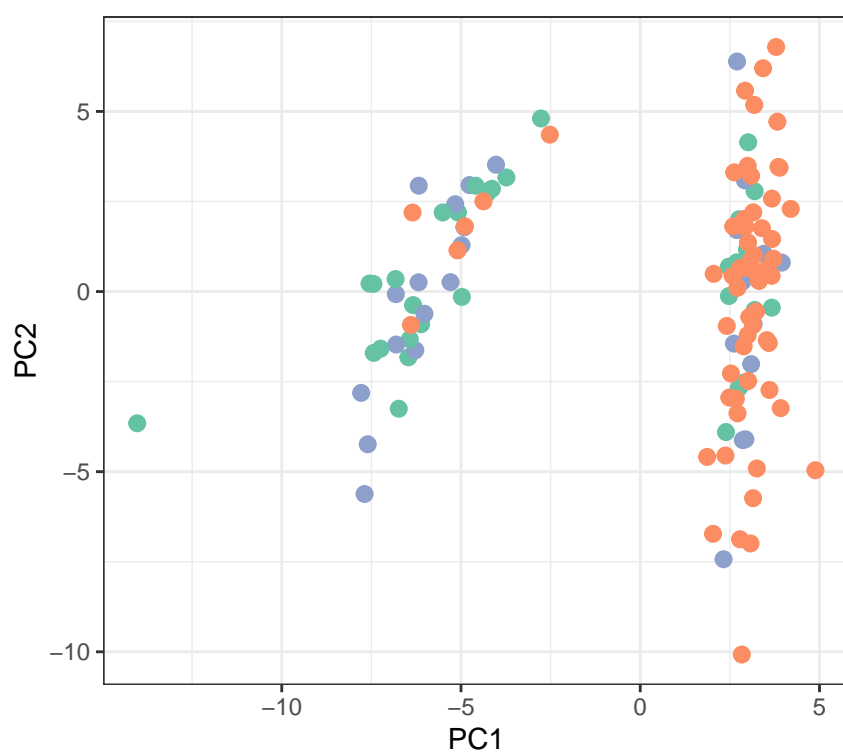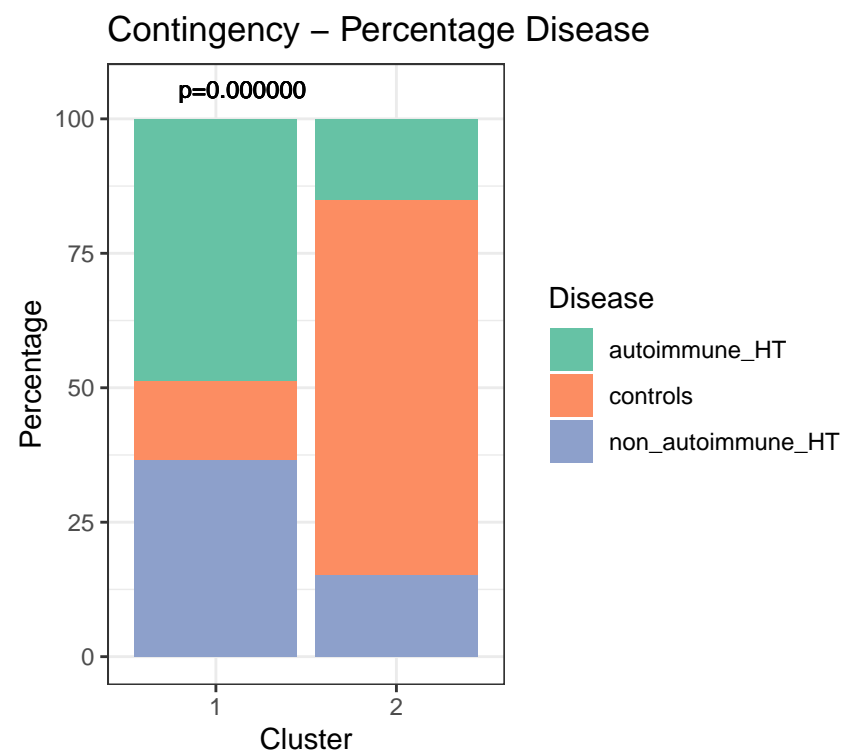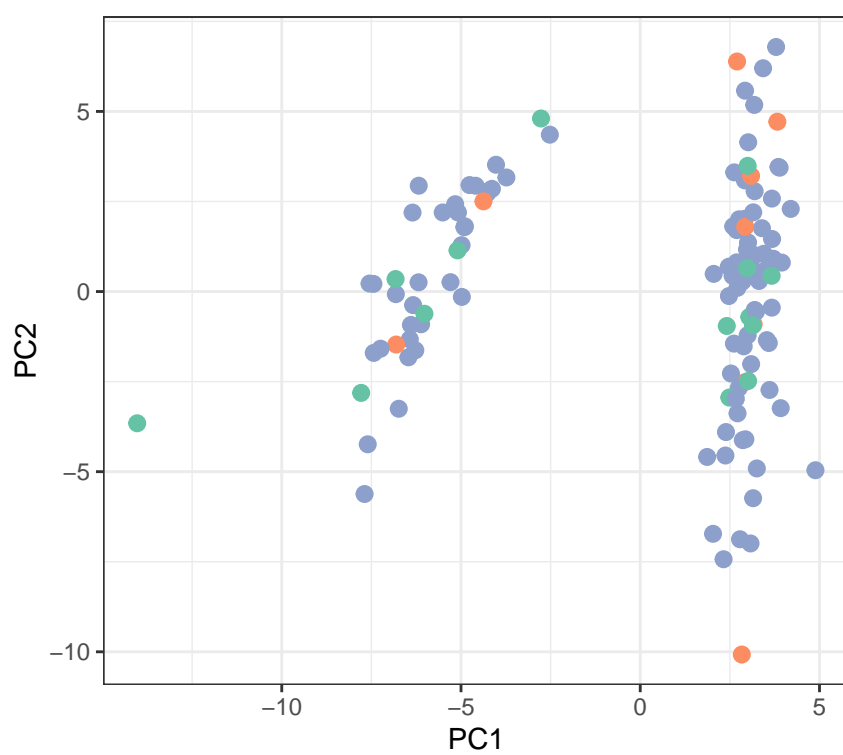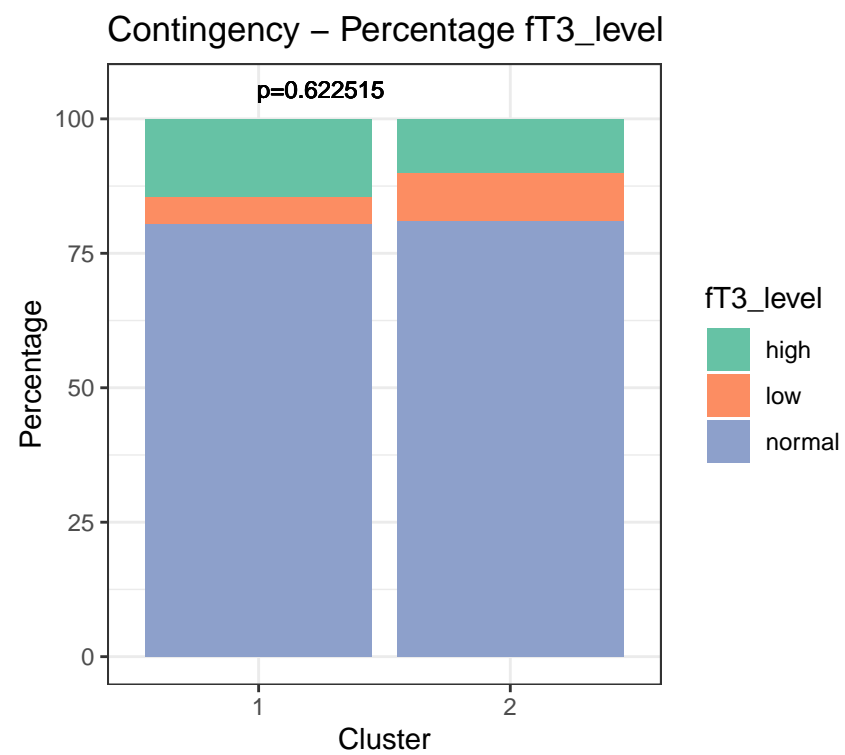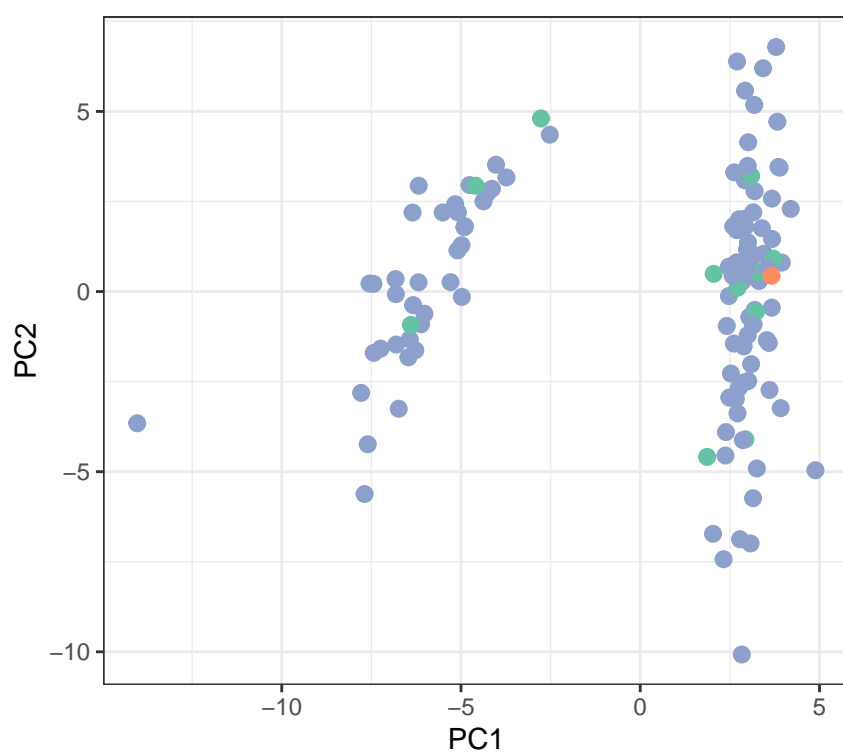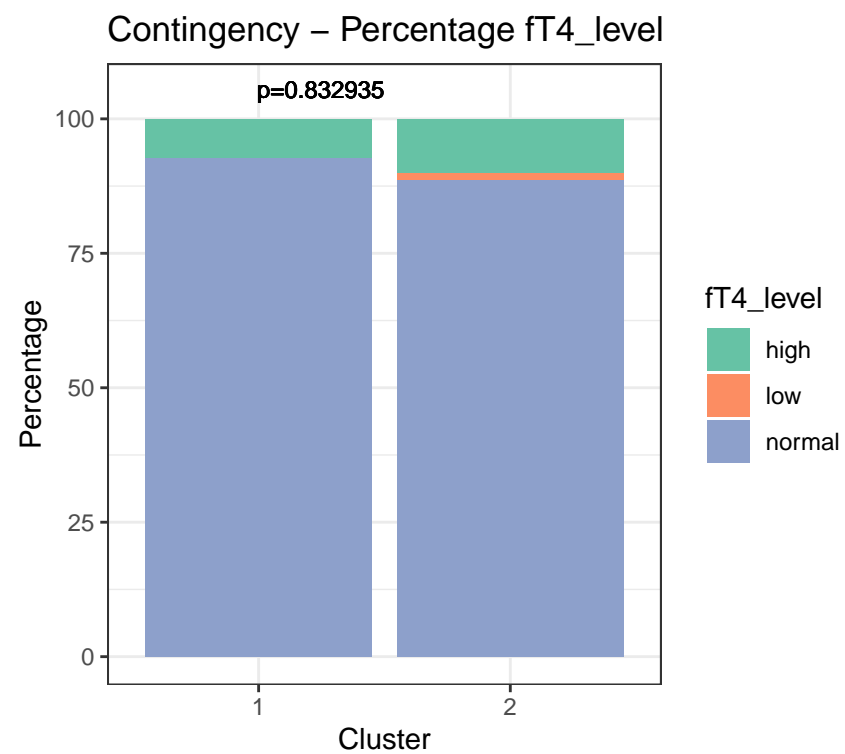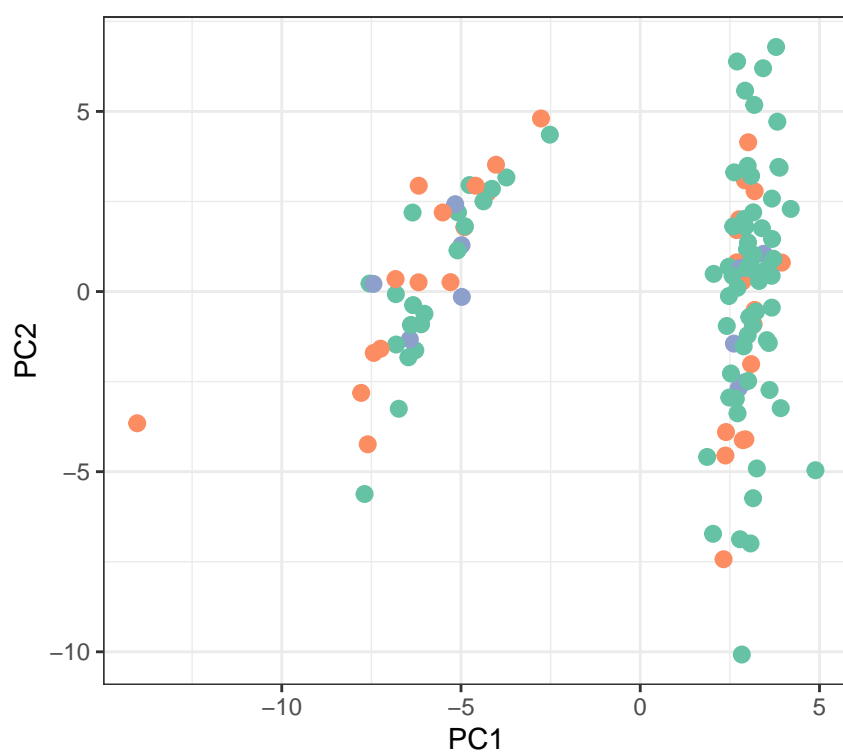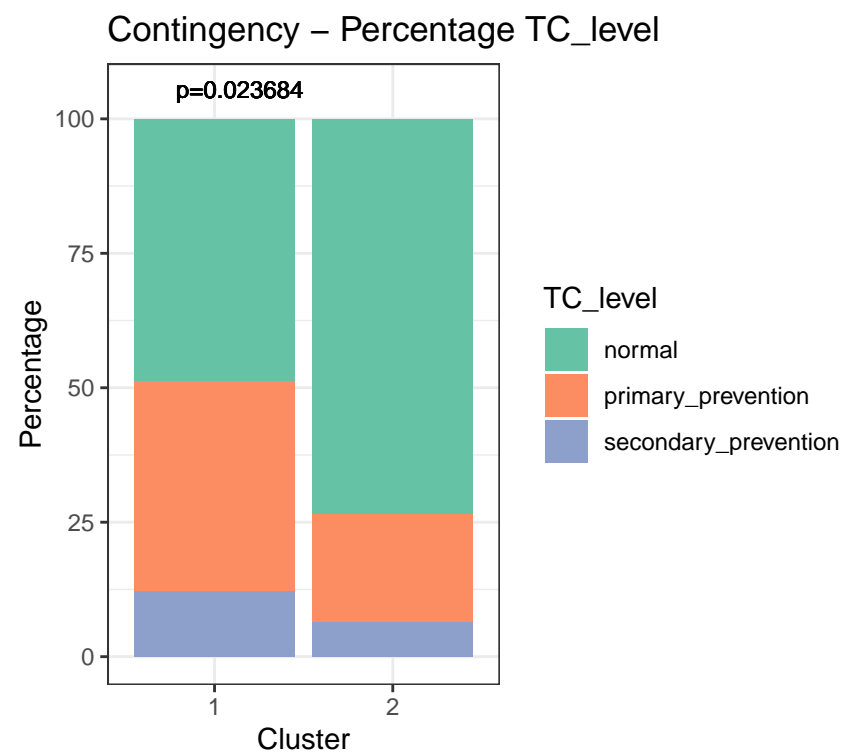

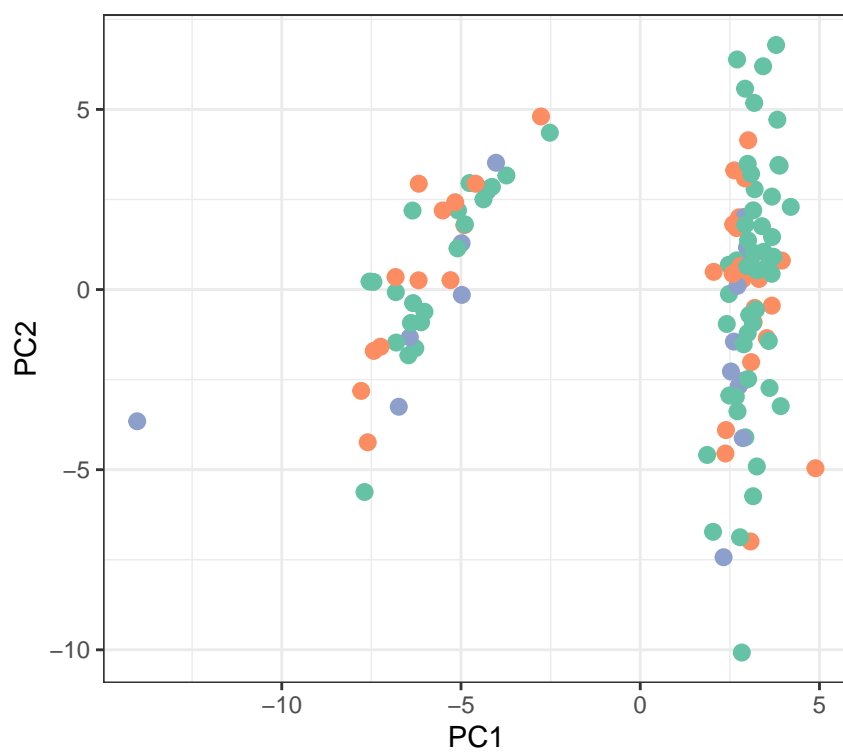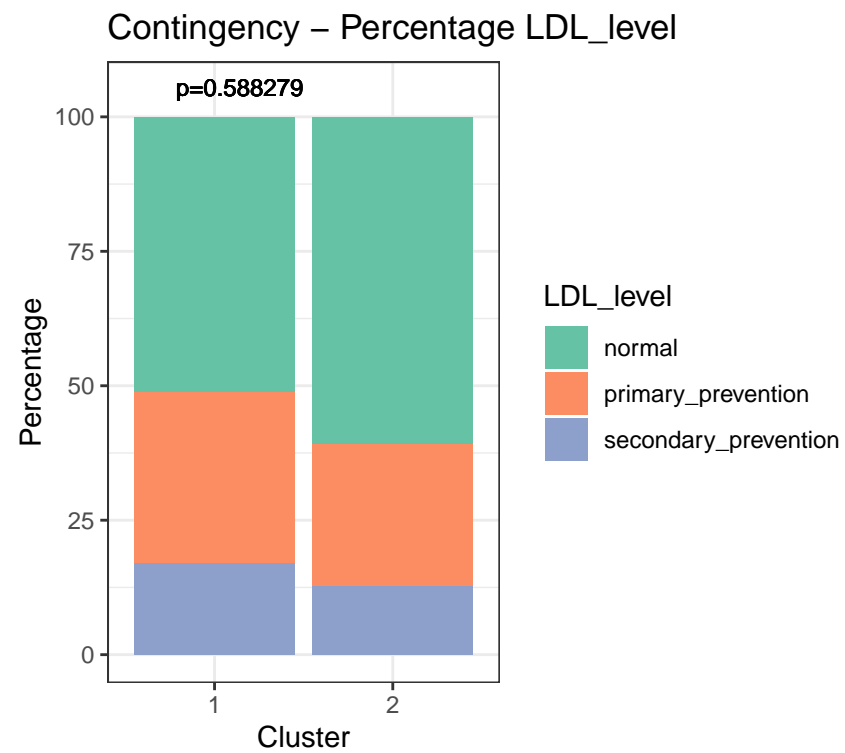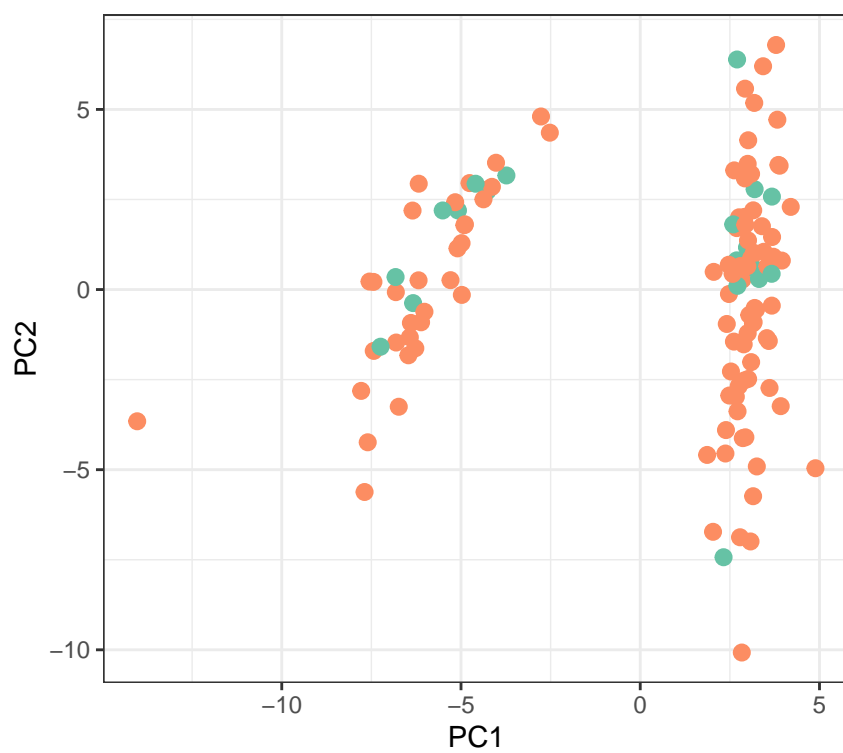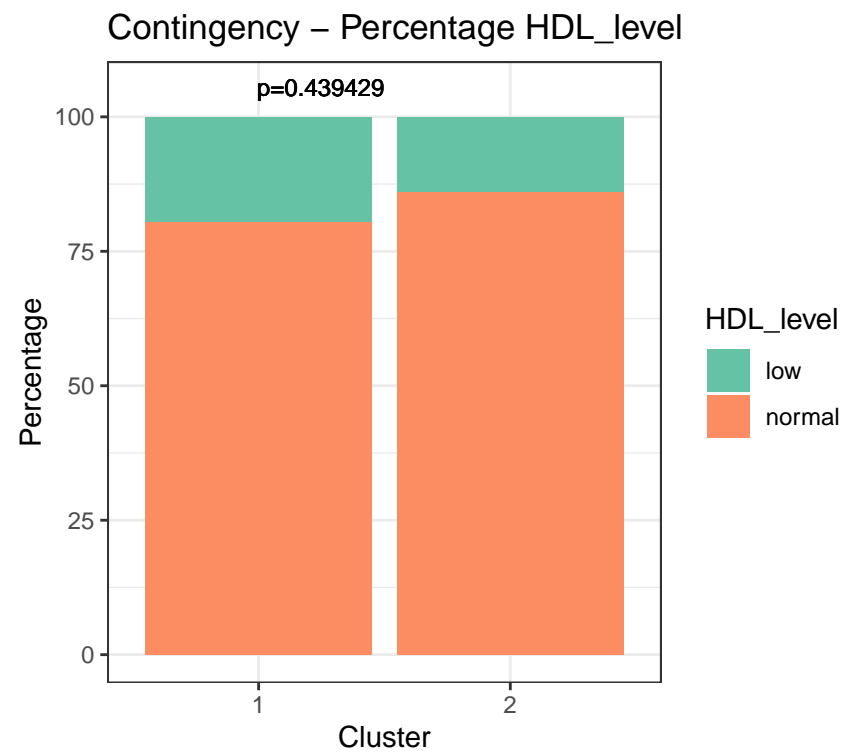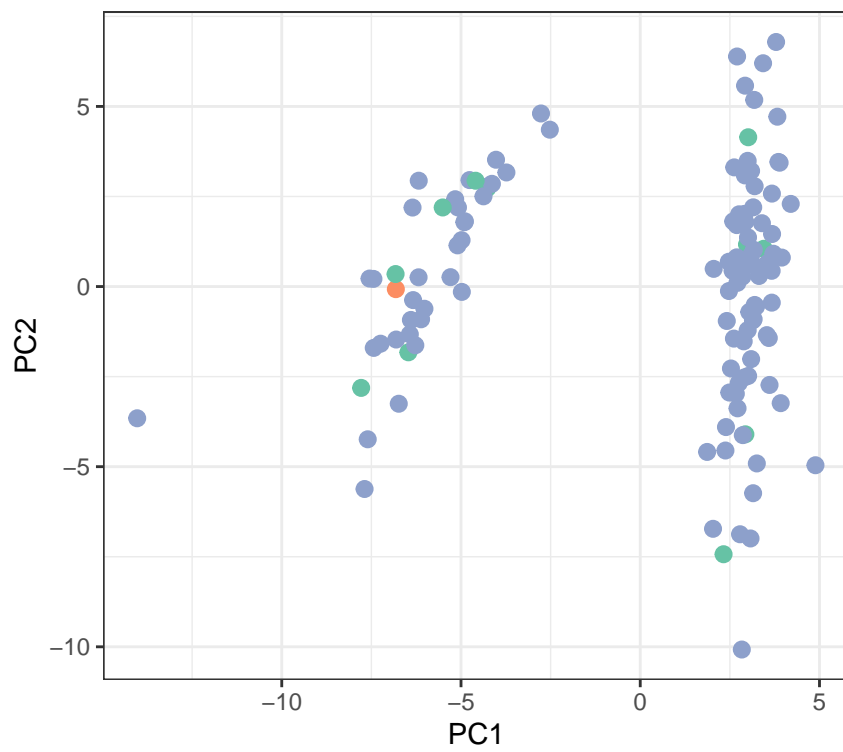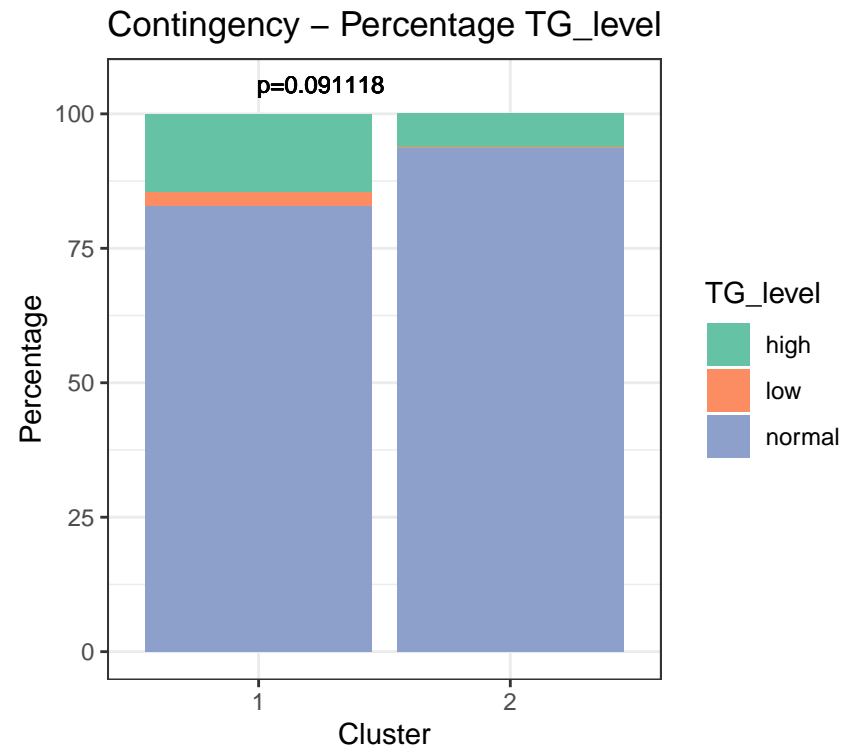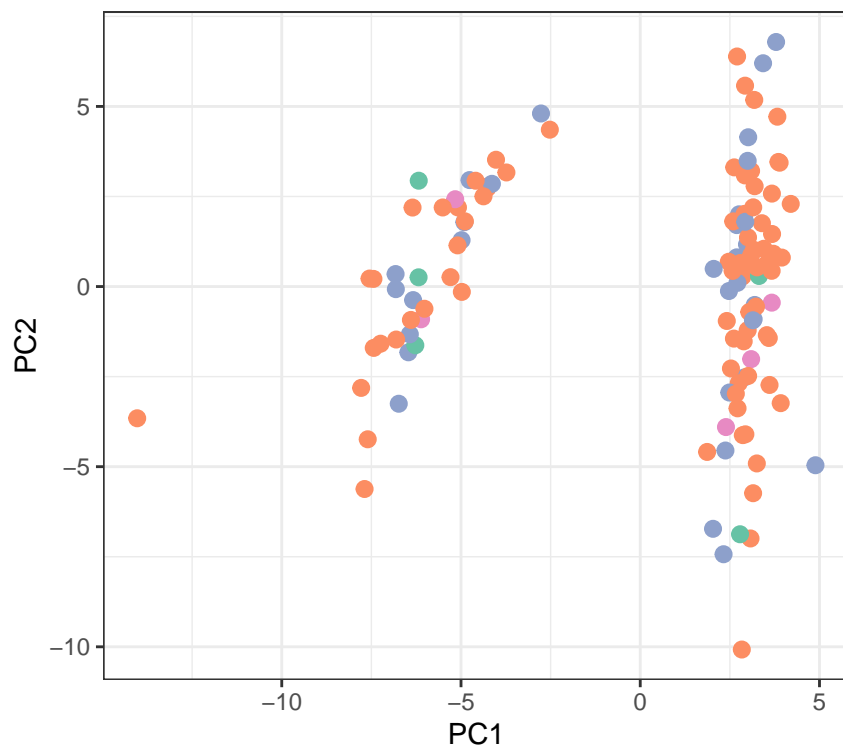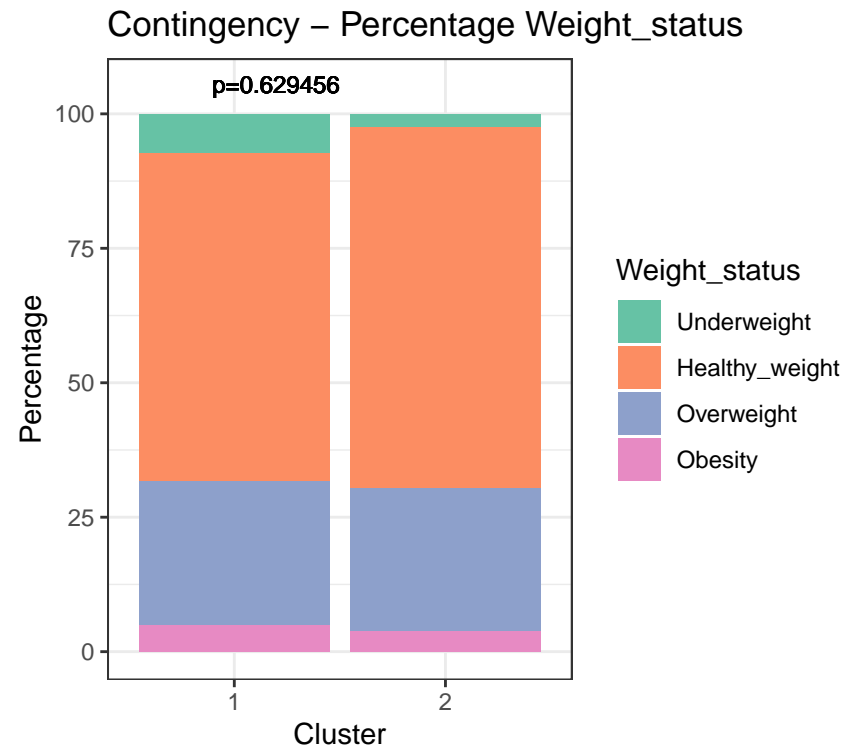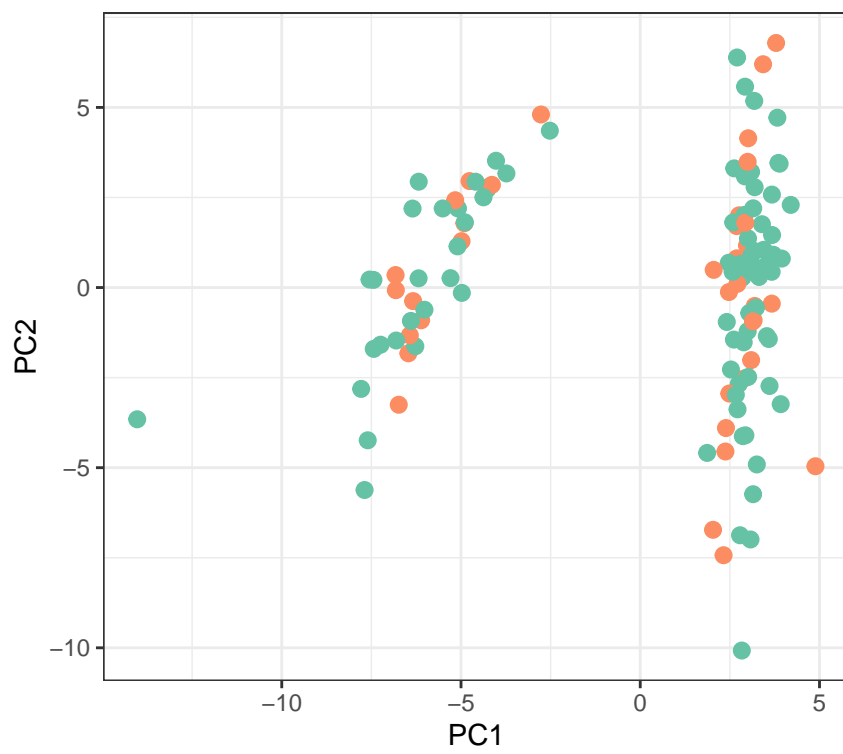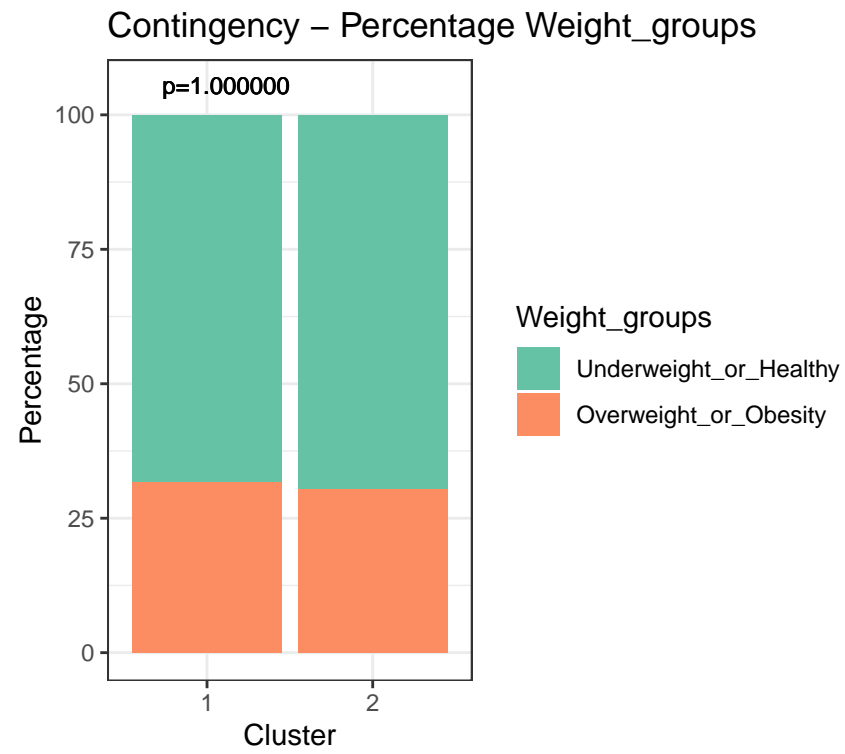

Supplement: Supplementary file 1 [file molecules-29-05169-s001.zip › molecules-3242400-supplementary/S6_all_patients_glycerophospholipids.pdf]

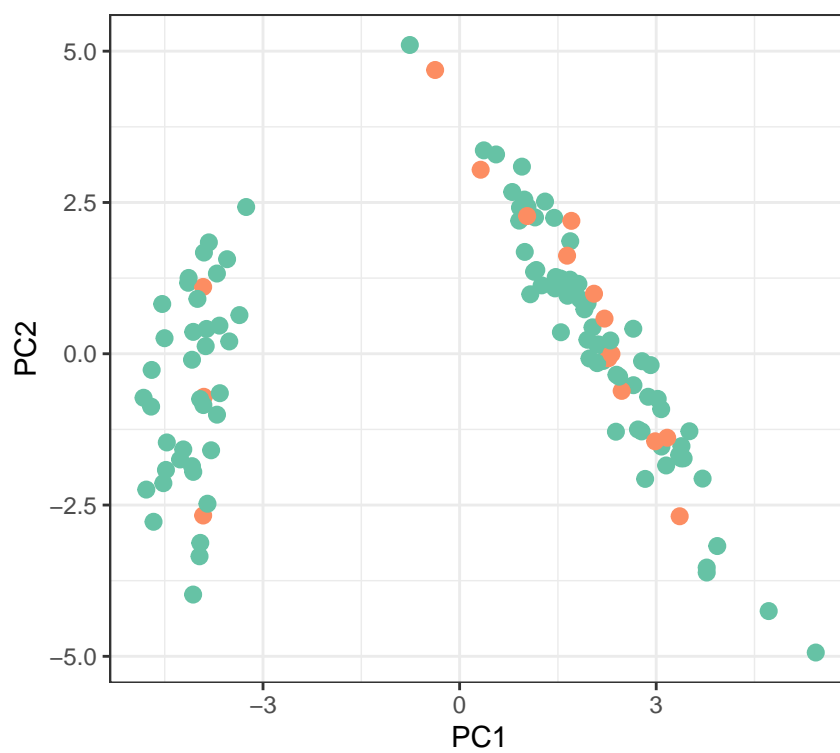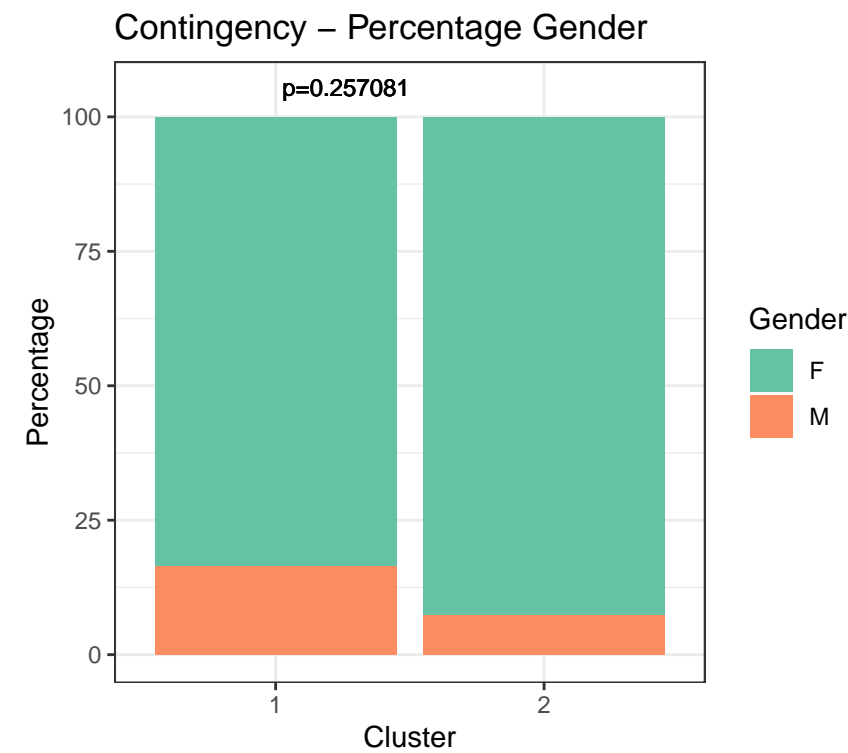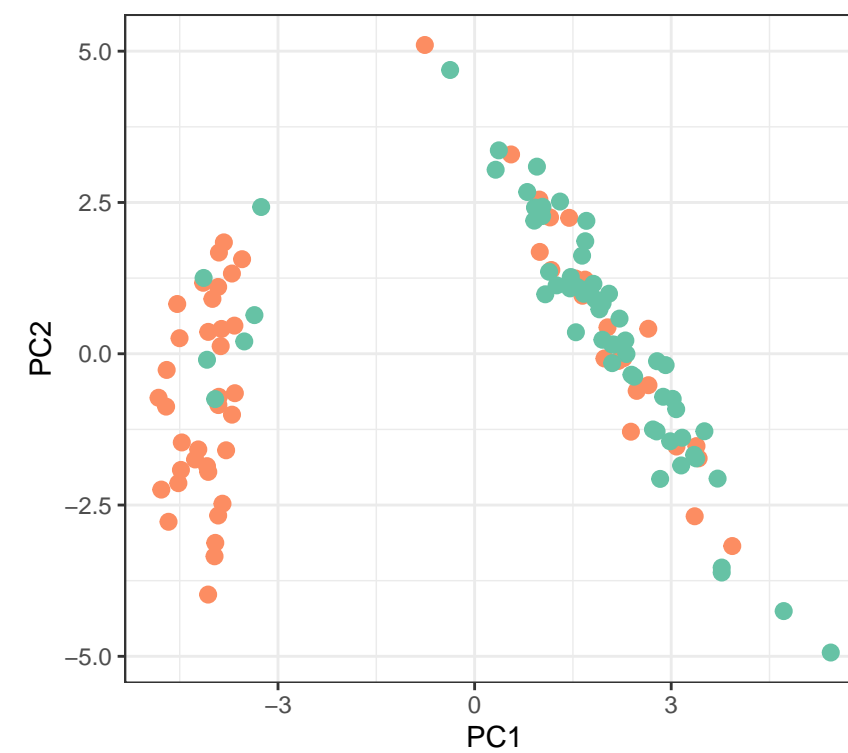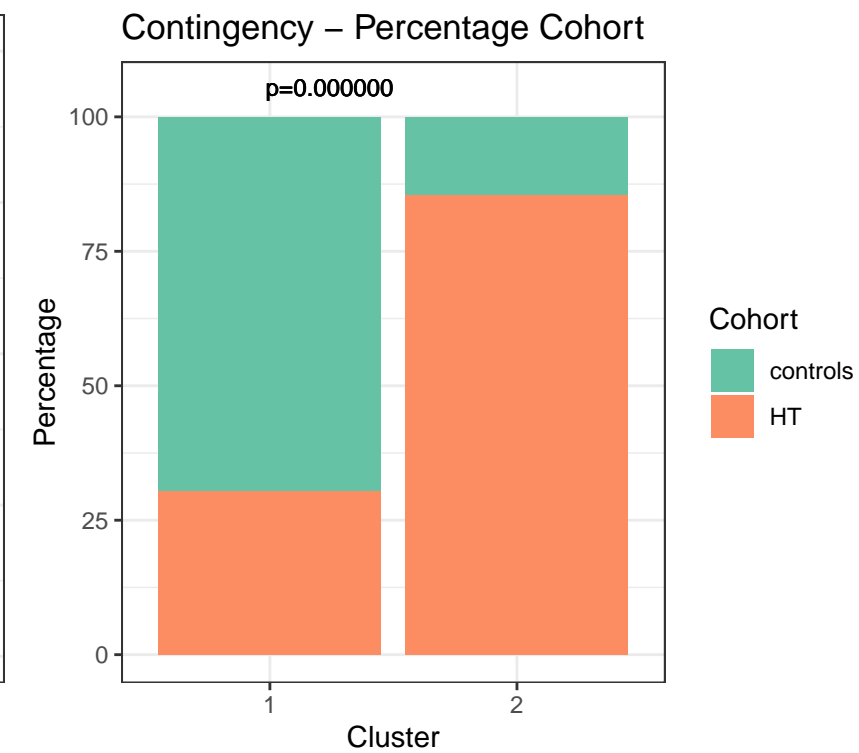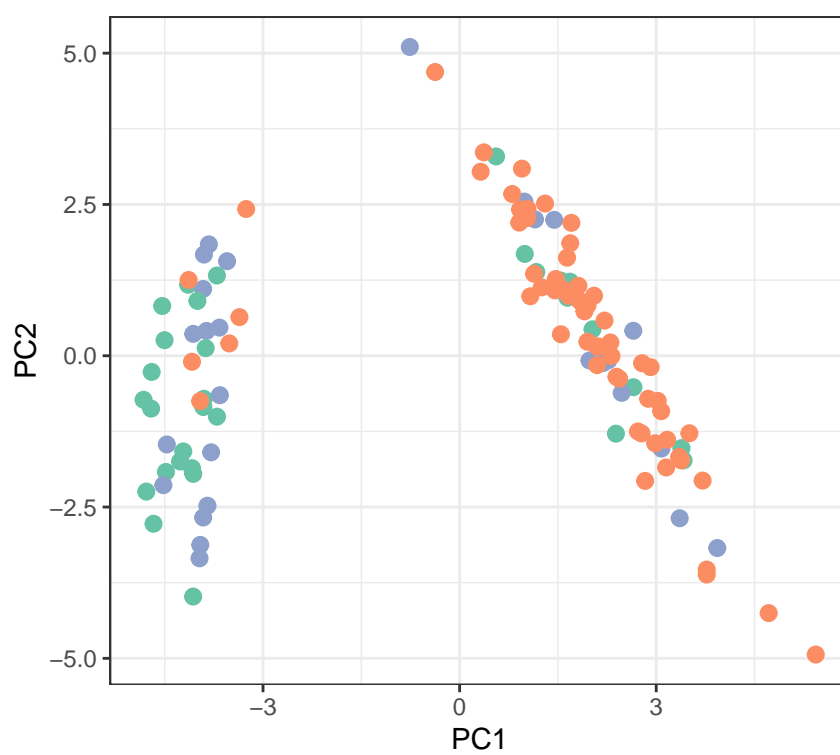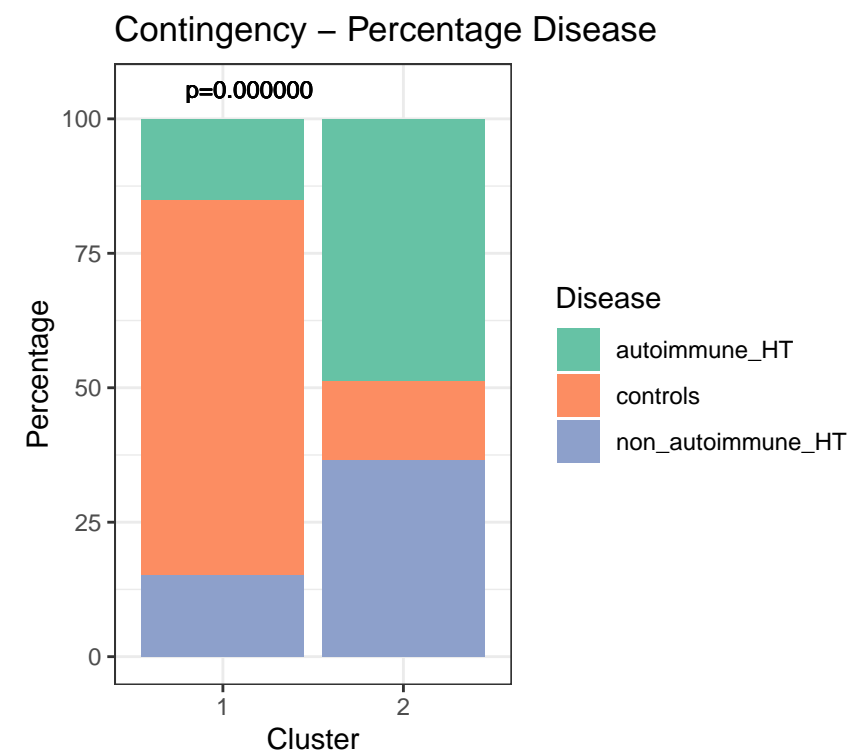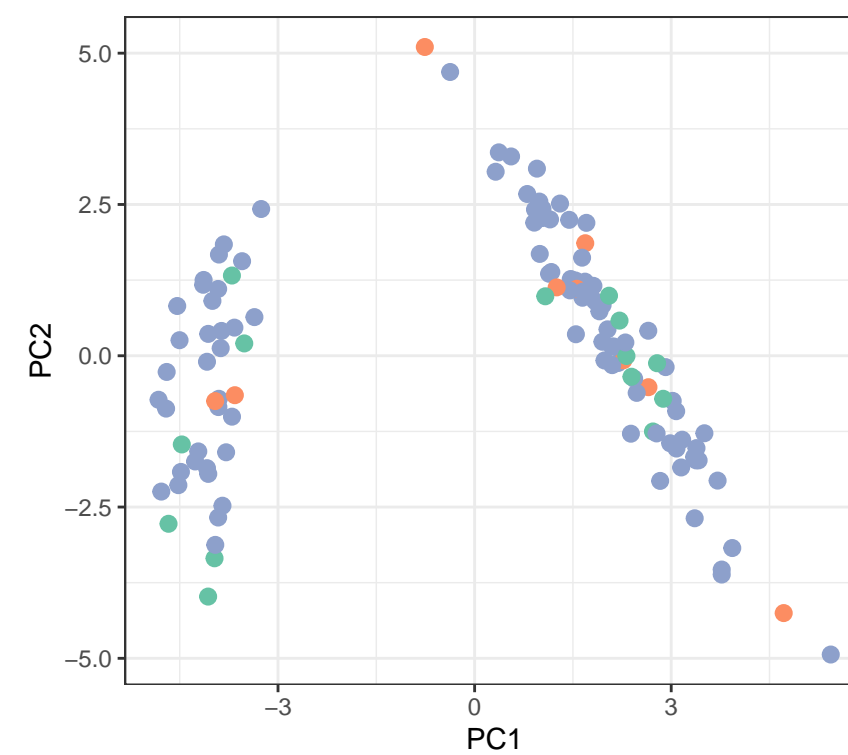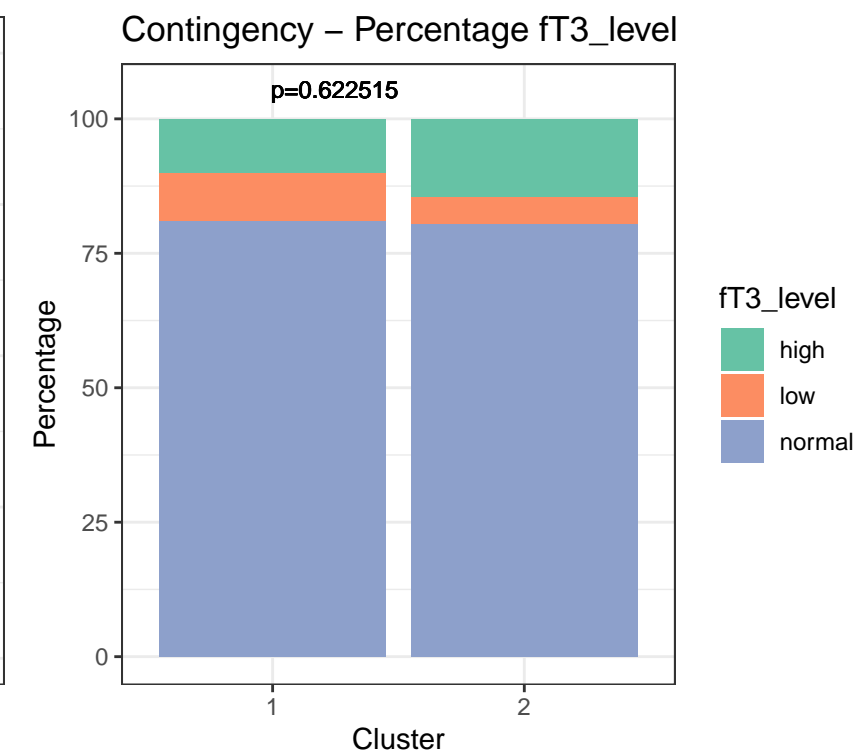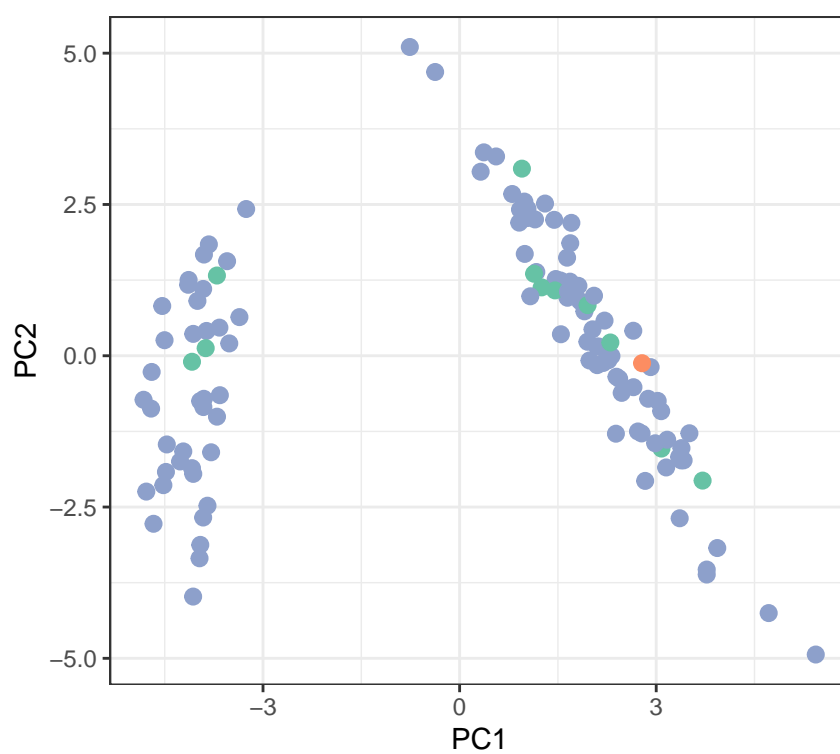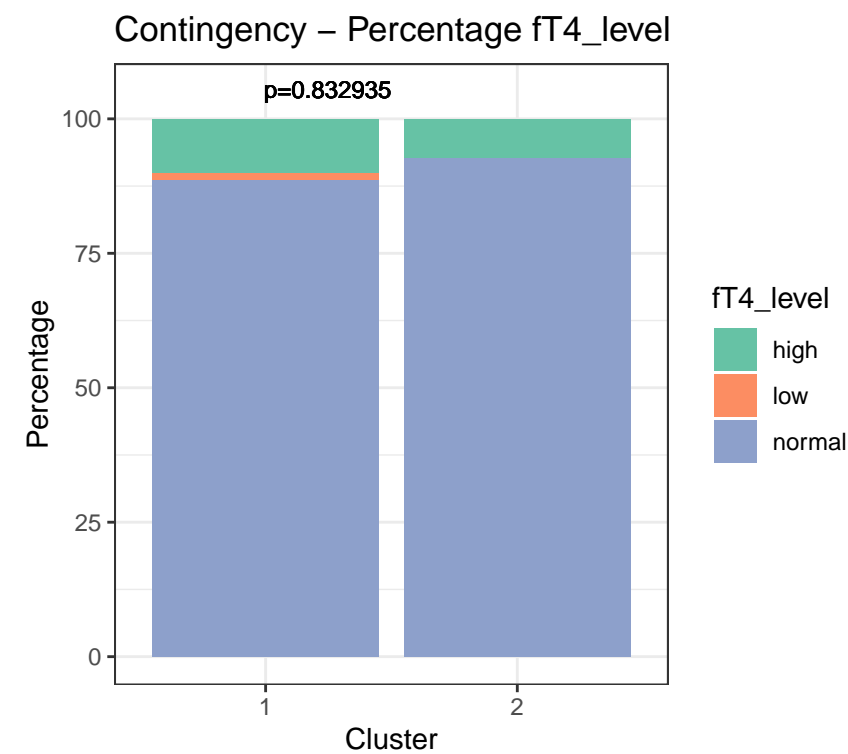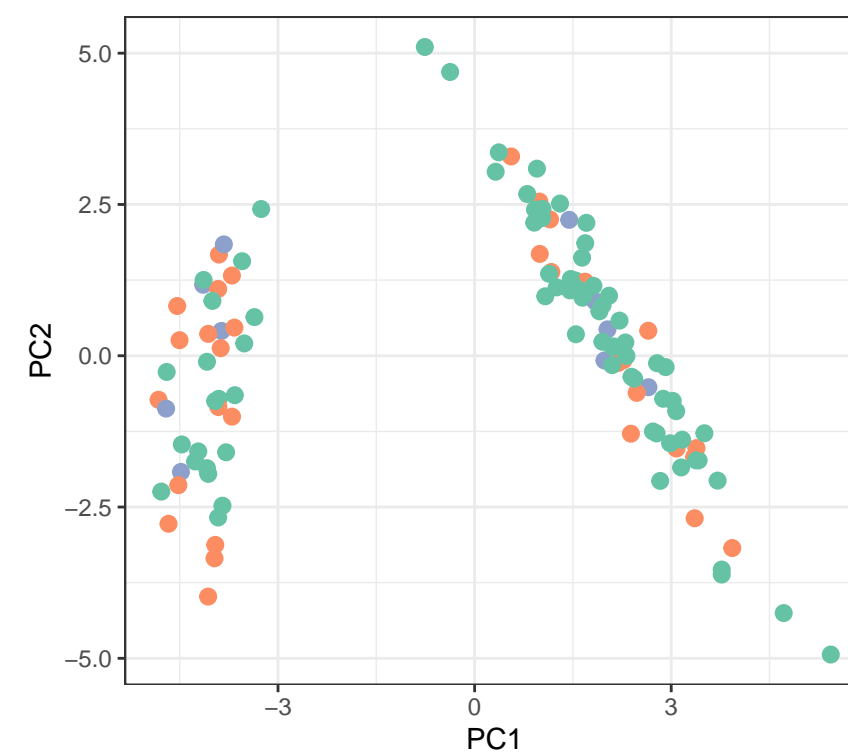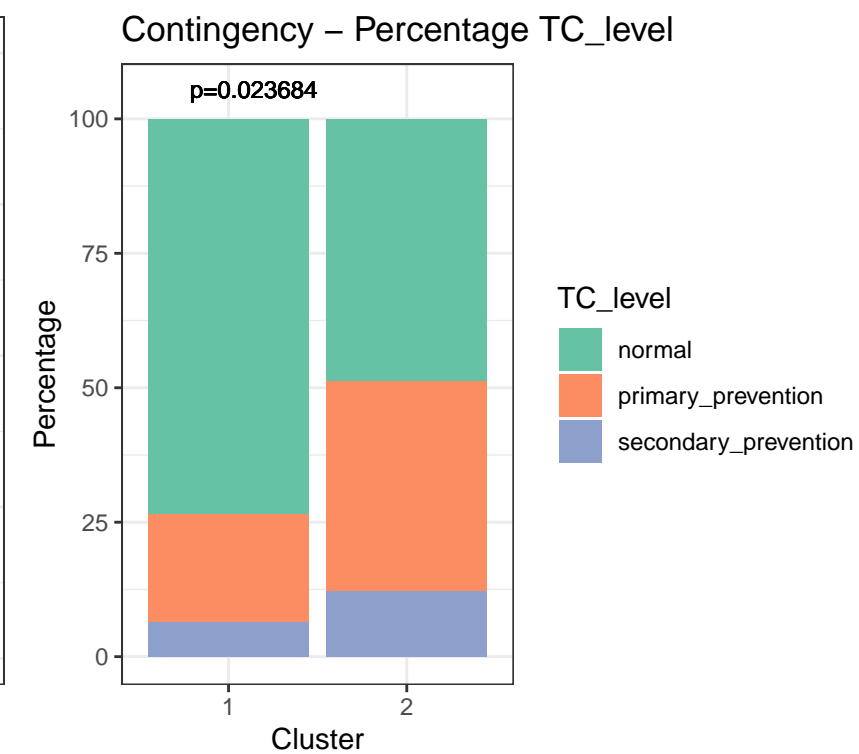

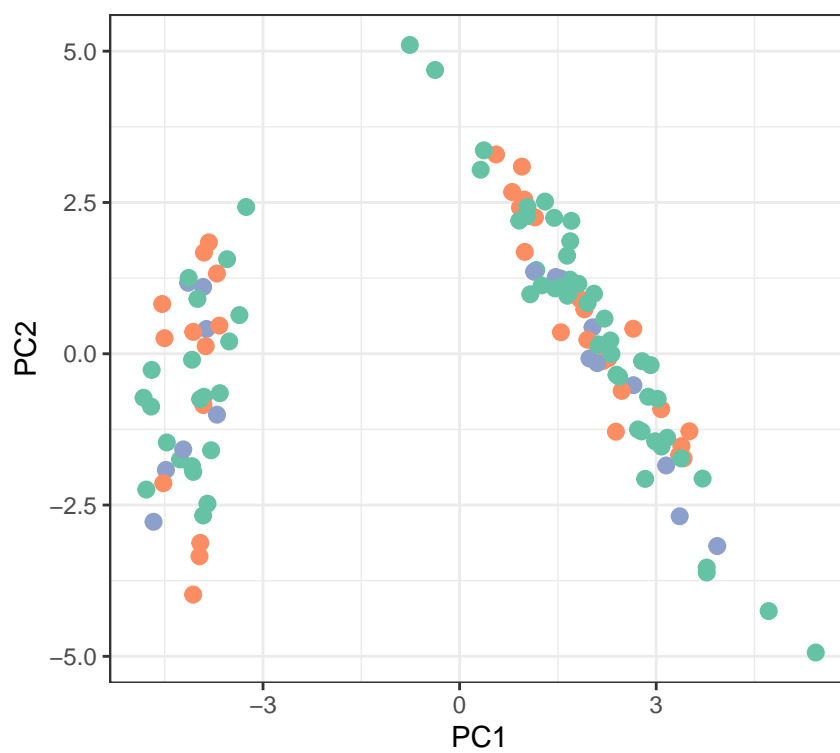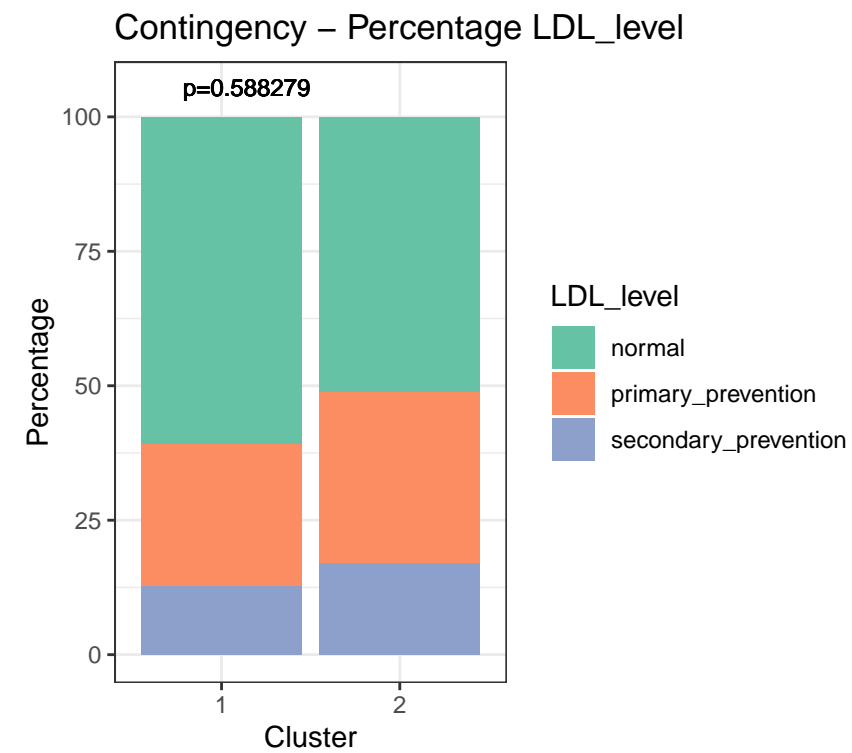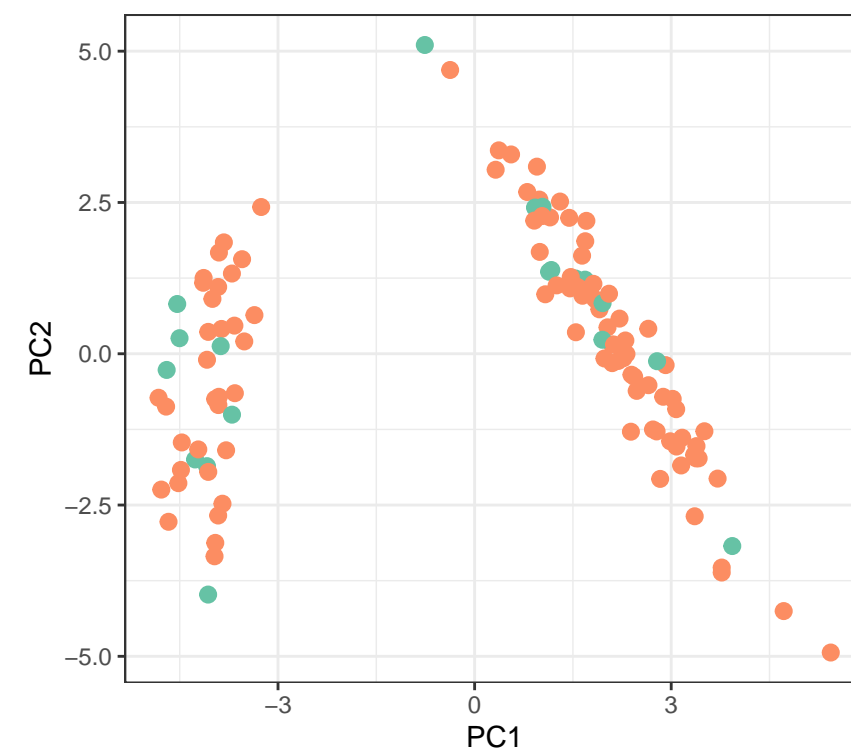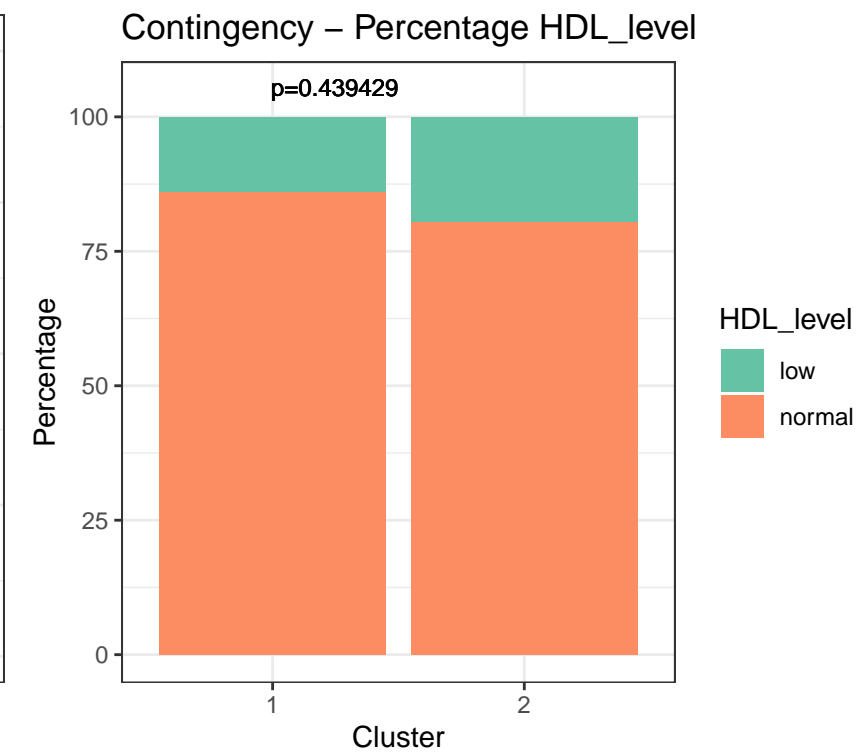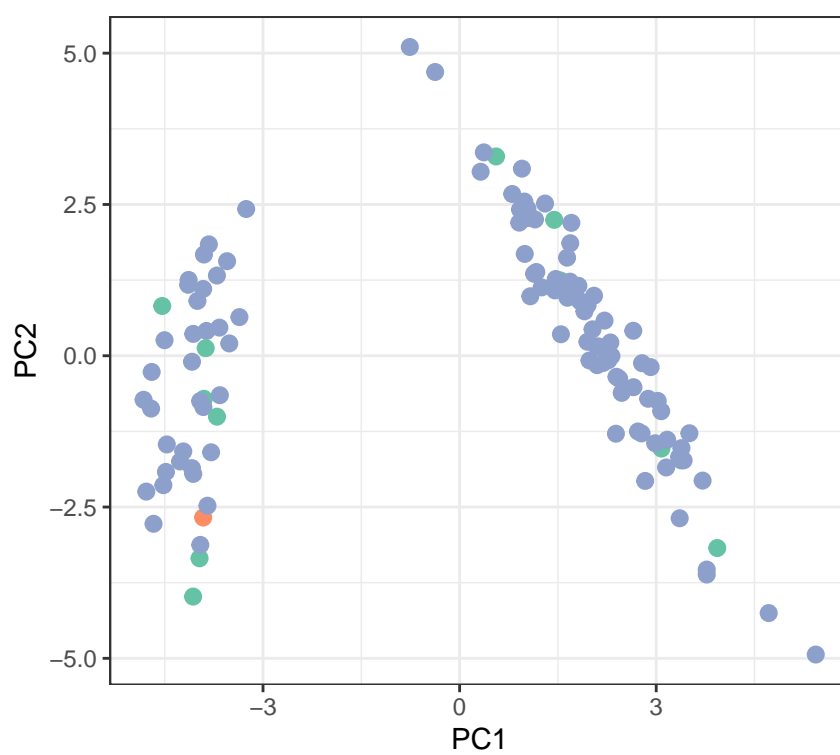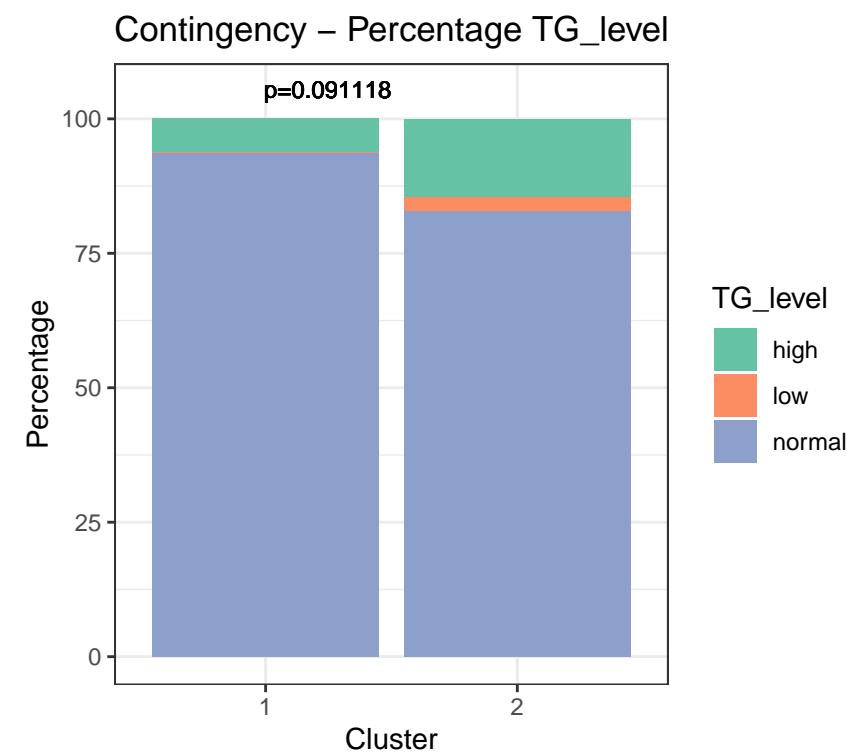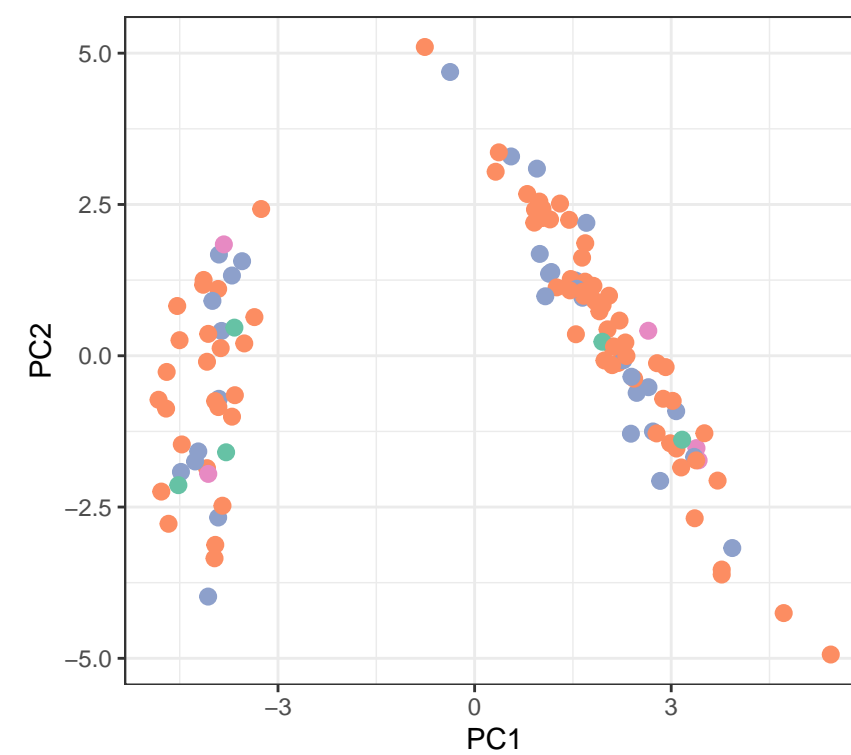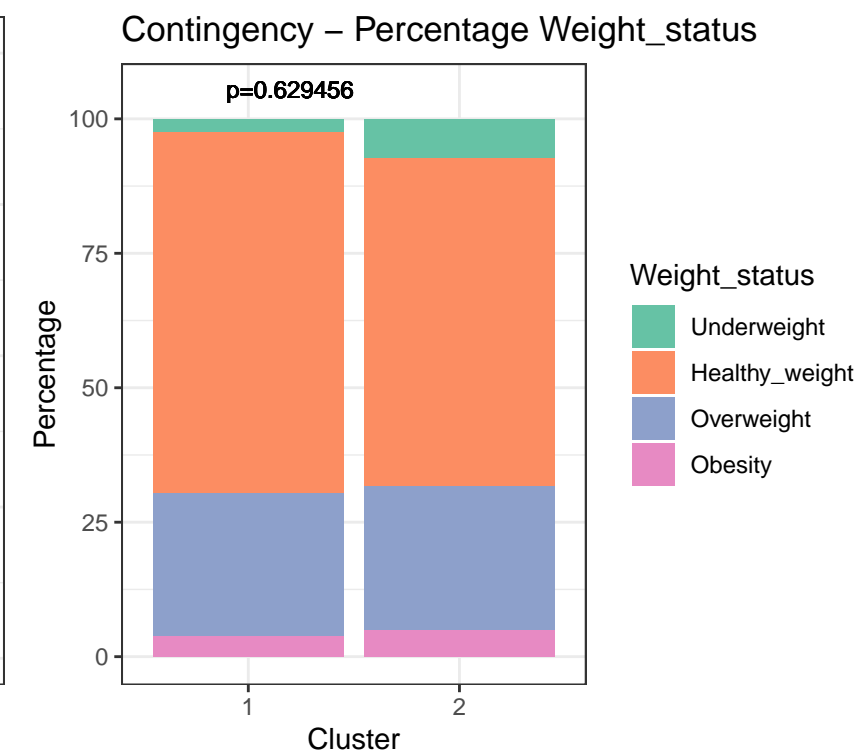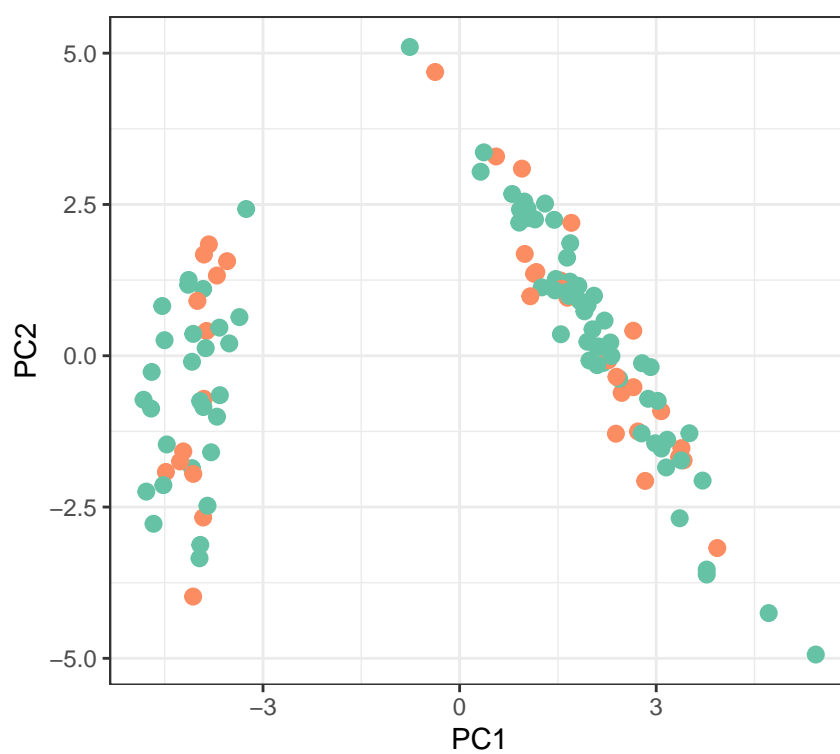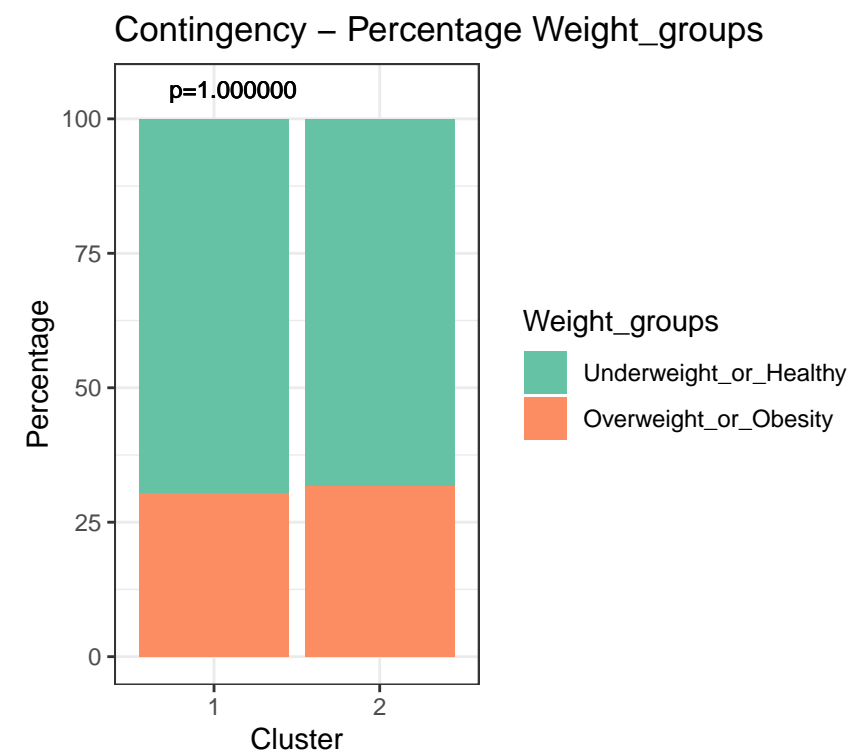

Supplement: Supplementary file 1 [file molecules-29-05169-s001.zip › molecules-3242400-supplementary/S7_all_patients_sphingolipids.pdf]

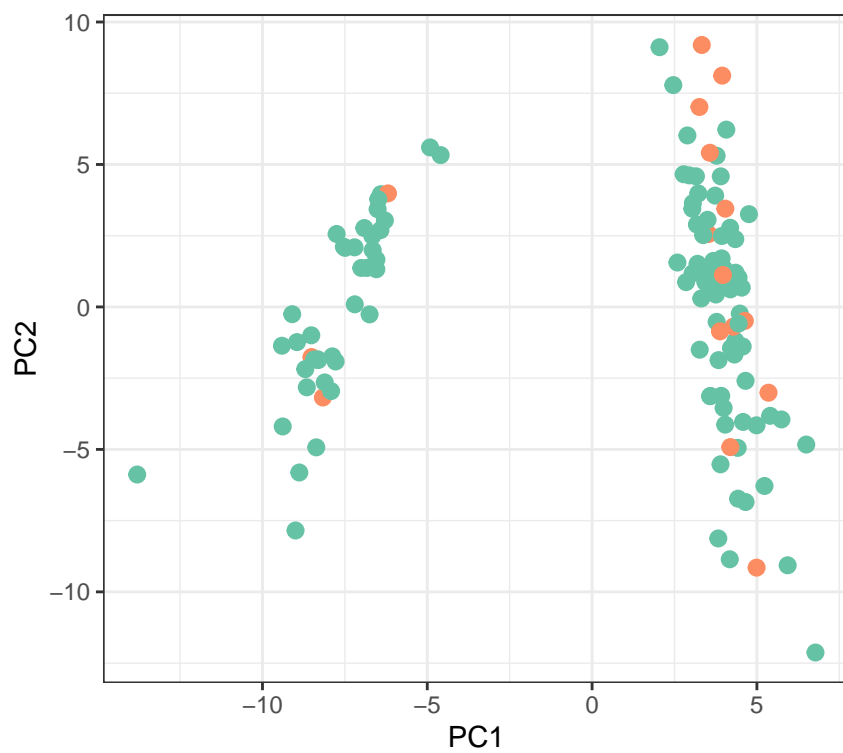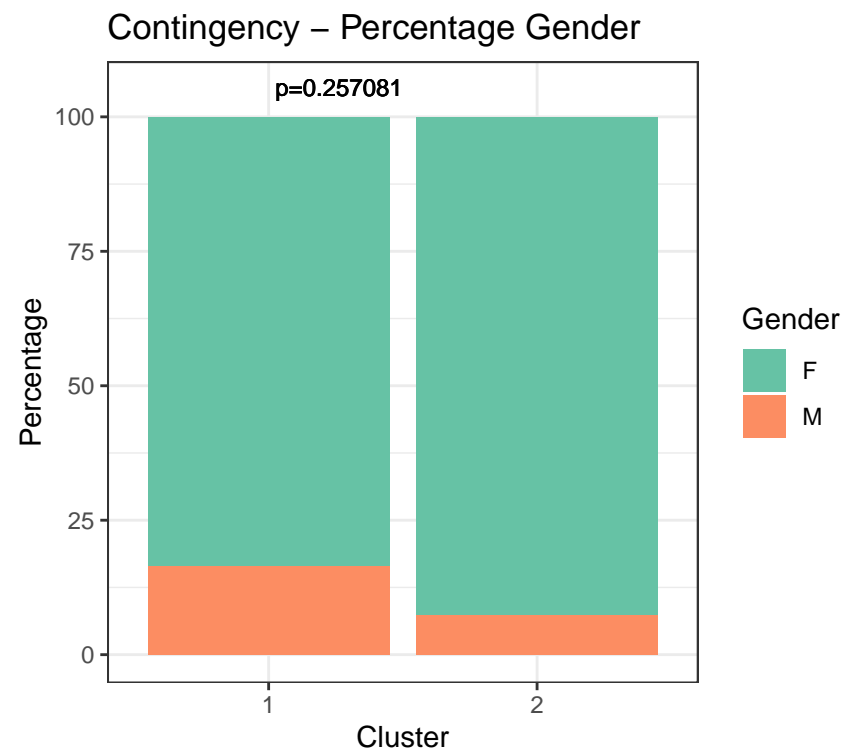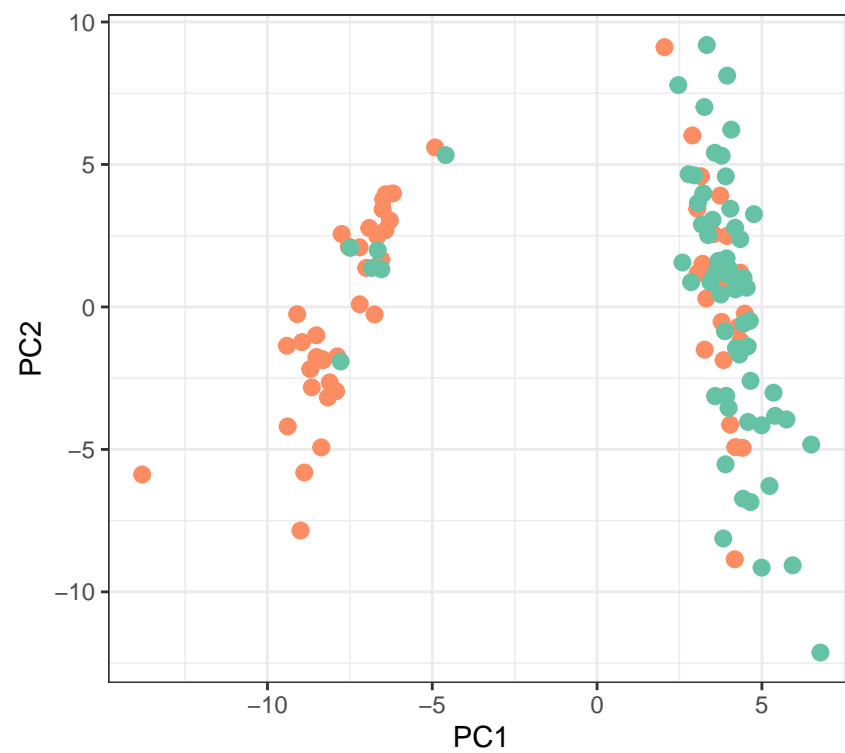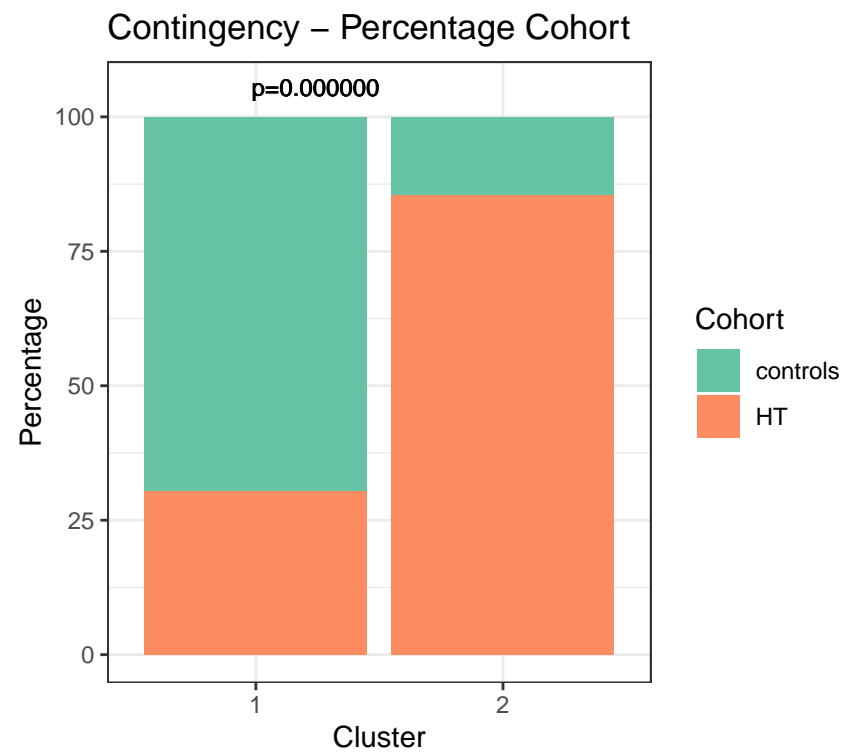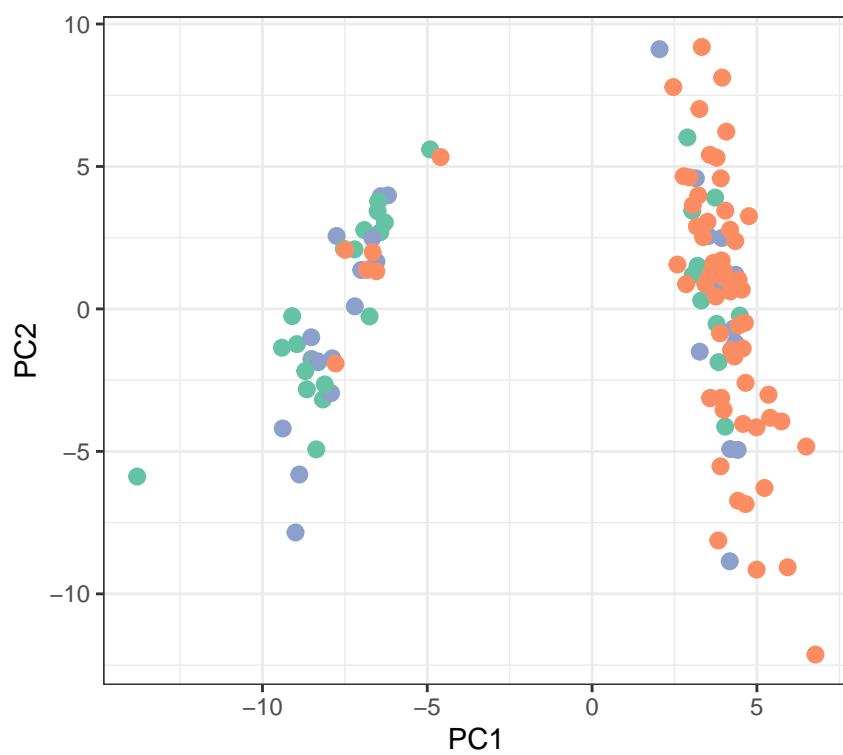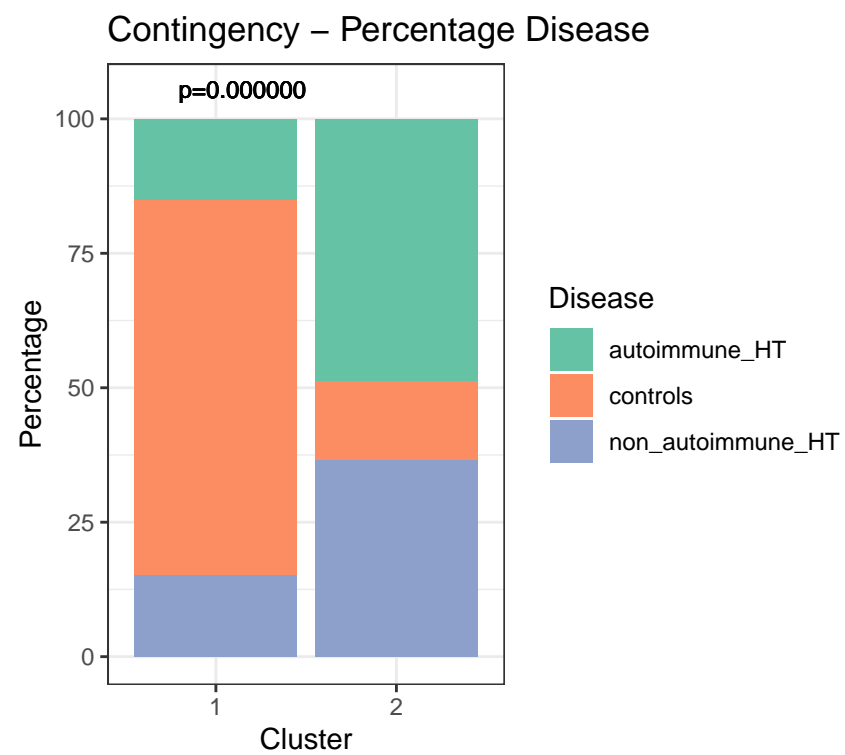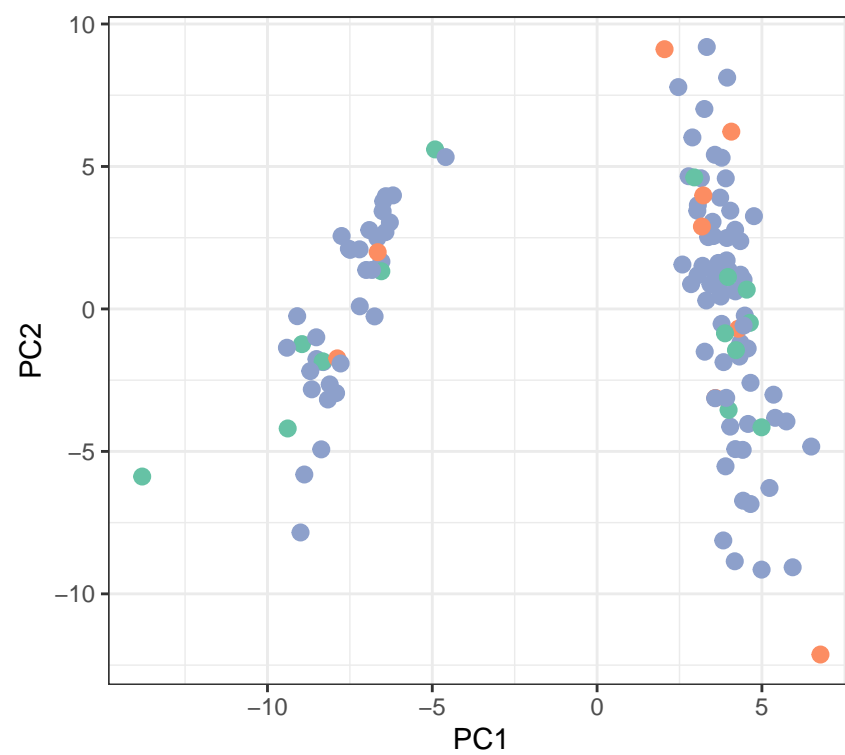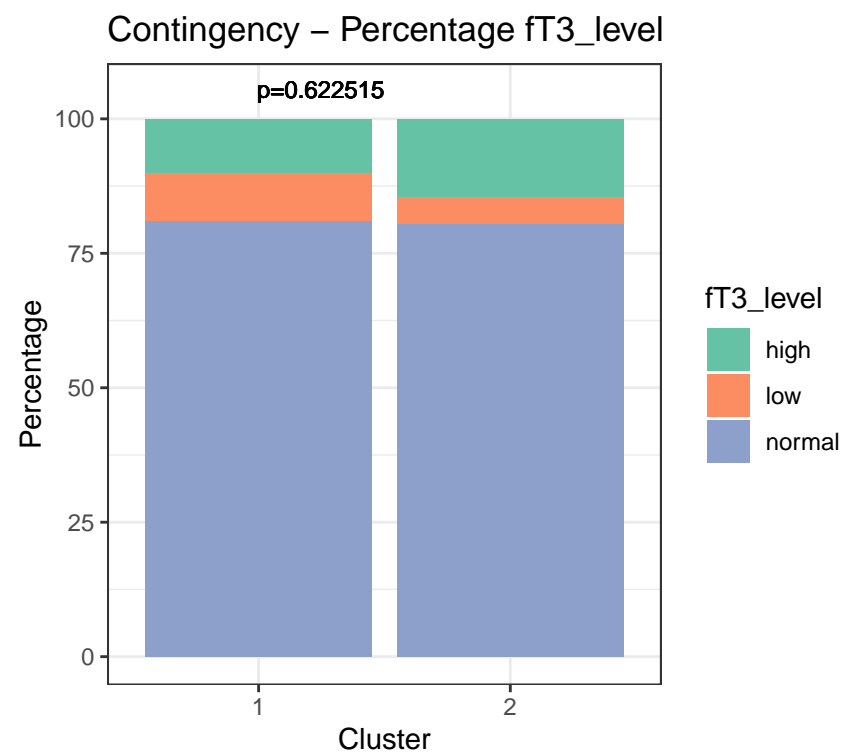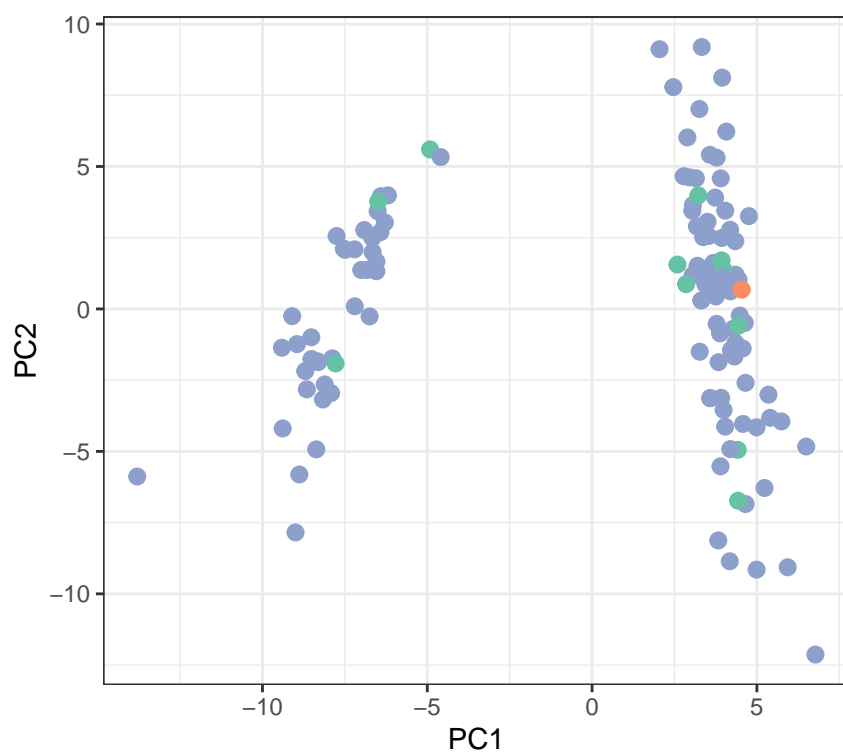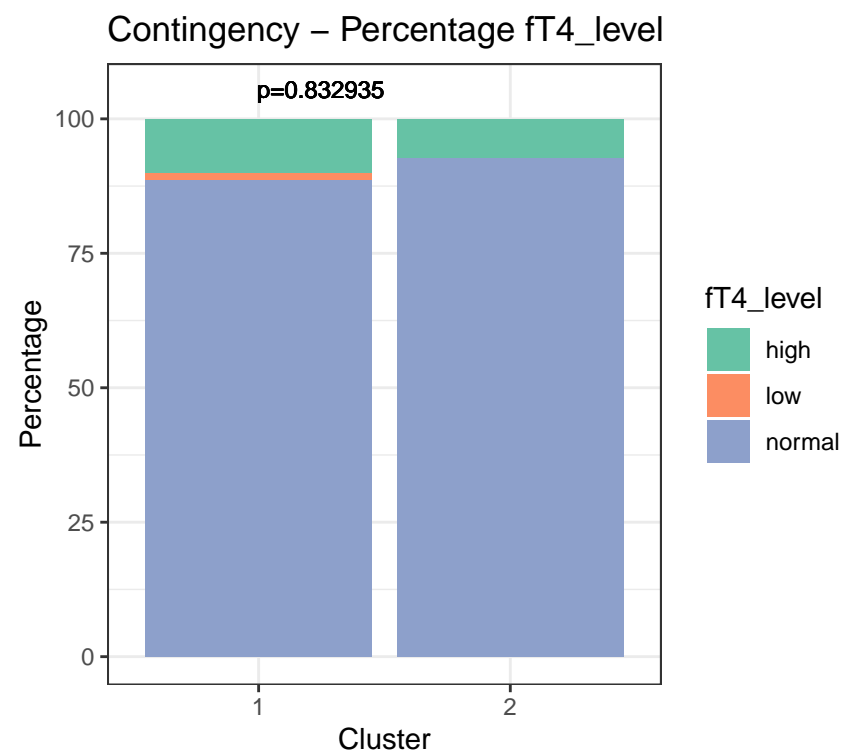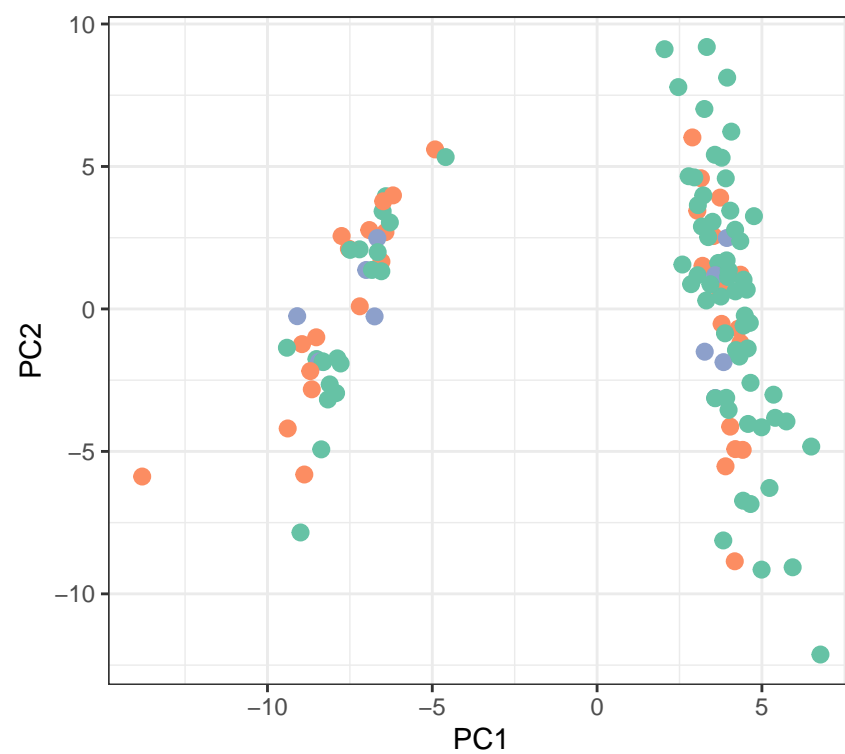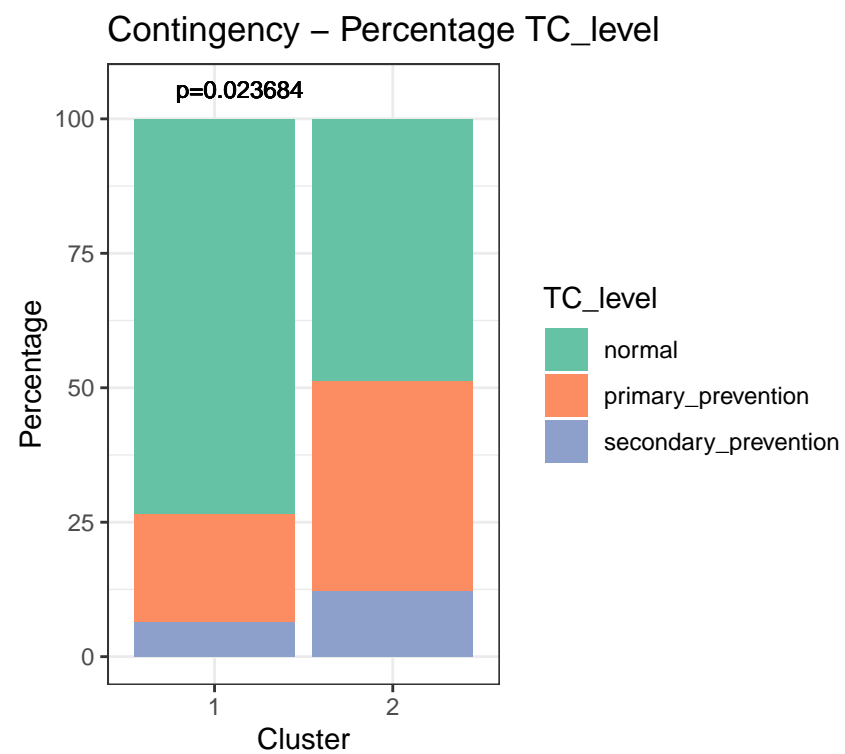

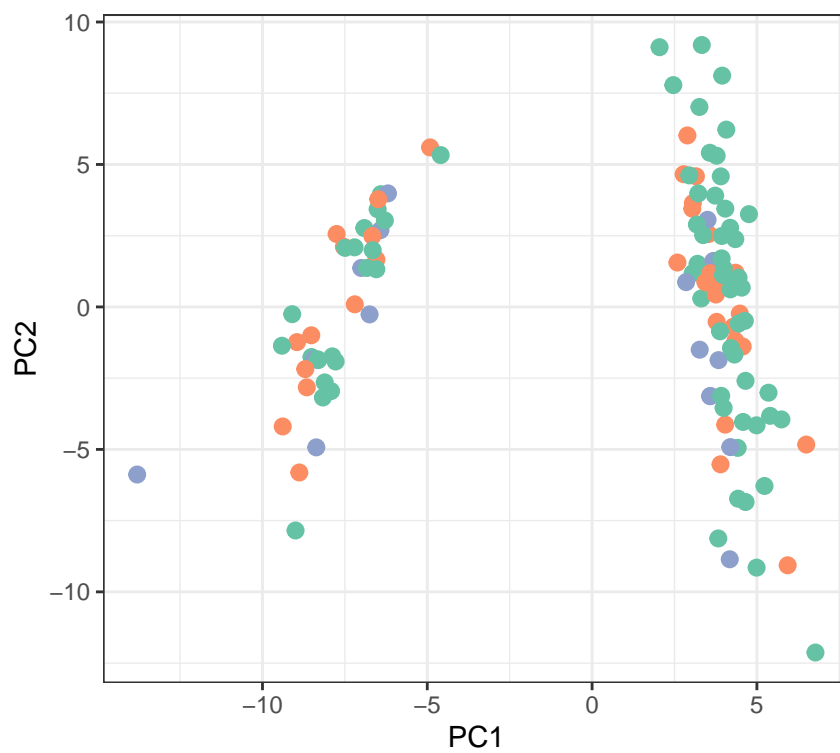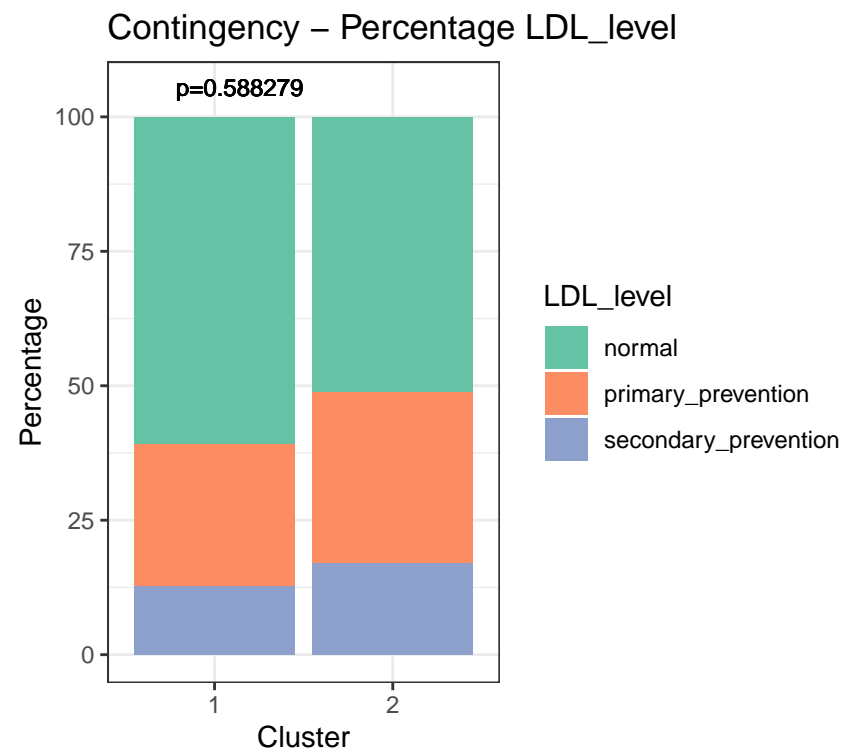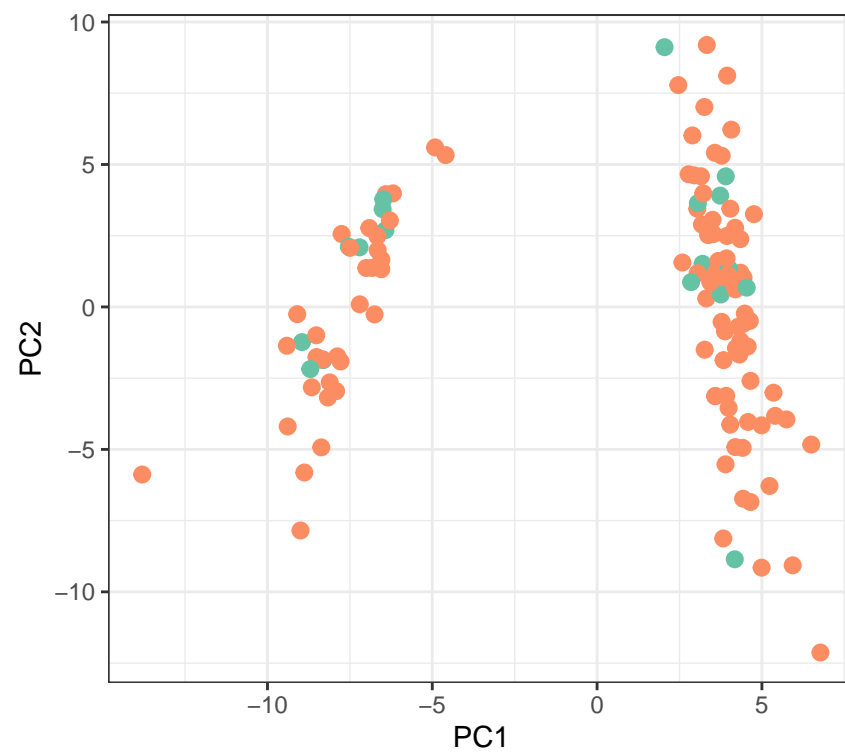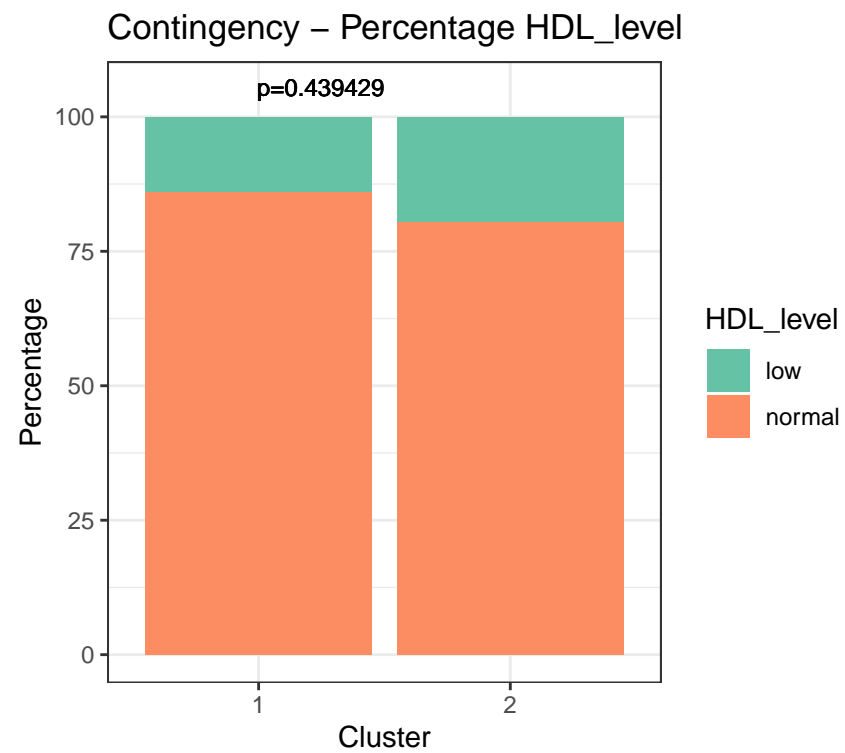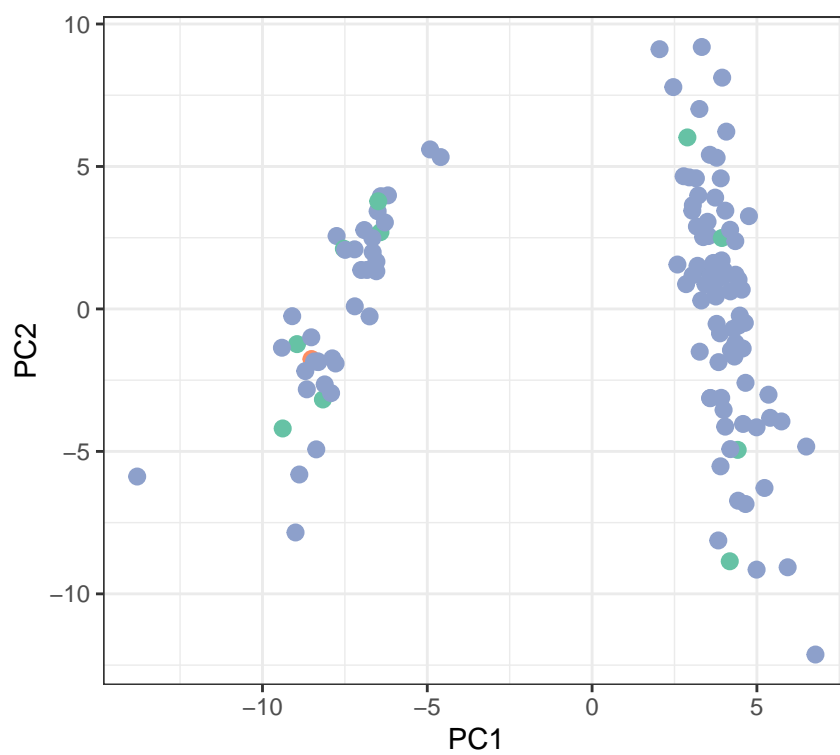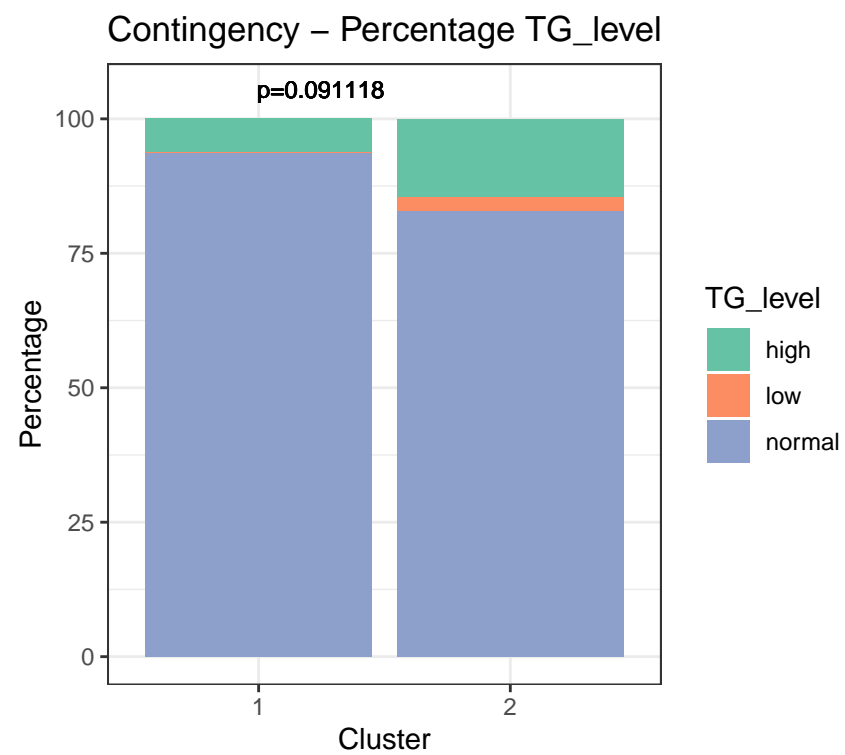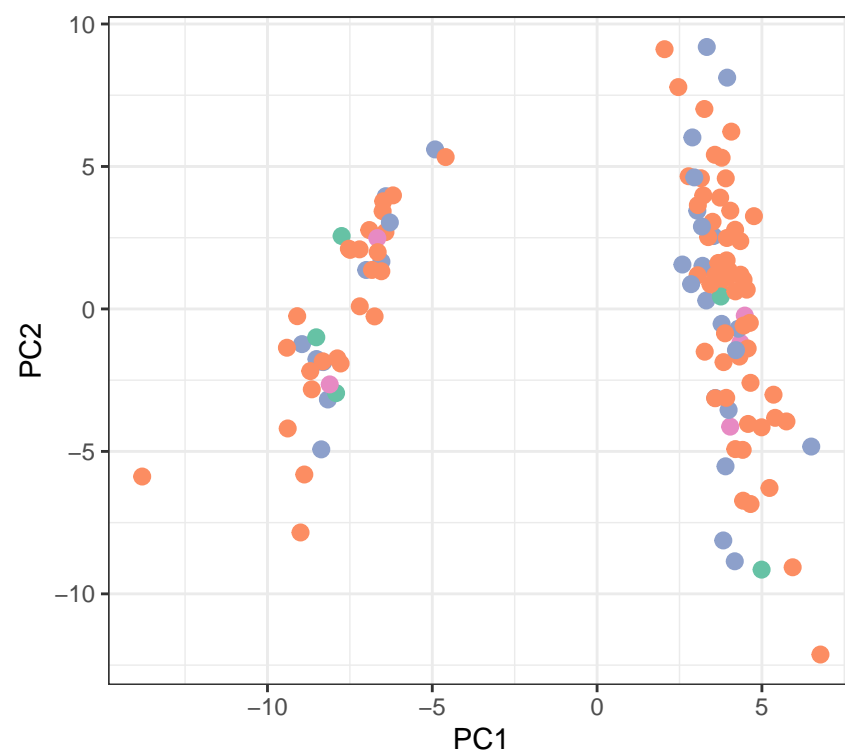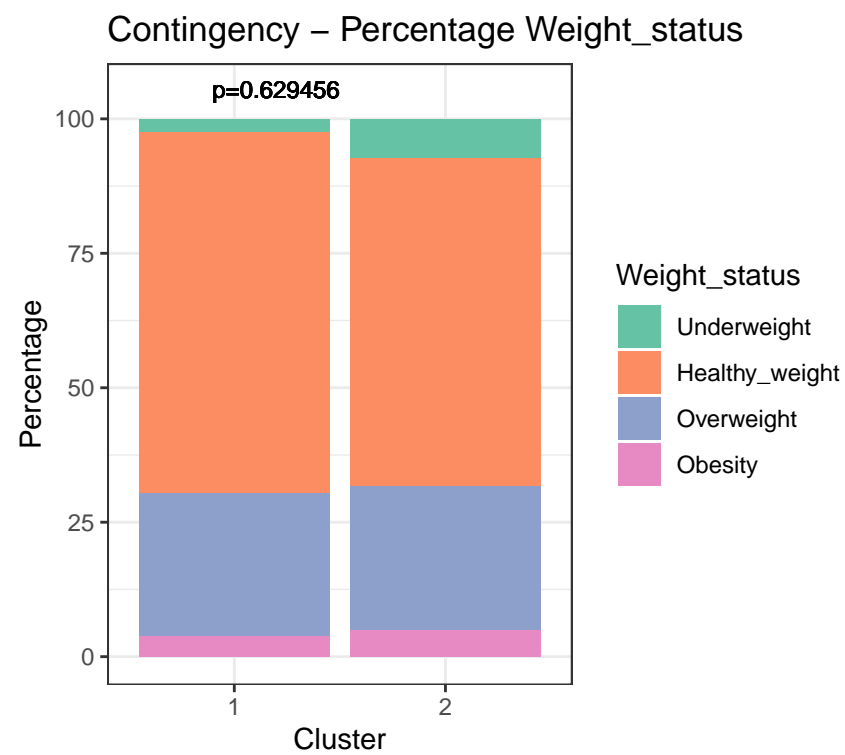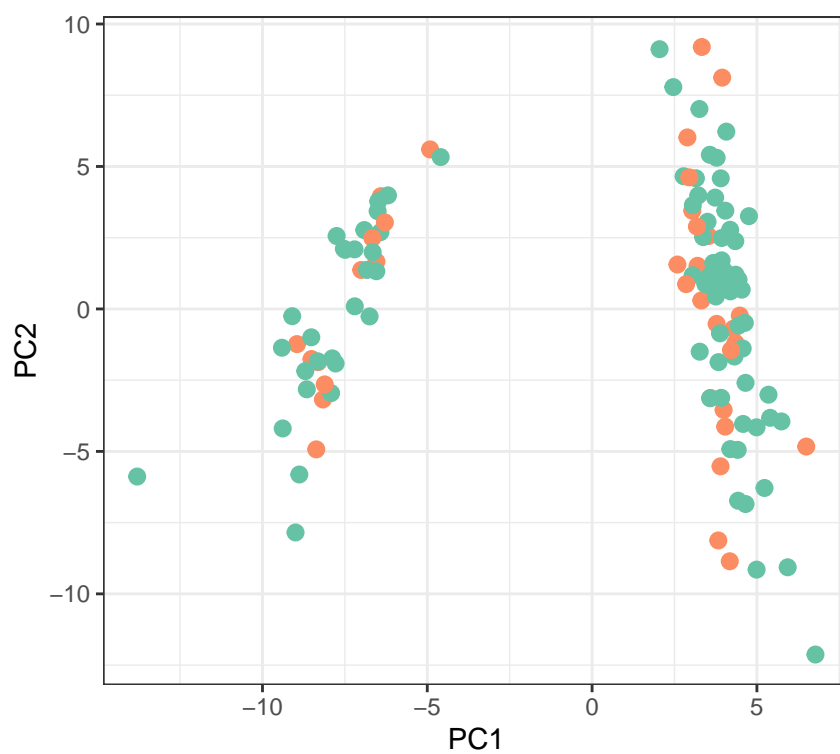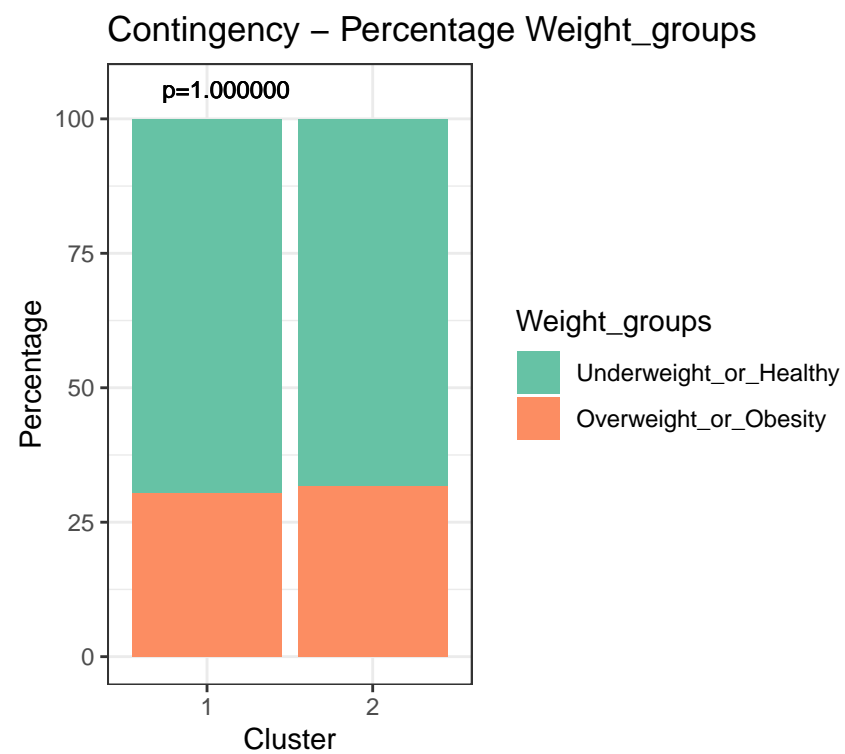

Supplement: Supplementary file 1 [file molecules-29-05169-s001.zip › molecules-3242400-supplementary/S8_all_patients_lipids_toxic_elements.pdf]
