# Supplementary material for: Application of Human Plasma Targeted Lipidomics and Analysis of Toxic Elements to Capture the Metabolic Complexities of Hypothyroidism
Source: Molecules. 2024 Oct 31;29(21):5169. doi: 10.3390/molecules29215169 (PMC11547455; doi:10.3390/molecules29215169)
Supplement: Supplementary file 1 [file F11_filtered_biplot_eig_contrib.pdf › molecules-3242400-supplementary/F11_filtered_biplot_eig_contrib.pdf]

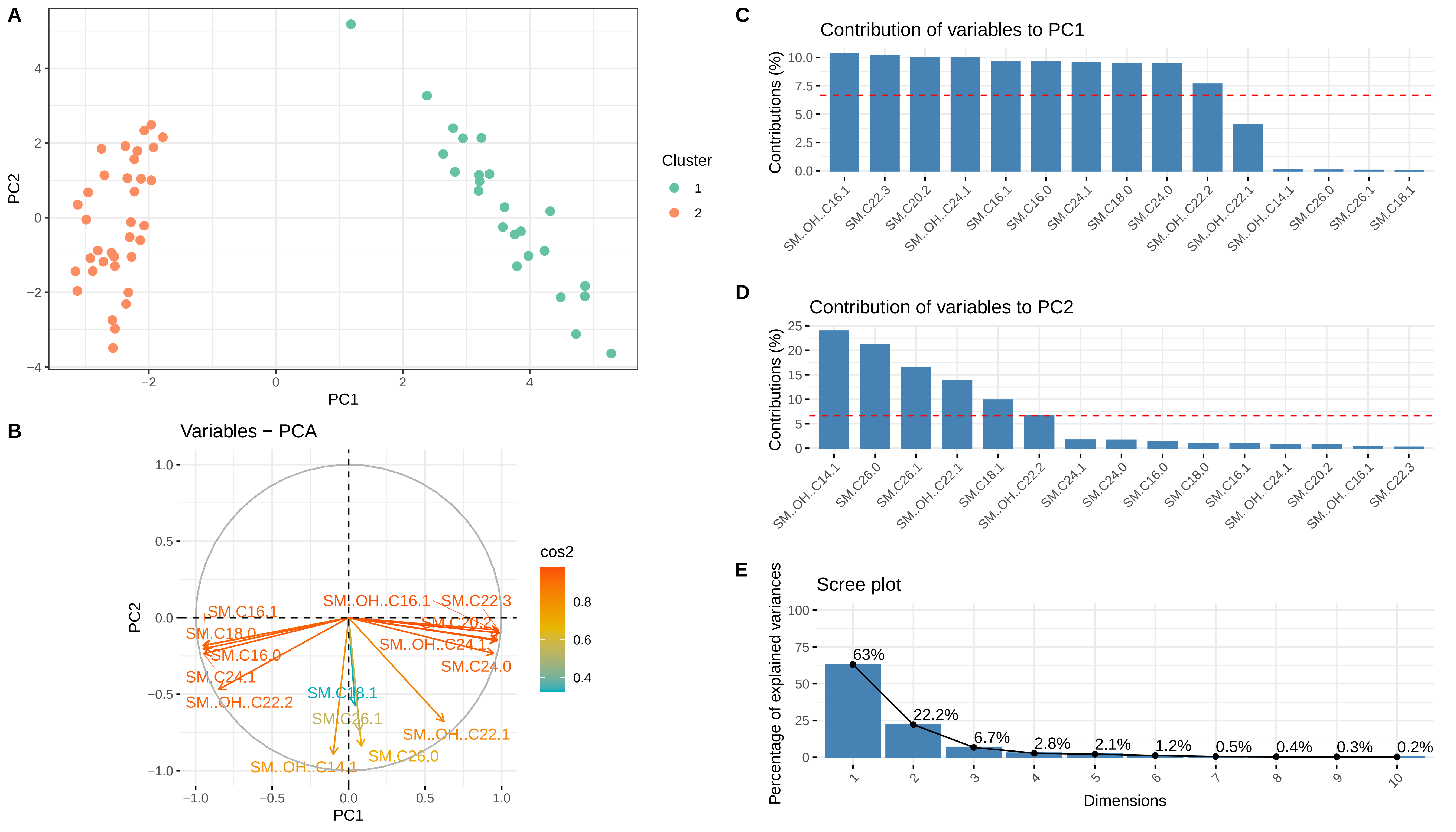
A
PC1
PC2
Cluster
1
2
B
Variables − PCA
cos2
PC1
PC2
SM..OH..C16.1
SM.C22.3
SM.C20.2
SM..OH..C24.1
SM.C24.0
SM.C16.1
SM.C18.0
SM.C16.0
SM.C24.1
SM..OH..C22.2
SM.C18.1
SM.C26.1
SM..OH..C22.1
SM.C26.0
SM..OH..C14.1
C
Contribution of variables to PC1
Contributions (%)
D
Contribution of variables to PC2
Contributions (%)
E
Scree plot
Percentage of explained variances
Dimensions
63%
22.2%
6.7%
2.8%
2.1%
1.2%
0.5%
0.4%
0.3%
0.2%
