# Supplementary material for: Application of Human Plasma Targeted Lipidomics and Analysis of Toxic Elements to Capture the Metabolic Complexities of Hypothyroidism
Source: Molecules. 2024 Oct 31;29(21):5169. doi: 10.3390/molecules29215169 (PMC11547455; doi:10.3390/molecules29215169)
Supplement: Supplementary file 1 [file molecules-29-05169-s001.zip › molecules-3242400-supplementary/Tables S1-S8.zip!/ST1 Nomenclature of lipids.pdf]

Table S1. Nomenclature of lipids.*

Lipid abbreviations used in this study		Lipid abbreviations conformed to LipidMaps standards	
**ACYLCARNITINES**			
C0	Carnitine	–	–
C2	Acetylcarnitine	–	–
C3	Propionylcarnitine	–	–
C3-OH	Hydroxypropionylcarnitine	–	–
C3:1	Propenoylcarnitine	–	–
C4	Butyrylcarnitine	–	–
C4-OH (C3-DC)	Hydroxybutyrylcarnitine (Malonylcarnitine)	–	–
C4:1	Butenylcarnitine	–	–
C5	Valerylcarnitine	–	–
C5-DC (C6-OH)	Glutarylcarnitine (Hydroxyhexanoylcarnitine)	–	–
C5-M-DC	Methylglutarylcarnitine	–	–
C5-OH (C3-DC-M)	Hydroxyvalerylcarnitine (Methylmalonylcarnitine)	–	–
C5:1	Tiglylcarnitine	–	–
C5:1-DC	Glutaconylcarnitine	–	–
C6 (C4:1-DC)	Hexanoylcarnitine (Fumarylcarnitine)	–	–
C6:1	Hexenoylcarnitine	–	–
C7-DC	Pimeloylcarnitine	–	–
C8	Octanoylcarnitine	–	–
C9	Nonaylcarnitine	–	–
C10	Decanoylcarnitine	–	–
C10:1	Decenoylcarnitine	–	–
C10:2	Decadienoylcarnitine	–	–
C12	Dodecanoylcarnitine	–	–
C12-DC	Dodecanedioylcarnitine	–	–
C12:1	Dodecenoylcarnitine	–	–
C14	Tetradecanoylcarnitine	–	–
C14:1	Tetradecenoylcarnitine	–	–
C14:1-OH	Hydroxytetradecenoylcarnitine	–	–
C14:2	Tetradecadienoylcarnitine	–	–
C14:2-OH	Hydroxytetradecadienoyl-carnitine	–	–
C16	Hexadecanoylcarnitine	–	–
C16-OH	Hydroxyhexadecanoylcarnitine	–	–
C16:1	Hexadecenoylcarnitine	–	–
C16:1-OH	Hydroxyhexadecenoylcarnitine	–	–
C16:2	Hexadecadienoylcarnitine	–	–
C16:2-OH	Hydroxyhexadecadienoyl-carnitine	–	–
C18	Octadecanoylcarnitine	–	–
C18:1	Octadecenoylcarnitine	–	–
C18:1-OH	Hydroxyoctadecenoylcarnitine	–	–
C18:2	Octadecadienylcarnitine	–	–
**GLYCEROPHOSPHOLIPIDS**			
lysoPC a C14:0		LPC 14:0	
lysoPC a C16:0		LPC 16:0	
lysoPC a C16:1		LPC 16:1	
lysoPC a C17:0		LPC 17:0	

lysoPC a C18:0	LPC 18:0
lysoPC a C18:1	LPC 18:1
lysoPC a C18:2	LPC 18:2
lysoPC a C20:3	LPC 20:3
lysoPC a C20:4	LPC 20:4
lysoPC a C24:0	LPC 24:0
lysoPC a C26:0	LPC 26:0
lysoPC a C26:1	LPC 26:1
lysoPC a C28:0	PC O-28:0
lysoPC a C28:1	PC O-28:1
PC aa C24:0	PC 24:0
PC aa C26:0	PC 26:0
PC aa C28:1	PC 28:1
PC aa C30:0	PC 30:0
PC aa C30:22	PC 30:22
PC aa C32:0	PC 32:0
PC aa C32:1	PC 32:1
PC aa C32:2	PC 32:2
PC aa C32:3	PC 32:3
PC aa C34:1	PC 34:1
PC aa C34:2	PC 34:2
PC aa C34:3	PC 34:3
PC aa C34:4	PC 34:4
PC aa C36:0	PC 36:0
PC aa C36:1	PC 36:1
PC aa C36:2	PC 36:2
PC aa C36:3	PC 36:3
PC aa C36:4	PC 36:4
PC aa C36:5	PC 36:5
PC aa C36:6	PC 36:6
PC aa C38:0	PC 38:0
PC aa C38:12	PC 38:12
PC aa C38:3	PC 38:3
PC aa C38:4	PC 38:4
PC aa C38:5	PC 38:5
PC aa C38:6	PC 38:6
PC aa C40:1	PC 40:1
PC aa C40:2	PC 40:2
PC aa C40:3	PC 40:3
PC aa C40:4	PC 40:4
PC aa C40:5	PC 40:5
PC aa C40:6	PC 40:6
PC aa C42:0	PC 42:0
PC aa C42:1	PC 42:1
PC aa C42:2	PC 42:2
PC aa C42:4	PC 42:4
PC aa C42:5	PC 42:5
PC aa C42:6	PC 42:6
PC ae C30:0	PC O-30:0
PC ae C30:1	PC O-30:1
PC ae C30:2	PC O-30:2
PC ae C32:1	PC O-32:1
PC ae C32:2	PC O-32:2
PC ae C34:0	PC O-34:0
PC ae C34:1	PC O-34:1
PC ae C34:2	PC O-34:2
PC ae C34:3	PC O-34:3

PC ae C36:0	PC O-36:0
PC ae C36:1	PC O-36:1
PC ae C36:2	PC O-36:2
PC ae C36:3	PC O-36:3
PC ae C36:4	PC O-36:4
PC ae C36:5	PC O-36:5
PC ae C38:0	PC O-38:0
PC ae C38:1	PC O-38:1
PC ae C38:2	PC O-38:2
PC ae C38:3	PC O-38:3
PC ae C38:4	PC O-38:4
PC ae C38:5	PC O-38:5
PC ae C38:6	PC O-38:6
PC ae C40:1	PC O-40:1
PC ae C40:2	PC O-40:2
PC ae C40:3	PC O-40:3
PC ae C40:4	PC O-40:4
PC ae C40:5	PC O-40:5
PC ae C40:6	PC O-40:6
PC ae C42:0	PC O-42:0
PC ae C42:1	PC O-42:1
PC ae C42:2	PC O-42:2
PC ae C42:3	PC O-42:3
PC ae C42:4	PC O-42:4
PC ae C42:5	PC O-42:5
PC ae C44:3	PC O-44:3
PC ae C44:4	PC O-44:4
PC ae C44:5	PC O-44:5
PC ae C44:6	PC O-44:6
**SPHINGOLIPIDS**	
SM (OH) C14:1	SM 33:1
SM C16:0	SM 34:1
SM C16:1	SM 34:2
SM (OH) C16:1	SM 35:1
SM C18:0	SM 36:1
SM C18:1	SM 36:2
SM C20:2	SM 38:3
SM (OH) C22:1	SM 41:1
SM (OH) C22:2	SM 41:2
SM C22:32	SM 40:42
SM C24:0	SM 42:1
SM C24:1	SM 42:2
SM (OH) C24:1	SM 43:1
SM C26:0	SM 44:1
SM C26:1	SM 44:2

*please note that dual nomenclature was used due to technical limitations of the programming environment
