# Supplementary material for: Application of Human Plasma Targeted Lipidomics and Analysis of Toxic Elements to Capture the Metabolic Complexities of Hypothyroidism
Source: Molecules. 2024 Oct 31;29(21):5169. doi: 10.3390/molecules29215169 (PMC11547455; doi:10.3390/molecules29215169)
Supplement: Supplementary file 1 [file molecules-29-05169-s001.zip › molecules-3242400-supplementary/Tables S1-S8.zip!/ST6_raw_data.pdf]

Błażewicz et all Application of human plasma targeted lipidomics and analysis of toxic elements to capture the metabolic complexities of hypothyroidism

ID	Cohort	Disease	C0	C2	C3	C3.DC..C4.OH.	C3.OH	C3.1	C4	C4.1	C5
1	HT	non_autoimmune_HT	61,1	10,6	0,448	0,038	0	0	0,194	0	0,184
2	HT	non_autoimmune_HT	41,8	11,4	0,227	0	0	0	0,164	0	0,088
3	HT	autoimmune_HT	28,7	4,96	0,175	0,065	0	0	0,163	0	0,06
4	HT	autoimmune_HT	54,5	5,81	0,436	0	0	0	0,198	0	0,084
5	HT	autoimmune_HT	35,4	6,04	0,477	0	0	0,013	0,168	0	0,071
6	HT	non_autoimmune_HT	34,7	6,07	0,42	0,05	0	0	0,134	0	0,046
7	HT	autoimmune_HT	16,6	3,51	0,245	0	0	0	0,112	0	0,054
8	HT	autoimmune_HT	24,4	3,89	0,536	0,054	0	0	0,209	0	0,094
9	HT	non_autoimmune_HT	38,2	7,72	0,26	0,054	0	0	0,132	0	0,056
10	HT	autoimmune_HT	42,3	5,34	0,344	0,17	0	0	0,164	0	0,048
11	HT	autoimmune_HT	37	5,67	0,397	0	0	0,014	0,193	0	0,082
12	HT	non_autoimmune_HT	37	6,55	0,567	0,049	0	0	0,234	0	0,129
13	HT	autoimmune_HT	22,2	4,02	0,372	0	0	0,013	0,179	0	0,092
14	HT	autoimmune_HT	31,6	5,78	0,386	0,045	0	0	0,133	0	0,097
15	HT	autoimmune_HT	25,2	4,15	0,331	0	0	0,01	0,192	0	0,086
16	HT	autoimmune_HT	29,4	6,03	0,353	0	0	0,011	0,148	0	0,094
17	HT	non_autoimmune_HT	37	5,9	0,365	0	0	0	0,12	0	0,103
18	HT	autoimmune_HT	22,5	4,77	0,373	0,048	0	0,018	0,217	0,051	0,098
19	HT	non_autoimmune_HT	30,9	7,65	0,408	0,06	0	0	0,219	0	0,076
20	HT	autoimmune_HT	22,8	4,9	0,441	0,047	0	0	0,133	0	0,06
21	HT	autoimmune_HT	36,2	5,43	0,389	0,038	0	0	0,162	0	0,15
22	HT	non_autoimmune_HT	31,8	8,15	0,474	0	0	0,019	0,311	0	0,081
23	HT	non_autoimmune_HT	48,2	5,26	0,451	0,054	0	0	0,204	0	0,099
24	HT	non_autoimmune_HT	37,8	5,03	0,334	0,038	0,014	0	0,243	0	0,136
25	HT	autoimmune_HT	41,8	10,9	0,514	0,063	0	0,017	0,194	0	0,171
26	HT	autoimmune_HT	31	6,53	0,308	0,045	0	0,014	0,161	0	0,055
27	HT	non_autoimmune_HT	37,1	6,37	0,444	0	0	0	0,186	0	0,089
28	HT	non_autoimmune_HT	43,7	4,83	0,407	0	0	0	0,233	0	0,072
29	HT	autoimmune_HT	26,2	3,87	0,284	0	0	0	0,162	0	0,63
30	HT	non_autoimmune_HT	25,8	5,6	0,274	0,056	0	0,013	0,193	0,048	0,079
31	HT	non_autoimmune_HT	37	7,6	0,395	0,039	0	0	0,214	0	0,091
32	HT	autoimmune_HT	16,8	4,63	0,298	0	0,014	0	0,158	0	0,073
33	HT	non_autoimmune_HT	43,4	5,4	0,589	0	0	0,022	0,198	0,048	0,148
34	HT	autoimmune_HT	39,1	4,94	0,373	0	0	0	0,158	0	0,072
35	HT	autoimmune_HT	25,2	2,6	0,197	0	0,019	0	0,148	0	0,055
36	HT	autoimmune_HT	31,2	3,78	0,47	0,024	0,009	0,015	0,151	0	0,011
37	HT	autoimmune_HT	45,8	4,62	0,281	0,029	0	0,008	0,205	0	0,082
38	HT	autoimmune_HT	32,4	12,5	0,249	0,045	0,007	0	0,216	0	0,011
39	HT	autoimmune_HT	41,9	7,6	0,352	0,032	0	0	0,193	0	0,129
40	HT	autoimmune_HT	35,7	7,25	0,412	0,01	0	0	0,266	0,03	0,066
41	HT	autoimmune_HT	29,4	4,28	0,346	0,022	0	0	0,177	0	0,067
42	HT	autoimmune_HT	20,7	3,2	0,192	0,058	0	0	0,173	0	0,038
43	HT	autoimmune_HT	51	10,2	0,465	0,038	0	0	0,207	0	0,155
44	HT	autoimmune_HT	34	5,2	0,311	0,104	0,007	0,008	0,138	0	0
45	HT	non_autoimmune_HT	38,6	6,6	0,295	0,015	0	0	0,16	0	0,013
46	HT	autoimmune_HT	41,5	4,3	0,3	0,026	0	0	0,134	0	0,07

47	HT	autoimmune_HT	18,3	2,57	0,214	0,017	0,007	0	0,16	0	0,031
48	HT	non_autoimmune_HT	35,5	6,16	0,322	0,027	0	0	0,179	0	0,095
49	HT	non_autoimmune_HT	38,7	6,32	0,451	0,053	0,008	0,008	0,154	0	0,005
50	HT	non_autoimmune_HT	24	6,91	0,295	0,026	0,002	0	0,198	0	0,005
51	HT	autoimmune_HT	30,4	7,09	0,182	0,044	0,016	0,008	0,124	0	0,018
52	HT	non_autoimmune_HT	30,8	6,23	0,321	0,038	0	0	0,168	0	0,019
53	HT	non_autoimmune_HT	32,8	9,98	0,335	0,052	0	0,005	0,834	0	0,073
54	HT	non_autoimmune_HT	40,3	5,08	0,392	0,024	0,002	0	0,189	0	0,034
55	HT	non_autoimmune_HT	29,4	6,46	0,347	0,03	0	0	0,206	0	0,005
56	HT	non_autoimmune_HT	34,9	4,69	0,365	0,023	0	0	0,231	0	0,052
57	HT	non_autoimmune_HT	20,5	2,65	0,194	0,027	0	0,004	0,098	0	0,047
58	HT	non_autoimmune_HT	27	5,95	0,39	0,019	0,005	0	0,211	0	0,034
59	HT	non_autoimmune_HT	37,6	7,98	0,271	0,012	0	0	0,32	0,035	0,074
60	controls	controls	32,5	5,63	0,268	0,026	0	0,004	0,204	0	0,62
61	controls	controls	32,4	5,71	0,312	0,013	0	0,008	0,262	0	0,054
62	controls	controls	42,5	4,91	0,338	0	0,002	0	0,178	0	0,03
63	controls	controls	38	5,09	0,525	0,029	0,008	0,002	0,226	0	0,011
64	controls	controls	29,6	3,94	0,251	0,017	0,006	0,007	0,193	0	0,028
65	controls	controls	25,1	6,94	0,237	0,027	0	0,018	0,201	0	0,047
66	controls	controls	38,2	8,85	0,485	0,049	0	0	0,21	0	0,104
67	controls	controls	28,1	4,21	0,334	0,039	0,008	0	0,202	0	0,059
68	controls	controls	27,4	5,66	0,264	0,042	0,008	0	0,149	0	0,014
69	controls	controls	31	3	0,322	0,03	0	0	0,202	0	0,038
70	controls	controls	23,5	4,87	0,142	0,017	0,004	0,001	0,171	0	0
71	controls	controls	38,8	5,13	0,301	0,016	0,01	0	0,158	0	0,126
72	controls	controls	40,1	5,9	0,499	0,024	0	0	0,232	0	0,066
73	controls	controls	42,6	4,97	0,464	0,017	0	0	0,151	0	0,074
74	controls	controls	43,6	4,78	0,457	0,02	0,004	0	0,211	0	0,055
75	controls	controls	34,7	5,94	0,36	0,016	0,001	0,002	0,177	0	0
76	controls	controls	48,3	7,04	0,542	0,052	0	0	0,155	0	0,019
77	controls	controls	20	4,42	0,17	0,031	0	0	0,14	0	0,001
78	controls	controls	56,1	5,86	0,588	0,044	0	0,002	0,199	0	0,089
79	controls	controls	30,7	4,2	0,347	0,018	0	0	0,191	0	0
80	controls	controls	25,3	8,96	0,241	0,019	0	0	0,14	0	0,032
81	controls	controls	36,4	5,54	0,23	0,052	0,003	0,005	0,186	0	0
82	controls	controls	47,9	5,33	0,66	0,018	0	0,006	0,425	0	0,155
83	controls	controls	36,6	4,55	0,289	0,031	0,004	0	0,212	0,003	0,122
84	controls	controls	43,1	6,04	0,365	0,041	0,011	0,013	0,197	0	0,062
85	controls	controls	24,2	4,96	0,202	0,033	0	0	0,348	0	0,077
86	controls	controls	35,6	6,77	0,311	0,02	0	0,007	0,333	0	0,063
87	controls	controls	40,1	5,58	0,572	0,064	0	0	0,158	0	0
88	controls	controls	57,7	8,3	0,59	0,063	0	0,007	0,346	0	0,15
89	controls	controls	55,3	8,5	0,417	0,01	0	0	0,316	0	0,045
90	controls	controls	50	9,75	0,436	0,048	0,005	0,003	0,162	0	0,071
91	controls	controls	25,2	8,62	0,165	0,086	0	0,007	0,085	0	0,013
92	controls	controls	55,4	4,64	0,373	0,059	0,027	0,008	0,25	0	0,151
93	controls	controls	24,1	2,5	0,229	0,038	0,005	0,01	0,373	0	0,377
94	controls	controls	34,9	7,92	0,282	0,068	0,013	0,017	0,339	0	0,109
95	controls	controls	33,7	3,38	0,252	0,038	0	0	0,186	0	0,033
96	controls	controls	37,6	5,75	0,414	0,03	0	0	0,175	0	0

97	controls	controls	32,7	4,25	0,307	0,027	0	0,002	0,202	0	0,06
98	controls	controls	42	6,51	0,44	0,02	0	0	0,255	0	0,124
99	controls	controls	27,2	7,26	0,196	0,052	0,002	0,001	0,117	0	0
100	controls	controls	44,8	11,2	0,508	0,022	0	0,006	0,265	0	0,062
101	controls	controls	33,1	3,32	0,563	0,007	0,019	0	0,186	0	0,168
102	controls	controls	38,1	5,63	0,612	0,067	0	0	0,23	0,006	0,101
103	controls	controls	41,6	4,4	0,288	0,011	0,017	0	0,197	0	0,065
104	controls	controls	48,2	20,6	0,595	0,094	0,035	0,012	0,584	0	0,272
105	controls	controls	37,1	3,83	0,464	0,017	0	0,001	0,178	0	0,118
106	controls	controls	30	3,64	0,183	0,021	0	0,012	0,117	0	0
107	controls	controls	44,3	2,97	0,268	0,031	0,024	0	0,144	0	0,228
108	controls	controls	49	8,56	0,385	0,038	0,019	0	0,272	0	0,05
109	controls	controls	32,4	4,59	0,224	0,085	0,019	0	0,194	0	0
110	controls	controls	32,4	6,16	0,342	0,033	0,013	0,01	0,136	0	0
111	controls	controls	56,7	5,67	0,469	0,005	0	0	0,154	0	0
112	controls	controls	26,2	3,83	0,231	0,029	0,002	0	0,169	0	0
113	controls	controls	57,2	5,12	0,243	0,043	0,01	0	0,248	0	0,076
114	controls	controls	25,9	5,38	0,234	0,006	0	0	0,363	0	0,076
115	controls	controls	39,3	3,69	0,568	0	0	0	0,257	0	0,091
116	controls	controls	46,9	5,03	0,353	0	0	0,012	0,192	0	0,08
117	controls	controls	29,7	9,82	0,379	0,065	0	0	0,892	0	0,099
118	controls	controls	51,4	8,64	0,431	0,045	0	0	0,203	0	0,187
119	controls	controls	37,6	3,78	0,321	0	0,014	0	0,122	0	0,072
120	controls	controls	18,7	4,44	0,156	0	0	0,014	0,109	0	0,048

C5.DC..C6.OH.	C5.M.DC	C5.OH..C3.DC.M.	C5.1	C5.1.DC	C6..C4.1.DC.	C6.1	C7.DC	C8	C9	C10
0	0	0	0	0	0	0	0,059	0,434	0,041	0,359
0,045	0	0	0	0	0	0	0	0,752	0	0,519
0	0	0	0	0,032	0	0	0	3,11	0	1,4
0	0	0	0,053	0	0	0	0	0,131	0	0,226
0,045	0	0	0	0,029	0,101	0	0	0,259	0	0,561
0	0	0	0,062	0,042	0	0	0	0	0	0,265
0	0	0	0	0	0	0	0	0	0	0,141
0	0	0	0	0	0	0	0,037	0,145	0	0,304
0	0	0	0	0	0	0	0	0,09	0	0,207
0	0	0	0	0	0,103	0	0	0,175	0	0,366
0	0	0	0	0,03	0	0	0	0	0	0,144
0	0	0	0	0,033	0,102	0	0	0,113	0	0,398
0	0	0	0	0,034	0	0	0	0	0	0,214
0	0	0	0,059	0	0	0	0,038	0,163	0	0,303
0	0	0	0	0	0	0	0	0	0	0,147
0	0	0	0	0,033	0	0	0	0,074	0	0,157
0	0	0	0	0	0	0	0	0	0	0,109
0,049	0	0	0	0	0	0	0	0	0	0,242
0	0	0	0,063	0	0	0	0	0,108	0	0,272
0	0	0	0	0	0,114	0	0,038	0,303	0	0,568
0	0	0	0,053	0	0	0	0	0,091	0	0,304
0	0	0	0	0,038	0,094	0	0,048	0,139	0	0,442
0	0	0	0	0,041	0	0	0	0	0,048	0,175
0	0	0	0	0,029	0	0	0	0,099	0,042	0,3
0	0	0	0,048	0,037	0	0,046	0,052	0,095	0	0,374
0	0	0	0	0	0	0	0	0,12	0	0,336
0	0	0	0,052	0	0	0	0	0,115	0	0,251
0	0	0	0	0	0	0	0	0	0	0,162
0	0	0	0,076	0	0	0	0,038	0,15	0,041	0,367
0	0	0	0	0	0	0	0	0,102	0	0,238
0,046	0	0	0	0,042	0,264	0	0	0,98	0,043	1,58
0,042	0	0	0	0,029	0,117	0	0	0,254	0	0,541
0,04	0	0	0	0,04	0,099	0	0,042	0	0	0,206
0	0	0	0	0	0	0	0	0	0	0,161
0	0	0	0	0	0	0	0	0	0	0,109
0,023	0,008	0,021	0,009	0	0,039	0,007	0,01	0,144	0,032	0,181
0,024	0,016	0,015	0,016	0	0,064	0,023	0,025	0,509	0,023	0,385
0,014	0	0,014	0	0	0,089	0,002	0,034	4,86	0,014	2,19
0,018	0,018	0,004	0,015	0,02	0,125	0,014	0,044	0,452	0,026	0,774
0,004	0,006	0,022	0	0,019	0,201	0,027	0,031	0,974	0,045	1,6
0,02	0,012	0,009	0,007	0	0,067	0,014	0,008	0,185	0,032	0,23
0,014	0	0,016	0,018	0,006	0,051	0	0,014	0,139	0,02	0,158
0,027	0,015	0,037	0	0,014	0,058	0	0,047	0,771	0,031	0,489
0,02	0,026	0	0,015	0	0,119	0,025	0,02	0,265	0,023	0,356
0,024	0,03	0,02	0,001	0,019	0,072	0,019	0,024	0,151	0,006	0,172
0,028	0,031	0,019	0,007	0,006	0,075	0,008	0,039	0,222	0,04	0,308

0,007	0,031	0	0	0	0,043	0,003	0,006	0,123	0,024	0,11
0,001	0,012	0,016	0	0	0,054	0	0,001	0,085	0,017	0,099
0,022	0,007	0,022	0	0,003	0,077	0,013	0,033	0,183	0,02	0,293
0,021	0,031	0,015	0,005	0,015	0,153	0,022	0,02	0,361	0,018	0,56
0,023	0,009	0,015	0,004	0,007	0,075	0	0,009	0,146	0,02	0,197
0	0,012	0,005	0,016	0,013	0,078	0,006	0,029	0,212	0,021	0,316
0,035	0,027	0,024	0,009	0,003	0,102	0,014	0,057	0,358	0,02	0,55
0,017	0,022	0,007	0	0	0,081	0,001	0,018	0,163	0,043	0,196
0,023	0,031	0,037	0,012	0,004	0,069	0	0,016	0,176	0,026	0,259
0,006	0	0,027	0	0	0,074	0,023	0,012	0,147	0,013	0,157
0,004	0,013	0	0,019	0,001	0,053	0,013	0,011	0,092	0,01	0,077
0,024	0,026	0,005	0	0,014	0,089	0,005	0,029	0,238	0,031	0,324
0,031	0,013	0,01	0	0	0,089	0	0,029	0,289	0,017	0,432
0,005	0,012	0,017	0	0,009	0,077	0,01	0,044	0,253	0,037	0,397
0,012	0,008	0,006	0	0	0,082	0	0,039	0,229	0,054	0,329
0,012	0,027	0,021	0,003	0,009	0,051	0,006	0,026	0,153	0,026	0,186
0	0,01	0,016	0	0	0,09	0	0,014	0,202	0,029	0,265
0,016	0,005	0,012	0	0,012	0,076	0,001	0,025	0,176	0,026	0,218
0,014	0,001	0,025	0,002	0,004	0,13	0,002	0,027	0,425	0,018	0,661
0,027	0,035	0	0	0,018	0,082	0,027	0,02	0,213	0,015	0,333
0,017	0,021	0	0	0,02	0,069	0,015	0,013	0,134	0,032	0,16
0,017	0,019	0,017	0	0	0,074	0	0,024	0,193	0,026	0,26
0	0	0,018	0,026	0	0,032	0	0,018	0,106	0,021	0,11
0,012	0,017	0,016	0,012	0,013	0,086	0,003	0,03	0,363	0,027	0,503
0,012	0,01	0,013	0,009	0,007	0,071	0,026	0,013	0,231	0,028	0,282
0,036	0	0,031	0	0,002	0,078	0	0,013	0,262	0,023	0,348
0	0,03	0,022	0,005	0	0,026	0	0,033	1,27	0,021	0,683
0,001	0,015	0,004	0,007	0,012	0,054	0,004	0,013	0,175	0,03	0,174
0,038	0,019	0,021	0,005	0	0,066	0,012	0,022	0,205	0,022	0,286
0,016	0,005	0,015	0	0,033	0,072	0	0,027	0,164	0,021	0,262
0,023	0,035	0,005	0	0,009	0,049	0,002	0,03	0,171	0,025	0,221
0,02	0,049	0,006	0,015	0	0,055	0,021	0,02	0,11	0,007	0,126
0,02	0,007	0	0,021	0,017	0,066	0,013	0,022	0,21	0,033	0,215
0,032	0,013	0	0	0,014	0,084	0,022	0,03	0,191	0,023	0,264
0,027	0,038	0,013	0	0,022	0,07	0,021	0,011	0,142	0,023	0,211
0,018	0,024	0,011	0	0	0,069	0	0,026	0,128	0,051	0,161
0,02	0,014	0,026	0	0	0,056	0,013	0,033	0,132	0,02	0,171
0,02	0,039	0,029	0,031	0,009	0,047	0	0,012	0,17	0,036	0,186
0,009	0	0,015	0,019	0	0,065	0	0,04	0,13	0,021	0,199
0,003	0,022	0,015	0,01	0,006	0,144	0	0,028	0,473	0,033	0,591
0,012	0	0,007	0	0,01	0,069	0,016	0,026	0,196	0,024	0,251
0,007	0,006	0,009	0,003	0	0,097	0,001	0,018	0,231	0,01	0,247
0,033	0	0,003	0	0,023	0,092	0,007	0,025	0,237	0,013	0,27
0,024	0,044	0,015	0	0,016	0,069	0,012	0,014	0,199	0,012	0,195
0,033	0,015	0	0	0	0,117	0	0,009	0,116	0,015	0,114
0,029	0,015	0,005	0	0	0,08	0	0,016	0,136	0	0,135
0,029	0,01	0,026	0,008	0	0,039	0,014	0,01	0,132	0	0,077
0,017	0,023	0,019	0	0	0,098	0,013	0,009	0,213	0,023	0,192
0,029	0,014	0,017	0	0,006	0,062	0,004	0,02	0,17	0,008	0,223
0,015	0,016	0,016	0,013	0,02	0,079	0	0,019	0,18	0,026	0,224

0	0,022	0,027	0,018	0,035	0,081	0,023	0,023	0,235	0,053	0,347
0,018	0,011	0,037	0	0,039	0,086	0,006	0,021	0,191	0,01	0,196
0,033	0,021	0	0	0,007	0,074	0,001	0,002	0,175	0,021	0,251
0,022	0,039	0,02	0	0,024	0,103	0	0,039	0,259	0,029	0,317
0,013	0,018	0	0,013	0	0,064	0,016	0,011	0,125	0,019	0,147
0,038	0,017	0,035	0,007	0	0,057	0	0,017	0,119	0,028	0,12
0,015	0,031	0,026	0	0,011	0,062	0,013	0,014	0,169	0,011	0,224
0,032	0,052	0,048	0,003	0,049	0,089	0,021	0,032	0,123	0,012	0,143
0,032	0,018	0	0	0,015	0,093	0,021	0,025	0,146	0,026	0,218
0,02	0,032	0,019	0	0	0,064	0,014	0,015	0,135	0,016	0,111
0,001	0,022	0,028	0	0	0,049	0	0,022	0,147	0,017	0,096
0,028	0,01	0	0,018	0	0,105	0,017	0,028	0,18	0,017	0,244
0,027	0,059	0,02	0,009	0,01	0,093	0,002	0,015	0,144	0,012	0,103
0,029	0,002	0	0,002	0,003	0,073	0,005	0,008	0,17	0,034	0,209
0	0,011	0,023	0	0	0,059	0,019	0,034	0,134	0,017	0,146
0,018	0,018	0,004	0	0,026	0,056	0,019	0,035	0,166	0,02	0,127
0,011	0,02	0,012	0,026	0,026	0,066	0,006	0,019	0,205	0,012	0,137
0,019	0	0	0	0,022	0,089	0,005	0,046	0,182	0,029	0,22
0	0	0	0	0	0	0	0	0	0	0,125
0	0	0	0	0	0	0	0,038	0,179	0	0,275
0	0	0	0	0,122	0,122	0	0,064	0,264	0,036	0,558
0	0	0	0,058	0,17	0,17	0	0,06	0,423	0,037	0,833
0	0	0	0,057	0	0	0	0,039	0	0,04	0,23
0	0	0	0	0,033	0	0	0,036	0	0	0,197

C10.1	C10.2	C12	C12.DC	C12.1	C14	C14.1	C14.1.OH	C14.2	C14.2.OH	C16	C16.OH	C16.1	C16.1.OH
0,125	0	0,125	0	0,133	0	0,108	0,02	0	0	0,178	0	0,04	0
0	0	0,079	0	0	0	0,067	0	0	0	0,176	0	0	0
0	0	0,081	0	0	0	0,11	0	0	0	0,127	0	0	0
0	0	0,078	0	0	0	0,068	0	0	0	0,136	0	0	0
0,159	0	0,118	0	0,123	0	0,113	0	0	0	0,141	0	0	0
0,105	0	0,103	0	0,12	0	0,098	0,019	0	0	0,16	0	0	0
0	0	0	0	0	0	0,076	0	0	0	0,095	0	0	0
0,143	0,035	0,102	0	0,16	0	0,106	0	0	0	0,13	0	0,041	0
0,127	0	0,076	0	0	0	0,137	0	0	0	0,122	0	0	0
0,134	0	0,092	0	0,118	0	0,161	0	0	0	0,106	0	0	0
0	0	0,071	0	0	0	0,069	0	0	0	0	0	0	0
0,122	0	0,103	0	0	0	0,081	0,018	0	0	0,109	0	0	0
0,123	0,038	0	0	0,119	0	0,089	0	0	0	0,097	0	0	0
0,107	0,045	0,091	0	0,135	0	0,154	0	0	0	0,124	0	0,043	0
0	0	0	0	0	0	0,053	0	0	0	0,114	0	0	0
0	0	0,072	0	0	0	0,112	0	0	0	0,143	0	0,043	0
0	0,036	0	0	0	0	0,061	0	0	0	0,144	0	0	0
0	0	0,098	0	0	0	0,081	0	0,08	0	0,179	0	0	0
0	0	0,097	0	0	0	0,091	0	0,063	0	0,193	0	0,039	0
0,198	0	0,151	0	0,187	0	0,171	0	0,062	0	0,112	0	0,038	0
0,155	0,036	0,078	0	0	0	0,099	0	0	0	0,131	0	0	0
0,143	0	0,142	0	0,167	0	0,131	0,018	0,07	0	0,143	0	0,046	0
0	0	0,07	0	0	0	0,065	0	0,068	0	0,155	0	0	0
0,105	0	0,125	0	0,117	0	0,105	0	0,092	0	0,149	0	0	0
0,127	0,043	0,164	0	0,142	0	0,142	0	0,125	0	0,126	0	0,043	0
0,127	0	0,116	0	0,14	0	0,133	0	0,077	0	0,128	0	0,039	0
0,107	0	0,084	0	0	0	0,074	0	0,075	0	0,142	0	0,041	0
0	0	0	0	0	0	0,05	0	0	0	0,12	0	0	0
0,159	0,044	0,14	0	0,157	0	0,127	0,019	0,061	0	0,14	0	0,038	0
0	0	0,073	0	0	0	0,082	0	0	0	0,15	0	0	0
0,408	0,077	0,258	0	0,236	0	0,192	0,025	0,111	0,011	0,129	0	0,039	0
0,162	0,04	0,117	0	0,134	0	0,121	0	0,098	0	0,136	0	0	0
0	0,044	0,072	0	0	0	0,095	0,017	0,119	0	0,163	0	0	0
0	0	0,102	0	0	0	0,107	0	0,1	0	0,135	0	0	0
0	0	0	0	0	0	0,046	0	0	0	0,091	0	0	0
0,135	0,039	0,073	0,027	0,092	0,026	0,057	0,008	0,026	0,008	0,037	0	0,018	0,002
0,143	0,045	0,093	0,046	0,105	0,035	0,061	0,008	0,027	0,003	0,088	0,005	0,028	0,002
0,097	0,018	0,093	0,041	0,1	0,045	0,084	0,009	0,018	0	0,092	0,004	0,03	0
0,251	0,045	0,198	0,041	0,185	0,053	0,188	0,017	0,057	0,008	0,122	0,004	0,04	0,01
0,413	0,073	0,235	0,039	0,242	0,058	0,187	0,012	0,071	0,006	0,093	0,004	0,035	0,005
0,113	0,044	0,074	0,043	0,106	0,033	0,06	0,006	0,02	0,005	0,07	0,005	0,021	0,001
0,071	0,026	0,055	0,037	0,069	0,015	0,039	0,008	0,014	0	0,035	0,003	0,025	0,004
0,101	0,023	0,125	0,036	0,101	0,033	0,094	0,01	0,022	0,007	0,144	0,006	0,032	0,011
0,136	0,025	0,096	0,038	0,117	0,039	0,103	0,009	0,026	0,003	0,066	0	0,028	0,008
0,097	0,024	0,074	0,035	0,102	0,036	0,099	0,012	0,02	0	0,109	0,001	0,03	0,004
0,123	0,024	0,116	0,038	0,125	0,044	0,098	0,01	0,033	0,006	0,092	0	0,032	0,01

0,065	0,025	0,05	0,038	0,076	0,018	0,095	0,005	0,009	0,004	0,071	0,003	0,02	0,008
0,037	0,029	0,045	0,033	0,054	0,041	0,065	0,008	0,009	0,001	0,096	0	0,02	0,01
0,107	0,017	0,118	0,034	0,123	0,041	0,088	0,01	0,017	0	0,125	0,002	0,032	0,012
0,182	0,04	0,114	0,036	0,14	0,04	0,109	0,009	0,033	0,004	0,092	0	0,03	0,008
0,113	0,027	0,075	0,025	0,088	0,039	0,103	0,011	0,023	0,004	0,075	0,002	0,03	0,01
0,143	0,027	0,113	0,032	0,154	0,042	0,159	0,01	0,033	0	0,08	0,003	0,034	0,009
0,213	0,026	0,191	0,038	0,228	0,067	0,213	0,022	0,061	0,001	0,137	0,007	0,062	0,015
0,075	0,018	0,072	0,039	0,084	0,041	0,061	0,008	0,017	0,002	0,101	0,002	0,02	0,004
0,093	0,019	0,085	0,039	0,088	0,039	0,096	0,011	0,016	0,003	0,151	0,005	0,032	0,008
0,069	0,028	0,061	0,04	0,07	0,031	0,05	0,006	0,009	0,004	0,084	0,002	0,017	0,006
0,036	0,016	0,042	0,045	0,046	0,016	0,024	0,003	0,005	0,003	0,045	0,003	0,025	0,007
0,127	0,02	0,115	0,039	0,142	0,043	0,092	0,011	0,027	0,008	0,109	0,007	0,037	0,008
0,135	0,031	0,129	0,042	0,153	0,049	0,125	0,005	0,037	0,005	0,095	0,003	0,036	0,005
0,157	0,023	0,14	0,04	0,168	0,05	0,125	0,016	0,032	0,007	0,108	0	0,039	0,008
0,127	0,024	0,123	0,037	0,14	0,048	0,118	0,009	0,03	0,007	0,125	0,002	0,025	0,009
0,083	0,022	0,101	0,04	0,103	0,042	0,09	0,009	0,019	0,004	0,114	0,002	0,025	0,008
0,091	0,017	0,087	0,032	0,088	0,042	0,07	0,008	0,017	0,004	0,109	0,003	0,037	0,007
0,082	0,02	0,09	0,039	0,093	0,032	0,075	0,002	0,017	0,007	0,105	0,006	0,024	0,001
0,188	0,037	0,184	0,046	0,193	0,064	0,157	0,014	0,049	0	0,114	0,006	0,041	0,008
0,096	0,026	0,152	0,043	0,13	0,04	0,121	0,006	0,032	0,002	0,079	0,005	0,024	0,006
0,092	0,022	0,074	0,042	0,067	0,035	0,051	0,005	0,011	0,002	0,083	0	0,018	0,009
0,101	0,026	0,074	0,033	0,106	0,042	0,069	0,004	0,016	0,003	0,104	0,002	0,025	0,005
0,079	0,033	0,049	0,033	0,062	0,021	0,048	0,008	0,011	0,001	0,062	0,006	0,021	0,006
0,147	0,03	0,104	0,038	0,112	0,037	0,089	0,008	0,025	0,003	0,098	0,003	0,028	0,005
0,114	0,023	0,078	0,035	0,079	0,032	0,072	0,005	0,026	0,005	0,096	0	0,029	0,006
0,096	0,024	0,094	0,039	0,094	0,045	0,08	0,01	0,02	0,004	0,075	0,005	0,024	0,005
0,102	0,03	0,087	0,042	0,078	0,041	0,058	0,008	0,026	0,005	0,15	0,004	0,026	0
0,088	0,023	0,073	0,036	0,061	0,045	0,063	0,008	0,019	0	0,084	0,006	0,026	0,008
0,108	0,014	0,083	0,034	0,111	0,046	0,078	0	0,022	0,004	0,116	0	0,028	0,008
0,116	0,022	0,108	0,038	0,135	0,044	0,117	0,014	0,016	0,006	0,125	0,009	0,045	0,008
0,114	0,015	0,075	0,047	0,114	0,031	0,083	0,011	0,024	0,003	0,079	0,003	0,021	0,007
0,08	0,032	0,071	0,044	0,084	0,043	0,078	0,006	0,014	0	0,086	0,003	0,02	0,01
0,089	0,032	0,047	0,036	0,091	0,034	0,072	0,008	0,012	0	0,082	0,005	0,023	0,006
0,087	0,024	0,121	0,041	0,128	0,067	0,095	0,014	0,03	0,005	0,151	0,005	0,059	0,012
0,085	0,039	0,091	0,04	0,091	0,037	0,066	0,01	0,022	0,002	0,126	0,001	0,035	0
0,093	0,024	0,083	0,043	0,084	0,037	0,061	0,012	0,02	0,003	0,085	0,007	0,029	0,009
0,086	0,03	0,08	0,043	0,11	0,033	0,103	0,007	0,022	0,006	0,078	0,001	0,025	0,008
0,098	0,035	0,089	0,04	0,102	0,032	0,077	0,012	0,024	0,001	0,105	0,001	0,035	0,007
0,096	0,024	0,094	0,045	0,109	0,028	0,119	0,012	0,018	0,007	0,114	0,004	0,028	0,009
0,264	0,044	0,105	0,04	0,133	0,041	0,191	0,013	0,036	0,006	0,115	0,003	0,034	0,01
0,107	0,033	0,122	0,045	0,163	0,067	0,168	0,015	0,03	0,009	0,183	0,008	0,049	0,005
0,123	0,046	0,08	0,039	0,098	0,03	0,083	0,01	0,022	0,005	0,145	0,007	0,04	0,004
0,126	0,033	0,089	0,037	0,139	0,047	0,129	0,013	0,027	0	0,11	0	0,056	0,01
0,104	0,029	0,075	0,044	0,129	0,044	0,141	0,008	0,022	0,005	0,11	0,009	0,04	0,008
0,069	0,02	0,071	0,038	0,123	0,039	0,099	0,004	0,027	0,007	0,046	0,004	0,04	0,008
0,08	0,015	0,074	0,043	0,089	0,034	0,057	0,01	0,016	0	0,044	0,004	0,02	0,004
0,05	0,021	0,037	0,028	0,051	0,033	0,042	0,001	0,003	0	0,01	0,004	0,013	0,004
0,07	0,019	0,077	0,034	0,085	0,028	0,066	0	0,012	0,002	0,066	0	0,025	0
0,101	0,026	0,074	0,043	0,093	0,034	0,069	0,005	0,023	0	0,087	0,007	0,024	0,006
0,09	0,01	0,064	0,037	0,081	0,046	0,069	0,007	0,009	0	0,112	0,003	0,026	0,002

0,141	0,029	0,113	0,036	0,135	0,049	0,126	0,015	0,031	0	0,097	0,006	0,037	0,011
0,084	0,016	0,061	0,044	0,094	0,042	0,097	0,007	0,026	0,005	0,129	0,004	0,048	0,001
0,129	0,015	0,119	0,037	0,108	0,051	0,141	0,013	0,036	0,003	0,104	0,01	0,038	0,014
0,154	0,036	0,104	0,045	0,145	0,048	0,121	0,013	0,029	0,004	0,105	0,006	0,042	0,007
0,091	0,02	0,047	0,04	0,063	0,026	0,069	0,004	0,016	0	0,104	0,004	0,024	0,005
0,068	0,02	0,06	0,04	0,076	0,019	0,055	0,01	0,021	0,004	0,075	0,005	0,024	0,005
0,136	0,035	0,074	0,038	0,097	0,037	0,076	0,001	0,025	0,001	0,102	0,003	0,03	0,007
0,068	0,025	0,063	0,044	0,118	0,034	0,082	0,008	0,016	0	0,104	0,003	0,047	0,007
0,085	0,028	0,076	0,047	0,076	0,042	0,07	0,007	0,019	0,001	0,112	0,007	0,028	0,008
0,046	0,02	0,026	0,037	0,061	0,029	0,055	0,005	0,004	0,004	0,069	0,003	0,031	0,004
0,068	0,016	0,038	0,039	0,059	0,016	0,031	0,006	0,011	0	0,095	0,004	0,023	0,009
0,127	0,025	0,086	0,038	0,116	0,047	0,11	0,012	0,02	0,002	0,127	0,003	0,036	0,011
0,06	0,017	0,058	0,04	0,073	0,034	0,045	0,004	0,008	0,005	0,115	0,006	0,031	0,009
0,1	0,031	0,075	0,044	0,098	0,046	0,098	0,008	0,026	0,006	0,117	0	0,039	0,001
0,087	0,01	0,076	0,04	0,085	0,037	0,085	0,009	0,022	0,003	0,08	0,008	0,03	0,003
0,07	0,013	0,044	0,038	0,07	0,033	0,065	0,004	0,01	0,004	0,077	0,006	0,028	0,003
0,07	0,023	0,047	0,036	0,07	0,016	0,055	0,005	0,011	0,003	0,035	0,003	0,022	0
0,1	0,019	0,107	0,034	0,121	0,037	0,1	0,004	0,023	0,006	0,11	0,008	0,028	0,003
0	0	0	0	0	0	0,066	0	0	0	0,109	0	0	0
0,123	0,036	0,086	0	0	0	0,086	0	0	0	0,129	0	0	0
0,213	0	0,212	0	0,22	0	0,235	0,022	0,105	0,011	0,178	0	0,065	0
0,275	0,041	0,222	0	0,219	0	0,225	0,021	0,069	0	0,15	0	0,045	0
0	0	0,087	0	0	0	0,07	0	0	0	0,118	0	0	0
0	0,036	0,085	0	0	0	0,083	0	0	0	0,101	0	0	0

C16.2	C16.2.OH	C18	C18.1	C18.1.OH	C18.2	lysoPC.a.C14.0	lysoPC.a.C16.0	lysoPC.a.C16.1	lysoPC.a.C17.0
0	0	0,05	0,209	0	0,064	2,05	136	2,43	2,45
0	0	0,057	0,17	0	0,074	2,45	132	3,62	2,12
0	0	0,041	0,136	0	0,039	1,27	117	2,09	2,07
0	0	0,05	0,091	0	0,027	1,92	143	2,87	2,94
0	0	0,051	0,174	0	0,046	1,12	105	2,66	1,83
0	0	0,044	0,15	0	0,039	1,48	134	2,71	2,53
0	0	0,024	0,114	0	0,033	1,29	79,3	2,2	1,5
0	0	0,04	0,16	0	0,04	1,78	118	3,17	2,26
0	0	0,041	0,122	0,01	0,047	1,54	88,8	1,76	1,31
0	0	0,05	0,15	0	0,05	1,4	103	2,11	1,51
0	0	0,032	0,081	0	0,04	1,61	124	3,01	2
0	0	0,039	0,124	0	0,035	2,08	126	2,81	1,94
0	0	0,037	0,09	0	0,03	2,87	117	2,62	2,26
0	0	0,032	0,138	0	0,035	3,18	149	4,92	2,64
0	0	0,041	0,096	0	0,029	2,21	135	3,05	2,42
0	0	0,05	0,155	0	0,048	2,29	138	3,86	1,73
0	0	0,036	0,097	0	0,027	3,09	158	3,22	2,32
0	0	0,044	0,108	0	0,025	4,11	174	4,81	1,53
0	0	0,071	0,159	0	0,04	1,87	145	3,48	2,74
0	0	0,037	0,14	0	0,042	1,91	117	3,39	1,95
0	0	0,034	0,152	0	0,052	1,36	105	2,08	1,85
0	0	0,046	0,131	0,009	0,038	1,54	122	3,02	1,88
0	0	0,057	0,09	0	0,023	3,98	164	4,04	3,36
0	0	0,06	0,133	0	0,028	1,88	106	2,45	2,08
0	0	0,045	0,14	0	0,042	1,02	92,2	1,96	1,42
0	0	0,046	0,163	0	0,04	1,06	101	1,78	1,58
0	0	0,053	0,128	0	0,043	2,02	93,6	2,28	1,82
0	0	0,039	0,107	0	0,036	2,17	124	3,15	1,47
0	0	0,035	0,126	0	0,032	2,33	110	3,2	1,36
0	0	0,057	0,129	0	0,034	1,46	117	2,7	2,42
0,019	0	0,052	0,175	0,009	0,055	1,75	119	2,61	2,33
0	0	0,032	0,127	0	0,037	1,49	92,6	2,05	0,853
0	0	0,06	0,207	0	0,08	2,84	128	2,43	2,12
0	0	0,057	0,129	0	0,045	2,08	134	2,9	2,49
0	0	0,03	0,091	0	0,021	1,77	98,2	1,92	1,73
0,006	0,005	0,02	0,084	0,002	0,042	1,91	161	3,42	2,43
0,008	0,003	0,037	0,109	0,005	0,033	1,61	136	2,62	2,15
0,006	0,006	0,04	0,141	0,006	0,042	1,27	144	2,35	2,51
0,011	0,008	0,045	0,162	0,002	0,042	1,33	132	2,67	1,97
0,012	0,006	0,035	0,156	0,002	0,052	1,55	129	2,52	2,37
0,008	0,004	0,03	0,081	0,003	0,027	2,83	140	2,89	2,69
0,005	0,011	0,021	0,114	0,003	0,029	1,1	84,2	2,22	1,68
0,003	0,012	0,035	0,194	0,006	0,062	1,96	150	2,51	2,67
0,008	0,006	0,025	0,136	0,004	0,046	1,11	94	1,94	1,44
0,009	0,009	0,04	0,162	0,006	0,047	2,29	151	3,64	1,96
0,003	0,009	0,03	0,109	0,005	0,036	2,02	129	2,74	2,28

0,006	0,008	0,019	0,085	0,001	0,024	1,02	121	1,81	2,08
0,004	0,009	0,038	0,085	0	0,027	3,47	189	3,78	3
0,002	0,006	0,046	0,15	0,006	0,039	1,59	143	2,65	3,08
0,009	0,006	0,029	0,123	0,007	0,04	1,81	128	2,58	1,22
0,009	0,006	0,033	0,116	0,007	0,043	1,51	110	1,84	1,5
0,005	0,009	0,041	0,146	0,006	0,036	0,923	129	2,08	1,79
0,012	0,016	0,041	0,213	0,011	0,06	1,26	129	3,15	2,3
0,004	0,005	0,05	0,082	0,006	0,021	3,87	196	4,75	3,75
0,001	0,008	0,057	0,143	0,003	0,046	1,84	167	3,7	3,26
0,009	0,009	0,028	0,106	0,002	0,033	2,11	159	3,4	1,84
0,006	0,011	0,017	0,072	0,005	0,011	0,576	48,8	1,3	0,669
0,004	0,011	0,031	0,157	0	0,036	1,34	142	3,51	2,55
0,009	0,009	0,036	0,13	0,008	0,039	1,39	138	3,27	1,9
0,009	0,007	0,024	0,123	0,004	0,032	2,56	144	3,73	1,7
0,003	0,012	0,052	0,146	0,005	0,033	1,9	132	2,95	2,27
0,005	0,005	0,051	0,126	0,001	0,045	2,15	146	2,66	2,85
0,009	0,005	0,036	0,136	0,005	0,04	2,08	124	2,53	2,4
0	0,006	0,028	0,09	0,005	0,026	3,75	190	3,83	1,83
0,01	0,006	0,028	0,152	0,005	0,043	1,88	145	4,31	2,36
0,007	0,008	0,022	0,122	0,004	0,029	1,04	101	2,23	1,55
0,006	0,015	0,031	0,101	0,003	0,027	2,01	151	3,27	3,04
0,005	0,007	0,057	0,12	0,004	0,032	1,68	147	2,97	2,88
0,005	0,003	0,038	0,113	0	0,038	1,79	136	3,22	2,01
0,004	0,006	0,028	0,146	0,005	0,04	0,861	102	2,23	1,77
0,007	0,011	0,033	0,136	0,007	0,05	1,46	123	2,57	2,46
0,005	0,007	0,03	0,1	0,006	0,03	2,03	141	2,89	2,46
0,007	0,009	0,054	0,155	0	0,067	2,91	161	4	2,34
0,002	0,008	0,042	0,181	0,004	0,069	2,51	127	2,56	2,02
0,007	0,014	0,031	0,142	0	0,03	3,26	155	4,86	2,7
0,011	0,013	0,05	0,15	0,008	0,033	1,61	191	4,61	2,99
0,006	0,004	0,023	0,108	0,005	0,037	2,46	149	4,83	1,75
0,003	0,009	0,039	0,118	0,005	0,037	1,89	196	3,49	1,86
0,004	0,003	0,032	0,078	0,003	0,021	1,47	122	2,34	2,11
0,011	0,01	0,052	0,269	0,012	0,065	1,54	137	3,09	2,62
0,004	0,007	0,049	0,157	0,001	0,053	2,41	177	5,57	3,48
0,008	0,005	0,035	0,087	0,002	0,03	1,28	122	2,99	1,72
0,005	0,01	0,025	0,111	0,007	0,032	2,74	172	4,3	3,18
0,01	0,013	0,045	0,151	0,004	0,051	1,73	171	3,49	2,87
0,005	0,005	0,039	0,113	0,004	0,026	1,01	192	2,96	2,2
0,009	0,002	0,041	0,133	0,007	0,042	1,77	224	4,16	3,26
0,003	0,016	0,035	0,13	0,007	0,042	2,5	225	4,03	4,34
0,006	0,008	0,038	0,228	0,008	0,079	1,88	182	4,48	2,16
0,01	0,007	0,047	0,23	0,005	0,075	2,25	178	5,4	3,51
0,011	0,007	0,024	0,182	0,008	0,044	1,23	142	4,49	1,98
0,008	0,005	0,013	0,071	0,007	0,019	0,537	76,3	1,22	1,24
0,008	0,002	0,017	0,044	0,007	0,008	0,924	86	1,73	1,42
0,002	0,003	0,008	0,023	0,004	0,007	1,46	123	2,85	1,77
0,001	0,008	0,013	0,09	0,005	0,016	0,639	103	1,61	0,887
0,008	0,009	0,026	0,081	0	0,026	1,88	134	3,09	2,06
0,006	0,009	0,021	0,104	0,007	0,034	3,6	179	4,42	2,19

0,008	0,009	0,04	0,115	0,002	0,029	1,73	170	2,81	3,15
0,004	0,006	0,029	0,307	0,006	0,083	1,01	132	3,03	1,65
0,008	0,006	0,047	0,108	0,007	0,034	0,633	133	1,52	2,04
0,01	0,013	0,03	0,129	0,007	0,041	1,22	196	3,71	2,46
0,007	0,009	0,042	0,112	0,006	0,04	2,06	200	3,95	4,53
0,009	0,008	0,031	0,096	0,006	0,039	1,01	175	2,15	2,22
0,009	0,01	0,019	0,11	0	0,057	1,89	227	3,87	2,86
0,006	0,012	0,025	0,132	0,007	0,029	0,511	136	2,9	1,06
0,001	0,009	0,039	0,152	0,005	0,059	2,05	183	2,66	3,38
0,005	0,008	0,027	0,064	0,009	0,016	1,01	136	2,01	2,09
0,004	0,006	0,033	0,092	0,009	0,033	1,45	139	3,12	1,77
0,009	0,003	0,048	0,144	0,005	0,032	2,32	243	4,29	4,49
0	0,008	0,023	0,148	0,001	0,037	1,56	156	2,99	1,36
0,005	0,014	0,033	0,163	0,007	0,045	2,35	182	3,7	3,73
0,006	0,008	0,033	0,101	0,006	0,036	2,33	201	3,44	2,8
0,005	0,009	0,034	0,11	0,008	0,033	1,32	151	2,74	2,64
0,007	0,005	0,009	0,103	0,006	0,023	0,563	87,4	2,09	0,844
0,011	0,009	0,053	0,111	0,001	0,03	1,09	201	2,95	2,52
0	0	0,058	0,142	0	0,049	2,09	143	3,34	2,51
0	0	0,039	0,111	0	0,038	1,72	116	2,31	1,72
0	0	0,056	0,22	0	0,066	1,38	121	3,17	2,12
0	0	0,058	0,201	0	0,065	1,5	131	2,61	1,91
0	0	0,035	0,097	0	0,033	1,57	85,3	2,15	1,53
0	0	0,028	0,107	0	0,037	2,73	132	4,81	1,55

lysoPC.a.C18.0	lysoPC.a.C18.1	lysoPC.a.C18.2	lysoPC.a.C20.3	lysoPC.a.C20.4	lysoPC.a.C24.0	lysoPC.a.C26.0
35,2	24,3	30,7	2,66	7,58	0,324	0,524
35,6	34,8	53,5	3,19	11,1	0,132	0,228
38,4	33,2	55,1	3,37	8,81	0,16	0,134
49,2	39,5	65,4	2,89	10,2	0,203	0,4
30,5	30,2	38,2	2,56	11,8	0,219	0,225
45,2	29,1	39,6	2,17	7,02	0,296	0,569
18,5	17,9	19,1	1,25	4,12	0,364	0,653
34,9	25,3	31	1,89	7,43	0,576	1,33
25,4	15,1	22,1	1,63	3,79	0,203	0,296
41,8	37,7	57,7	3,56	7,42	0,188	0,136
36,2	35,1	66,4	2,99	6,06	0,169	0,187
39,4	28,3	42,4	2,57	7,54	0,124	0,19
41	18,9	28	2,02	5,95	0,127	0,169
44,9	31	28	2,52	8,36	0,493	0,822
38,9	28,3	31,7	2,38	6,77	0,179	0,174
43	33,1	39,6	2,32	6,59	0,23	0,291
45,8	26,4	33,4	1,83	5,65	0,122	0,156
47,2	32,1	26,2	3,3	9,29	0,262	0,295
53,8	36	58,6	3,46	11,9	0,192	0,238
34,5	29,8	38,2	2,77	6,36	0,224	0,322
33,1	22,3	38,2	1,74	5,01	0,247	0,465
36,5	28,1	40,6	2,57	7,71	0,138	0,128
47,3	35,4	39,9	2,23	10,8	0,086	0,213
30,2	26,5	31,6	1,96	7,32	0,202	0,254
25,8	23,9	34,5	1,7	4,89	0,21	0,275
35,8	23,7	28	1,05	4,84	0,216	0,212
29,9	18,7	28,7	1,44	5,59	0,284	0,49
38,3	31,6	50	4,28	8,74	0,215	0,404
30,2	23,2	24,8	2,31	7,09	0,298	0,639
39,3	30,2	37,2	1,83	8,23	0,31	0,436
47,5	33,4	45,2	2,61	8,72	0,201	0,199
19,7	13,2	12	1,74	4,76	0,103	0,188
42,7	25,2	40,3	2,33	6,34	0,152	0,183
51,8	34,4	49	2,89	7,97	0,201	0,362
22,1	17,4	13,9	1,39	3,85	0,321	0,734
52	41,9	66,1	3,32	7	0,162	0,185
43,9	27,9	37,6	1,97	8,73	0,224	0,133
45,8	36,5	54,9	3,49	8,5	0,058	0,335
44,3	28,8	34,1	2,25	9,41	0,106	0,152
55	33,8	42,2	2,6	8,26	0,179	0,094
48,1	21,1	28,4	1,96	6,07	0,123	0,15
19,1	18,4	16,5	1,21	3,8	0,186	0,22
40,5	24,6	27,9	2,46	7,48	0,13	0,25
37,3	31,2	43,3	2,67	5,84	0,073	0,106
48,4	34,2	35,7	2,19	6,82	0,42	0,639
40,5	30,3	46,2	2,52	5,31	0,195	0,264

31,7	20,1	12,2	1,35	3,98	0,119	0,181
62,7	32,3	32,6	2,29	7,18	0,087	0,152
46,9	28,4	36,4	1,77	6,31	0,07	0,077
26,4	15,6	12,1	1,66	5,12	0,074	0,238
31,1	16,9	21,9	1,82	4,13	0,047	0,16
47,7	30	29,3	1,28	5,51	0,193	0,184
34,3	32,2	38,1	2,7	7,56	0,065	0,383
59,8	38,5	39,6	2,63	12,1	0,175	0,165
62,2	40	58	3,75	12,1	0,158	0,196
48,9	35,2	47,5	4,01	9,27	0,107	0,123
18	8,39	5,35	0,984	2,35	0,081	0,1
41,8	27,4	29,4	1,82	7,42	0,098	0,143
41	29	38,2	2,64	7,59	0,109	0,082
41,4	28,5	26,2	2,67	8,01	0,206	0,339
38,5	31,6	33,7	2,54	8,1	0,215	0,421
58,5	35	48	2,47	8,45	0,223	0,287
40,4	22,1	29,1	1,78	6,14	0,14	0,143
55,5	31,3	21,4	3,16	8,02	0,117	0,2
43,2	34,2	37,6	2,92	6,81	0,05	0,13
28,7	23,9	30,3	1,53	4,29	0,114	0,195
43,6	30,1	30,5	2,37	6,77	0,114	0,18
48,4	34,2	39,7	1,93	8,78	0,291	0,265
47,3	35,8	51,8	2,12	7,63	0,186	0,159
29,3	26,1	30,4	1,87	9,51	0,145	0,092
40,3	24,6	36,1	1,54	5,39	0,134	0
44,8	29,9	40	2,15	7,26	0,111	0,076
43,5	38,1	52,4	3,32	11,5	0,174	0,207
42,4	23,7	33	1,78	5,41	0,114	0,293
47,6	29,3	24,5	2,26	8	0,284	0,416
61,9	32,6	23,3	2,48	14,6	0,5	0,472
31,8	35,1	45,2	3,05	6,29	0,148	0,277
63,8	37,5	39,1	3,07	9,45	0,169	0,13
47,6	30,5	36,1	2,13	7,58	0,072	0,08
36,1	28	23,7	2,01	8,56	0,102	0,143
68	41,5	44	2,58	11,1	0,181	0,222
42,1	20,7	15,2	2,27	4,18	0,209	0,236
52,1	31,3	31,3	2,94	5,93	0,161	0,293
60,5	37,4	35,8	2,55	8,39	0,183	0,546
63	37,6	42,4	4,42	9,63	0,129	0,285
88,7	42,3	31	2,23	11,7	0,218	0,149
79,3	29,9	22,4	3,24	11,9	0,246	0,172
48,6	29,9	23,5	3	6,87	0,118	0,105
61	33,9	21,9	3,17	10,3	0,082	0,149
46,6	26,3	18,2	2,06	6,85	0,313	0,505
15,3	8,63	5,45	0,981	4,56	0,201	0,33
22,7	9,81	10,5	1,48	5,16	0,139	0,159
45	22,8	14	3,07	7,7	0,131	0,156
26,1	13,6	17,4	2,67	9	0,133	0,144
39	20,9	28,2	3,15	8,92	0,128	0,179
46,9	27,4	28,9	2,57	7,56	0,193	0,218

61,5	27	27,3	2,78	9,08	0,207	0,107
37	19,6	8,33	2,69	6,61	0,184	0,442
54	29,6	28,4	1,57	8,18	0,225	0,348
60,9	35,8	28,4	2,5	8,81	0,191	0,223
86,4	27,4	29	3,41	12,1	0,394	0,595
47,1	28,9	27,9	2,06	7,25	0,125	0,109
83	30,4	27,5	3,66	10,5	0,133	0,224
37,1	21,7	15,7	2,44	9,27	0,062	0,087
59,3	30,8	27,9	2,17	10,6	0,195	0,217
42,5	18,8	14,1	1,8	5,7	0,133	0,162
50,7	27,2	22,5	3	6,13	0,196	0,318
83,9	45,4	38,8	4,51	12,9	0,355	0,576
28,7	16	8	3,05	3,46	0,126	0,709
62,6	32,3	20,3	3,01	10,7	0,307	0,508
69,6	24,5	24,2	2,94	8,34	0,285	0,556
47,8	33,8	35,5	2,2	8,07	0,242	0,418
19,6	15,1	8,85	1,19	4,57	0,117	0,169
66,2	38,1	41,8	4,47	8,83	0,155	0,269
49,4	41	62,5	2,6	8,73	0,236	0,368
35,2	25,4	39,1	2,09	8,91	0,154	0,117
29,6	32,3	41,9	2,97	7,21	0,159	0,194
42,3	30,7	40,7	2,81	10,1	0,491	1,38
26,3	21,3	36,4	1,88	3,94	0,126	0,176
27,9	32,2	46,5	2,97	5,89	0,222	0,429

lysoPC.a.C26.1	lysoPC.a.C28.0	lysoPC.a.C28.1	PC.aa.C24.0	PC.aa.C26.0	PC.aa.C28.1	PC.aa.C30.0	PC.aa.C30.2
0,294	0,442	0,455	0,16	0,841	2,58	2,34	0
0,101	0,204	0,186	0	0	1,59	2,95	0
0,097	0,196	0,215	0	0	1,66	1,98	0
0,207	0,35	0,394	0,115	0	2,93	4,25	0
0,216	0,209	0,35	0,125	0	1,58	1,86	0
0,295	0,46	0,681	0,163	1,18	2,77	3,82	0
0,392	0,489	0,793	0,142	0,859	2,71	2,45	0
0,886	0,955	1,22	0,384	2,42	3,84	3	0
0,125	0,251	0,476	0	0	2,92	3,67	0
0,088	0,189	0,186	0	0	1,48	2,02	0
0,153	0,314	0,473	0,083	0	3,01	2,93	0
0,121	0,193	0,327	0	0	2,17	3,82	0
0,146	0,218	0,553	0	0	4,12	4,61	0
0,541	0,652	0,915	0,211	1,11	3,02	4,48	0
0,111	0,22	0,324	0	0	3,06	3,04	0
0,153	0,25	0,206	0	0	2,41	4,36	0
0,148	0,175	0,266	0	0	3,13	4,45	0
0,153	0,273	0,532	0,15	0	3,13	10,3	0
0,203	0,266	0,418	0,099	0	3,58	3,93	0
0,127	0,228	0,382	0	0	2,17	2,31	0
0,213	0,312	0,387	0,105	0,874	2,6	2,2	0
0,097	0,166	0,202	0	0	1,99	2,35	0
0,219	0,223	0,412	0,088	0	3,84	7,34	0
0,135	0,242	0,327	0	0	2,64	3,63	0
0,139	0,245	0,345	0,076	0	2,06	1,9	0
0,174	0,209	0,318	0	0	2,37	1,86	0
0,39	0,463	0,645	0,163	1,04	3,87	4,22	0
0,177	0,295	0,476	0,132	0,849	2,02	3,32	0
0,469	0,457	0,529	0,236	1,21	2,33	3,74	0
0,396	0,36	0,568	0,124	0,874	2,97	3,75	0
0,128	0,185	0,353	0	0	2,3	2,71	0
0,115	0,144	0,267	0	0	2,13	3,69	0
0,14	0,279	0,47	0	0	3,74	3,65	0
0,252	0,244	0,575	0,087	0	3,23	4,22	0
0,215	0,364	0,618	0,132	0,913	2,22	4,4	0
0,173	0,069	0,602	3,21	2,93	0	12,5	6,45
0,24	0,118	0,599	1,83	1,94	0	8,64	5,45
0,172	0,089	0,679	1,91	2,3	0	9,08	5,08
0,144	0,057	0,418	2,12	2,09	0	7,8	5,32
0,067	0,052	0,356	2,19	2,35	0	9,03	5,6
0,086	0,048	0,41	3,53	4,41	0	9,51	9,64
0,134	0,075	0,425	2,45	2,12	0	8,15	7,82
0,114	0,076	0,529	2,39	2,08	0	8,82	6,68
0,089	0,018	0,445	1,52	2,06	0	6,78	4,8
0,304	0,201	1,25	2,76	4,49	0	13,4	18,4
0,145	0,049	0,511	1,91	2,95	0	8,85	8,17

0,111	0,039	0,38	2,18	4,23	0	12,1	16,6
0,132	0,043	0,433	3,06	4,23	0	12,3	15,9
0,108	0,021	0,381	2,38	3,65	0	12	10,4
0,106	0,029	0,4	2,24	3,81	0	13,1	17,3
0,095	0,067	0,46	2,78	3,47	0	10,2	8,67
0,14	0,055	0,425	2,24	1,92	0	8,51	4,07
0,21	0,104	0,747	1,73	1,94	0	10,3	10,9
0,194	0,102	0,538	3,73	7,09	0	15,4	19,7
0,136	0,072	0,41	3,4	3,77	0	12,8	10,6
0,074	0,05	0,412	1,88	2,94	0	10,2	11,3
0,06	0,048	0,321	0,75	1,41	0	7,2	7,54
0,106	0,053	0,406	3,29	3	0	13	11,4
0,087	0,045	0,357	1,84	2,02	0	7,86	7,65
0,174	0,092	0,62	2,33	3,64	0	10,5	18,8
0,222	0,102	0,762	2,78	4,39	0	14,3	11,8
0,144	0,078	0,6	3,17	4,37	0	12,4	10,8
0,104	0,063	0,413	3,89	4,24	0	12,3	9,69
0,119	0,112	0,577	2,75	9,2	0	21,2	35,3
0,078	0,038	0,371	2,31	2,56	0	9,73	11,1
0,105	0,058	0,469	1,75	1,76	0	8,36	6,75
0,2	0,091	0,618	3,02	2,85	0	10,5	10
0,19	0,083	0,608	3,06	4,08	0	17,7	11,8
0,119	0,088	0,578	1,98	2,19	0	9,2	8,18
0,075	0,037	0,348	1,38	1,63	0	8,51	6,59
0,099	0,041	0,352	2,33	2,08	0	8,22	4,86
0,07	0,018	0,361	1,8	3,23	0	9,12	8,69
0,127	0,081	0,442	1,58	2,75	0	8,34	12,2
0,205	0,091	0,662	3,51	3,26	0	8,51	6,63
0,198	0,132	0,737	3,02	4,42	0	12,2	22,3
0,445	0,29	0,857	3,27	3,26	0	18,6	13,6
0,143	0,039	0,658	2,47	3,68	0	10,8	24,3
0,106	0,055	0,42	1,73	2,49	0	12,4	10,9
0,047	0,032	0,359	2,11	2,28	0	9,34	6,58
0,103	0,055	0,4	2,43	2,54	0	9,97	8,6
0,138	0,129	0,509	2,79	4,96	0	16,4	23,8
0,077	0,071	0,532	2,5	2,91	0	12,2	13
0,126	0,095	0,661	3,43	2,69	0	8,68	9,26
0,256	0,18	0,877	2,43	2,7	0	12,8	7,28
0,13	0,055	0,505	1,96	1,88	0	11,3	6,52
0,165	0,054	0,359	2,97	2,7	0	12,3	7,4
0,132	0,075	0,338	2,44	7,33	0	20,1	24,1
0,076	0,054	0,396	1,69	2,49	0	8,75	16,3
0	0,042	0,343	3,06	2,77	0	12,6	8,13
0,248	0,139	0,563	1,7	2,36	0	15	13,9
0,356	0,143	0,811	2,46	1,82	0	9,99	9,83
0,124	0,048	0,443	2,17	1,78	0	8,65	9,38
0,135	0,05	0,332	1,81	2,15	0	10,5	8,34
0,077	0,06	0,393	2,39	1,8	0	10,3	9,4
0,122	0,056	0,388	2,26	3,14	0	9,88	12,6
0,096	0,065	0,537	4,25	6,92	0	18,4	26,7

0,158	0,062	0,419	3,77	3,86	0	14,5	10,6
0,204	0,11	0,62	1,43	1,53	0	11,2	11,4
0,327	0,165	0,766	2,4	2,38	0	11,4	5,68
0,182	0,093	0,614	1,56	1,87	0	10,8	6,09
0,204	0,25	0,437	2,28	2,99	0	9,86	7,59
0,086	0,066	0,406	1,38	0,982	0	7,27	3,01
0,151	0,132	0,368	1,37	1,81	0	8,34	8,44
0,116	0,079	0,483	1,19	1,04	0	8,5	5,89
0,176	0,097	0,56	2,24	2,25	0	9,63	5,29
0,098	0,087	0,483	2,29	2,5	0	10,7	8,1
0,204	0,105	0,721	1,32	1,68	0	7,3	12
0,233	0,187	0,753	2,7	3,86	0	14,1	13
0,103	0,123	1,03	1,22	2,01	0	9,94	16,6
0,301	0,195	0,764	2,95	3,22	0	10,6	9,07
0,273	0,117	0,903	2,04	2,38	0	10,2	7,4
0,162	0,155	0,807	2,46	2,64	0	10,2	10,4
0,124	0,074	0,506	1,12	1,04	0	7,01	7,47
0,12	0,055	0,617	2,15	2,14	0	11,5	6,46
0,252	0,308	0,37	0,187	0,867	2,5	2,66	0
0,122	0,144	0,21	0,065	0,409	1,76	2,03	0
0,094	0,143	0,208	0,048	0,415	1,63	1,89	0
0,75	0,898	1,14	0,346	1,92	2,81	2,29	0
0,134	0,202	0,22	0,073	0,53	1,64	2,63	0
0,351	0,337	0,492	0,118	1,19	2,62	3,6	0

PC.aa.C32.0	PC.aa.C32.1	PC.aa.C32.2	PC.aa.C32.3	PC.aa.C34.1	PC.aa.C34.2	PC.aa.C34.3	PC.aa.C34.4	PC.aa.C36.0
10,1	7,98	1,86	0,231	166	334	7,31	1,05	0,847
8,84	13,8	2,76	0,249	195	334	12	1,45	0,763
9,39	6,25	1,41	0,203	151	363	7,96	0,652	0,499
15	11,5	2,95	0,352	224	498	12,1	1,21	0,835
9,97	8,26	0,966	0,276	171	293	7,95	0,696	1,17
15	12,2	2,02	0,397	213	440	11,5	0,82	1,32
9,26	9,17	2,25	0,247	176	399	11,4	0,947	0,81
13,8	13	1,9	0,376	208	415	10,5	0,998	1,01
12,6	11,6	5,07	0,361	212	668	13,6	1,48	0,767
7,7	5,46	1,2	0,206	116	258	8,76	0,489	0,198
13,5	7,86	2,44	0,442	184	544	14,8	0,958	1,15
11,1	10,4	2,27	0,37	184	417	12,3	1,35	1,05
11,5	11,8	5,47	0,553	161	485	24,3	2,41	0,73
12,6	23,8	3,89	0,493	246	361	19	2,12	0,721
10,7	10,8	2,15	0,287	217	413	12,8	1,15	1,72
14,3	20,4	3,56	0,412	284	471	14,7	1,38	1,17
12,3	17,8	3,45	0,4	240	475	16,4	1,62	0,276
25,8	43,6	5,9	0,534	457	562	23,6	3,65	1,16
14,4	12,7	2,71	0,33	201	478	10,5	1,29	2,06
9,11	10,7	2,18	0,284	164	327	11,7	0,925	0,574
9,17	5,63	1,9	0,332	140	381	10,5	0,818	1,06
9,6	10,3	1,77	0,203	171	360	9,8	0,829	0,36
16,5	22,4	4,2	0,42	240	429	13,8	3,17	1,1
12,8	11,2	2,1	0,278	190	353	10,2	1,06	1,11
9,77	7,86	1,29	0,233	170	356	12	0,561	0,748
8,96	4,92	0,37	0,238	152	321	5,44	0,343	1,35
12	9,97	2,96	0,413	149	404	12,4	1,35	1,74
10,8	13,3	2,6	0,306	181	375	11,8	1,27	0,125
10,5	20,9	3,4	0,297	215	318	13,8	1,88	0,517
16,9	11,4	1,66	0,305	215	419	10,8	0,865	2,1
10,3	6,37	1,88	0,446	172	387	19,9	1,31	2,05
13,8	17,9	3,38	0,321	255	490	15,7	1,91	1,72
10,5	8,96	4,92	0,458	178	529	19,6	2	0,811
13,4	11,6	2,97	0,396	188	400	12,5	1,49	1,58
12,7	17,8	3,69	0,442	246	404	15,6	1,75	1,34
2,62	0,4	170	490	13	0,95	0,987	36,7	258
1,69	0,276	125	263	7,39	1,04	1,23	26,6	148
1,2	0,23	148	345	7,47	0,695	0,588	30,1	177
1,25	0,18	139	261	7,05	0,762	0,391	27,2	151
2,02	0,396	156	339	17,1	1,12	1,9	40,1	239
5,09	0,565	132	399	19,9	2,25	0,476	33,3	217
2,33	0,224	149	342	9,9	0,897	0,606	27	145
1,82	0,192	143	281	6,4	0,889	0,779	24,2	131
1,13	0,167	102	224	7,38	0,426	0,324	29,1	154
3,29	0,41	267	438	13,4	1,28	1,13	54	233
2,22	0,299	142	348	8,68	0,706	0,705	27,7	181

3,78	0,425	243	396	15,4	1,74	1,46	40,2	176
3,5	0,397	222	434	14,5	1,53	0,302	49,4	229
1,92	0,225	183	374	9,25	0,74	1,16	36,3	200
3,71	0,356	238	452	14	1,85	1,86	34	171
4,39	0,306	175	541	10,9	1,27	0,714	35	236
0,427	0,21	136	279	4,56	0,316	1,5	30,4	169
1,74	0,291	195	345	10,4	0,642	0,436	28,5	159
4,12	0,412	215	375	11,9	2,93	0,848	44,9	195
2,45	0,321	186	437	9,5	1,2	2,17	42,8	246
2,51	0,264	165	341	11	1,19	0,227	37,4	187
0,526	0,061	80,4	91,2	2,68	0,346	0,647	16,8	63,5
1,92	0,256	187	373	9,27	0,995	0,904	31,9	182
1,68	0,218	140	302	7,83	0,663	0,458	23,3	147
3,28	0,286	202	289	12,8	1,77	0,57	35,1	150
2,28	0,305	201	361	10,2	1,22	1,2	37,2	176
2,72	0,344	175	366	11,1	1,33	1,76	44	227
3,26	0,407	147	393	12	1,31	1,65	30,5	202
5,58	0,437	378	457	19,3	3,12	1,27	71,4	241
2,45	0,325	174	335	11,5	0,948	0,548	30,7	165
1,16	0,235	139	288	9,47	0,487	0,504	23,3	141
2,64	0,307	203	379	11,6	1,2	1,22	36,4	192
1,86	0,299	214	406	10,9	0,896	2,24	41,7	216
2,28	0,258	165	332	10,2	0,898	0,498	35,1	207
0,844	0,189	138	228	6,16	0,582	1,05	21	111
1,8	0,269	124	331	9,28	0,765	0,916	25,7	177
2,13	0,314	151	330	9,74	1,11	0,902	33,6	179
2,76	0,26	175	295	9,98	1,37	0,947	28,8	144
4,45	0,423	144	406	14,9	1,76	0,656	32,5	224
3,84	0,425	231	324	16,3	1,99	0,707	40,8	180
1	0,39	226	326	9,55	0,95	1,41	40,5	178
4,96	0,391	258	463	21,3	1,73	0,576	39	179
1,28	0,162	179	276	8,94	0,754	0,821	36,5	174
1,95	0,21	125	286	7	0,802	0,625	27,6	179
1,44	0,258	134	242	7,3	0,953	0,687	21,3	117
3,44	0,444	261	434	15,5	1,55	0,865	52,9	284
1,33	0,208	164	253	7,78	0,746	0,182	43,2	162
3,17	0,31	148	317	10,6	0,991	0,464	30	165
1,55	0,263	165	300	7,24	0,728	0,919	34,2	185
0,535	0,17	171	356	6,22	0,489	0,301	32,9	193
1,23	0,317	185	336	8,78	0,944	1,34	38,5	205
1,95	0,313	258	283	11,5	1,62	2,39	52,8	160
1,72	0,284	218	263	11,3	0,895	0,544	42,8	136
1,43	0,287	161	242	8,02	1,08	2,11	33,1	155
1,22	0,25	191	246	7,46	0,667	2,67	33,1	157
0,546	0,274	162	298	5,57	0,487	0,473	18,1	99,5
1,12	0,233	145	266	6,58	0,468	0,578	18,8	110
0,584	0,132	149	161	4,44	0,565	0	39,6	112
0,504	0,19	161	306	5,14	0,359	0,062	24,4	126
1,82	0,341	162	303	10,9	1,43	0,497	31,3	158
5,58	0,372	267	510	17,6	2,48	0,549	44,3	236

2,19	0,383	181	379	10,9	1,25	1,64	37,1	216
1,05	0,129	222	266	7,96	0,601	0	35,7	130
0,638	0,28	164	292	6,4	0,473	1,31	33,6	181
0,6	0,209	160	270	6,63	0,422	0,766	28,4	146
1,18	0,308	138	248	6,62	0,947	0,764	35,3	163
0,634	0,171	122	253	5,04	0,39	0,486	22	121
0,449	0,188	161	188	4,75	0,613	1,26	37,5	120
0,233	0,151	122	204	3,37	0,232	0	21	102
1,55	0,3	137	248	8,09	1,13	1,28	29,4	135
1,36	0,244	165	312	7,84	0,773	0,857	29,4	149
0,936	0,157	165	184	6,42	0,557	0	45,4	127
2,18	0,274	223	343	10,9	1,36	1,31	43,9	199
1,08	0,115	200	188	7,31	0,417	0	27,3	74,5
1,81	0,337	170	226	9,71	1,29	0,705	31,9	133
2,84	0,317	136	340	8,14	1,3	0,951	28,1	191
2,2	0,293	216	395	9,49	0,719	0,652	35,3	198
0,51	0,207	164	211	5,49	0,28	0,416	18,3	87,8
0,501	0,176	182	378	6,24	0,509	0,192	34,9	205
11,6	10,8	2,61	0,338	221	455	13,9	1,14	0,75
9,35	6,3	1,88	0,292	142	306	8,79	1,09	1,17
10,5	12,1	1,56	0,274	214	383	11,6	0,694	0,441
9,46	6,94	1,57	0,285	164	315	8,34	0,874	0,515
7,54	6,93	1,94	0,256	118	287	7,37	0,645	0,392
10,7	24,3	4,84	0,442	260	495	22,4	1,88	0,451

PC.aa.C36.1	PC.aa.C36.2	PC.aa.C36.3	PC.aa.C36.4	PC.aa.C36.5	PC.aa.C36.6	PC.aa.C38.0	PC.aa.C38.1	PC.aa.C38.3
24,2	139	83	140	19,8	0,571	1,9	0,304	27,9
30,2	148	71,8	142	25,5	0,795	1,92	0,318	22
27,9	167	87,6	125	7,75	0,284	1,61	0,328	25,5
43,6	260	110	169	13,8	0,381	2,1	0,772	24,7
24,2	127	59,7	147	17	0,412	2,68	0,516	22,5
38,1	215	82,2	131	14,5	0,525	2,66	0,569	27
28,4	158	107	148	24,1	0,664	1,77	0,847	22,4
35,3	202	92,9	177	15,5	0,557	1,81	0,571	26,8
38,8	273	162	197	8,41	0,701	2,45	0,582	47,7
33,1	171	82,3	87	9,17	0,242	1,56	0,377	27,9
36,6	276	156	150	12,5	0,489	2,62	0,413	35,2
40,9	219	97,3	153	11,4	0,429	1,84	0,199	30,9
38,9	255	124	184	22,2	0,87	1,46	0	37,6
43,5	197	111	181	22,6	0,842	1,74	0,986	41,5
38,3	204	111	168	18,2	0,596	2,88	0,635	32
55,6	245	107	156	17,4	0,705	3,01	0,825	32,4
49,1	242	109	168	23,1	0,727	1,65	0,357	32,4
82,2	280	167	316	59,2	1,73	2,45	0,484	60,8
44,5	258	112	197	11,6	0,656	3,67	0,535	33,9
28	161	90,9	110	14,9	0,51	1,56	0,646	26,2
27,2	199	88	111	14,5	0,505	2,13	0,622	25,2
28,1	173	84,6	125	9,37	0,306	1,67	0,327	26,3
48,1	216	104	259	17,9	0,767	2,06	0,195	30,1
35,2	167	85,8	151	11,7	0,416	2,17	0,364	25,5
28	172	88,7	99,3	13,2	0,363	2	0,39	23,1
33,2	183	66,4	106	33,9	0,504	2,23	0,535	18,2
30,3	204	87,6	147	24,5	0,777	2,3	0,621	28,3
38,4	201	115	155	16,8	0,498	1,5	0,169	37,6
35,9	157	93,9	169	21,7	0,761	1,54	0,661	29,5
41,3	218	86,2	161	24,3	0,773	3	1,01	24,8
42	255	109	161	21,5	0,847	3,6	1,01	33,2
35,1	173	142	253	16	0,948	3,75	0,269	38,9
39	270	128	172	16,9	0,759	2,01	0,379	35,8
45,6	246	95,2	141	21,5	0,776	3,15	0,692	34,7
38,4	171	102	168	14,5	1,21	3,14	1,13	29,5
149	137	11,3	0,389	2,32	0,39	36,6	62,4	30,6
66,4	136	21,9	0,65	2,53	0,606	25	81,3	36,3
92,9	127	7,68	0,29	1,69	0,293	27,7	64,7	26,9
67	127	10,7	0,311	1,38	0,539	23,2	73	28,8
101	148	19	0,672	3,5	0,903	34,1	98,7	41,2
106	159	18,9	0,779	1,35	0,486	34,1	81,5	37,4
100	138	21,4	0,572	1,61	0,538	21,2	54,9	30,9
80,5	130	18	0,473	1,66	0,239	27,1	62	34,3
72,5	74,6	7,68	0,235	1,39	0,321	25,9	43,8	19,2
105	150	16,6	0,776	2,86	1,02	32,5	73,6	38,6
87,3	90,2	7,08	0,386	2,15	0,221	24,9	43	21,3

108	173	14,6	1,32	3,21	0,976	33,4	68,2	37,4
102	157	20,8	0,618	1,58	0,418	34,4	80,4	38
78	121	13,5	0,465	2,39	0,504	25,2	60,6	31,6
134	242	15,3	0,85	3,67	0,308	40,7	92,9	38,8
141	174	6,66	0,59	2,17	0,402	41,7	79,8	29,4
60,2	94,1	30,4	0,471	2,26	0,602	19,5	56,8	38,1
89,3	118	9,93	0,312	1,44	0,434	27,9	63,6	33,7
94,8	234	15,8	0,739	1,87	0,484	29,1	107	42,5
107	186	11,1	0,525	3,49	0,503	34,8	95,6	38,7
109	148	15,4	0,533	1,53	0,117	38,6	75,9	36,9
35,8	57,7	4,77	0,164	1,19	0,295	21,3	41,8	14,3
85	161	14,3	0,46	1,73	0,407	27,1	84,8	37,5
76	109	7,92	0,3	1,43	0,223	24,3	53,8	25,1
88,5	161	20,1	0,677	1,51	0,331	30,9	75,5	36,6
91	157	12	0,493	2,22	0,472	28,6	80,8	35,1
89,3	130	19,3	0,757	3,18	0,511	35,1	74,2	34,4
85,3	145	23,2	0,818	2,43	0,673	30,7	73,5	35,8
144	269	49,6	1,5	2,41	0,351	57	136	76,9
94	112	14,7	0,539	1,79	0,563	29,9	56,8	29,9
72,9	80,5	10,6	0,305	1,67	0,36	20,2	40,2	21,8
105	159	16,3	0,608	2,91	0,866	32,4	78,5	38,7
86	160	23,1	0,686	3,18	0,849	28	82,8	43,3
88,2	115	17,9	0,483	1,9	0,347	23,7	63,2	34,9
51,9	124	14,4	0,261	2,29	0,592	19,9	68,5	30,6
79,2	97,2	12,4	0,466	2	0,675	24,8	53,8	25,9
81,9	125	9,8	0,424	1,53	0,333	27,9	65,3	26,3
71,5	136	23,3	0,672	1,84	0,371	22,7	60,9	34,7
106	136	13,3	0,594	1,7	0,22	31,2	71,6	30,4
101	167	19,8	0,69	1,64	0,531	40,1	99,2	47,7
86,5	242	28,7	0,938	2,24	0,561	38,7	150	61,1
118	152	22,7	0,817	1,48	0	29,2	55,2	33,2
82,1	132	15,1	0,348	1,53	0,723	30	74,3	29,6
73,5	105	11,7	0,398	1,68	0,465	24,8	61	27
66,5	131	11,4	0,317	1,47	0	20,6	70,8	34,2
106	206	19,6	0,795	2,41	0,84	35,4	118	49,1
96,2	97,7	10,9	0,319	1,45	0,431	45,2	64,5	29,1
98	87,9	7,7	0,445	1,48	0	36,3	49,1	22,7
92,8	132	12,5	0,384	2,15	0,269	33,6	83,3	38,7
112	121	5,52	0,152	1,33	0	42	66,7	24,2
89,3	183	15,4	0,546	2,61	0,408	41,1	126	51,9
90,6	222	24,6	1,28	3,71	0,874	51,7	133	55
83,3	122	23,4	0,587	1,34	0,482	32,9	64,8	37
97,4	158	18,3	0,747	3,43	0,947	44,6	112	41,9
71,4	127	19,9	0,68	4,3	1,4	39,2	82,1	37,9
59,4	199	13,8	0,25	1,06	0,385	17,1	81,4	30,9
52,7	110	10,1	0,305	1,4	0,179	20,1	61,7	23,3
73,2	103	7,53	0,262	0,832	0,368	41,2	75	23,5
69,7	151	15,5	0,256	0,862	0,133	25,3	75,6	28
98,8	184	20,7	0,643	1,58	0,474	41,6	97,8	35
143	229	23,6	0,852	1,79	0	46,2	106	48,1

109	189	18,2	0,641	2,24	0,705	44,9	113	42,4
121	151	12,7	0,294	1,24	0,065	50,6	89,3	31,2
62,7	124	7,67	0,278	2,3	0,757	25,3	82,8	27,2
74,7	106	7,88	0,282	2,1	0,586	29,3	66,5	25,3
76,9	157	12,3	0,527	1,54	0,451	41,8	116	36,3
71,9	106	10,7	0,288	0,961	0,215	22,4	51,5	27,9
66,3	121	40,5	0,978	1,98	0,418	38,1	81	54,1
54,8	108	5,12	0,098	0,882	0,346	25,4	60,6	18,4
63,9	141	26	0,695	2,23	0,855	26,9	77,4	38,7
77,9	124	13,4	0,455	1,55	0,275	28,1	67,6	26
74,6	87	6,78	0,255	0,863	0,535	39,4	61,3	24,1
120	187	24,7	0,801	2,41	0,958	46,1	106	54
98,5	70,5	9,79	0,229	0,61	0	33,3	35,3	23,2
78,8	140	16,7	0,588	1,4	0,163	38	86,5	43,5
100	156	12,8	0,628	2,05	0	42,5	91,8	36,6
85,2	132	6,11	0,343	1,6	0,713	25,9	64	28,2
56,5	94,9	6,25	0,145	0,939	0,35	17	48,4	21,5
117	123	5,65	0,177	1,41	0	42,8	66,9	25
41,3	268	117	154	23,6	0,589	2,19	0,766	28,7
29,2	164	73	151	24,9	0,753	2,52	0,29	26
29,4	173	97,1	129	10,8	0,258	1,58	0,482	27,7
30,6	169	77,7	144	13,1	0,363	1,66	0,415	25
22,4	149	68,7	71,5	5,64	0,298	1,63	0,411	18,8
39,3	184	120	159	23,3	0,908	1,35	0,249	27,4

PC.aa.C38.4	PC.aa.C38.5	PC.aa.C38.6	PC.aa.C40.1	PC.aa.C40.2	PC.aa.C40.3	PC.aa.C40.4	PC.aa.C40.5	PC.aa.C40.6
64,8	34,8	66,7	0	0,158	0,259	1,71	4,85	16,8
60,4	33,2	56,7	0	0,135	0,248	0,955	4,05	15,2
62,3	25,9	47,5	0	0,138	0,255	1,41	3,97	12,2
70,4	35,2	42,1	0	0,174	0,32	2,01	4,92	11,1
76,7	35	67,5	0	0,187	0,332	1,38	4,46	15,6
64,5	32,3	66,2	0	0,174	0,338	1,44	4,85	18,3
58,2	32,8	72,8	0	0,201	0,327	1,18	3,25	14
83,1	37,3	70,3	0	0,196	0,326	1,81	5,38	19,3
91,2	33,2	91	0	0,202	0,449	2,22	5,27	21,7
49,2	20,8	29,9	0	0,138	0,292	1,4	3,64	11,6
62,2	30,5	45,2	0	0,25	0,47	1,64	3,93	11,5
73,7	29,4	40,4	0	0,128	0,283	1,78	4,49	13,2
91,8	40,8	58,2	0	0,131	0,305	1,88	6,22	17,2
104	51	65	0	0,15	0,348	2,67	9,34	23,7
77,3	38,2	68,2	0	0,152	0,305	1,78	4,88	16,6
73,2	38,4	77,1	0	0,235	0,302	2,18	6,18	24,3
78	37	54,1	0	0,15	0,237	1,78	5,51	19,8
146	85,4	140	0	0,175	0,461	4,52	16,9	52
94	38,5	72,1	0	0,264	0,392	2,36	5,83	18,6
51,2	27,8	45,4	0	0,154	0,241	1,38	3,38	10,8
55,6	26,5	61,5	0	0,17	0,343	1,1	3,81	19,1
60,2	26,5	50,7	0	0,096	0,224	1,33	4	12,8
108	43,1	57,2	0	0,096	0,24	2,21	5,34	15,6
72,4	31,7	50,8	0	0,144	0,275	1,53	4,16	13
44,1	23,7	55,4	0	0,166	0,263	1,05	2,8	12,8
52,2	35,5	67,5	0	0,186	0,27	0,967	4,22	21,1
68,9	33,5	61,1	0	0,173	0,389	1,52	3,83	16,2
74,3	36,1	45,8	0	0,193	0,309	1,88	5,76	15
73,1	35,9	52,5	0	0,162	0,286	1,85	4,61	14
75,7	40,8	87,3	0,391	0,257	0,448	1,95	5,54	23,2
99	41,8	81,8	0	0,145	0,306	1,99	5,89	27,3
90,1	37,7	106	0	0,174	0,259	2,16	5,57	28
82,2	34,8	46,1	0	0,178	0,324	1,89	5,1	13,8
75	34,8	54,1	0,371	0,22	0,468	2,05	4,94	17,5
61,4	34,2	123	0	0,19	0,392	1,88	4,82	29,2
45,2	0,331	0,287	0,346	1,69	3,91	11,5	0,464	0,151
69,5	0,331	0,197	0,348	1,63	5,32	21,6	0,418	0,194
51,4	0,254	0,132	0,291	1,46	4,04	13,5	0,302	0,147
44,5	0,194	0,112	0,244	1,57	4,64	13	0,181	0,103
79,3	0,294	0,196	0,33	1,93	5,91	27,3	0,448	0,216
52,2	0,23	0,143	0,284	1,66	5,59	15,2	0,222	0,092
68,3	0,221	0,182	0,291	1,16	2,92	13,3	0,304	0,128
63,1	0,239	0,077	0,199	1,67	4,66	16,5	0,271	0,142
26,5	0,267	0,151	0,227	1,39	3,38	10,4	0,251	0,148
76,9	0,331	0,143	0,322	2,13	6,18	24,3	0,414	0,217
44,7	0,304	0,15	0,272	1,16	3,12	12,1	0,513	0,188

136	0,272	0,136	0,321	2,07	5,59	32,9	0,644	0,212
54,6	0,201	0,075	0,251	1,78	5,87	20,3	0,199	0,126
63	0,257	0,162	0,261	1,55	4,59	17,3	0,411	0,17
108	0,354	0,146	0,306	2,28	5,92	28,4	0,544	0,23
79,4	0,244	0,167	0,359	2,06	4,66	20	0,283	0,13
71,6	0,278	0,165	0,255	1,09	4,51	22,4	0,299	0,132
39,1	0,29	0,142	0,256	1,81	5,39	10,9	0,379	0,165
55,8	0,223	0,116	0,244	2,27	5,05	15,1	0,211	0,138
72,4	0,346	0,233	0,304	2,43	5,92	19	0,6	0,269
46,9	0,245	0,171	0,234	2,01	5,95	15,6	0,331	0,125
23,5	0,214	0,066	0,156	1,33	3,02	10,5	0,17	0,105
72,1	0,268	0,168	0,303	1,72	5,68	19,5	0,293	0,126
47,1	0,24	0,057	0,192	1,31	3,41	12	0,285	0,129
53,8	0,258	0,137	0,231	1,94	4,85	14,7	0,343	0,152
57,8	0,279	0,103	0,291	1,72	4,85	15,1	0,504	0,193
53,3	0,363	0,171	0,412	2,05	4,85	17,2	0,515	0,241
64,7	0,26	0,179	0,302	1,63	4,09	16,8	0,336	0,174
126	0,269	0,184	0,309	4,23	15,8	47,5	0,323	0,17
51,4	0,21	0,137	0,281	1,48	3,97	11,6	0,318	0,146
49	0,256	0,101	0,204	1,01	2,38	10,9	0,293	0,16
67,8	0,276	0,14	0,312	1,87	4,98	17	0,306	0,149
93,9	0,4	0,282	0,426	2,19	5,99	24,3	0,661	0,284
40,1	0,285	0,152	0,264	1,48	4,39	11,5	0,39	0,174
57,8	0,314	0,179	0,276	1,36	3,84	13,8	0,557	0,226
59,8	0,255	0,095	0,29	1,04	3,73	18,4	0,294	0,162
36,1	0,24	0,074	0,243	1,57	4,31	11,7	0,236	0,114
57,1	0,255	0,146	0,234	1,08	4,2	15	0,248	0,101
39,7	0,22	0,136	0,282	1,65	4,6	11,8	0,192	0,092
61,6	0,242	0,145	0,335	2,38	8,61	23	0,332	0,191
90,5	0,378	0,725	0,824	3,23	8,1	27,5	0,332	0,172
64,4	0,283	0,174	0,272	1,55	4,58	16,1	0,29	0,124
37,3	0,219	0,193	0,335	2,1	4,72	12,8	0,216	0,113
36,9	0,258	0,185	0,314	1,47	3,71	10,7	0,28	0,12
37,8	0,205	0,133	0,276	1,8	4,89	10,3	0,211	0,132
70,4	0,25	0,293	0,485	2,29	7,67	24,2	0,399	0,204
23,5	0,245	0,16	0,272	2,68	6,21	10	0,239	0,097
37,5	0,32	0,144	0,386	1,48	3,81	12,1	0,279	0,194
41,5	0,294	0,239	0,456	1,93	5,65	12,9	0,338	0,193
25,6	0,234	0,154	0,316	2,1	4,35	7,32	0,114	0,071
85,1	0,343	0,182	0,399	2,33	8,86	28,2	0,5	0,257
170	0,397	0,337	0,488	4,17	14,3	73,7	0,585	0,325
70,2	0,229	0,173	0,297	1,76	6,54	24,6	0,104	0,072
74,4	0,295	0,152	0,39	1,89	6,02	28,2	0,361	0,207
85,9	0,476	0,405	0,658	1,88	5,55	33,4	0,669	0,309
72,3	0,194	0,103	0,198	1,16	4	16,2	0,173	0,078
68	0,24	0,091	0,22	1,24	3,52	19,6	0,295	0,125
39,5	0,206	0,127	0,22	2,12	5,63	20,1	0,166	0,098
48,6	0,221	0,14	0,275	1,33	5,08	16,7	0,152	0,072
43,7	0,38	0,396	0,507	1,85	5,28	16,2	0,274	0,151
67,1	0,222	0,17	0,379	2,66	7,06	18,4	0,206	0,15

63,8	0,297	0,363	0,479	2,46	6,52	19,3	0,272	0,171
49,9	0,26	0,168	0,324	2,81	6,04	17,6	0,261	0,125
49,2	0,374	0,543	0,646	2,14	4,72	15,7	0,31	0,189
54,7	0,218	0,18	0,267	1,84	4,3	17	0,234	0,117
55,5	0,427	0,617	0,6	2,47	7,45	21,7	0,26	0,181
55,8	0,232	0,083	0,254	1,03	3,59	14,2	0,131	0,086
79,4	0,287	0,397	0,593	2,05	9,2	32,5	0,259	0,128
29,4	0,203	0,061	0,235	1,18	3,1	9,83	0,127	0,08
58,3	0,311	0,244	0,434	1,55	4,61	16,4	0,363	0,197
59,1	0,261	0,251	0,336	1,53	4,04	18,7	0,27	0,129
29,1	0,245	0,14	0,22	2,13	6,14	15,2	0,157	0,125
76,9	0,481	0,475	0,625	2,65	8,41	22,5	0,444	0,282
28,3	0,199	0,078	0,15	1,57	4,84	8,83	0,089	0,065
46	0,364	0,463	0,578	2,57	7,24	15,2	0,287	0,182
67,9	0,315	0,231	0,358	2,38	6,31	22,4	0,264	0,137
41,7	0,309	0,225	0,389	1,93	4,74	11,4	0,299	0,178
35,2	0,201	0,107	0,24	0,982	2,64	8,38	0,197	0,077
24,7	0,208	0,217	0,389	2,19	4,54	7,4	0,159	0,069
75,6	42,2	49	0,326	0,221	0,373	1,76	5,32	13,5
83,8	38,1	71,9	0,315	0,229	0,335	1,6	5,52	22,5
63,7	34,4	41,1	0,265	0,11	0,289	1,9	5,55	11,4
77,8	31,9	47,8	0,265	0,177	0,266	1,73	5,09	13,9
32,1	16,3	33,8	0,277	0,099	0,179	0,819	2,2	8,7
51,2	31,9	61,7	0,272	0,148	0,356	1,42	4,06	15,2

PC.aa.C42.0	PC.aa.C42.1	PC.aa.C42.2	PC.aa.C42.4	PC.aa.C42.5	PC.aa.C42.6	PC.ae.C30.0	PC.ae.C30.1	PC.ae.C30.2
0,317	0,146	0,187	0,101	0,19	0,199	0,331	0,077	0,072
0,238	0,107	0,106	0,083	0,112	0,171	0,246	0	0,06
0,297	0,121	0,096	0,063	0,156	0,146	0,287	0	0,043
0,369	0,113	0,176	0,097	0,211	0,177	0,466	0,06	0,075
0,569	0,271	0,151	0,1	0,281	0,236	0,312	0,016	0,073
0,409	0,186	0,177	0,098	0,205	0,253	0,471	0,095	0,092
0,302	0,183	0,165	0,1	0,229	0,292	0,258	0	0,099
0,298	0,147	0,138	0,123	0,219	0,249	0,404	0,37	0,173
0,343	0,175	0,166	0,124	0,283	0,267	0,306	0	0,084
0,32	0,175	0,122	0,097	0,162	0,211	0,157	0	0,044
0,429	0,222	0,149	0,104	0,208	0,227	0,416	0	0,106
0,286	0,116	0,075	0,108	0,145	0,15	0,325	0	0,065
0,252	0,125	0,131	0,122	0,207	0,201	0,412	0	0,104
0,306	0,172	0,106	0,138	0,247	0,291	0,394	0,089	0,118
0,314	0,156	0,101	0,067	0,187	0,233	0,347	0	0,056
0,44	0,181	0,136	0,167	0,244	0,294	0,393	0	0,083
0,201	0,09	0,125	0,112	0,176	0,213	0,366	0	0,086
0,355	0,209	0,166	0,122	0,403	0,444	0,359	0	0,102
0,648	0,289	0,193	0,155	0,204	0,226	0,515	0	0,069
0,297	0,144	0,132	0,069	0,168	0,202	0,303	0	0,052
0,328	0,104	0,128	0,063	0,206	0,215	0,24	0	0,086
0,352	0,163	0,112	0,066	0,15	0,113	0,311	0	0,051
0,214	0,103	0,086	0,093	0,153	0,198	0,548	0	0,1
0,379	0,219	0,107	0,081	0,148	0,171	0,386	0	0,067
0,335	0,178	0,166	0,074	0,155	0,172	0,29	0,062	0,074
0,322	0,165	0,145	0,057	0,197	0,226	0,207	0	0,067
0,311	0,183	0,109	0,115	0,199	0,204	0,486	0,054	0,121
0,317	0,161	0,142	0,096	0,155	0,225	0,251	0,024	0,083
0,269	0,155	0,133	0,106	0,148	0,251	0,43	0,115	0,1
0,703	0,311	0,198	0,134	0,366	0,291	0,475	0,021	0,09
0,438	0,224	0,195	0,11	0,247	0,317	0,27	0	0,064
0,565	0,229	0,12	0,106	0,259	0,312	0,238	0	0,07
0,3	0,121	0,096	0,078	0,207	0,202	0,409	0	0,092
0,525	0,256	0,171	0,13	0,192	0,283	0,42	0	0,124
0,407	0,183	0,131	0,13	0,299	0,334	0,452	0,062	0,085
0,171	0,102	0,177	0,23	0,254	0,383	0,343	0	0,085
0,141	0,094	0,213	0,226	0,196	0,353	0,25	0,055	0,087
0,111	0,09	0,156	0,185	0,266	0,447	0,321	0,119	0,057
0,077	0,092	0,152	0,159	0,141	0,245	0,167	0	0,064
0,189	0,082	0,19	0,296	0,169	0,244	0,246	0	0,059
0,116	0,08	0,162	0,213	0,159	0,427	0,345	0	0,095
0,132	0,094	0,177	0,249	0,173	0,241	0,133	0	0,064
0,147	0,093	0,152	0,149	0,22	0,313	0,291	0,063	0,066
0,112	0,1	0,139	0,203	0,164	0,196	0,2	0	0,045
0,15	0,142	0,255	0,35	0,502	0,531	0,405	0,185	0,12
0,11	0,108	0,104	0,161	0,234	0,303	0,296	0,003	0,064

0,123	0,139	0,318	0,32	0,178	0,411	0,379	0	0,075
0,12	0,099	0,187	0,193	0,208	0,236	0,359	0	0,088
0,136	0,083	0,195	0,211	0,243	0,229	0,397	0	0,063
0,109	0,119	0,252	0,318	0,113	0,185	0,22	0	0,042
0,131	0,122	0,225	0,271	0,234	0,266	0,268	0	0,076
0,117	0,075	0,17	0,229	0,127	0,283	0,19	0	0,082
0,096	0,121	0,127	0,192	0,24	0,255	0,237	0,036	0,062
0,1	0,125	0,142	0,185	0,217	0,46	0,489	0	0,09
0,175	0,139	0,201	0,254	0,176	0,327	0,396	0	0,063
0,115	0,113	0,152	0,161	0,156	0,228	0,214	0	0,053
0,084	0,079	0,095	0,158	0,067	0,123	0,128	0,06	0,021
0,105	0,082	0,206	0,233	0,168	0,305	0,277	0	0,062
0,097	0,047	0,121	0,119	0,195	0,161	0,261	0,03	0,026
0,142	0,11	0,187	0,281	0,313	0,33	0,348	0,063	0,065
0,133	0,087	0,188	0,245	0,269	0,479	0,386	0	0,071
0,152	0,085	0,252	0,32	0,284	0,45	0,391	0,125	0,089
0,108	0,094	0,222	0,205	0,199	0,404	0,41	0	0,085
0,151	0,143	0,33	0,454	0,134	0,256	0,317	0	0,065
0,109	0,065	0,145	0,206	0,149	0,284	0,25	0,062	0,06
0,101	0,083	0,104	0,188	0,116	0,265	0,23	0,092	0,048
0,105	0,097	0,212	0,206	0,153	0,34	0,298	0,002	0,08
0,225	0,186	0,364	0,373	0,354	0,345	0,496	0	0,105
0,183	0,121	0,189	0,306	0,213	0,242	0,249	0,047	0,066
0,136	0,082	0,202	0,174	0,137	0,154	0,224	0,083	0,047
0,126	0,068	0,152	0,186	0,133	0,2	0,182	0	0,053
0,102	0,082	0,108	0,145	0,14	0,184	0,235	0	0,047
0,107	0,06	0,131	0,124	0,218	0,201	0,195	0	0,049
0,09	0,095	0,161	0,211	0,26	0,416	0,328	0	0,089
0,124	0,127	0,232	0,292	0,469	0,41	0,362	0,041	0,09
0,222	0,411	0,299	0,349	0,314	0,657	0,254	0,027	0,161
0,148	0,112	0,246	0,321	0,187	0,266	0,322	0,133	0,055
0,108	0,134	0,185	0,169	0,166	0,154	0,177	0	0,026
0,114	0,14	0,114	0,175	0,098	0,213	0,206	0	0,056
0,093	0,093	0,154	0,168	0,167	0,185	0,282	0	0,069
0,217	0,184	0,263	0,325	0,174	0,484	0,365	0	0,111
0,129	0,079	0,152	0,207	0,233	0,219	0,254	0	0,074
0,122	0,157	0,172	0,205	0,311	0,461	0,283	0	0,081
0,163	0,176	0,221	0,261	0,249	0,47	0,283	0,114	0,096
0,093	0,094	0,145	0,163	0,216	0,199	0,166	0	0,053
0,186	0,131	0,293	0,309	0,1	0,337	0,19	0	0,096
0,258	0,344	0,492	0,608	0,286	0,459	0,383	0	0,091
0,132	0,115	0,139	0,228	0,133	0,151	0,131	0	0,051
0,15	0,121	0,236	0,326	0,192	0,288	0,253	0,019	0,085
0,213	0,365	0,382	0,334	0,307	0,56	0,257	0,028	0,096
0,069	0,079	0,15	0,164	0,231	0,419	0,256	0	0,087
0,076	0,168	0,177	0,146	0,137	0,237	0,211	0	0,078
0,1	0,129	0,146	0,157	0,17	0,185	0,157	0	0,033
0,085	0,123	0,172	0,139	0,053	0,18	0,138	0,034	0,057
0,143	0,232	0,248	0,25	0,214	0,282	0,184	0	0,085
0,132	0,167	0,26	0,342	0,269	0,367	0,415	0	0,084

0,171	0,214	0,288	0,32	0,133	0,439	0,271	0	0,119
0,11	0,166	0,2	0,212	0,164	0,323	0,168	0,033	0,052
0,218	0,278	0,267	0,292	0,36	0,496	0,167	0	0,105
0,136	0,146	0,149	0,185	0,074	0,206	0,163	0	0,055
0,181	0,361	0,337	0,304	0,431	0,46	0,219	0	0,115
0,117	0,082	0,149	0,156	0,15	0,129	0,102	0	0,045
0,196	0,417	0,235	0,249	0,121	0,193	0,135	0	0,041
0,074	0,09	0,128	0,123	0,032	0,182	0,084	0,037	0,033
0,154	0,166	0,174	0,26	0,151	0,311	0,235	0	0,064
0,144	0,183	0,201	0,208	0,183	0,297	0,19	0	0,063
0,107	0,165	0,155	0,183	0,313	0,231	0,165	0,039	0,048
0,288	0,296	0,361	0,399	0,48	0,481	0,37	0,028	0,116
0,055	0,06	0,094	0,135	0,382	0,306	0,119	0,13	0,044
0,193	0,287	0,273	0,289	0,371	0,51	0,324	0	0,133
0,158	0,182	0,225	0,298	0,441	0,458	0,194	0,094	0,096
0,17	0,189	0,193	0,204	0,291	0,432	0,226	0	0,117
0,112	0,099	0,143	0,131	0,137	0,161	0,101	0,015	0,04
0,118	0,102	0,166	0,177	0,242	0,338	0,195	0	0,083
0,498	0,242	0,19	0,13	0,243	0,311	0,27	0	0,084
0,464	0,218	0,139	0,12	0,193	0,252	0,216	0	0,052
0,382	0,16	0,115	0,1	0,174	0,2	0,28	0,077	0,057
0,214	0,114	0,11	0,114	0,187	0,191	0,297	0,242	0,131
0,388	0,153	0,11	0,06	0,128	0,162	0,293	0,1	0,06
0,277	0,174	0,159	0,113	0,293	0,303	0,296	0,114	0,1

PC.ae.C32.1	PC.ae.C32.2	PC.ae.C34.0	PC.ae.C34.1	PC.ae.C34.2	PC.ae.C34.3	PC.ae.C36.0	PC.ae.C36.1	PC.ae.C36.2
2,02	0,459	0,97	6,53	8,05	5,17	0,446	5,1	8,86
1,52	0,373	0,882	6,72	8,39	5,14	0,38	4,74	7,94
2,06	0,402	0,829	6,82	8,33	4,67	0,416	4,7	9,49
3,37	0,776	1,47	11,5	16,7	10,1	0,62	7,85	16,1
2,77	0,608	0,707	8,53	8,49	6,71	0,407	4,4	8,85
3,05	0,767	1,31	11,3	13,3	8,25	0,73	7,28	13,2
1,5	0,509	0,718	7,21	8,4	4,21	0,387	5,75	11,3
2,7	0,84	1,09	8,43	8,81	5,76	0,664	6,71	11,4
2,33	0,598	1,16	9,32	12,6	7,64	0,663	6,61	15,4
1,58	0,346	0,523	5,7	5,84	4,6	0,402	3,44	6,53
3,16	0,772	0,963	10,3	16,6	9,77	0,623	5,6	13,5
2,09	0,508	1,02	7,64	14	10,1	0,434	5,25	10,6
2,23	0,502	1,1	8,38	11,8	8,19	0,402	7,24	12,6
2,41	0,61	1,16	9,64	8,68	5,29	0,664	6,38	9,61
2,4	0,603	1,13	9,39	12,3	6,57	0,687	6,71	12
2,85	0,671	1,14	10,7	12,8	7,65	0,806	6,19	11
1,93	0,475	0,97	7,97	9,09	4,65	0,479	6,01	10,4
3,18	0,708	1,12	10,3	10,8	7,31	0,546	7,44	9,08
3,93	0,836	1,53	11	15,3	10,7	0,686	7,26	14,1
2,25	0,486	0,756	7,94	10	7,09	0,457	4,57	8,89
1,58	0,452	0,7	6,28	8,9	4,96	0,462	5,14	11
2,16	0,452	0,684	6,59	9,08	6,18	0,483	3,85	7,78
2,6	0,648	2,07	11,2	13,7	5,98	0,68	8,36	13
2,58	0,585	1,07	9,86	11,1	7,35	0,586	5,68	10,1
2,3	0,507	0,79	7,73	8,16	5,49	0,55	4,6	8,7
1,53	0,454	0,799	5,71	5,52	4,82	0,571	4,22	7,16
2,96	0,701	1,19	8,38	12,2	8,08	0,559	5,81	12,1
1,85	0,454	0,652	7,08	7,69	5,41	0,478	3,91	7,65
1,96	0,446	1,04	7,96	7,08	5,3	0,636	4,76	6,94
2,95	0,737	1,43	10,8	12,2	11,2	0,951	7,57	12,6
1,97	0,601	0,997	7,99	10,5	7,31	0,615	5,85	11,7
2,14	0,478	0,788	9,06	11,9	6,66	0,472	4,46	8,49
2,17	0,574	0,936	8,68	15,2	7,84	0,455	6,46	13,3
2,85	0,73	1,47	9,6	17,3	8,92	0,607	6,61	12,8
2,35	0,592	1,05	11,4	12,9	6,44	0,411	7,01	11,4
2,96	0,747	0,867	9,78	14,6	9,19	0,493	5,43	12,7
1,72	0,4	0,641	5,7	6,67	4,66	0,45	3,82	6,74
2,15	0,434	0,758	6,69	8,16	4,36	0,358	4,58	9,03
1,47	0,327	0,499	5,98	8,45	5,09	0,411	4,02	6,8
2,09	0,536	0,918	7,16	9,58	6,46	0,579	5,14	10,9
1,89	0,577	1,05	6,87	9,68	6,88	0,302	5,9	10,7
1,25	0,421	0,559	5,96	6,99	3,68	0,304	5,08	10,1
1,76	0,439	0,809	5,87	7,36	4,41	0,298	4,56	7,98
1,49	0,368	0,47	5,21	5,09	4,03	0,363	2,96	5,56
2,67	0,718	1,02	10,4	12,4	7,14	0,64	6,09	10,4
1,9	0,483	0,755	8,07	11,5	5,65	0,412	4,54	10,5

2,16	0,556	1,03	11	12,6	6,28	0,334	7,03	11,2
1,75	0,435	0,922	7,73	8,08	4,44	0,462	5,99	9,81
2,73	0,552	1,11	9,74	11,8	7,08	0,517	5,86	11,6
2,18	0,48	0,702	8,37	11,5	6,17	0,329	4,54	7,79
1,96	0,482	0,868	7,67	10,3	6,29	0,403	5,21	12,7
1,48	0,428	0,688	5,31	5,61	4,43	0,476	3,89	6,6
2,4	0,556	0,823	8,28	9,75	7,34	0,482	5,04	10,3
2,32	0,671	1,63	10,4	13,1	6,04	0,589	7,26	11,8
3,92	0,778	1,22	10	13,9	10	0,659	6,91	13,5
1,63	0,431	0,571	6,5	7,41	5,2	0,359	3,76	6,89
1,49	0,35	0,351	3,23	3,84	1,92	0,226	2,08	2,43
2,44	0,602	0,982	7,97	8,51	5,57	0,459	5,66	10
1,86	0,408	0,536	5,65	7,8	5,04	0,357	3,2	6,53
1,85	0,405	1	7,19	6,55	5,39	0,502	4,37	6,49
2,76	0,676	1,29	10,6	11,9	8,04	0,597	6,11	11
2,86	0,711	1,24	8,89	15,9	8,37	0,575	6,28	12,1
2,95	0,668	1,02	8,41	12,2	8,69	0,473	5,7	11,9
2,66	0,625	0,916	8,46	8,97	6,37	0,535	6,01	7,34
2,51	0,499	0,734	8,13	9,8	7,51	0,472	4,64	9,02
1,87	0,397	0,597	6,69	7,04	4,87	0,421	3,68	7,29
2,27	0,543	0,989	9,52	12,5	6,56	0,448	6,44	11,4
3,17	0,715	1,54	11,5	13	11,5	0,949	7,36	12,4
1,88	0,501	0,713	8,15	9,49	5,31	0,404	4,66	9,39
2,39	0,47	0,647	6,76	7,34	5,71	0,349	3,78	7,1
1,42	0,398	0,593	5,53	7,85	4,58	0,345	4,51	9,85
1,68	0,458	0,777	5,99	12,2	8,45	0,308	4,17	9,13
1,42	0,333	0,841	6,47	8,06	4,88	0,423	4,3	6,94
1,85	0,494	0,817	6,63	12,3	6,19	0,349	5,38	10,5
2,26	0,629	1,13	9,15	8,42	5,55	0,513	6,04	8,84
1,87	0,656	1,28	8,35	7,44	5,11	0,47	10,1	8,97
1,72	0,418	0,829	7,22	7,13	5,56	0,5	5,57	8,56
2,25	0,36	0,726	5,82	6,54	3,75	0,355	4,54	5,32
1,56	0,351	0,645	5,92	6,82	3,76	0,236	5,39	7,75
1,85	0,423	0,932	6,64	8,1	4,69	0,322	4,83	7,67
2,82	0,762	1,48	11,2	12,6	7,03	0,495	9	12,5
2,44	0,531	0,886	7,43	9,82	4,1	0,443	5	7,17
1,83	0,45	0,735	7,26	7,23	4,96	0,358	6,95	9,45
2,11	0,47	0,85	7,77	7,74	4,73	0,425	6,57	8,76
1,5	0,356	0,668	6,96	10,7	5,21	0,551	4,65	7,5
1,86	0,467	0,767	6,94	8,87	5,23	0,457	5,49	8,35
2,66	0,709	2,16	10,4	7,15	3,89	0,607	8,81	8,69
1,16	0,29	0,558	5,09	4,23	2,46	0,44	5,34	5,09
2,41	0,596	1,08	8,1	7,58	4,02	0,49	6,25	8,38
3,46	0,852	0,899	8,88	8,52	3,68	0,549	6,36	7,26
1,47	0,441	0,694	5,89	5,36	2,67	0,251	5,11	7,28
1,3	0,293	0,717	5,28	4,69	1,72	0,277	5,73	6,44
1,14	0,244	0,721	4,5	2,46	1,12	0,515	4,01	3,83
1,18	0,301	0,482	4,14	2,75	1,37	0,369	4,32	4,14
1,18	0,384	0,747	5,88	5,34	2,54	0,177	7,95	6,86
2,7	0,558	1,29	9,34	11,8	7,2	0,479	6,95	10,6

2,24	0,599	1,26	7,74	11,4	5,4	0,441	9,03	11,3
1,55	0,3	0,62	6,88	3,81	1,55	0,296	4,86	5,52
1,89	0,457	0,814	6,55	8,51	6,61	0,66	8,97	7,36
1,69	0,369	0,619	6,32	4,77	2,7	0,322	4,88	5,66
1,24	0,431	0,92	5,69	4,98	2,58	0,235	8,01	7,88
0,874	0,2	0,363	3,36	3,17	2,41	0,262	3,71	4,64
1,27	0,372	0,497	4,24	3,79	2,26	0,262	6,09	4,19
0,857	0,221	0,328	3,47	2,64	1,65	0,276	2,91	2,99
1,64	0,392	0,741	5,42	7,52	3,28	0,427	5,71	6,98
1,44	0,353	0,82	6,04	6,07	2,88	0,332	6,55	8,06
1,08	0,295	0,502	4,37	4,08	4,09	0,292	4,2	3,9
2,86	0,633	1,23	8,95	9,57	5,77	0,513	8,83	10,4
0,899	0,199	0,45	4,49	2,61	1,22	0,217	3,3	3,03
1,66	0,489	0,902	7,21	7,33	3,95	0,33	7,96	7,72
1,48	0,434	0,691	5,47	8,39	4,6	0,314	4,97	7,68
1,73	0,451	0,908	8,63	9,38	5,53	0,297	10,1	10,8
1,31	0,339	0,291	4,68	3,88	2,25	0,183	4,1	3,86
1,56	0,399	0,733	7,67	11,3	5,2	0,566	5,97	8,09
2,75	0,668	0,914	10	11,6	6,5	0,682	5,92	12,6
1,82	0,41	0,627	6,55	7,49	4,85	0,507	4,04	7,56
2,51	0,486	0,877	9,05	10,3	7,57	0,545	5,44	11
1,8	0,561	0,681	7,15	9,67	5,67	0,465	5,08	8,24
1,41	0,372	0,751	6,88	9,47	4,75	0,342	3,74	8,51
1,6	0,458	0,922	7,62	7,29	5,49	0,583	5,8	9

PC.ae.C36.3	PC.ae.C36.4	PC.ae.C36.5	PC.ae.C38.0	PC.ae.C38.1	PC.ae.C38.2	PC.ae.C38.3	PC.ae.C38.4	PC.ae.C38.5
4,88	12,9	8,31	1,31	0,136	1,02	2,38	8,63	11,1
4,27	11,8	7,59	1,38	0,158	0,7	1,54	6,24	10,6
4,77	11,6	6,26	0,942	0,051	0,915	2,21	8,56	10,1
8,59	19,1	11,1	1,22	0,015	1,58	2,43	11,5	15,9
5,61	17	10,5	1,17	0,396	0,877	2,12	11,8	17,1
6,37	15,7	9,56	1,28	0,219	1,26	2,46	9,24	13,5
4,31	7,53	5,17	1,88	0	1,01	2,33	6,62	8,61
4,23	11,3	8,36	1,45	0,309	0,899	2,39	8,15	10,8
7,25	12,9	8,65	1,5	0,198	1,32	3,34	9,44	12,5
3,85	7,24	5,41	0,854	0,119	0,776	1,5	6,02	9,94
9,73	20,1	10,5	1,11	0,321	1,12	2,96	10,2	18,1
7,81	20,4	12,8	1,05	0	0,634	2,2	9,14	15,4
6,21	14,5	9,47	1,55	0	1,15	3,01	10,1	11,5
4,59	13,9	9,21	1,54	0,276	0,667	2,69	9,43	12,3
7,26	23,4	12	1,59	0,102	1	2,72	10,9	18,1
7,45	15,7	8,89	1,81	0,13	1,03	2,13	8,86	15,5
4,18	12,2	7,27	1,33	0,071	0,974	1,9	6,61	10,2
5,77	20,6	14	2,37	0	0,862	2,54	9,3	14,7
8,67	28,8	16,3	1,72	0	1,47	3,33	16,3	22,5
6,36	12,4	7,47	1,26	0,172	0,895	2,26	6,97	11,9
4,35	10,6	5,99	1,29	0	0,916	2,06	6,18	9,39
4,93	11,7	6,35	0,983	0,068	0,635	1,91	7,23	10,9
6,17	20,6	13	1,41	0	0,847	2,76	13,1	17,9
5,79	15,4	10,1	1,05	0,171	0,669	2,3	10,1	14,9
4,38	9,62	5,4	1,24	0,129	1,09	1,54	6,45	9,85
3,07	8,23	7,83	1,51	0	0,434	1,06	4,48	8,98
5,77	17,1	13,8	1,64	0,05	1,03	2,42	9,24	14,2
4,74	11,7	7,23	1,22	0,271	0,782	2,18	7,65	11,6
4,14	11,9	6,93	1,34	0,159	1	1,92	7,93	11,4
6,32	16,9	14,6	1,78	0,313	1,28	2,4	10,8	16,7
6,53	17	13,2	1,8	0	1	2,19	10,6	18,1
6,84	20,2	9,94	1,65	0,052	0,611	2,39	10,1	15,8
8,54	18,5	9,2	1,36	0	1,12	2,93	11,2	15,7
9,77	24,2	13,7	1,58	0,37	1,32	2,91	13,2	19,1
6,63	14	7,53	2,08	0,245	1,04	2,98	9,52	13,5
9,18	18,9	9,83	1,21	0,212	1,5	3,08	10,1	17,7
3,75	13,2	7,97	1,69	0,135	0,721	1,61	8,41	14,4
4,61	11,6	6,07	0,895	0,059	0,931	2,21	9,2	11,2
4,8	12,9	6,81	0,971	0	0,56	1,56	7,45	12,5
6,13	16,2	11,7	1,84	0,071	1,1	2,22	9,96	16,9
5,13	12,6	8,14	1,47	0	1,02	2,67	8,96	10,5
3,67	6,71	4,69	1,63	0,029	0,811	2,03	6,17	8,32
4,2	11,7	7,26	1,27	0,223	0,711	2,17	8,62	10,8
3,49	6,28	4,65	0,751	0,172	0,713	1,5	5,38	9,26
7,13	14,9	8,29	1,73	0,351	1,23	2,22	8,58	15,2
6,68	11,4	5,19	0,982	0,16	1,12	2,27	7,34	11,7

6,68	13,9	7,78	2,26	0,015	1,18	2,89	9,76	13,9
4,07	11,3	6,77	1,21	0,137	0,954	1,96	6,79	10,2
5,55	13,9	8,56	1,25	0,05	0,93	2,13	8,74	13
6,5	19,9	9,25	1,69	0	0,547	2,66	9,99	15,8
5,95	10,5	6,97	1,22	0	1,22	3,08	8,47	11,2
2,68	7,81	7,52	1,51	0	0,559	1,17	4,88	9,9
5,94	13,1	7,18	0,886	0,272	1,13	2,31	8,64	12,9
5,67	18,6	11,7	1,33	0,226	1,06	2,62	12,7	17,4
8,05	26,2	15,7	1,53	0,119	1,43	3,04	16,5	22,3
4,52	11,5	6,77	1,11	0,184	1,04	2,14	7,37	11,6
2,1	9,05	5,37	0,522	0,361	0,623	1,76	4,28	7,47
4,19	10,4	8,03	1,35	0	0,863	2,25	8,12	10,8
4,4	10,2	5,67	0,902	0,22	0,848	1,62	6,49	10,1
4,26	11,2	6,97	1,25	0,072	0,738	2,03	7,66	10,6
6,43	16,6	10,4	1,24	0,101	1,17	2,53	11,3	16,1
8,74	22,5	12,9	1,54	0,363	1,46	2,91	12,3	19,1
5,78	17,1	13,6	1,5	0,185	0,916	2,49	9,86	15,1
4,83	17,2	12,2	2,06	0	0,649	2,19	7,97	13
6,32	12,8	7,84	1,29	0	1,05	2,3	7,59	12,4
3,64	8,12	4,33	1,04	0	0,811	1,48	5,68	7,96
6,71	22,7	11,4	1,5	0,484	0,777	2,65	11	17,5
6,23	17,2	14,8	1,9	0,246	1,43	2,29	11,2	16,8
5,31	10,8	7,01	1,3	0,276	1,12	1,93	7,35	13,3
4,46	13,3	8,66	0,983	0,165	0,762	1,75	10,8	15,8
3,93	9,39	5,51	1,22	0,053	0,801	1,73	5,96	8,79
6,57	17,9	10,8	0,915	0,13	0,827	1,76	8,3	14
4,1	10,9	6,92	1,42	0,068	0,516	1,59	6,08	10,7
6,84	15,4	7,29	1,14	0	1,22	2,28	9,52	13
4,48	13,3	8,44	1,36	0,258	0,76	2,51	9,04	11,7
4,19	16,2	10,3	2,39	1,99	2,8	11,9	12,9	15,3
4,66	7,6	4,88	1,65	0	1,04	2,09	6,07	7,6
3,88	14,1	9,12	0,868	0,754	1,48	3,73	7,8	13,6
3,77	11,8	6,69	1,09	0,867	1,57	3,59	7,68	12,2
4,11	15,8	9,85	0,964	0	0,968	2,72	10,6	14,9
6,2	16,5	8,12	1,73	1,23	2,06	5,15	12,7	15,2
6,19	12,8	7,22	0,742	0,656	1,16	3,11	8,64	13,8
4,61	8,6	4,96	1,14	0,536	1,79	3,83	7,06	8,62
4,94	14,5	8,59	1,21	0,852	1,92	4,76	9,9	15,9
7,69	20,5	7,61	0,549	0,453	1,32	3,55	10	16
4,59	16	9,77	1,66	0	0,678	2,37	10,7	16,6
4,15	17	12,6	2,58	0,301	1,35	5,43	14,2	15,8
2,56	6,85	4,72	1,42	0,809	1,44	3,35	4,89	7,68
4,79	16,6	12,9	1,69	0	0,842	3,42	11,4	17,3
4,92	18	11,7	1,91	1,38	1,95	5,96	9,37	18,6
2,46	7,24	4,91	0,915	0,136	0,471	2,06	7,79	7,32
2,12	6,57	3,93	1,08	0,463	0,927	3,76	6,58	6,25
1,79	6,84	4,21	0,73	0,382	0,928	2,95	6,19	7
1,64	5,87	4,65	0,679	0,43	0,973	3,58	4,73	6,43
3,35	9,29	5,12	1,19	1,49	2,97	8,79	8,33	8,65
6,3	16,6	10,2	1,62	0,251	1,22	3,69	10,8	14,9

6,37	18,1	12,2	1,79	1,36	2,69	8,4	12,1	16
2,87	9,76	4,67	0,884	0,612	1,78	5,22	8,37	10
4,29	13,8	9,21	1,18	2,81	2,68	8,6	8,95	13,9
2,95	9,83	7,03	0,955	0,712	1,46	3,34	6,79	12,2
2,78	9,48	6,42	1,38	1,68	2,95	8,49	9,97	9,63
1,86	5,04	4,21	0,975	0,235	1,17	2,56	3,71	6,08
2,36	8,26	7,64	2,23	1,64	2,76	7,77	7,9	9,87
1,64	6,58	4,1	0,487	0,573	0,889	3,02	3,98	7,39
3,92	13,5	8,55	1,51	0,802	1,33	4,73	9,26	13,4
3,42	10,5	6,5	1,27	1,27	1,86	5,87	7,85	9,92
2,86	8,09	5,21	0,775	0,851	1,63	3,4	5,18	7,06
5,73	18	10,9	2,04	1,58	3,18	8,1	12,3	16,3
2,09	3,68	1,84	0,483	0,392	1,15	2,39	3	3,79
4,04	14,5	7,68	1,45	1,4	2,59	8,82	10,8	13,4
4,26	14,2	8,85	1,35	0,263	1,45	4,19	8,14	12,1
5,16	9,7	6,4	1,08	1,9	2,64	6,98	7,85	11,3
2,07	5,98	4,57	0,717	0,633	1,28	3,79	4,36	7,45
8,12	22,4	8,14	0,503	0,936	2,01	4,86	10,8	17,1
7,08	14,7	9,05	1,56	0,604	1,61	2,34	8,74	16,3
4,39	15	8,99	1,75	0,087	0,973	1,72	8,24	15,1
6,52	14	7,98	0,908	0,208	1,18	2,3	9,11	13,1
5,35	14,8	7,32	1,16	0,088	0,553	1,64	8,46	13,3
5,38	9,03	4,2	0,794	0,039	0,762	1,82	5,49	8,81
4,45	7,84	4,81	1,77	0	1,04	2,06	5,73	7,42

PC.ae.C38.6	PC.ae.C40.1	PC.ae.C40.2	PC.ae.C40.3	PC.ae.C40.4	PC.ae.C40.5	PC.ae.C40.6	PC.ae.C42.0	PC.ae.C42.1
4,87	0,778	0,948	0,571	1,36	2,06	2,81	0	0,173
4,59	0,776	0,893	0,403	0,866	1,49	2,28	0	0,121
3,77	0,685	0,839	0,579	1,33	1,87	2,38	0	0,15
5,68	0,887	1,11	0,588	1,79	2,69	2,87	0	0,17
6,31	0,641	0,939	0,659	2,05	3,08	3,95	0	0,184
6,21	0,965	1,67	0,74	1,54	2,12	3,41	0	0,185
4,03	0,717	1,19	0,531	1,07	1,48	2,8	0,591	0,201
5,1	0,906	1,81	0,68	1,3	2,02	2,82	0,481	0,261
5,13	0,844	1,66	0,744	1,47	2,08	3,24	0,564	0,228
2,99	0,577	0,632	0,455	1,15	1,72	1,88	0	0,143
6,37	0,818	1,46	0,879	1,42	2,52	2,89	0	0,226
5,53	0,591	1,21	0,533	1,25	1,9	2,49	0	0,124
5	0,67	1,64	0,73	1,42	1,94	2,61	0	0,184
5,33	0,79	1,14	0,617	1,49	2,14	2,79	0	0,219
7,74	0,788	1,44	0,638	1,42	2,19	3,34	0	0,154
6,7	0,967	1,22	0,627	1,34	2,14	3,2	0	0,219
4,65	0,558	0,863	0,459	0,859	1,43	1,96	0	0,118
8,15	1,34	1,34	0,615	1,45	2,03	3,27	0,493	0,272
9,27	1,19	1,71	0,889	2,54	3,36	4,86	0,396	0,229
4,64	0,648	0,872	0,559	1,14	1,78	1,96	0	0,186
4,82	0,712	1,45	0,46	0,858	1,39	2,8	0,472	0,161
4,05	0,55	0,924	0,44	1,15	1,77	2,19	0	0,122
6,79	0,943	1,77	0,547	1,38	2,22	2,82	0	0,207
5,45	0,776	1,24	0,619	1,46	2,34	2,8	0	0,174
4,12	0,712	0,819	0,53	1,22	1,57	2,46	0,388	0,15
5,71	0,614	1,12	0,412	0,794	1,4	2,63	0	0,121
6,69	0,72	1,45	0,581	1,32	2,09	2,99	0	0,183
4,09	0,979	0,903	0,575	1,31	1,88	1,99	0	0,207
3,89	0,826	0,926	0,568	1,3	1,95	1,96	0	0,179
7,84	1,16	1,62	0,781	1,8	3,06	4,17	0,473	0,263
8,05	0,917	1,19	0,542	1,57	2,53	4,07	0,441	0,162
8,07	0,811	1,16	0,628	1,39	2,2	3,57	0,459	0,189
5,8	0,774	1,39	0,586	1,66	2,1	2,55	0	0,167
8,92	0,998	1,31	0,793	2	2,89	4,02	0	0,208
7,22	0,89	1,19	0,77	1,51	2,44	4,46	0,61	0,231
6,09	0,788	1,56	0,883	1,54	2,45	2,94	0,344	0,18
5,56	0,878	1,22	0,481	1,39	2,37	3,25	0,382	0,211
4,14	0,701	0,928	0,615	1,42	1,89	2,53	0,283	0,158
3,9	0,547	0,781	0,433	1,08	1,67	2,06	0,249	0,156
7,75	0,916	1,21	0,597	1,55	2,38	3,98	0,363	0,193
4,52	0,611	1,44	0,621	1,28	1,77	2,43	0,284	0,141
3,64	0,599	1,06	0,465	0,989	1,48	2,5	0,504	0,197
4,69	0,732	0,958	0,463	1,21	2,07	2,9	0,302	0,151
2,62	0,526	0,634	0,414	1,04	1,59	1,81	0,315	0,106
6,5	1,07	1,23	0,778	1,34	2,34	3,21	0,318	0,24
4,45	0,541	1,16	0,785	1,35	2,16	2,68	0,307	0,15

7,42	0,836	1,37	0,757	1,63	2,67	5	0,578	0,201
4,57	0,58	0,87	0,494	0,856	1,34	2,14	0,285	0,105
6,04	0,647	1,5	0,61	1,27	1,96	3,2	0,27	0,14
7,67	0,753	1,2	0,569	1,45	2,33	3,77	0,459	0,214
4,73	0,72	1,52	0,663	1,22	1,8	2,99	0,515	0,198
5,52	0,657	1,16	0,42	0,776	1,47	2,75	0,32	0,113
4,15	0,567	0,788	0,709	1,67	2,57	2,23	0,282	0,209
6,33	0,97	1,77	0,592	1,33	2,18	2,86	0,25	0,198
8,87	1,13	1,76	0,8	2,53	3,33	4,91	0,332	0,24
4,12	0,781	0,825	0,488	1,29	1,98	2,05	0,287	0,124
2,87	0,279	0,55	0,789	0,905	1,47	1,34	0,256	0,123
4,67	0,726	1,71	0,609	1,22	1,94	2,9	0,356	0,163
3,53	0,575	0,746	0,453	1,09	1,71	2,06	0,277	0,148
3,97	0,776	0,897	0,539	1,33	2,08	2,03	0,25	0,182
5,94	0,678	1,42	0,77	1,48	2,75	3,38	0,328	0,187
8,7	0,961	1,27	0,771	1,9	3,03	3,85	0,348	0,192
7,32	0,678	1,42	0,621	1,41	2,14	3,25	0,318	0,166
7,1	1,15	1,18	0,543	1,29	1,88	2,87	0,398	0,247
5,11	0,64	1,1	0,632	1,16	1,82	2,33	0,303	0,134
3,44	0,568	0,74	0,433	0,926	1,52	2,25	0,234	0,157
7,59	0,866	1,4	0,561	1,45	2,04	3,44	0,289	0,201
7,93	1,09	1,76	0,888	1,99	3,18	4,82	0,459	0,182
4,51	0,786	0,986	0,602	1,48	2,36	2,36	0,323	0,207
5,81	0,47	0,731	0,578	1,63	2,72	3,34	0,344	0,133
4,59	0,586	1,48	0,479	0,785	1,37	2,84	0,365	0,113
5,26	0,436	1,03	0,456	1,09	1,66	2,01	0,239	0,129
4,54	0,692	0,853	0,403	0,854	1,39	2,27	0,337	0,134
4,81	0,633	1,24	0,599	1,26	1,86	2,3	0,297	0,171
5,09	0,714	1,03	0,618	1,31	1,96	2,74	0,322	0,183
6,47	1,31	2,42	6,26	4,8	8,2	3,6	0,471	0,57
3,28	0,804	1,14	0,597	1,09	1,77	2,11	0,365	0,196
4,14	0,607	1,05	1,65	1,75	2,65	1,88	0,287	0,187
4,29	0,558	1,15	1,46	1,7	2,95	2,41	0,283	0,168
4,53	0,602	1,37	0,811	1,65	2,58	2,5	0,295	0,178
5,53	1,21	1,39	2,1	2,51	4,21	3,65	0,369	0,318
3,86	0,495	0,995	0,962	1,48	2,33	1,94	0,242	0,221
3,48	0,549	1,37	1,38	1,62	2,55	2,35	0,346	0,187
5,21	0,869	1,45	2,13	2,36	3,71	2,8	0,374	0,297
3,95	0,508	0,989	1,02	1,41	1,97	1,61	0,282	0,18
6,1	0,858	1,57	0,879	1,77	3,09	3,71	0,493	0,214
9,14	1,44	1,91	2,09	2,68	6,14	6,43	0,372	0,279
3,56	0,605	1,01	1,48	1,48	2,99	1,94	0,35	0,153
7,9	0,645	2,08	0,93	1,61	2,55	4,11	0,496	0,224
9,81	0,73	2,02	2,89	2,76	6,33	4,37	0,521	0,314
3,1	0,34	1,04	0,468	0,986	1,42	2,16	0,286	0,149
3,23	0,358	1,05	1,45	1,51	3,32	2,45	0,236	0,126
2,26	0,693	1,01	1,19	1,24	2,04	1,55	0,25	0,2
2,5	0,265	1,04	1,41	1,41	2,43	1,32	0,259	0,139
3,5	0,734	1,91	3,82	2,69	4,12	1,92	0,339	0,337
5,16	0,983	1,4	0,995	1,65	2,51	2,55	0,362	0,271

6,8	0,892	2,61	3,48	2,88	4,79	3,31	0,363	0,293
2,69	0,348	1,31	1,86	2,05	3,42	1,94	0,305	0,221
4,65	0,865	1,51	4,9	3,42	6,66	2,6	0,361	0,443
4,1	0,552	1,05	1,61	1,59	3,19	2,25	0,374	0,129
3,57	0,779	2,49	5,5	3,51	5,85	2,57	0,35	0,396
2,34	0,544	0,785	0,913	1,2	2,2	1,51	0,312	0,198
5,52	0,721	1,84	4,48	4,99	8,74	2,89	0,374	0,292
2,11	0,243	0,985	1,49	1,12	1,97	1,14	0,244	0,122
5,95	0,805	1,65	2,01	2,49	4,08	3,18	0,353	0,265
4,05	0,641	1,56	2,49	2,07	4,79	2,51	0,282	0,215
2,49	0,671	1,22	1,86	1,69	2,28	1,39	0,34	0,213
6,7	1,27	2,32	3,39	3,76	6,44	3,71	0,497	0,416
1,2	0,262	0,542	0,713	0,92	1,23	0,841	0,2	0,132
4,3	0,846	1,91	4,45	3,9	5,66	2,6	0,387	0,429
5,19	1,05	1,12	1,69	1,77	3,2	2,55	0,452	0,38
3,7	0,804	1,37	2,83	2,46	4,81	2,36	0,286	0,303
2,52	0,331	0,647	1,65	1,41	2,86	1,35	0,275	0,154
3,96	0,519	1,11	1,67	1,72	2,32	1,79	0,295	0,26
5,71	1,02	1,14	0,753	1,93	2,78	3	0,452	0,187
6,17	0,756	1,16	0,542	1,61	2,39	3,46	0,442	0,172
4,08	0,65	0,741	0,735	1,88	2,4	2,53	0,288	0,187
4,26	0,714	0,887	0,51	1,21	1,63	2,16	0,351	0,241
3,46	0,498	0,809	0,6	0,979	1,57	2	0,276	0,108
3,09	0,968	1,15	0,563	1,05	1,53	2,05	0,371	0,213

PC.ae.C42.2	PC.ae.C42.3	PC.ae.C42.4	PC.ae.C42.5	PC.ae.C44.3	PC.ae.C44.4	PC.ae.C44.5	PC.ae.C44.6
0,29	0,488	0,518	1,22	0,082	0,211	1,06	0,708
0,256	0,336	0,346	0,938	0,035	0,114	0,869	0,483
0,269	0,411	0,588	1,33	0,053	0,265	1,24	0,675
0,391	0,584	0,701	1,69	0,059	0,258	1,66	0,887
0,383	0,628	0,71	2,58	0,076	0,394	2,38	1,46
0,364	0,573	0,582	1,42	0,052	0,209	1,31	0,839
0,304	0,421	0,443	1,1	0,099	0,165	1,07	0,61
0,326	0,552	0,449	1,37	0,096	0,285	1,3	0,74
0,307	0,421	0,518	1,33	0,075	0,222	1,08	0,67
0,286	0,355	0,534	1,46	0,063	0,218	1,25	0,733
0,358	0,555	0,709	1,78	0,089	0,335	1,51	0,918
0,283	0,327	0,461	1,24	0,054	0,214	1,36	0,769
0,334	0,449	0,517	1,12	0,073	0,272	0,919	0,677
0,303	0,447	0,46	1,42	0,048	0,232	1,17	0,785
0,321	0,481	0,52	1,33	0,066	0,231	1,1	0,595
0,484	0,691	0,713	1,76	0,086	0,305	1,72	1,02
0,257	0,222	0	0,874	0,042	0,116	0,694	0,423
0,57	0,629	0,433	1,22	0,077	0,279	1,16	0,755
0,456	0,63	1,07	2,32	0,103	0,421	1,99	1,34
0,235	0,443	0,472	1,28	0,074	0,209	1,16	0,646
0,246	0,387	0,391	0,96	0,06	0,196	0,787	0,598
0,274	0,416	0,461	1,51	0,042	0,22	1,22	0,752
0,347	0,366	0	1,14	0,043	0,178	0,818	0,481
0,338	0,453	0,604	1,72	0,071	0,283	1,57	0,936
0,29	0,445	0,591	1,35	0,096	0,242	1,22	0,841
0,321	0,314	0,335	0,922	0,042	0,148	0,526	0,542
0,295	0,467	0,54	1,36	0,071	0,216	1,13	0,77
0,34	0,471	0,559	1,47	0,094	0,324	1,42	0,775
0,305	0,425	0,558	1,47	0,077	0,206	1,23	0,678
0,558	0,722	0,724	2,09	0,111	0,286	1,98	1,34
0,353	0,478	0,512	1,55	0,058	0,2	1,19	0,755
0,251	0,424	0,499	1,7	0,036	0,271	1,23	0,815
0,296	0,415	0,561	1,27	0,052	0,198	1,04	0,619
0,394	0,583	0,744	2,18	0,055	0,29	1,72	1,16
0,389	0,58	0,704	1,77	0,082	0,286	1,83	0,94
0,294	0,552	0,689	1,71	0,059	0,302	1,66	0,955
0,324	0,486	0,587	1,65	0,089	0,308	1,5	0,972
0,3	0,43	0,666	1,42	0,051	0,299	1,35	0,755
0,203	0,248	0,405	0,99	0,034	0,141	0,865	0,591
0,28	0,494	0,469	1,47	0,04	0,215	1,16	0,707
0,291	0,371	0,478	1,01	0,034	0,22	0,951	0,578
0,277	0,392	0,367	1,08	0,06	0,178	0,864	0,621
0,288	0,439	0,53	1,14	0,045	0,243	0,99	0,578
0,228	0,351	0,452	1,27	0,058	0,214	1,13	0,686
0,478	0,806	0,74	1,83	0,08	0,377	1,81	0,967
0,324	0,726	0,724	1,78	0,099	0,395	1,87	1,04

0,354	0,637	0,68	1,99	0,065	0,327	2,06	1,04
0,21	0,301	0,276	0,89	0,013	0,152	0,704	0,403
0,345	0,443	0,533	1,36	0,046	0,239	1,29	0,817
0,292	0,515	0,485	1,76	0,066	0,207	1,46	0,884
0,256	0,422	0,424	1,23	0,066	0,213	0,952	0,592
0,26	0,301	0,297	0,937	0,054	0,127	0,484	0,573
0,377	0,561	0,863	1,91	0,069	0,409	2,03	1,1
0,307	0,346	0,328	1,11	0,053	0,141	0,724	0,462
0,5	0,684	1,07	2,24	0,054	0,423	2,05	1,31
0,368	0,431	0,559	1,42	0,078	0,228	1,36	0,787
0,14	0,156	0,243	1,11	0,043	0,131	0,491	0,342
0,281	0,434	0,417	1,22	0,077	0,235	1,3	0,76
0,24	0,379	0,419	1,32	0,043	0,254	1,08	0,702
0,293	0,401	0,493	1,43	0,071	0,186	1,29	0,656
0,336	0,561	0,64	1,96	0,079	0,309	1,78	1,02
0,389	0,529	0,772	2,03	0,081	0,273	1,75	1,05
0,26	0,399	0,515	1,34	0,07	0,226	1,37	0,84
0,492	0,576	0,47	1,18	0,057	0,2	1,03	0,731
0,238	0,411	0,517	1,39	0,043	0,276	1,19	0,698
0,197	0,43	0,469	1,23	0,053	0,214	1,03	0,691
0,314	0,432	0,483	1,46	0,063	0,194	1,1	0,67
0,513	0,73	0,774	2,22	0,107	0,303	1,99	1,37
0,342	0,557	0,663	1,75	0,069	0,248	1,57	1
0,337	0,532	0,813	2,04	0,079	0,297	2,07	1,15
0,223	0,361	0,282	0,9	0,047	0,129	0,755	0,572
0,221	0,304	0,353	1,19	0,057	0,169	1,18	0,665
0,248	0,38	0,277	0,899	0,051	0,116	0,763	0,544
0,229	0,367	0,527	1,13	0,065	0,219	0,894	0,527
0,265	0,372	0,504	1,4	0,081	0,168	1,22	0,704
0,519	0,757	1,02	2,69	0,221	0,211	0,825	0,572
0,333	0,569	0,546	1,14	0,084	0,292	1,14	0,731
0,188	0,294	0,442	1,41	0,055	0,141	0,724	0,537
0,216	0,353	0,423	1,57	0,04	0,164	0,854	0,6
0,271	0,31	0,476	1,41	0,055	0,118	1,03	0,691
0,479	0,612	0,778	2,03	0,09	0,257	1,3	0,743
0,25	0,307	0,439	1,21	0,053	0,199	0,914	0,606
0,27	0,484	0,67	1,44	0,106	0,218	0,951	0,766
0,303	0,404	0,558	1,75	0,096	0,23	1,13	0,71
0,296	0,286	0,403	0,861	0,036	0,165	0,585	0,403
0,406	0,466	0,539	1,9	0,083	0,24	1,79	1,06
0,56	0,829	0,974	3,25	0,165	0,332	1,63	1,08
0,258	0,315	0,337	1,25	0,104	0,103	0,365	0,265
0,278	0,344	0,407	1,46	0,045	0,192	1,01	0,702
0,392	0,593	0,795	3,31	0,128	0,251	1,5	1,05
0,152	0,268	0,298	0,87	0,045	0,136	0,759	0,407
0,192	0,259	0,465	1,51	0,064	0,144	0,698	0,551
0,245	0,327	0,393	1,29	0,048	0,12	0,614	0,394
0,199	0,217	0,415	1,27	0,049	0,135	0,6	0,459
0,209	0,415	0,713	2,07	0,096	0,206	0,807	0,491
0,3	0,401	0,521	1,3	0,078	0,19	1,04	0,661

0,342	0,502	0,611	1,93	0,105	0,185	0,692	0,56
0,22	0,298	0,67	1,75	0,086	0,172	1,08	0,618
0,379	0,595	0,825	2,63	0,149	0,192	0,725	0,53
0,182	0,319	0,43	1,43	0,063	0,12	0,666	0,431
0,381	0,496	0,957	2,76	0,109	0,172	0,673	0,477
0,205	0,317	0,235	0,918	0,054	0,103	0,411	0,355
0,388	0,555	1,06	3,94	0,179	0,134	0,544	0,485
0,149	0,206	0,245	1,06	0,054	0,082	0,555	0,403
0,412	0,536	0,521	1,97	0,107	0,158	1,16	0,746
0,304	0,464	0,581	2,15	0,109	0,193	0,898	0,552
0,236	0,337	0,57	1,36	0,106	0,133	0,536	0,347
0,604	0,869	1,06	2,77	0,154	0,309	1,55	0,888
0,147	0,197	0,264	0,739	0,05	0,107	0,38	0,247
0,529	0,566	0,909	2,41	0,144	0,193	1,14	0,59
0,363	0,526	0,487	1,53	0,12	0,171	0,693	0,615
0,333	0,554	0,754	1,98	0,105	0,179	1,08	0,664
0,135	0,227	0,324	1,26	0,081	0,08	0,633	0,404
0,323	0,352	0,504	0,953	0,052	0,155	0,533	0,354
0,467	0,611	0,834	2,14	0,101	0,294	1,93	1,13
0,327	0,505	0,639	1,81	0,064	0,226	1,56	0,956
0,326	0,546	0,891	1,9	0,057	0,424	2,06	1,12
0,254	0,362	0,456	1,15	0,078	0,176	0,887	0,638
0,253	0,526	0,512	1,42	0,04	0,367	1,54	0,764
0,294	0,574	0,467	1,17	0,092	0,236	1,16	0,747

SM..OH..C14.1	SM..OH..C16.1	SM..OH..C22.1	SM..OH..C22.2	SM..OH..C24.1	SM.C16.0	SM.C16.1	SM.C18.0
4,08	2,1	5,7	5,48	0,543	72,7	9,46	13,6
2,72	1,52	6,1	5,33	0,45	64,9	8,09	11,7
3,98	2,14	6,83	5,97	0,632	109	17,1	22,7
4,97	2,56	7,83	6,87	0,61	83,7	11,8	15,7
2,96	1,68	5	5,33	0,563	88,3	10,1	14,9
6,07	3,53	7,93	7,83	0,809	75,9	11,7	12,6
4,7	2,06	8,62	6,55	0,745	107	14,2	20,3
5,55	2,9	7,68	6,93	0,917	113	12,6	19
6,67	3,04	11	10,4	1,06	65,9	9,12	16,5
3,02	1,59	6	5,52	0,64	77,5	9,31	10,9
5,88	2,65	8,09	7,99	0,857	93	11,6	14,8
3,5	2,19	7,62	5,77	0,762	84,2	9,81	16,7
6,94	3,43	15	10,9	1,32	124	13	20,8
5,59	2,78	9,45	9,09	0,7	111	15,1	24,3
4,96	2,59	7,75	7,96	0,706	103	15,7	19
4,2	2,44	7,85	7,28	0,735	99,3	9,2	14,4
4,72	1,83	6,89	5,88	0,646	84,3	8,95	11,8
4,43	2,51	9,92	9,04	0,771	130	15,2	22,9
6,6	3,69	10,8	9,06	0,999	96	14,6	9,54
4,29	2	6,55	6,55	0,6	135	16	18,4
4,39	2,15	8,17	6,79	0,824	85,4	9,42	15
3,05	1,64	5,2	4,67	0,419	80,1	10	15,4
7,07	3,79	10,5	9,81	1,11	87	11,5	18,9
4,04	2,21	6,6	5,84	0,665	90,8	9,92	16,8
3,71	1,78	6,27	5,81	0,581	86,7	10,6	13,8
4,14	2,09	6,07	5,49	0,607	87,3	12,1	14,3
6,29	3,15	8,66	9,4	0,741	94	11,5	18,5
2,97	1,51	6,34	5,35	0,559	77,4	10,4	11,3
3,08	1,41	5,31	5,04	0,527	93,5	14,3	24
6	3,11	8,84	8,44	0,878	90,2	12,5	18,5
3,56	2,15	7,94	6,71	0,814	90,2	8,89	14,1
3,89	2,15	8,43	7,29	0,563	112	14,7	21,7
6,19	2,76	10,5	9,23	0,996	114	16,1	17,8
5,22	2,38	9,41	9,39	0,705	92,1	12,4	16,1
3,83	2,17	7,38	8,67	0,524	83,1	11,8	15,6
5,27	104	13,6	2,59	17,5	8,03	0,217	0
2,73	70,4	7,86	1,59	10,9	4,78	0,14	0
3,74	82	9,1	2,06	14,6	6,03	0,086	0
3,89	79	9,95	1,97	13,8	6,16	0,116	0
3,87	90,5	9,39	2,16	13,7	6,1	0,194	0
5,8	85	11,2	2,8	15,9	6,51	0,171	0
3,75	60,1	7,14	1,67	10,1	3,56	0,163	0
3,77	71	9,11	2,05	12,6	6,22	0,123	0
2,5	63,5	7,32	1,28	8,86	3,82	0,086	0
4,3	111	13,5	2,4	20,6	10,2	0,187	0
3,92	78,8	9,08	2,13	13,6	6,28	0,149	0

4,63	100	14,3	2,61	17,5	10,6	0,295	0
4,89	88,6	14,6	2,01	13,2	6,68	0,032	0
5,38	95,3	10,9	3,18	17,8	8,18	0,174	0
3,68	90,6	12	2,06	20,5	8,9	0,254	0
5,43	105	14,3	2,78	16,1	7,7	0,236	0
4,48	95,1	13,6	2,17	16,4	7,79	0,152	0
3,22	79,3	11,4	1,74	10,7	5,94	0,133	0
6,53	109	14,9	3,56	23,2	10,7	0,123	0
6,69	136	15,7	3,76	24,8	11,3	0,288	0
3,1	79,7	10,8	1,54	11,4	5,34	0,092	0
1,36	37,5	4,56	0,793	5,91	2,74	0,062	0
5,01	97	11,3	2,66	18,3	7,14	0,149	0
2,74	73	9,6	1,52	14,1	6,25	0,147	0
3,6	78,5	10,8	1,65	11,4	5,44	0,084	0
4,92	107	12,1	2,82	19,8	8,48	0,201	0
5,21	119	16,6	2,63	16,8	8,26	0,198	0
6,52	112	15,8	3,22	20,5	9,85	0,386	0
4,39	94,3	12,4	2,41	22,5	9,58	0	0
3,99	89	11	1,94	14,2	6,19	0,174	0
2,82	68	8,39	1,5	10,9	5,07	0,114	0
4,79	90,2	11,2	2,43	15,5	6,9	0,221	0
5,88	111	12	3,14	18,1	7,58	0,162	0
3,48	74,1	9,26	1,82	10,8	5,06	0,163	0
2,48	76,2	8,43	1,44	12,5	6,22	0,113	0
4,25	86,1	9,57	2,23	14,7	5,8	0,12	0
3,31	76,8	9,39	2,01	14,8	6,67	0,138	0
2,42	61,6	7,61	1,49	11,2	5,55	0,04	0
5,09	88,9	12,6	2,31	14,4	6,96	0,105	0
4,63	90,2	12,5	2,26	15	7,08	0,081	0
4,8	119	17,7	3,29	28,7	14,4	0,367	0
3,45	77	8,63	1,73	13,6	5,04	0,128	0
2,99	93	9,55	1,54	13,7	4,77	0,082	0
3,58	86,4	8,99	1,71	12,4	4,94	0,202	0
4,77	76,5	9,06	2,53	14,6	5,46	0,096	0
5,49	105	14,8	2,94	19	9,67	0,18	0
4,49	84,5	9,87	1,96	14,8	5,71	0,091	0
5,65	94,9	11,8	2,42	15,4	6,2	0,23	0
4,21	94,9	10,6	1,97	12	5,05	0,168	0
4,31	133	14,9	2,21	17,7	7,45	0,179	0
5,97	137	22,2	2,99	22,6	12	0,34	0
6,28	149	15,6	4,38	26,6	11,5	0,057	0
2,55	61,5	9,02	1,23	11,4	5,07	0,106	0
5,35	102	11,3	3,35	24,9	9,44	0,407	0
4,21	115	14,8	2,73	25,1	11,6	0,306	0
4,58	63,6	10,2	2,49	16,5	7,67	0,271	0
3,49	57,8	8,59	1,94	14,3	6,3	0,215	0
3,13	71,9	7,18	1,95	17,4	5,93	0,072	0
2,83	77,5	10,3	1,93	23,4	8,33	0,222	0
3,81	68	10,3	2,28	17,6	8,27	0,106	0
5,52	112	16,1	2,48	17,6	7,35	0,221	0

6,76	121	14,9	3,68	22,9	10	0,331	0
2,24	67,9	7,48	1,42	16,5	5,42	0,1	0
5,04	127	14,1	2,66	21	8,65	0,158	0
3,37	95,9	10,7	1,82	15,2	6,16	0,197	0
4,53	95,7	11	3,37	26,5	11,3	0,202	0
2,13	64,3	8,22	1,29	12,5	5,49	0,13	0
2,16	73,5	9,58	1,73	22,3	10,5	0,168	0
1,86	77,8	8,85	1,19	18,3	6,63	0,168	0
3,47	90,5	11,3	1,97	16,1	7,36	0,205	0
4,21	96,7	10,8	2,6	22	8,14	0,166	0
1,88	50,8	5,99	1,08	8,97	3,46	0,044	0
5,08	111	13,7	2,86	20,1	8,72	0,185	0
1,47	43,6	5,28	0,836	9,4	3,76	0,052	0
4,82	102	15,6	2,57	17,8	9,31	0,178	0
3,43	87,6	12,9	1,94	16,1	8,5	0,199	0
5,1	104	11,8	2,52	17,5	7,44	0,194	0
1,85	52	8,84	1,02	10,7	6,03	0,159	0
4,33	131	14,5	2,13	17,1	7,29	0,162	0
4,32	2,11	6,4	6,25	0,728	94,6	12,2	14,2
3,14	1,82	7,37	5,27	0,915	79,7	9,17	12,8
3,35	1,85	5,65	5,96	0,488	87,5	12,5	11,8
4,69	2,31	8,07	6,74	0,706	94,5	11,5	16,7
2,59	1,47	5,03	4,2	0,572	55,1	5,98	10,1
3,38	1,73	8,14	5,53	0,653	72,8	8,06	12,4

SM.C18.1	SM.C20.2	SM.C22.3	SM.C24.0	SM.C24.1	SM.C26.0	SM.C26.1	Al	Ni	As	Cd
6,28	0,126	0	8,57	27,2	0,016	0,175	5,99	2,9	0,71	0,985
5,87	0,069	0	9,36	28,7	0,079	0,149	2,89	0,51	0,72	0,85
9,1	0,127	0	11	32,6	0,068	0,16	12,948	5,85	6,92	3,2767
7,09	0,151	0	10,6	32,5	0,096	0,222	6,95	0,71	0,99	1,65
6,91	0,169	0	8,83	37,3	0,063	0,179	6,899	1,14	0,83	1,56
8,62	0,263	0	10,1	37,6	0,081	0,293	5,39	0,912	0,72	0,685
11,4	0,24	0	13	33,5	0,105	0,212	10,788	4,24	8,66	4,025
7,37	0,2	0	11,7	40,6	0,098	0,324	9,458	9,81	9,83	3,552
5,94	0,376	0	20,5	61,6	0,108	0,327	5,79	2,24	0,741	0,658
4,84	0,103	0	12,3	33,7	0,083	0,176	8,838	1,54	2,1228	0,258
8,41	0,322	0	12,4	46,2	0,118	0,229	9,37	3,97	3,588	1,63
7,24	0,164	0	13,4	37	0,066	0,204	2,37	0,94	3,652	0,95
7,68	0,211	0	17,4	38,5	0,153	0,24	11,89	8,58	7,058	4,0598
8,98	0,249	0	13,5	45,1	0,092	0,238	6,73	2,55	5,885	2,55
10,5	0,145	0	11,3	41	0,096	0,243	10,8896	7,53	8,56858	3,02
4,47	0,208	0	14,1	45,2	0,103	0,373	7,37387	1,23	1,158	1,986
6,33	0,144	0	11,3	30,2	0,055	0,223	2,435	4,98	3,555	2,269
9,9	0,139	0	13,7	44,9	0,141	0,24	12,888	9,56	9,0888	4,999
7,45	0,306	0	14,9	42,7	0,11	0,273	4,77	3,43	2,955	0,885
11,3	0,256	0	10,3	39,3	0,059	0,149	13,84	8,38	10,088	4,255
5,99	0,197	0	12,6	40,7	0,117	0,309	6	1,1265	0,525	0,08
6,57	0,117	0	8,1	32	0,083	0,171	4,987	0,893	0,8912	1,58
6,44	0,269	0	14,2	44,2	0,141	0,349	5,99	2,78	0,368	2,43
7,39	0,218	0	10	36,1	0,062	0,197	4,839	4,33	5,858	0,989
6,18	0,169	0	10,5	33,8	0,062	0,18	6,699	2,89	2,099	1,55
6,8	0,182	0	9,02	28,7	0,076	0,262	9,56	1,219	1,4546	1,58
4,55	0,36	0	11,7	45	0,113	0,21	1,566	1,31	3,854	0,358
5,24	0,12	0	12,5	32,1	0,053	0,132	4,858	1,24	2,289	0,0955
9,56	0,109	0	8,22	26	0,057	0,105	10,94	5,14	13,58	3,55
7,77	0,187	0	12,1	43	0,134	0,421	5,8943	0,93	5,29	0,589
6,22	0,141	0	11,8	37,6	0,123	0,287	4,455	1,32	1,06	2,555
9,15	0,358	0	12,9	47,7	0,057	0,248	15,48	9,27	11,1089	4,568
8,4	0,234	0	13,6	40,6	0,077	0,239	5,99	3,88	3,69	1,58
7,7	0,187	0	13,6	42,1	0,095	0,236	6,255	1,02	2,58	0,89
8,65	0,24	0	9,65	43,1	0,039	0,152	12,889	6,78	10,85	5,59656
7,91	7,44	12,5	43,2	0,778	0,074	0,284	15,93	8,04	8,56	4,56
6,49	4,86	13,6	37	0,675	0,109	0,309	9,4	9,48	6,59	5,56
7,08	5,97	10,8	31	0,597	0,076	0,2	12,12	6,13	4,45	4,55
7,09	5,6	13,4	33,9	0,646	0,089	0,13	6,01	7,34	8,78	3,25
8,25	6,85	12,6	38	0,667	0,086	0,257	15,13	8,55	6,45	6,58
12	9,05	13,8	31,4	1,04	0,109	0,188	9,43	2,45	3,68	4,5
6,69	5,51	10,9	27,4	0,639	0,087	0,157	8,68	7,08	9,896	2,78
5,62	5,9	8,35	27,3	0,443	0,085	0,217	6,22	5,21	8,163	3,59
4,63	4,1	9,15	25,7	0,469	0,035	0,172	8,13	5,6	7,26	5,55
7,22	6,54	13,8	42	0,597	0,149	0,394	5,43	7,98	8,56	4,51
7,17	5,73	11,8	33,5	0,723	0,069	0,229	10,68	8,19	5,232	3,02

9,59	10,8	13	53,4	0,809	0,141	0,224	6,22	0,93	1,16	0,09
7,48	6,59	12,6	32,3	0,691	0,089	0,227	2,13	1,27	2,23	0,26
7,4	7,54	9,86	34,2	0,825	0,092	0,206	5,43	1,22	0,998	0,27
8,63	7,41	13,1	46,6	0,568	0,065	0,242	4,68	1,18	0,45	0,85
9,65	8,83	17,9	51,8	0,893	0,116	0,286	6,22	0,32	0,75	0,68
7,68	7,01	11,7	36,3	0,752	0,109	0,302	5,02	1,24	0,25	1,25
5,17	5,42	8,63	28,1	0,448	0,056	0,138	2,45	0,29	0,65	0,31
9,92	8,89	13,8	43,1	1,02	0,138	0,305	4,33	1,28	0,12	0,096
11,3	9,71	16,1	45,9	1,06	0,125	0,337	2,23	1,13	0,18	0,258
5,91	5,17	12,6	31,9	0,568	0,048	0,149	5,37	1,16	0,11	0,093
1,98	2,25	3,14	14,9	0,226	0,023	0,135	5,99	0,59	0,175	0,092
7,54	6,92	11,6	38,9	0,783	0,076	0,277	5,2	1,25	0,123	0,11
4,52	4,08	7,46	29,1	0,416	0,062	0,17	3,95	1,09	0,12	0,095
6,44	5,73	10,1	29,8	0,61	0,092	0,168	6,5	1,21	0,095	0,256
7,7	6,84	11,8	42,3	0,788	0,144	0,297	3,45	0,42	0,093	0,092
9,41	9,23	14,3	43	0,757	0,116	0,27	1,13	1,25	0,096	0,12
8,52	9,5	12	43,1	0,774	0,116	0,238	1,24	1,26	0,11	0,18
10,1	8,54	14,4	44,5	0,804	0,1	0,283	1,19	0,94	0,15	0,11
6,41	6,14	10,2	36,8	0,53	0,086	0,204	1,23	1,03	0,091	0,175
5,04	4,48	8,48	26,9	0,452	0,05	0,183	1,66	1,14	0,12	0,123
7,27	7,48	10,6	38,2	0,604	0,082	0,25	2,15	0,55	0,23	0,12
8,69	7,87	12	40,7	0,947	0,125	0,409	3,26	0,45	0,32	0,095
5,47	5,06	8,5	27,7	0,522	0,104	0,204	2,56	1,72	0,17	0,093
4,67	4,77	7,75	32,8	0,481	0,048	0,124	3,56	1,09	0,15	0,096
7,92	6,42	12,2	38,6	0,742	0,108	0,301	1,24	1,25	0,26	0,711
6,66	5,2	12,4	33,2	0,774	0,109	0,201	3,65	1,32	0,19	0,15
5,93	5,19	9,29	27,8	0,546	0,083	0,143	2,58	0,56	0,158	0,091
8,27	7,17	11	31,7	0,737	0,071	0,186	5,26	1,15	0,188	0,12
7,14	6,64	10,9	33,9	0,575	0,08	0,135	1,3	0,98	0,115	0,23
8,12	7,78	12,9	45,8	0,87	0,083	0,318	2,45	1,43	0,123	0,32
8,84	5,77	16,5	35,5	0,824	0,081	0,229	3,25	0,36	0,34	0,17
6,37	4,62	10,5	39,8	0,522	0,082	0,29	1,2	1,27	0,125	0,15
6,93	5,57	9,62	35	0,704	0,086	0,229	3,66	1,25	0,256	0,26
7,22	6,35	9,96	30,9	0,711	0,107	0,201	2,59	1,13	0,129	0,19
6,87	7,41	10,7	36	0,708	0,086	0,22	1,88	1,32	0,115	0,158
6,46	5,68	9,12	29	0,502	0,076	0,195	4,56	1,22	0,096	0,188
10,2	9,14	14,2	41,7	0,861	0,113	0,242	2,5	1,16	0,086	0,115
8,46	6,76	12,9	38,1	0,772	0,102	0,215	3,58	1,18	0,093	0,123
8,53	7,15	11,9	43,9	0,745	0,09	0,189	2,558	1,25	0,092	0,34
12,2	12	19,4	64,3	1,2	0,156	0,485	3,255	0,63	0,11	0,125
11,8	10,2	18,9	58,5	1	0,154	0,453	4,55	1,43	0,095	0,256
6,16	4,38	10,8	28	0,525	0,055	0,185	5,23	1,37	0,095	0,129
11,2	9,36	15,5	50,3	1,3	0,177	0,496	2,26	1,22	0,092	0,115
5,2	7,94	8,64	63,2	0,635	0,128	0,589	1,23	0,68	0,92	0,021
7,3	6,75	8,31	25,7	0,693	0,063	0,159	3,56	0,68	1,22	0,26
6,63	4,94	8,91	23,1	0,683	0,11	0,234	4,66	0,75	1,46	0,015
4,76	3,91	7,35	26,7	0,583	0,074	0,211	2,25	1,49	0,88	0,034
5,22	4,09	9,86	34,8	0,539	0,068	0,213	3,55	1,84	1,16	0,046
9,03	7	11,1	29,6	0,826	0,063	0,151	2,55	1,21	1,94	0,022
10,2	8,21	17,6	42,9	0,974	0,129	0,2	1,88	0,99	1,18	0,15

10,8	9,27	15,2	47,7	1,04	0,147	0,391	3,22	1,24	1,68	0,018
4,8	3,51	9,82	40,8	0,473	0,07	0,208	2,88	0,85	1,17	0,031
7,53	6,47	12,9	46,6	0,812	0,084	0,316	4,56	1,12	1,52	0,15
5,56	4,89	8,39	38,1	0,521	0,071	0,192	1,66	1,53	1,99	0,034
10,1	7,2	14,9	42,6	0,93	0,13	0,305	5,22	1,33	1,26	0,022
5,6	4,2	9,12	29,1	0,489	0,081	0,212	1,22	1,14	1,31	0,0165
7,28	4,66	13,4	35,1	0,586	0,112	0,248	2,58	1,12	1,35	0,027
4,11	3,76	8,27	47,7	0,574	0,072	0,293	3,56	0,45	1,55	0,023
6,84	6,54	10,4	42,7	0,625	0,107	0,341	1,59	1,6	1,26	0,033
7,3	5,14	12,4	40,7	0,639	0,075	0,223	1,66	0,98	1,22	0,051
4,24	2,96	8	22,9	0,423	0,092	0,17	2,98	1,19	1,67	0,032
9,71	7,42	15,7	46	0,984	0,086	0,354	3,26	0,93	1,23	0,526
3,26	2,2	5,91	14,6	0,218	0,036	0,096	5,66	1,27	1,29	0,019
8,18	8,37	12,4	42,9	0,813	0,09	0,254	4,12	1,22	1,42	0,0198
8,59	6,71	13,4	34,2	0,679	0,094	0,234	3,29	1,18	2,01	0,26
7	6,85	11,1	41,1	0,682	0,1	0,269	3,26	1,32	1,98	0,765
3,42	3,06	5,84	23,8	0,36	0,064	0,159	1,25	1,24	3,29	0,5898
8,07	6,66	11,7	42,9	0,887	0,098	0,185	2,26	1,29	2,69	2,22
6,38	0,149	0	9,72	34,7	0,098	0,201	1,778	0,78	2,52	1,23
5,36	0,236	0	15,2	41,5	0,089	0,321	4,89	1,393	1,66	1,45
6,7	0,156	0	9,15	32	0,045	0,174	2,99	1,587	1,88	0,62
7,18	0,225	0	15,8	38,5	0,09	0,232	2,89	0,83	2,336	1,4374
4,39	0,083	0	7,97	22,9	0,063	0,176	3,09	1,4576	0,89	1,045
4,61	0,164	0	14,3	32,5	0,112	0,178	6,89	4,563	6,9	3,27

Pb
3,285
1,3585
7,295
1,656
0,05522
0,04598
5,5325
4,095
0,458
0,0959632
0,243894
0,566
6,2
0,434
8,03
0,085
0,32589
6,07533
0,9277
7,344
0,05875
0,345
0,245
0,1434
0,194
0,09589
0,0985
0,4
0,0456
0,068954
0,309
0,06569
0,18
0,097
0,09652
4,125
2,825
3,59
3,58
2,89
1,299
0,89
0,59
3,59
1,59
7,56

0,05
0,125
0,089
0,269
0,358
0,59
0,26
0,2
0,095
0,125
0,0975
0,125
0,075
0,225
0,15
0,125
0,125
0,1
0,2
0,1
0,175
0,125
0,1625
0,225
0,095
0,15
0,075
0,175
0,75
0,125
0,175
0,269
0,0958
0,175
0,089
0,59
0,098
0,116
0,1699
0,785
1,69
2,599
0,099
0,59
0,579
0,248
0,2258
0,3549
0,595
0,755

0,0912
0,69
0,599
0,533
1,259
0,298
0,1865
0,246
0,855
0,169
0,266
0,49
0,8796
1,265
0,5588
0,088
0,091
0,79
0,931
0,24
1,0593
0,125
0,1
3,3274
