# Supplementary material for: Application of Human Plasma Targeted Lipidomics and Analysis of Toxic Elements to Capture the Metabolic Complexities of Hypothyroidism
Source: Molecules. 2024 Oct 31;29(21):5169. doi: 10.3390/molecules29215169 (PMC11547455; doi:10.3390/molecules29215169)
Supplement: Supplementary file 1 [file molecules-29-05169-s001.zip › molecules-3242400-supplementary/Tables S1-S8.zip!/Table S7. LODs of toxic elements.docx.pdf]

Table S7. Limits of detection of toxic elements in plasma of the study population (N=120).

Element	Target analyte isotope monitored	LR [μg/L]	Levels	EC	R^2	LOD [μg/L]
Aluminum	27Al	0.1-20.0<br>0.1-100.0	4<br>6	$y = 654.7x + 2210.4$<br>$y = 691.6x + 2549.1$	0.9995<br>0.9997	2.014<br>1.230
Arsenic	75As	0.1-100.0	6	$y = 304.6x + 87.0$	0.9997	0.030
Cadmium	111Cd	0.1-100.0	6	$y = 952.9x + 104.0$	0.9999	0.063
Lead	208Pb	0.1-100.0	6	$y = 12673.3x + 4366.6$	0.9999	0.082
Nickel	60Ni	0.1-100.0	6	$y = 776.6x + 870.4$	0.9999	0.115

LR - linear range of the calibration curve; EC - equation of the calibration curve; y — signal (count/s; CPS); x - concentration of the analyte [μg/L], the value at x is defined as sensitivity; R^2 – correlation coefficient of the calibration curve; LOD – limit of detection (calculated as 3*SD (standard deviation) of 100 blanks)
