# Supplementary material for: Application of Human Plasma Targeted Lipidomics and Analysis of Toxic Elements to Capture the Metabolic Complexities of Hypothyroidism
Source: Molecules. 2024 Oct 31;29(21):5169. doi: 10.3390/molecules29215169 (PMC11547455; doi:10.3390/molecules29215169)
Supplement: Supplementary file 1 [file molecules-29-05169-s001.zip › molecules-3242400-supplementary/Tables S1-S8.zip!/Table S8. Limits of detection of studied lipid species in plasma.pdf]

Table S8. Limits of detection (µM/L) of studied lipid species in plasma of the study population (N=120).

C0		4.83
C2		0.18
C3		0.03
C3-OH		0.04
C3:1		0.01
C4		0.01
C4-OH (C3-DC)		0.02
C4:1		0.04
C5		0.04
C5-DC (C6-OH)		0.04
C5-M-DC		0.07
C5-OH (C3-DC-M)		0.06
C5:1		0.05
C5:1-DC		0.03
C6 (C4:1-DC)		0.09
C6:1		0.04
C7-DC		0.04
C8		0.07
C9		0.04
C10		0.09
C10:1		0.10
C10:2		0.03
C12		0.07
C12-DC		0.15
C12:1		0.11
C14		0.09
C14:1		0.01
C14:1-OH		0.02
C14:2		0.06
C14:2-OH		0.01
C16		0.09
C16-OH		0.02
C16:1		0.04
C16:1-OH		0.02
C16:2		0.02
C16:2-OH		0.02
C18		0.02
C18:1		0.02
C18:1-OH		0.01
C18:2		0.01
lysoPC a C14:0	LPC 14:0	0.43
lysoPC a C16:0	LPC 16:0	0.11
lysoPC a C16:1	LPC 16:1	0.07
lysoPC a C17:0	LPC 17:0	0.01
lysoPC a C18:0	LPC 18:0	0.33
lysoPC a C18:1	LPC 18:1	0.04
lysoPC a C18:2	LPC 18:2	0.09
lysoPC a C20:3	LPC 20:3	0.46

lysoPC a C20:4	LPC 20:4	0.01
lysoPC a C24:0	LPC 24:0	0.05
lysoPC a C26:0	LPC 26:0	0.05
lysoPC a C26:1	LPC 26:1	0.04
lysoPC a C28:0	PC O-28:0	0.11
lysoPC a C28:1	PC O-28:1	0.03
PC aa C24:0	PC 24:0	0.08
PC aa C26:0	PC 26:0	0.70
PC aa C28:1	PC 28:1	0.05
PC aa C30:0	PC 30:0	0.11
PC aa C30:22	PC 30:22	0.01
PC aa C32:0	PC 32:0	0.01
PC aa C32:1	PC 32:1	0.01
PC aa C32:2	PC 32:2	0.01
PC aa C32:3	PC 32:3	0.00
PC aa C34:1	PC 34:1	0.02
PC aa C34:2	PC 34:2	0.08
PC aa C34:3	PC 34:3	0.03
PC aa C34:4	PC 34:4	0.00
PC aa C36:0	PC 36:0	0.11
PC aa C36:1	PC 36:1	0.01
PC aa C36:2	PC 36:2	0.03
PC aa C36:3	PC 36:3	0.02
PC aa C36:4	PC 36:4	0.07
PC aa C36:5	PC 36:5	0.04
PC aa C36:6	PC 36:6	0.02
PC aa C38:0	PC 38:0	0.02
PC aa C38:1	PC 38:1	0.01
PC aa C38:3	PC 38:3	0.01
PC aa C38:4	PC 38:4	0.02
PC aa C38:5	PC 38:5	0.03
PC aa C38:6	PC 38:6	0.01
PC aa C40:1	PC 40:1	0.37
PC aa C40:2	PC 40:2	0.01
PC aa C40:3	PC 40:3	0.00
PC aa C40:4	PC 40:4	0.00
PC aa C40:5	PC 40:5	0.00
PC aa C40:6	PC 40:6	0.03
PC aa C42:0	PC 42:0	0.02
PC aa C42:1	PC 42:1	0.01
PC aa C42:2	PC 42:2	0.06
PC aa C42:4	PC 42:4	0.01
PC aa C42:5	PC 42:5	0.01
PC aa C42:6	PC 42:6	0.07
PC ae C30:0	PC O-30:0	0.09
PC ae C30:1	PC O-30:1	0.01
PC ae C30:2	PC O-30:2	0.00
PC ae C32:1	PC O-32:1	0.01
PC ae C32:2	PC O-32:2	0.01
PC ae C34:0	PC O-34:0	0.01

PC ae C34:1	PC O-34:1	0.01
PC ae C34:2	PC O-34:2	0.01
PC ae C34:3	PC O-34:3	0.01
PC ae C36:0	PC O-36:0	0.03
PC ae C36:1	PC O-36:1	0.02
PC ae C36:2	PC O-36:2	0.02
PC ae C36:3	PC O-36:3	0.01
PC ae C36:4	PC O-36:4	0.02
PC ae C36:5	PC O-36:5	0.01
PC ae C38:0	PC O-38:0	0.09
PC ae C38:1	PC O-38:1	0.01
PC ae C38:2	PC O-38:2	0.00
PC ae C38:3	PC O-38:3	0.01
PC ae C38:4	PC O-38:4	0.01
PC ae C38:5	PC O-38:5	0.01
PC ae C38:6	PC O-38:6	0.02
PC ae C40:1	PC O-40:1	0.02
PC ae C40:2	PC O-40:2	0.00
PC ae C40:3	PC O-40:3	0.01
PC ae C40:4	PC O-40:4	0.05
PC ae C40:5	PC O-40:5	0.01
PC ae C40:6	PC O-40:6	0.01
PC ae C42:0	PC O-42:0	0.39
PC ae C42:1	PC O-42:1	0.03
PC ae C42:2	PC O-42:2	0.01
PC ae C42:3	PC O-42:3	0.01
PC ae C42:4	PC O-42:4	0.30
PC ae C42:5	PC O-42:5	0.49
PC ae C44:3	PC O-44:3	0.04
PC ae C44:4	PC O-44:4	0.04
PC ae C44:5	PC O-44:5	0.02
PC ae C44:6	PC O-44:6	0.17
SM (OH) C14:1	SM 33:1	0.00
SM C16:0	SM 34:1	0.05
SM C16:1	SM 34:2	0.01
SM (OH) C16:1	SM 35:1	0.01
SM C18:0	SM 36:1	0.07
SM C18:1	SM 36:2	0.01
SM C20:2	SM 38:3	0.01
SM (OH) C22:1	SM 41:1	0.01
SM (OH) C22:2	SM 41:2	0.01
SM C22:3	SM 40:42	0.01
SM C24:0	SM 42:1	0.02
SM C24:1	SM 42:2	0.01
SM (OH) C24:1	SM 43:1	0.01
SM C26:0	SM 44:1	0.01
SM C26:1	SM 44:2	0.01
